# Challenges for FAIR-compliant description and comparison of crop phenotype data with standardized controlled vocabularies

**DOI:** 10.1093/database/baab028

**Published:** 2021-05-15

**Authors:** Liliana Andrés-Hernández, Razlin Azman Halimi, Ramil Mauleon, Sean Mayes, Abdul Baten, Graham J King

**Affiliations:** Southern Cross Plant Science, Southern Cross University, PO Box 157, Lismore, NSW 2480, Australia; Southern Cross Plant Science, Southern Cross University, PO Box 157, Lismore, NSW 2480, Australia; Southern Cross Plant Science, Southern Cross University, PO Box 157, Lismore, NSW 2480, Australia; School of Biosciences, University of Nottingham, Sutton Bonington, Leicestershire, LE12 5RD,Nottingham, Nottingham, UK; Institute of Precision Medicine & Bioinformatics, Sydney Local Health District, Royal Prince Alfred Hospital, Missenden Road, Camperdown, NSW 2050, Australia; Southern Cross Plant Science, Southern Cross University, PO Box 157, Lismore, NSW 2480, Australia

## Abstract

Crop phenotypic data underpin many pre-breeding efforts to characterize variation within germplasm collections. Although there has been an increase in the global capacity for accumulating and comparing such data, a lack of consistency in the systematic description of metadata often limits integration and sharing. We therefore aimed to understand some of the challenges facing findable, accesible, interoperable and reusable (FAIR) curation and annotation of phenotypic data from minor and underutilized crops. We used bambara groundnut (*Vigna subterranea*) as an exemplar underutilized crop to assess the ability of the Crop Ontology system to facilitate curation of trait datasets, so that they are accessible for comparative analysis. This involved generating a controlled vocabulary Trait Dictionary of 134 terms. Systematic quantification of syntactic and semantic cohesiveness of the full set of 28 crop-specific COs identified inconsistencies between trait descriptor names, a relative lack of cross-referencing to other ontologies and a flat ontological structure for classifying traits. We also evaluated the Minimal Information About a Phenotyping Experiment and FAIR compliance of bambara trait datasets curated within the CropStoreDB schema. We discuss specifications for a more systematic and generic approach to trait controlled vocabularies, which would benefit from representation of terms that adhere to Open Biological and Biomedical Ontologies principles. In particular, we focus on the benefits of reuse of existing definitions within pre- and post-composed axioms from other domains in order to facilitate the curation and comparison of datasets from a wider range of crops.

**Database URL**: https://www.cropstoredb.org/cs_bambara.html

## Introduction

Technological advances in data acquisition have driven massive increases in the accumulation of crop trait data and increased the potential for comparative analysis. Trait data that describe phenotypes underpin pre-breeding efforts to characterize variation within germplasm collections, including genomic analysis of phenotype–genotype associations (1). Although trait data tend to be disseminated via publications, or stored within institutional and consortia data repositories (2), the systematic description of associated metadata often lacks consistency (1). This issue has been recognized and addressed by a number of initiatives, including the recently updated Minimal Information About Plant Phenotyping Experiment (MIAPPE) (3) (www.miappe.org) and the Breeding Application Programming Interface (BrAPI) (4).

Unfortunately, reuse of trait datasets for characterization of plant genetic resources continues to be limited by a lack of standardization in trait names, particularly between crops. These are often divergent, originating from independent descriptive vocabularies adopted by breeders, researchers and genetic resource managers (5). The lack of standardization inhibits the organization, integration and sharing of associated data (6, 7) and reduces the potential for extensive genome-wide association and other comparative studies.

Recent efforts to standardize biological data have increasingly become aligned to the generic findable, accessible, interoperable, and reusable (FAIR) principles (8). These are guiding wider adoption of standardized and integrated information, facilitating the reuse of data with minimal human intervention (9, 10). The use of controlled vocabularies such as ontologies helps facilitate programmatic yet intelligent data access and exchange (11). MIAPPE provides a metadata framework for associating phenotype data with details of project, study, experimental design and environmental conditions (3). Originally developed and proposed with broad consultation of researchers and breeders (12), recent updates led to registration of the Plant Phenotype Experiment Ontology (PPEO) (13) (http://purl.org/ppeo). Although the formal MIAPPE data model is expected to contribute to the wider adoption and reuse of experimental metadata, formal definitions and relationships within PPEO (http://agroportal.lirmm.fr/ontologies/PPEO) do not directly reuse terms from other ontologies, limiting scope for machine readability and inference.

A number of initiatives have aimed to establish standardized crop trait names. Although some are associated with formalized metadata, none adhere to a fully integrated ontological system. The International Union for the Protection of New Varieties of Plants or UPOV (http://www.upov.int) descriptor lists are less relevant here, as they are used primarily to describe botanical traits for establishing distinctness, uniformity and stability of new cultivars (14–16). Apart from GRIN (https://www.grin-global.org/userdocs.htm#obs), crop descriptor lists (CDLs) (https://www.bioversityinternational.org/e-library/publications/detail/developing-crop-descriptor-lists/) curated by Bioversity International (https://www.bioversityinternational.org/) are the most prominent. CDLs were initially promoted for evaluation of *ex situ* plant genetic resources and led to the generation of Trait Dictionaries (TDs) used by the Crop Ontology (CO) system (17). TDs are controlled vocabularies generated for specific crops. TD trait names may correspond to single descriptors from existing CDLs and be associated with metadata including methods and scale (17). Within MIAPPE, the CO provides metadata for phenotypic trait and environmental observed variables.

The CO system aims to harmonize trait descriptors for individual crops as measured by breeders, researchers and genetic resource managers. However, due to inconsistencies, both in use and categorization of terms (1, 18), the scope for comparative analyses between crops is limited. This is of particular concern for minor and underutilized crops and so motivated a deeper examination of the CO system in order to identify possible improvements.

The challenge for comparison of crop traits starts with the collection and curation of datasets. Various database platforms have been developed (19, 20), with an increasing number compatible with BrAPI (4), contributing to interoperability. CropStoreDB is a relational schema for explicit management of data and metadata relating to plant experimental genetic resources, traits, trials and associated genetic information (21, 22). This database has been used within the interoperable InterStoreDB for linking crop genetic and genomic information (22) and underpins the interactive Brassica Information Portal (23), for which the API has some BrAPI compatibility. CropStoreDB use-cases have also been developed for biomass crops, commercial tea tree oil (*Melaleuca*) and hemp (*Cannabis*), as well as for *Macadamia* nut genetic mapping and associated populations (24) (https://cropstoredb.org/cs_macadamia.html).

We wished to understand how phenotypic data may be curated so that they adhere to FAIR criteria and facilitate comparison of different crops. We used bambara groundnut (*Vigna subterranea*) as an exemplar underutilized crop (25), to assess the ability of the CO system to facilitate curation of trait datasets so that they are accessible for comparative analysis. We outline the challenges in assembling and using a crop-specific TD and quantify the extent to which crop-specific COs derive knowledge from existing ontological definitions and relationships. Having assigned CO terms to trait descriptors, we then evaluated the MIAPPE and FAIR compliance of datasets curated within the CropStoreDB schema. We highlight systematic limitations of the CO system and suggest a more robust and generic approach to establishing controlled vocabularies associated with different aspects of crop phenotypes. In particular, we focus on the benefits of reuse of existing definitions within pre- and post-composed axioms from other domains in order to facilitate the curation and comparison of datasets from a wider range of crops.

## Materials and methods

TDs for 28 crops managed within the CO system were downloaded in csv format from the CO web portal (September, 2019) and compiled into a single spreadsheet (integrated multi-species TD, Supplementary Table S1). This enabled identification of inconsistencies in syntactic structure of semantically equivalent trait names (Supplementary Table S1). Since different methods and scales may be associated with the same trait name for a given crop, trait name entries duplicated within a TD were removed. Trait names present in one or more TD were retained.

### Consistency analysis

**Figure 1.**
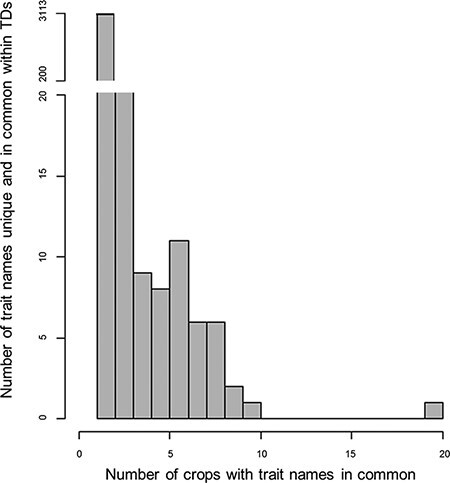
Histogram for the counts of trait names within the 28 Trait Dictionaries (TDs). The histogram represents 3627 trait names within the TDs, along with the number of trait names across the TD for the 28 crop species. The gap in the data representing trait names that are repeated one or two times across the TDs was not plotted in the histogram; for more information, refer Supplementary Table S2 table.

**Figure 2.**
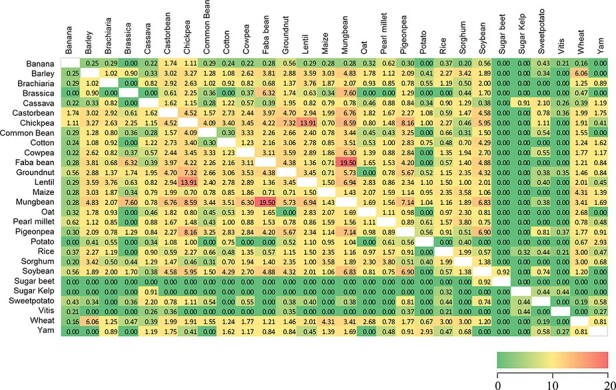
Similarity heatmap for the shared trait names across 28 Trait Dictionaries (TDs) in the Crop Ontology. Values were calculated using the ‘simple matching coefficient’, colour gradient shading is relative to the pairwise percentage of trait names shared across the 28 TDs, with red indicating high values and green zero (Supplementary Table S3).

To quantify standardization and reusability of trait names between TDs, frequency and similarity analyses were performed. Identical trait names shared by the 28 TDs were identified by direct string matching (Supplementary Table S2), occurrence frequencies calculated (Figure 1) (‘Grand total’ column in Supplementary Table S2) and a similarity matrix (Supplemental Table S3) generated (Supplementary Table S2) using the simple matching coefficient (SMC) (26) of shared trait names between each pair of crops (Equation 1) (Figure 2).

(1) \begin{equation*}SMC = {{{N_{shared}}} \over {{N_{total1}} + {N_{total2}}}} \times 100\end{equation*}

Equation 1: Simple matching coefficient was calculated from pairwise *N*_shared_ trait names shared between crops, divided by the trait name totals for each crop.

As a preliminary assessment of semantic equivalence, the number of TD terms having formal cross-reference to other ontologies was determined.

### Curation of existing descriptor lists

**Figure 3.**
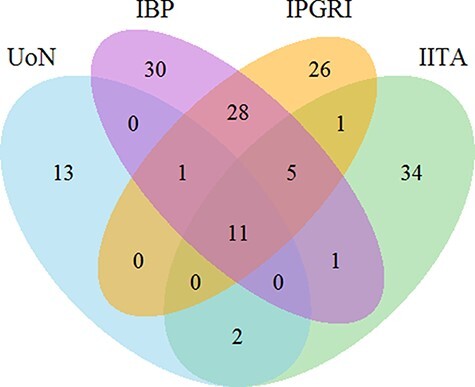
Venn diagram for the count of 230 trait names in bambara groundnut unique and shared across the different institutions. The numbers show the number of unique and shared trait names across the different institutions. Abbreviations in the sets are as follows: University of Nottingham (UoN), Integrated Breeding Platform (IBP), International Plant Genetic Resources Institute (IPGRI) and International Institute of Tropical Agriculture (IITA).

Phenotypic trait descriptors for bambara groundnut were curated (Table 1). Trait names from datasets 1, 2 and 3 (Table 1) were pre-processed to resolve redundancy due to abbreviations, orthography (spelling) or syntax (e.g. word order and order of words). By default if other descriptors were semantically equivalent, the trait name as published in the IPGRI CDL for bambara groundnut (Table 1) was adopted. Name matching between datasets (Table 1) was quantified using an exact string matching routine implemented in R (using the ‘value matching operator’) (27). Counts of exact matches were presented (Figure 3) using the ‘Venn.diagram’ R function (27).

**Table 1.** Datasets used to develop the Trait Dictionary (TD) for bambara groundnut

Dataset	Description	Total trait names
Dataset 1(IPGRI)	Characterization of the crop descriptor list for bambara groundnut from the International Plant Genetic Resources Institute (IPGRI) (https://www.bioversityinternational.org/fileadmin/_migrated/uploads/tx_news/Descriptors_for_Bambara_groundnut__Vigna_subterranea__324.pdf)	73 trait names
Dataset 2 (UoN)	Dataset from Crops For the Future (CFF) from the University of Nottingham (UoN)	27 trait names
Dataset 3 (IITA)	Information from the webpage of the International Institute of Tropical Agriculture (IITA) (http://my.iita.org/accession2/collection.jspx?id=8)	54 trait names
Dataset 4 (IBP)	Trait Dictionary developed by the Integrated Breeding Platform (IBP) in the template v5 (https://www.cropontology.org/)	76 trait names
Multispecies TD	In total, 28 crop-specific Trait Dictionaries were consolidated into a file from the Crop Ontology (brachiaria, cassava, castor bean, chickpea, cowpea, groundnut, lentil, maize, mungbean, pearl millet, pigeon pea, potato, rice, sorghum, soybean, sugar kelp, sweet potato, wheat and yam)	4631 trait names 3627 without duplicated trait names within species

Trait descriptor names were conflated into a single TD Excel spreadsheet (.xlsx) with additional metadata curation (Table 1). New variable names and trait name classification followed guidelines from the CO website. The provisional TD was checked and validated by colleagues with domain expertise prior to submission to the CO curation team using the CO curation tool. This created the unique CO root (CO_366) and term identifiers (e.g. CO_366: 0000181) for the four concepts (variable, trait, method and scale) (Supplementary Table S4).

To ensure that the TD, CO and MIAPPE follow the FAIR principles, specific examples and descriptions of each principle from the GO FAIR webpage (www.go-fair.org/fair-principles) were addressed and tabulated.

## Results

### Harmonization of bambara trait names

Collation of bambara groundnut trait descriptors from four sources indicated only 11 string-matched terms in common from a total of 230 (Figure 3; Table 1). Mutually exclusive trait descriptor names were identified with each source, with 80 failing to coincide with the reference IPGRI CDL. For example, ‘Number of leaves’ defined in the IPGRI CDL was semantically equivalent to ‘Leaf number per plant’ within the Integrated Breeding Platform (IBP) dataset and also equivalent to ‘Number of leaves per plant’ (International Institute of Tropical Agriculture) and ‘Leaf number’ (University of Nottingham) (Supplementary Table S6).

### Assigning descriptors to a TD

In order to assign descriptor names to the formal CO-TD, we first evaluated the ease with which terms, definitions and relationships could be reused within the existing CO system and derive information from external ontologies. Comprehensive examination of the 28 CO-TDs (Supplementary Table S1) indicated 4739 crop–trait name combinations, which reduced to 3627 after removal of within-crop duplicates based on string matching. String-matching also indicated that ‘Plant height’ was the most frequent trait name shared by 20 crops, followed by ‘Harvest index’ (10 crops), ‘Drought tolerance’ and ‘Seed weight’ (nine each) (Supplementary Table S2). In total 514 duplicated trait names occurrences were removed. The frequency distribution (Figure 1) of the remaining 3113 trait names, based on occurrence in the 28 TDs, clearly demonstrates that the majority (90%) are not shared between TDs.

To determine which crops share the most and least exact string-matched trait names, a simple matching coefficient was calculated for each pair of TDs, after removing string-matched duplicated trait names for each crop. In general, consistency between the crops was low. Indeed, the highest matching coefficients were associated with the legumes mungbean and faba bean, each sharing 20% of the terms (Figure 2), with reduced similarity shared between lentil and chickpea (14%) as well as mungbean and pigeon pea with chickpea (8%) (Supplementary Table S3). By comparison, the TDs for sugar beet, sugar kelp and *Vitis* have the fewest (0–0.4%) trait terms in common.

Similarity analysis indicated that the proportion of trait names shared between crops is not always associated with taxonomic relatedness (Figure 2), although some effect is apparent due to single or common groups of institutions being involved in the generation of CO:TDs. This is evident for groundnut, chickpea, pigeon pea and mungbean, primarily described by one institution. Conversely, the legumes soybean and cowpea both included entries from the same institution but only shared 2.7% similarity among trait names.

### Semantic classification

In addition to string matching, we assessed semantic equivalence (distinct vocabulary and similar meaning) by sampling a subset of trait names from the TDs (Table 2). Ambiguity was observed both within the semantic content of the higher-level trait classes and in the classification of trait names. For example, within the class ‘quality’, different TDs had inconsistent labels for the semantically equivalent sub-classes ‘quality trait’, ‘quality’ and ‘quality traits’. Likewise, within the ‘biochemical’ class, semantically equivalent ‘biochemical trait’, ‘biochemical’ and ‘biochemical traits’ appear in different TDs. Moreover, assignment of some trait names to trait class was inconsistent in different TDs. For example, ‘seed protein content’ was assigned to the ‘biochemical traits’ class for soybean, ‘quality traits’ for chickpea and the ‘quality trait’ class for mungbean (Supplementary Table S1). While these latter assignments may potentially reflect different roles or priorities within a breeding context, seed protein content within ‘quality trait’ could benefit from the reuse of the ‘biochemical’ concept of protein within its formal definition, along with the reuse of a formal definition of seed (e.g. from PO) and indeed of concentration (from Phenotype and Trait Ontology, PATO) (28). We also considered a more extensive analysis of semantic equivalence where external Plant Ontology (PO) and Trait Ontology (TO) terms had already been assigned as cross-references following the process outlined by Laporte *et al.* (18). However, we concluded that more extensive analyses would require thorough reconfiguration of the ontology.

**Table 2.** Examples of inconsistencies for specific trait names from the 4739 Trait Dictionaries in the Crop Ontology

Crop	Trait name
Pearl millet	100 grain weight
Pigeon pea	Weight of 100 seeds
Castor bean	Hundred seed dry weight
Castor bean	Leaf number
Rice	Leaf total number
Yam	Number of leaves
Cassava, maize, yam, sorghum, groundnut	Leaf color
Cowpea, mungbean	Leaf colour
Lentil	Number of seeds per pod
Chickpea	Seeds per pod
Cowpea	Seed per pod
Groundnut	Pod seed number

We also assessed semantic equivalence by counting cross-references to external ontologies within the integrated multi-species TD (Supplementary Table S1) and found only 12.6% of trait names (392) referenced, primarily to the PO (29) and TO (30) (Supplementary Table S1). These were confined to the soybean, chickpea, rice, yam and brassica TDs.

Following discussions and feedback from the CO curation team, the agreed vocabulary for the TD_bambara_ was formally submitted and published online as an ontology with crop code ‘CO_366’. The TD_bambara_.csv text file (https://www.cropontology.org/ontology/CO_366/Bambara%20groundnut) contains 134 variable_names. Of these, 130 were cross-referenced to PO and TO and 76 (57%) coincide with other CO:TDs (Supplementary Table S4).

### Implementation of MIAPPE metadata standards

We determined the extent to which individual or multiple data fields within the generic crop curation relational database CropStoreDB v. 9.2 complied with terms outlined in the MIAPPE v1.1 schema (Supplementary Table S5). In compliance with FAIR principles (31), the MIAPPE data model recommends a minimal set of explicit mandatory information to be recorded for plant experiments. This includes metadata describing investigation and study, identifiers of people involved, geographic location, organism, biosample and description of the experimental design. Although we demonstrated that MIAPPE helped manage different experimental information, we found it important first to understand the one-to-many relationships within the MIAPPE schema. Our assessment indicated that most data fields within the CropStoreDB table ‘plant_trials’ specify metadata information related to location, design factors and project descriptor of the experiment. These were aligned to data fields within the study section of the MIAPPE schema. Although most CropStoreDB metadata fields corresponded with MIAPPE terms, the MIAPPE concepts data file, environment, experimental factor and event were not fully represented.

### FAIR compliance

On the basis of this combination of standardization and curation activities involving TDs, CO MIAPPE and data entry to CropStoreDB, we carried out a qualitative assessment of phenotypic trait data against criteria for compliance with FAIR principles (Supplementary Table S7).

#### Findable

The criteria are met when datasets and associated metadata are easy to find, for both humans and machines (11). This includes assigning persistent identifiers such as digital object identifiers (DOIs) or handles, ensuring they are findable through disciplinary discovery portals. Variable names within the TD each have a globally unique CO identifier associated with searchable trait name, method and scale, with a cross-reference to the CO identifier in the corresponding trait descriptor table of CropStoreDB. Additionally, there is scope to mint DOIs for specific datasets described with indexed metadata fields that meet the MIAPPE standards.

#### Accessibility

This criterion is dependent on the ease with which standardized (machine) protocols may access data records and datasets, along with clarity relating to data status, ownership and licensing arrangements governing access and reuse. At present, CropStoreDB_bambara_ is available via human interaction with an online GUI, allowing advanced filtering of datasets and records. Each CropStoreDB crop database is accessed via a stable URL (http://www.cropstoredb.org/). CropStoreDB is building on existing RESTful JSON web services implemented for the Brassica Information Portal (23), which has demonstrated BrAPI compliance for some entities. The CropStoreDB schema enables record-level declaration of data provenance, ownership and status (e.g. pre-published, published and private). CropStoreDB_bambara_ data are publicly accessible under the Creative Commons CC BY 4.0 license.

#### Interoperability

Was a central concern, as it involves ensuring that data adopt community agreed formats, languages and vocabularies. This extends to the description of metadata meeting community agreed standards and vocabularies and incorporating unique ID cross-references to related information. Adoption of the community metadata standards provided by MIAPPE provides a substantial contribution to ensuring interoperability. The adoption of the CO:TD system for collating trait descriptors adhering to a controlled vocabulary was a key step, although with clear limitations in the consistency, structure and scope of the CO system itself. We found that establishing a pipeline involving string-matching and evaluation of semantic equivalence is an important step in reducing descriptive redundancy. However, this fails to resolve the wider problems inherent to the CO system as currently configured, which limit direct comparison of trait data for bambara groundnut with other legume or grain crops. The poor congruence between TDs in the CO system requires a comprehensive review with wider community engagement.

#### Reusability

Reuse of data is dependent upon retention of initial richness and granularity and is facilitated by clear machine-readable license and provenance information on how data were generated and processed. The CropStoreDB database allows management and reuse of data at the level of individual records with metadata relating to provenance, ownership and status facilitating subsequent processing of data subsets. Adherence to MIAPPE standards contributes contextual richness, with additional cross-referencing to other ontology systems and terms. The adoption of the discipline-specific CO:TD allows for some reuse. However, the relatively poor congruence limits reuse for direct comparison of data between crops. The TD_bambara_ itself can be used without licensing restrictions, and the corresponding CO may be downloaded as an OWL file. The additional discipline-specific data and metadata standards already described provide additional rich contextual information to facilitate reuse.

## Discussion

We carried out a detailed analysis of the requirements for curating trait data from the underutilized crop bambara groundnut within a FAIR compliant database. This use-case highlighted generic limitations of controlled vocabularies available for describing and comparing crop phenotypes. Our first step was to collate trait descriptors for bambara groundnut in order to establish a TD for this crop. This demonstrated that divergent trait names from different sources often share semantic equivalence. We then investigated this issue in greater depth through evaluation of the CO system, focusing on generic issues that currently limit comparison of trait data between crops. We found that semantically equivalent controlled trait descriptor vocabularies within CO are not harmonized or syntactically consistent (Table 2), which limits reuse for comparative analysis. Although greater cohesion and consistency is seen among a subset of legume crops, this is also incomplete. Indeed, only one trait name (plant height) was shared by 20 of the 28 available TDs (Supplementary Table S2).

Comparison and reuse of phenotypic trait data are limited by the ability to which any combination of different words may accurately convey precise information (32). Unfortunately, the definition of CO trait_name terms used in the different crop TDs does not appear coordinated. Syntactical variation was evident across the 28 CO:TDs where trait names were semantically equivalent. For example, the equivalent of ‘100-seed weight’ for soybean was described variously as ‘100-grain weight’ for pearl millet, ‘Weight of 100 seeds’ for pigeon pea and ‘hundred seed dry weight’ in the castor bean TD (Supplementary Table S1). Likewise, the number of plant leaves was found as ‘Leaf number’ in castor bean, ‘Leaf total number’ in rice and ‘Number of leaves’ in yam (Table 3). Another limitation to reuse of semantically equivalent terms is the simple issue of inconsistent orthography between British and American English. For example, to describe ‘leaf color’ and ‘leaf colour’, controlled vocabulary systems have to ensure that both descriptions are associated with databases via a single specific ID. This and direct synonyms (e.g. plant ‘hairs’ and ‘trichomes’) could be addressed by adopting embedded semiautomated look-up internationalization and thesaurus software (33).

**Table 3.** Trait names from bambara groundnut shared across the four datasets from different institutions

Trait names	Description
Terminal leaflet length	Shape of the terminal leaflet
Terminal leaflet width	Width of the terminal leaflet
Internode length	Length of internode
Petiole length	Length of the petiole
Peduncle length	Length of peduncle
Plant height	Height of the plant
Pod length	Length of the pod
Pod width	Width of the pod
Seed width	Width of seeds
Seed length	Length of the seed
Shell thickness	Thickness of the shell

The lack of harmonization between the CO:TDs has been recognized (5), as has the subsequent limited integration, interoperability and thus reuse of crop phenotypic data for comparative analysis (34, 35). As described in our preliminary analysis of the CO system (1), inconsistencies also exist at the level of classification and in semantic equivalence of the trait classes (Supplementary Table S1). This represents a significant challenge for comparative analyses between crops. The eight major trait classes (abiotic stress, biotic stress, agronomical, biochemical, morphological, phenological, physiological and quality) within the crop-specific COs are presented with categorical equivalence and have limited sub-class allocations that would be meaningful to breeders and researchers and facilitate more systematic data mining. In contrast, PATO (35) has a maximum sub-classification depth of 12, PO 10 and TO 9.

Increasing cross-referencing enhances connectivity and linguistic precision, recognized as key outcomes for cost-effective and high-quality ontologies (36–38). Within the CO system, this would not only increase the semantic interoperability (standardization) of trait descriptor terms used for each crop but also facilitate the direct comparison of phenotypic traits scored for different crops. However, the 28 crop-specific CO:TDs were mostly developed independently in order to facilitate exchange and comparison of data within breeder communities. However, the relatively superficial definitions based on existing breeders’ trait names often conflate concepts. This limits reuse and opportunities for downstream comparative analysis, as does the lack of formal definitions based on axioms where pre-composed entities and qualities incorporate external ontology terms and relationships. Although it appears that many trait names may be semantically equivalent across most of the TDs (Table 2), there is no evidence of explicit cross-referencing or attempt to check orthography and string-matching. The Planteome initiative has made some progress over the past 5 years in assigning PO (entity) and TO (quality) terms as external references (xrefs) for a small proportion of traits in a subset of COs (18, 29). As with MIAPPE, unfortunately these do not represent embedded terms within formal axioms or definitions. While ‘linking’ terms with xrefs adds to information content, when used alone this approach fails to make full use of the capability of a logically consistent and well-formed Open Biological and Biomedical Ontologies (OBO) ontology (39), where terms are reused from external ontologies within the formal definitions of terms themselves (40). This reduces ambiguity and provides considerably greater depth of knowledge, accessible for both human use and machine learning inferences.

**Figure 4.**
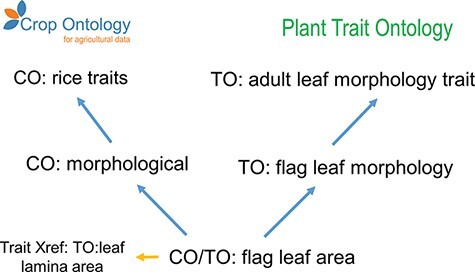
Example of granularity improvement for the CO for the ‘Flag leaf area’ term. Blue arrows represent the ‘is_a’ relationship. Abbreviations are related to existent ontologies: Crop Ontology (CO), Plant Trait Ontology (TO), Plant Ontology (PO), Phenotype and Trait Ontology (PATO) and Basic Formal Ontology (BFO).

We investigated an example of where the CO appears to benefit from cross-referencing (Figure 4). Unfortunately, the specific CO implementation for this example in rice appears flawed, as ‘flag leaf area’ [CO_320:0001075] is cross-referenced to the term ‘leaf lamina area’ [TO:0000827], rather than the term ‘flag leaf area’ [TO:0000996], which is available as a sibling term within the TO subclass ‘plant structure morphology trait’ [TO:0000839]. Adopting a cross-reference to the latter option would provide a richer conceptualization for the crop trait, as through correct reuse it would inherit from TO the concepts of ‘flag leaf area’ is a ‘flag leaf morphology’ as well as ‘flag leaf area’ is a ‘morphology trait’. This example also demonstrates the need for periodic review and update of cross-referenced terminologies.

Ontology systems gain value through their application and use (41–44). The need for more inclusive domain-community involvement in establishing controlled vocabulary systems is a generic and challenging issue (42). We suggest there is scope to establish a more generic and extensible controlled vocabulary for crop traits that adheres to the principles of OBO (45) from the outset, particularly the requirement to ensure orthogonality between ontologies by avoiding duplication of term definitions (46). To be applicable to a wide range of crops would require considerable effort in generating robust classifications of well-defined terms and relationships, incorporating wherever possible genus-defferentia definitions that themselves reuse existing terms (40). For the crop genetic resources, plant science and breeding communities, this would require balancing the reuse of term definitions and categorical relationships that are sufficiently explicit, reviewed and maintained by relevant experts.

A consistent, systematic and rich set of ontological terms, definitions and explicit relationships contribute to reuse (46) and to the quality and interoperability of data mining software applications (47) within deep machine processable systems. More specifically, development of a second-generation crop trait ontology system would be compatible with developments within the International Plant Phenotyping Network and the EMPHASIS consortium (6). To be applicable to a wider range of crops, specifications would include establishment of a universal, systematic and deeper set of ontology sub-classes that relate to TO and other relevant ontologies [Supplementary Table S4 of (1)]. Since the interests of crop breeders, researchers, end users and other stakeholders of plant genetic resources extend far beyond botanical properties and typically involve a complex vocabulary spanning diverse aspects/classes (48), this would require meaningful domain-specific sub-class definitions and relationships. Each trait domain may use distinct or overlapping domain-specific vocabulary, concepts and understanding of relationships between concepts and terms. Specialist language may be associated with crop production, agronomy and quality assessment throughout the production, processing and supply chain for different food and non-food uses (49). The latter is likely to require extensible trait classes that reflect diverse physical, chemical, biological and process attributes, as well as functional interactions relating to raw materials and derived products as they move from crop to pre- and post-processing, storage and end use.

Developing generic crop trait vocabularies would require careful consideration of axioms, in order to generate definitions that explicitly incorporate concepts such as ‘material entity’ from the Basic Formal Ontology (BFO) [BFO:0000040] and PATO ‘quality’ [PATO:0000001] as pre-composed terms. An example would be ‘seed coat color’, which extends the TO definition of a ‘plant_trait’ [TO:000038], as a measurable observable characteristic relating to a ‘plant anatomical entity’ [PO:0025131] or ‘plant structure developmental stage’ [PO:0009012]. This may be complemented by definition of post-composed crop- and domain-specific machine readable axioms and terms that maximize reuse from other domain-specific ontologies (50). As an example, the Crop Dietary Nutrition Ontology (CDNO) (1) is registered within the OBO foundry (http://purl.obolibrary.org/obo/cdno) and has benefited from multidisciplinary consultation between domain specialists including plant chemists and curators of food composition databases. The CDNO reuses terms from the OBO-registered Chemical Entities for Biological Interest (51), PATO, PO and Environmental Ontology (52).

For the curation of specific datasets, precomposed CDNO terms such as ‘concentration of caffeic acid’ [CDNO:0200243] can then be combined in a post-composed design pattern with terms reused from Food Ontology (FoodOn) (53) such as part of ‘coffee bean’ [FOODON:03301477], which itself reuses terms from PO and the NCBI organismal classification ontology (NCBI Taxon) (54). Ensuring that bambara groundnut data and metadata adhere to FAIR principles demonstrated the value of using the MIAPPE v1.1 terms and relationships. It also indicated that incorporating additional metadata categories within the CropStoreDB schema would increase reusability of datasets. The CO:_bambara_ and CropStoreDB_bambara_ resources are now available as platforms for accumulating a wider range of phenotypic data, allowing access through CO-compliant crop search portals such as AgTrials (http://www.agtrials.org/), AgroPortal (http://agroportal.lirmm.fr/) and CropStoreDB.

The use of metadata standards such as MIAPPE is critical for sharing and comparing phenotypic trait data and their associated experimental designs and environmental variables and facilitate adherence to any of the FAIR principles (55). MIAPPE standards are increasingly being adopted in published datasets that make use of data management and interchange frameworks such as BrAPI (4) (https://www.brapi.org/), IBP/Breeding Management system (https://bmspro.io/), Germinate (19) (https://germinateplatform.github.io/get-germinate/) as well as the Brassica Information Portal (23) (https://bip.earlham.ac.uk/) that is based on the CropStoreDB schema (http://www.cropstoredb.org/).

## Conclusion

Development of the TD_bambara_ and curation of publicly available datasets for an underutilized crop meeting FAIR criteria represents a significant advance for this crop. The exercise provided the opportunity to identify issues of generic relevance for integration and comparison of crop trait and related metadata. Of major concern within the CO system were inconsistencies in trait name assignments that limit reuse for a wider range of crops, a flat ontological structure for classifying traits and a relative lack of cross-referencing to other ontologies.

We discuss specifications for a more systematic and generic approach to crop trait–controlled vocabularies that would benefit from representation of terms that adhere to OBO principles. A second-generation crop trait ontology system should focus on the benefits of reusing existing definitions within pre- and post-composed axioms from other domains in order to facilitate the curation and comparison of datasets from a wider range of crops. Such an effort requires carefully managed and extensive consultation with concerted support and involvement. The sociology of pre-breeding characterization, *ex situ* genetic resource management and crop plant breeding is complex. However, efforts by communities of practice such as DivSeek International (https://divseekintl.org/) along with emerging tools and standards such as BrAPI and MIAPPE should ensure the practical, economic and humanitarian benefits of post-genomic predictive crop breeding.

## Supplementary Material

baab028_Supp

## Data Availability

The datasets used for this study are available in CropStoreDB, CO and in the supplementary data.
