# Supplementary material for: Challenges for FAIR-compliant description and comparison of crop phenotype data with standardized controlled vocabularies
Source: Database (Oxford). 2021 May 15;2021:baab028. doi: 10.1093/database/baab028 (PMC8122365; doi:10.1093/database/baab028)
Supplement: baab028_Supp [file baab028_supp.zip › SupplementalTable6.pdf]

Supplemental Table 6. Example of semantic equivalent phenotypic traits across the data sources from the four institutes Integrated Breeding Platform (IBP), International Institute of Tropical Agriculture (IITA), Plant Genetic Resources Institute (IPGRI), University of Nottingham (UoN).

**IBP**	**IITA**	**IPGRI**	**UoN**
Leaf number per plant	No of leaves per plant	Number of leaves	Leaf number
Pod number per plant	Pods per plant	N/A	Pods per plant
Seed number per pod	Seeds per pod	N/A	
Hundred seed adjusted weight	100 Seed weight	100-seed weight	N/A
Node number per stem	No of nodes per stem	Number of nodes per stem	N/A
Maturity time	Days to Maturity	Number of days from planting to maturity	Days to Maturity
Branch number per stem	No of branches per stem	Number of branches per stem	N/A
Terminal leaflet colour	Terminal Leaflet Colour	Colour of fully expanded terminal leaflet	N/A
Pod colour	Pod color	Pod colour	N/A
N/A	Days to 50% flowering	Number of days from sowing to 50% flowering	Days to 50% Flowering
