# Supplementary material for: Challenges for FAIR-compliant description and comparison of crop phenotype data with standardized controlled vocabularies
Source: Database (Oxford). 2021 May 15;2021:baab028. doi: 10.1093/database/baab028 (PMC8122365; doi:10.1093/database/baab028)
Supplement: baab028_Supp [file baab028_supp.zip › SupplementalTable7.pdf]

Supplemental Table S7. Indicative mapping of FAIR principles (https://www.go-fair.org/fair-principles/) to the MIAPPE, Trait dictionary (TD), and CropStoreDB. Boxes marked as checked are the principles that are covered by the TD or MIAPPE.

**Findable**	**CropStoreDB**	**MIAPPE**	**Trait Dictionary**
F1. (meta)data are assigned a globally unique and persistent identifier	✓	✓	✓
F2. data are described with rich metadata	✓	✓	✓
F3. metadata clearly and explicitly include the identifier of the data it describes	✓	✓	✓
F4. (meta)data are registered or indexed in a searchable resource	✓	✓	✓
**Accessible**			
A1. (meta)data are retrievable by their identifier using a standardized communications protocol	✓	✓	✓
A1.1 the protocol is open, free, and universally implementable	✓	✓	✓
A1.2 the protocol allows for an authentication and authorization procedure, where necessary	✓	✓	✓
A2. metadata are accessible, even when the data are no longer available	✓	✓	✓
**Interoperable**			
I1. (meta)data use a formal, accessible, shared, and broadly applicable language for knowledge representation.	✓	✓	✓
I2. (meta)data use vocabularies that follow FAIR principles	✓	✓	✓
I3. (meta)data include qualified references to other (meta)data	✓	✓	✓
**Reusable**			

R1. meta(data) are richly described with a plurality of accurate and relevant attributes	✓	✓	✓
R1.1. (meta)data are released with a clear and accessible data usage license	✓	✓	✓
R1.2. (meta)data are associated with detailed provenance	✓	✓	✓
R1.3. (meta)data meet domain-relevant community standards	✓	✓	
