# Supplementary material for: Challenges for FAIR-compliant description and comparison of crop phenotype data with standardized controlled vocabularies
Source: Database (Oxford). 2021 May 15;2021:baab028. doi: 10.1093/database/baab028 (PMC8122365; doi:10.1093/database/baab028)
Supplement: baab028_Supp [file baab028_supp.zip › SupplementalTable1.pdf]

Curation	Variable.ID	Variable.name	Variable.synonyms	Context.of.use	Growth.stage	Variable.status	Variable.Xref	Institution	Scientist	Date	Language	Crop	Trait.ID	Trait.name	Trait.class	Trait.description	Trait.synonyms	Main.trait.abbreviation	Alternative.trait.abbreviations	Entity	Attribute	Trait.status	Trait.Xref	Method.ID	Method.name	Method.class	Method.description	Formula
	CO_336:0000249	FlwT_50Count_days	FLOWERDATE	Always	R1-R2		SOY:0001353	IITA	Hesham Agrama	11-Feb-14	EN	Soybean	CO_336:0000000	Flowering time	Phenological traits	[illegible]	Open flower	FlwT		Plant	Flowering time	Recommended	NA	CO_336:0000001	Days to 50% flowering count	Counting	[illegible]	
	CO_336:0000250	BP_Est_1to5	NA	Always	R1-R6		NA	IITA	Hesham Agrama	12-Feb-14	EN	Soybean	CO_336:0000003	Bacterial Pustule	Biotic stress traits	[illegible]	Bacterial Pustule	BP		Leaf	Bacterial Pustule	Standard IITA	SOY:0001426	CO_336:0000004	Bacterial pustle assessment	Estimation	[illegible]	
	CO_336:0000251	RUST_Est_1to5	NA	Always	R1-R6		SOY:0001472	IITA	Hesham Agrama	13-Feb-14	EN	Soybean	CO_336:0000006	Soybean rust	Biotic stress traits	[illegible]	Asian rust	RUST		Plant	Soybean rust	Standard IITA	NA	CO_336:0000007	Rust assessment	Estimation	[illegible]	
	CO_336:0000252	SMV_Est_1to5	NA	Always	R6		NA	IITA	Hesham Agrama	14-Feb-14	EN	Soybean	CO_336:0000009	Soybean Mosaic Virus	Biotic stress traits	[illegible]	Viral infection, SMV	SMV		Leaf	Soybean Mosaic Virus	Standard IITA	SOY:0001501	CO_336:0000010	SMV assessment	Estimation	[illegible]	
	CO_336:0000253	FROGEYE_Est_1to5	NA	Always	R6		NA	IITA	Hesham Agrama	15-Feb-14	EN	Soybean	CO_336:0000012	Frogeye leafspot	Biotic stress traits	[illegible]	Frogeye	FROGEYE		Plant	Frogeye leafspot	Standard IITA	SOY:0001655	CO_336:0000013	Frog eye assessment	Estimation	[illegible]	
	CO_336:0000254	RtNod_PropEst_1to5	NA	Sometimes	R4-R6		NA	IITA	Hesham Agrama	16-Feb-14	EN	Soybean	CO_336:0000015	Nodulation	Agronomical traits	[illegible]	NA	NODULE		Root	Nodulation	Standard USDA	SOY:0001793	CO_336:0000016	Nodulation proportion observation	Estimation	[illegible]	
	CO_336:0000255	ROOTKNOT_Est_1to5	NA	Always	R4-R6		NA	IITA	Hesham Agrama	17-Feb-14	EN	Soybean	CO_336:0000018	Root knot nematode	Biotic stress traits	[illegible]	Root knot nematode	ROOTKNOT		Root	Reaction to Root knot nematode	Standard IITA	SOY:0001374	CO_336:0000019	Root knot assessment	Estimation	[illegible]	
	CO_336:0000256	MatT_Count_day	NA	Often	R8		SOY:0001608	IITA	Hesham Agrama	18-Feb-14	EN	Soybean	CO_336:0000021	Maturity time	Phenological traits	[illegible]	Maturity	MatTime		Plant	Maturity time	Recommended	NA	CO_336:0000022	Days to maturity count	Counting	[illegible]	
	CO_336:0000257	LPodHt_Meas_cm	NA	Sometimes	R3-R6		NA	IITA	Hesham Agrama	19-Feb-14	EN	Soybean	CO_336:0000024	Height of lowest pods	Morphological traits	Height from soil surface to first pod	NA	HLP		Plant	Height of lowest pods	Standard IITA	NA	CO_336:0000025	stalk height measurement	Measurement	Length of stem	
	CO_336:0000258	PlntHt_Meas_cm	NA	Rarely	R8		SOY:0001365	IITA	Hesham Agrama	20-Feb-14	EN	Soybean	CO_336:0000027	Plant height	Agronomical traits	[illegible]	Height	PTHT		Plant	Height	Recommended	NA	CO_336:0000028	Plant height measurement	Measurement	Length of the plant at harvest	
	CO_336:0000259	Lodg_Est_1to5	NA	Always	R8		SOY:0001393	IITA	Hesham Agrama	21-Feb-14	EN	Soybean	CO_336:0000030	Lodging	Agronomical traits	Tendency of plant to lodge	Plants lying on the ground	LODGING		Plant	Lodging	Recommended	NA	CO_336:0000031	Lodging estimation	Estimation	[illegible]	
	CO_336:0000260	PlntHvst_Count_plant	NA	Always	R8		NA	IITA	Hesham Agrama	22-Feb-14	EN	Soybean	CO_336:0000033	Plants Harvested	Agronomical traits	[illegible]	NA	PTHARV		Plant	Harvested number	Standard IITA	NA	CO_336:0000034	[illegible]	Counting	[illegible]	
	CO_336:0000261	SdYld_MeasPerPlot_gPlot	NA	Always	R8		SOY:0001622	IITA	Hesham Agrama	23-Feb-14	EN	Soybean	CO_336:0000036	Seed yield	Agronomical traits	[illegible]	Plot Yield, Seed weight	SdYld		Seed	Yield	Recommended	NA	CO_336:0000037	Weighing of grain per plot	Measurement	[illegible]	
	CO_336:0000262	FodYld_MeasPerPlot_gPlot	NA	Rarely	R8		NA	IITA	Hesham Agrama	24-Feb-14	EN	Soybean	CO_336:0000039	Fodder Yield	Agronomical traits	[illegible]	Haulm	FODDERWT		Fodder	Yield	Standard IITA	NA	CO_336:0000040	Weighing of fodder per plot	Measurement	[illegible]	
	CO_336:0000263	ShatEarly_EarlyEst_1to5	NA	Often	R8		SOY:0001348	IITA	Hesham Agrama	25-Feb-14	EN	Soybean	CO_336:0000042	Early shattering	Agronomical traits	Proportion of open pods at harvest	Pods open	SHATEARLY		Pod	Shattering	Obsolete	NA	CO_336:0000043	Early pod shattering estimation	Estimation	[illegible]	
	CO_336:0000264	PodShat_EarlyEst_1to5	NA	Often	R8		SOY:0001348	IITA	Hesham Agrama	15-Jul-15	EN	Soybean	CO_336:0000248	Pod shattering	Agronomical traits	Proportion of open pods	Pods open	PodShat		Pod	Shattering	Recommended	NA	CO_336:0000043	Early pod shattering estimation	Estimation	[illegible]	
	CO_336:0000265	Storab_Count_Pct	NA	Always	R7		NA	IITA	Hesham Agrama	26-Feb-14	EN	Soybean	CO_336:0000045	Storability	Agronomical traits	[illegible]	Storage	STORABILITY		Seed	Storability	Standard IITA	NA	CO_336:0000046	Germination in storage count	Counting	[illegible]	
	CO_336:0000266	SdOilCt_Comp_pct	NA	Often	R8		NA	IITA	Hesham Agrama	25-Apr-14	EN	Soybean	CO_336:0000048	Seed oil content	Biochemical traits	Oil content of soybean seed	%oil content, oil concentration	OIL		Seed	Oil content	Recommended	SOY:0001668	CO_336:0000049	[illegible]	Computation	[illegible]	
	CO_336:0000267	SdProtCt_Comp_pct	NA	Often	R8		NA	IITA	Hesham Agrama	25-Apr-14	EN	Soybean	CO_336:0000051	Seed protein content	Biochemical traits	Protein content of seed	[illegible]	PROTEIN		Seed	Protein content	Recommended	SOY:0001676	CO_336:0000052	Seed protein content measurement	Computation	[illegible]	
	CO_336:0000268	APHID_Est_Undefined	NA	Always	V1-V7		NA	IITA	Hesham Agrama	25-Apr-14	EN	Soybean	CO_336:0000054	Reaction to Aphis glycines	Biotic stress traits	[illegible]	Soybean aphid	APHID		Plant	Reaction to Aphis glycines	Standard IITA	SOY:0001851	CO_336:0000055	Aphid attack assessment	Estimation	[illegible]	
	CO_336:0000269	PlntBrnch_Est_1to5	NA	Sometimes	R1-R8		SOY:0001318	IITA	Hesham Agrama	25-Apr-14	EN	Soybean	CO_336:0000057	Plant branching	Morphological traits	[illegible]	Branching	BRANCHING		Plant	Branching	Standard IITA	NA	CO_336:0000058	Plant branching observation	Estimation	[illegible]	
	CO_336:0000270	ReprodT_Count_day	NA	Always	R1-R8		SOY:0001824	IITA	Hesham Agrama	25-Apr-14	EN	Soybean	CO_336:0000060	Reproductive period	Phenological traits	[illegible]	Reproductive stage length	REPROD		Plant	Reproductive time	Standard IITA	NA	CO_336:0000061	[illegible]	Counting	[illegible]	
	CO_336:0000271	FlwCol_Est_1to9	NA	Rarely	R1-R3		SOY:0001352	IITA	Hesham Agrama	25-Apr-14	EN	Soybean	CO_336:0000063	Flower color	Morphological traits	Color of the flower petals	NA	FLWRCOLOR		Flower	Color	Recommended	NA	CO_336:0000064	Flower color observation	Estimation	[illegible]	
	CO_336:0000272	PodLng_Meas_mm	NA	Sometimes	R4		NA	IITA	Hesham Agrama	25-Apr-14	EN	Soybean	CO_336:0000066	Pod length	Morphological traits	[illegible]	Fruit length	PODLENGTH		Pod	Length	Standard IITA	SOY:0001717	CO_336:0000067	Measure length of pod	Measurement	[illegible]	
	CO_336:0000273	TwinT_Est_mmdd	NA	Sometimes	V1-R8		SOY:0001618	IITA	Hesham Agrama	25-Apr-14	EN	Soybean	CO_336:0000069	Twining date	Phenological traits	Time when soybean twines	NA	TWINNING		Plant	Twinning time	Standard IITA	NA	CO_336:0000070	Twinning date observation	Estimation	[illegible]	
	CO_336:0000274	Flood_Est_undefined	NA	Often	V1-R8		SOY:0001351	IITA	Hesham Agrama	25-Apr-14	EN	Soybean	CO_336:0000072	Flooding tolerance	Abiotic stress traits	[illegible]	[illegible]	FLOODING		Plant	Flooding tolerance	Standard IITA	NA	CO_336:0000073	[illegible]	Estimation	[illegible]	
	CO_336:0000275	Chlorosis_Est_SPAD	NA	Sometimes	V1-R8		NA	IITA	Hesham Agrama	25-Apr-14	EN	Soybean	CO_336:0000075	Chlorosis	Abiotic stress traits	[illegible]	Yellowing	CHLOROSIS		Plant	Chlorosis	Standard IITA	NA	CO_336:0000076	Chlorophyll content measurement	Estimation	[illegible]	
	CO_336:0000276	Drought_Est_undefined	NA	Often	V1-R8		NA	IITA	Hesham Agrama	25-Apr-14	EN	Soybean	CO_336:0000078	Drought Tolerance	Abiotic stress traits	[illegible]	NA	DROUGHT		Plant	Drought Tolerance	Standard IITA	SOY:0001701	CO_336:0000079	[illegible]	Estimation	[illegible]	
	CO_336:0000277	HerbSens_Est_undefined	NA	Sometimes	V1-R8		NA	IITA	Hesham Agrama	25-Apr-14	EN	Soybean	CO_336:0000081	Herbicide sensitivity	Abiotic stress traits	[illegible]	sensitivity to herbicide toxicity	HERBSENSITIVE		Plant	Herbicide sensitivity	Standard IITA	SOY:0001814	CO_336:0000082	[illegible]	Estimation	[illegible]	
	CO_336:0000278	SdOilProtRatio_Comp_pct	NA	Sometimes	R8		SOY:0001689	IITA	Hesham Agrama	25-Apr-14	EN	Soybean	CO_336:0000084	Seed oil/protein content	Biochemical traits	[illegible]	Oil to protein content	OILPROTEIN		Seed	Oil content per protein content	Standard IITA	NA	CO_336:0000085	Oil content to protein content ratio	Computation	[illegible]	
	CO_336:0000279	SdFillT_Count_day	NA	Often	R4-R6		SOY:0001596	IITA	Hesham Agrama	25-Apr-14	EN	Soybean	CO_336:0000087	Seed filling period	Phenological traits	[illegible]	Seed fill	SEEDFILL		Plant	Seed filling time	Standard IITA	NA	CO_336:0000088	Counting days of seed fill	Counting	[illegible]	
	CO_336:0000280	PodCol_Est_1to4	NA	Sometimes	R4		SOY:0001715	IITA	Hesham Agrama	25-Apr-14	EN	Soybean	CO_336:0000090	Pod color	Morphological traits	Color of the pod.	NA	PODCOLOR		Pod	Color	Recommended	NA	CO_336:0000091	Pod color observation	Estimation	[illegible]	
	CO_336:0000281	HilumCol_Est_1to12	NA	Sometimes	R8		SOY:0001367	IITA	Hesham Agrama	25-Apr-14	EN	Soybean	CO_336:0000093	Hilum color	Morphological traits	[illegible]	Seed hilum color	HILUMCOLOR		Seed	Hilum color	Recommended	NA	CO_336:0000094	Hilum color observation	Estimation	Observe the colour of the hilum	
	CO_336:0000282	SdQual_Est_1to5	NA	Always	R8		SOY:0001418	IITA	Hesham Agrama	25-Apr-14	EN	Soybean	CO_336:0000096	Seed quality	Agronomical traits	[illegible]	Seed appearance	SEEDQUAL		Seed	Quality	Recommended	NA	CO_336:0000097	Seed quality visual assessment	Estimation	Visual assessment of seed quality	
	CO_336:0000283	LowTemp_Est_undefined	NA	Often	V1-R8		NA	IITA	Hesham Agrama	25-Apr-14	EN	Soybean	CO_336:0000099	Cold stress resistance	Abiotic stress traits	[illegible]	low temperature stress	LOWTEMP		Plant	Cold stress resistance	Standard IITA	SOY:0001883	CO_336:0000100	Cold reaction assessment	Estimation	[illegible]	
	CO_336:0000284	HighT_Est_undefined	NA	Often	V1-R8		NA	IITA	Hesham Agrama	25-Apr-14	EN	Soybean	CO_336:0000102	Heat stress resistance	Abiotic stress traits	[illegible]	High temperature stress	HIGHTEMP		Plant	Heat stress resistance	Standard IITA	SOY:0001882	CO_336:0000103	Hot reaction assessment	Estimation	[illegible]	
	CO_336:0000285	SdLinolncCt_Comp_pct	NA	Sometimes	R8		SOY:0001391	IITA	Hesham Agrama	28-Apr-14	EN	Soybean	CO_336:0000105	Seed linoleic acid content	Biochemical traits	[illegible]	[illegible]	LINOLEIC		Seed	Linoleic acid content of oil	Standard IITA	NA	CO_336:0000106	Linoleic acid content measurement	Computation	[illegible]	
	CO_336:0000286	SdLinolCt_Comp_pct	NA	Sometimes	R8		SOY:0001392	IITA	Hesham Agrama	28-Apr-14	EN	Soybean	CO_336:0000108	Seed linolenic acid content	Biochemical traits	[illegible]	[illegible]	LINOLENIC		Seed	Linolenic acid content of oil	Standard IITA	NA	CO_336:0000109	Linolenic acid content measurement	Computation	[illegible]	
[illegible]	[illegible]	[illegible]	[illegible]	[illegible]	[illegible]	[illegible]	[illegible]	[illegible]	[illegible]	[illegible]	[illegible]	[illegible]	[illegible]	[illegible]	[illegible]	[illegible]	[illegible]	[illegible]	[illegible]	[illegible]	[illegible]	[illegible]	[illegible]	[illegible]	[illegible]	[illegible]	[illegible]	[illegible]

CO_334:0000009	Vig_IITAVisScg_1to7	vigor	Breeding trials	default	Recommended	NA	IITA
CO_334:0000010	NoHvstPlnt_Count_Plnt	nohav	Breeding trials	default	Recommended	NA	IITA
CO_334:0000011	NoHvstRt_Count_Rt	rtno	Breeding trials	default	Recommended	NA	IITA
CO_334:0000012	RtWt_Wgh_kg	rtwt	Breeding trials	default	Recommended	NA	IITA
CO_334:0000013	RtYld_Fresh_tha	fyld	Breeding trials	null	Recommended	NA	IITA
CO_334:0000014	RtYld_Dry_tha	dyld	Breeding trials	null	Recommended	NA	IITA
CO_334:0000016	ShWt_Meas_kg	shtwt	Breeding trials	default	Recommended	NA	IITA
CO_334:0000017	RtYld_FreshAerial_tha	tyld	Breeding trials	null	Recommended	NA	IITA
CO_334:0000018	PlntH_Meas_cm	plthit	Characterization trials	default	Recommended	NA	IITA
CO_334:0000019	RtLng_VisScg_1to7	rtsz	Breeding trials	default	Recommended	NA	IITA
CO_334:0000020	RtShp_VisClsn_1to6	rtshp	Breeding trials	default	Recommended	NA	IITA
CO_334:0000021	PlpCol_VisClsn_1to3	plpcol	Breeding trials	default	Recommended	NA	IITA
CO_334:0000022	NkLng_VisClsn_0to7	nklgt	Breeding trials	default	Recommended	NA	IITA
CO_334:0000023	PtlCol_VisClsn_1to9	petcol	Characterization trials	null	Recommended	NA	IITA
CO_334:0000024	PtlLng_VisScg_0to7	petlgt	Characterization trials	null	Recommended	NA	IITA
CO_334:0000025	StPub_VisScg_1to7	stmpub	Characterization trials	null	Recommended	NA	IITA
CO_334:0000026	LfPub_VisClsn_0to7	apubes	Characterization trials	null	Recommended	NA	IITA
CO_334:0000034	CMBSev_Est_1to5	cmbs	Breeding trials	default	Recommended	NA	IITA
CO_334:0000041	CMBInc_Est_1to5	cmbi	Breeding trials	default	Recommended	NA	IITA
CO_334:0000042	SWfSev_VisScg_1to5	Swfs	Breeding trials	null	Recommended	NA	IITA
CO_334:0000043	SWfInc_Comp_r	Swfi	Breeding trials	null	Recommended	NA	IITA
CO_334:0000044	RSMSev_VisScg_1to5	rsms	Breeding trials	null	Recommended	NA	IITA
CO_334:0000045	RSMInc_Comp_r	rsmi	Breeding trials	null	Recommended	NA	IITA
CO_334:0000046	PtlAng_VisScg_0to7	petang	Characterization trials	null	Recommended	NA	IITA
CO_334:0000047	AbsCt_Meas_ugg	abscon	Breeding trials	null	Recommended	NA	IITA
CO_334:0000048	LfRet_VisSc_1to7	lfret	Breeding trials	default	Recommended	NA	IITA
CO_334:0000049	LfLife_Count_days	lflife	Breeding trials	null	Recommended	NA	IITA
CO_334:0000050	RtPed_VisClsn_0to7	rtpedu	Characterization trials	default	Recommended	NA	IITA
CO_334:0000051	RtConsPrsnc_VisClsn_0or1	rtcons	Characterization trials	default	Recommended	NA	IITA
CO_334:0000052	RtPos_VisClsn_1to3	rtpost	Characterization trials	null	Recommended	NA	IITA
CO_334:0000053	RtSurfCol_VisClsn_1to3	rtscol	Breeding trials	default	Recommended	NA	IITA
CO_334:0000054	RtSurfTex_VisClsn_1to7	rttext	Characterization trials	default	Recommended	NA	IITA
CO_334:0000055	RtDia_VisScg_1to7	rtdiam	Breeding trials	null	Recommended	NA	IITA
CO_334:0000056	CarunCol_VisClsn_1to3	carcol	Characterization trials	null	Recommended	NA	IITA
CO_334:0000057	StgmCol_VisClsn_1to5	stgmco	Characterization trials	null	Recommended	NA	IITA
CO_334:0000058	OvrCol_VisClsn_1to5	ovryco	Characterization trials	null	Recommended	NA	IITA
CO_334:0000059	DiscCol_VisClsn_1to5	discol	Characterization trials	null	Recommended	NA	IITA
CO_334:0000060	SepCol_VisClsn_1to5	splcol	Characterization trials	null	Recommended	NA	IITA
CO_334:0000061	AnthCol_VisClsn_1to5	anther	Characterization trials	null	Recommended	NA	IITA
CO_334:0000062	StmCol_VisClsn_1to4	stmcol	Characterization trials	default	Recommended	NA	IITA
CO_334:0000063	SdCol_VisClsn_1to5	seedco	Characterization trials	null	Recommended	NA	IITA
CO_334:0000064	RtPerldCol_VisClsn_1to4	outcol	Breeding trials	default	Recommended	NA	IITA
CO_334:0000065	AscACt_Meas_pct	null	Breeding trials	null	Recommended	NA	IITA
CO_334:0000066	MinCt_Burng_pct	ash	Breeding trials	null	Recommended	NA	IITA
CO_334:0000067	FibCt_Meas_pct	fibre	Breeding trials	null	Recommended	NA	IITA
CO_334:0000068	FeCt_Meas_ugg	fe	Breeding trials	null	Recommended	NA	IITA
CO_334:0000069	RtMoistCt_Comp_pct	mc	Breeding trials	default	Recommended	NA	IITA
CO_334:0000070	ProtCt_Meas_pct	protei	Breeding trials	null	Recommended	NA	IITA
CO_334:0000071	StrchCt_Est_pct	starch	Breeding trials	default	Recommended	NA	IITA
CO_334:0000072	SugPCt_Meas_pct	sugar	Breeding trials	null	Recommended	NA	IITA
CO_334:0000073	CarotCt_spectro_ugg	tc	Breeding trials	null	Recommended	NA	IITA
CO_334:0000074	Pd_SensScg_1to4	pd	Breeding trials	default	Recommended	NA	IITA

[illegible]

CO_320:0000844	RaggedStuntSev_Est_0to9	RAGGED_STUNT	null	null	null	IRIS:186, 186SES:36	IRRI	Jeffrey Detras	null	EN	Rice	CO_320:0000183	ragged stunt disease	Biotic stress	[illegible]	[illegible]	RaggedStuntSev	null	Ragged stunt	Severity	null	TO:0000386, IRIS:1112	CO_320:0000795	ragged stunt disease estimation	Estimation	[illegible]	null
CO_320:0000845	RatDam_Est_1to9	RAT_DAMAGE	null	null	Standard CIAT, FLAR	IRIS:213, SES:50	IRRI	Jeffrey Detras	null	EN	Rice	CO_320:0000214	rat damage	Biotic stress	[illegible]	null	RatDam	null	Rat	Damage	null	TO:0000117, IRIS:1170	CO_320:0000796	rat damage incidence estimation	Estimation	[illegible]	null
CO_320:0000846	RhyzForm_Est_1to6	RHIZOME_FORMATION	null	null	null	IRIS:534, RD:7.3.36	IRRI	Jeffrey Detras	null	EN	Rice	CO_320:0000124	rhizome and stolon formation	Morphological	[illegible]	null	RhyzForm	RHIFOR	Rhizome/stolon	Formation	null	TO:0002767	CO_320:0000284	rhizome formation observation	Estimation	[illegible]	null
CO_320:0000847	RBugDam_Est_0to9	RICE_BUG_DAMAGE	null	null	Standard CIAT, FLAR	SES:68	IRRI	Jeffrey Detras	null	EN	Rice	CO_320:0000203	rice bug damage	Biotic stress	[illegible]	rice bug	RBugDam	null	Rice bug	Damage	null	TO:0000220, IRIS:1159	CO_320:0000388	rice bug damage assessment	Estimation	[illegible]	null
CO_320:0000848	RDelphDam_EstFld_0to9	RICE_DELPHACID	null	null	null	SES:62	IRRI	Jeffrey Detras	null	EN	Rice	CO_320:0000216	rice delphacid	Biotic stress	[illegible]	rice delphacid	RDelphDam	null	Rice delphacid	Damage	null	TO:0000246	CO_320:0000797	[illegible]	Estimation	[illegible]	null
CO_320:0000849	RDelphDam_EstGH_0to9	RICE_DELPHACID	null	null	Standard CIAT, FLAR	IRIS:124, SES:62	IRRI	Jeffrey Detras	null	EN	Rice	CO_320:0000216	rice delphacid	Biotic stress	[illegible]	rice delphacid	RDelphDam	null	Rice delphacid	Damage	null	TO:0000246	CO_320:0000798	[illegible]	Estimation	[illegible]	null
CO_320:0000850	RTungroSev_Est_0to9	RTD1	null	null	null	IRIS:1, SES:36	IRRI	Jeffrey Detras	null	EN	Rice	CO_320:0000184	rice tungro disease	Biotic stress	[illegible]	[illegible]	RTungroSev	null	Rice tungro	Severity	null	TO:0000413, IRIS:1113	CO_320:0000350	rice tungro disease estimation	Estimation	[illegible]	null
CO_320:0000851	RTungroSev_GHComp_Pct	RTD1	null	null	null	IRIS:2	IRRI	Jeffrey Detras	null	EN	Rice	CO_320:0000184	rice tungro disease	Biotic stress	[illegible]	[illegible]	RTungroSev	null	Rice tungro	Severity	null	TO:0000413, IRIS:1113	CO_320:0000572	[illegible]	Computation	[illegible]	null
CO_320:0000852	RWhorlMaggDam_Est_0to9	RICE_WHORL_MAGGOT	null	null	null	IRIS:64, SES:67	IRRI	Jeffrey Detras	null	EN	Rice	CO_320:0000206	rice whorl maggot damage	Biotic stress	[illegible]	rice whorl maggot	RWhorlMaggDam	null	Rice whorl maggot	Damage	null	TO:0000332, IRIS:1162	CO_320:0000386	[illegible]	Estimation	[illegible]	null
CO_320:0000853	SaltInj_Est_1to9	SAL	null	null	null	IRIS:5, SES:71	IRRI	Jeffrey Detras	null	EN	Rice	CO_320:0000066	salt injury	Abiotic stress	[illegible]	salt tolerance, salinity injury, salinity	SaltInj	null	Salt	Injury	null	[illegible]	CO_320:0000799	salt injury assessment	Estimation	[illegible]	null
CO_320:0000854	Sct_SmellCook_0to2	SCT	null	null	null	IRIS:146, RD:8.1.2	IRRI	Jeffrey Detras	null	EN	Rice	CO_320:0000103	scent	Quality	Describes the aroma of rice grains	Aroma, caryopsis scent	Sct	null	Caryopsis	Scent	null	TO:0000087, IRIS:1252	CO_320:0000800	sensed aroma cooked	Estimation	[illegible]	null
CO_320:0000855	Sct_SmellUncook_0to2	SCT	null	null	null	IRIS:860, SES:129, UPOV:65	IRRI	Jeffrey Detras	null	EN	Rice	CO_320:0000103	scent	Quality	Describes the aroma of rice grains	Aroma, caryopsis scent	Sct	null	Caryopsis	Scent	null	TO:0000087, IRIS:1252	CO_320:0000801	sensed aroma uncooked	Estimation	[illegible]	null
CO_320:0000856	Pan2ndBr_Est_0to3	[illegible]	null	null	null	[illegible]	IRRI	Jeffrey Detras	null	EN	Rice	CO_320:0000034	secondary panicle branching	Morphological	[illegible]	null	Pan2ndBr	SECOND_BR	[illegible]	Pattern	null	TO:0000142, IRIS:1083	CO_320:0000420	[illegible]	Estimation	[illegible]	null
CO_320:0000857	SdGerm_Est_Pct	SG	null	null	Standard CIAT, FLAR	null	IRRI	Jeffrey Detras	null	EN	Rice	CO_320:0000086	seed germination	Agronomical	[illegible]	null	SdGerm	null	Seed	Germination	null	IRIS:1016	CO_320:0000802	seed germination 1	Estimation	[illegible]	null
CO_320:0000858	SdDate_Meas_Date	DATE_SEED	null	null	Standard CIAT, FLAR	RD:7.2.1	IRRI	Jeffrey Detras	null	EN	Rice	CO_320:0000514	seeding date	Agronomical	[illegible]	null	SdDate	null	Plant	seeding date	null	null	CO_320:0000803	Seeding date observation	Measurement	Record the date of effective seeding	null
CO_320:0000859	SeedlHt_Av_cm	SEEDLING_HEIGHT	null	null	null	RD:7.3.2, SES:91	IRRI	Jeffrey Detras	null	EN	Rice	CO_320:0000218	seedling height	Morphological	[illegible]	null	SeedlHt	SDHT	Seedling	Height	null	TO:0000019, IRIS:1006	CO_320:0000227	average seedling height	Computation	[illegible]	null
CO_320:0000860	SeedlHt_Av_1to7	SEEDLING_HEIGHT	null	null	null	RD:7.3.2	IRRI	Jeffrey Detras	null	EN	Rice	CO_320:0000218	seedling height	Morphological	[illegible]	null	SeedlHt	SDHT	Seedling	Height	null	TO:0000019, IRIS:1006	CO_320:0000227	average seedling height	Computation	[illegible]	null
CO_320:0000861	ShBlInc_Est_0to9	SHB	null	null	Standard CIAT, FLAR	IRIS:45, SES:37	IRRI	Jeffrey Detras	null	EN	Rice	CO_320:0000189	sheath blight	Biotic stress	[illegible]	null	ShBlInc	null	sheath blight	Relative height	null	TO:0000255, IRIS:1117	CO_320:0000354	[illegible]	Estimation	[illegible]	null
CO_320:0000862	ShRotInc_Est_0to9	SHEATH_ROT	null	null	Standard CIAT, FLAR	IRIS:54, SES:38	IRRI	Jeffrey Detras	null	EN	Rice	CO_320:0000185	sheath rot	Biotic stress	[illegible]	sheath rot	ShRotInc	null	sheath rot	Incidence	null	TO:0000086, IRIS:1114	CO_320:0000360	sheath rot incidence estimation	Estimation	[illegible]	null
CO_320:0000863	SpkFer_Est_1to5	SPIKELET_FERTILITY, SPKF, SpFert	null	null	null	IRIS:694, RD:7.5.14	IRRI	Jeffrey Detras	null	EN	Rice	CO_320:0000090	spikelet fertility	Agronomical	[illegible]	null	SpkFer	SPKF, SpFert	Spikelet	Fertility	null	TO:0000180, IRIS:1021	CO_320:0000302	spikelet fertility score	Estimation	[illegible]	null
CO_320:0000864	SpkFer_EstSES_1to9	SPIKELET_FERTILITY, SPKF, SpFert	null	null	Obsolete	IRIS:694, SES:9	IRRI	Jeffrey Detras	null	EN	Rice	CO_320:0000090	spikelet fertility	Agronomical	[illegible]	null	SpkFer	SPKF, SpFert	Spikelet	Fertility	null	TO:0000180, IRIS:1021	CO_320:0000481	spikelet fertility estimation SES	Estimation	[illegible]	null
CO_320:0000865	SpkFer_Est_1to9	SPIKELET_FERTILITY, SPKF, SpFert	null	null	null	IRIS:694, SES:9	IRRI	Jeffrey Detras	null	EN	Rice	CO_320:0000090	spikelet fertility	Agronomical	[illegible]	null	SpkFer	SPKF, SpFert	Spikelet	Fertility	null	TO:0000180, IRIS:1021	CO_320:0000302	spikelet fertility score	Estimation	[illegible]	null
CO_320:0000866	SpkSter_Comp_1to9	SPIKELET_STERILITY	null	null	Standard CIAT, FLAR	IRIS:197, SES:21B	IRRI	Jeffrey Detras	null	EN	Rice	CO_320:0000159	Spikelet sterility	Agronomical	[illegible]	null	SpkSter	SPKF, SpFert	Sterile spikelet	Proportion	null	TO:0000436	CO_320:0000328	spikelet sterility calculation	Computation	[illegible]	null
CO_320:0000867	StemBorer_YellowEst_0to9	STEM_BORER_DAMAGE	null	null	null	IRIS:42, SES:63	IRRI	Jeffrey Detras	null	EN	Rice	CO_320:0000208	stem borer damage	Biotic stress	[illegible]	stem borer	StemBorer	null	Stem borer	Damage	null	TO:0000454, IRIS:164	CO_320:0000378	[illegible]	Estimation	[illegible]	null
CO_320:0000868	StemBorer_DeadheartEst_0to9	STEM_BORER_DAMAGE	null	null	null	IRIS:62, SES:63	IRRI	Jeffrey Detras	null	EN	Rice	CO_320:0000208	stem borer damage	Biotic stress	[illegible]	stem borer	StemBorer	null	Stem borer	Damage	null	TO:0000454, IRIS:164	CO_320:0000573	[illegible]	Estimation	[illegible]	null
CO_320:0000869	StemBorer_WhiteHeadEst_0to9	STEM_BORER_DAMAGE	null	null	null	SES:63	IRRI	Jeffrey Detras	null	EN	Rice	CO_320:0000208	stem borer damage	Biotic stress	[illegible]	stem borer	StemBorer	null	Stem borer	Damage	null	TO:0000454, IRIS:164	CO_320:0000804	[illegible]	Estimation	[illegible]	null
CO_320:0000870	StemBorer_DeepWatEst_0to9	STEM_BORER_DAMAGE	null	null	Standard CIAT, FLAR	SES:63	IRRI	Jeffrey Detras	null	EN	Rice	CO_320:0000208	stem borer damage	Biotic stress	[illegible]	stem borer	StemBorer	null	Stem borer	Damage	null	TO:0000454, IRIS:164	CO_320:0000805	[illegible]	Estimation	[illegible]	null
CO_320:0000871	StmElong_Est_1to9	ELONGATION	null	null	null	IRIS:34, SES:85	IRRI	Jeffrey Detras	null	EN	Rice	CO_320:0000061	stem elongation	Abiotic stress	[illegible]	[illegible]	StmElong	null	Stem	Elongation	null	TO:0006036	CO_320:0000406	stem elongation estimation	Estimation	[illegible]	null
CO_320:0000872	StmRotInc_Est_0to9	STEM_ROT	null	null	Standard CIAT, FLAR	SES:42	IRRI	Jeffrey Detras	null	EN	Rice	CO_320:0000186	stem rot	Biotic stress	[illegible]	stem rot	StmRotInc	null	stem rot	Incidence	null	TO:0000323, IRIS:1115	CO_320:0000366	stem rot incidence estimation	Estimation	[illegible]	null
CO_320:0000873	StrLmCol_Est_1to4	STERILE_LEMMA_COLOR	null	null	null	[illegible]	IRRI	Jeffrey Detras	null	EN	Rice	CO_320:0000041	sterile lemma color	Morphological	Colour of the sterile lemmas	glume color	StrLmCol	SLCO, SLmc	Sterile lemma	Colour	null	TO:0000400, IRIS:1091	CO_320:0000300	sterile lemma color examination cult	Estimation	[illegible]	null
CO_320:0000874	StrLmCol_Est_1to9	STERILE_LEMMA_COLOR	null	null	null	RD:7.5.13	IRRI	Jeffrey Detras	null	EN	Rice	CO_320:0000041	sterile lemma color	Morphological	Colour of the sterile lemmas	glume color	StrLmCol	SLCO, SLmc	Sterile lemma	Colour	null	TO:0000400, IRIS:1091	CO_320:0000571	sterile lemma color examination wild	Estimation	[illegible]	null
CO_320:0000875	StrLmLng_AvShort_1to9	STERILE_LEMMA_LENGTH	null	null	null	IRIS:325, RD:7.5.10, UPOV:51	IRRI	Jeffrey Detras	null	EN	Rice	CO_320:0000042	sterile lemma length	Morphological	Length of sterile lemmas	glume length	StrLmLng	SLLT	Sterile lemma	Length	null	TO:0000240, IRIS:1091	CO_320:0000222	average sterile lemma length - shorter	Computation	[illegible]	null
CO_320:0000876	StrLmLng_AvLong_1to9	STERILE_LEMMA_LENGTH	null	null	null	IRIS:325, RD:7.5.11, UPOV:51	IRRI	Jeffrey Detras	null	EN	Rice	CO_320:0000042	sterile lemma length	Morphological	Length of sterile lemmas	glume length	StrLmLng	SLLT	Sterile lemma	Length	null	TO:0000240, IRIS:1091	CO_320:0000806	average sterile lemma length - longer	Computation	[illegible]	null
CO_320:0000877	StrLmLng_AvShort_mm	STERILE_LEMMA_LENGTH	null	null	null	IRIS:325, RD:7.5.10	IRRI	Jeffrey Detras	null	EN	Rice	CO_320:0000042	sterile lemma length	Morphological	Length of sterile lemmas	glume length	StrLmLng	SLLT	Sterile lemma	Length	null	TO:0000240, IRIS:1091	CO_320:0000222	average sterile lemma length - shorter	Computation	[illegible]	null
CO_320:0000878	LemaShp_Est_0to3	SLSH	null	null	null	IRIS:529, RD:7.5.12	IRRI	Jeffrey Detras	null	EN	Rice	CO_320:0000121	sterile lemma shape	Morphological	Shape of the sterile lemmas	null	LemaShp	null	Sterile lemma	Shape	null	TO:0002726	CO_320:0000298	Sterile lemma shape	Estimation	Examine the shape of sterile lemmas	null
CO_320:0000879	StgmCol_Est_1to5	STIGMA_COLOR	null	null	Standard CIAT, FLAR	[illegible]	IRRI	Jeffrey Detras	null	EN	Rice	CO_320:0000038	stigma color	Morphological	Color of stigma	null	StgmCol	STCO	Stigma	Colour	null	TO:0000185, IRIS:1087	CO_320:0000225	stigma color examination	Estimation	[illegible]	null
CO_320:0000880	StgExers_Comp_1to9	STIGMA_EXSERTION	null	null	null	IRIS:165, SES:25	IRRI	Jeffrey Detras	null	EN	Rice	CO_320:0000093	stigma exsertion	Morphological	[illegible]	null	StgExers	null	Stigma	Exsertion	null	TO:0000044, IRIS:1024	CO_320:0000334	stigma exsertion calculation	Computation	[illegible]	null
CO_320:0000881	SubTol_GHEst_1to9	SUB	null	null	null	SES:86	IRRI	Jeffrey Detras	null	EN	Rice	CO_320:0000067	submergence tolerance	Abiotic stress	[illegible]	null	SubTol	null	Submergence	Tolerance	null	[illegible]	CO_320:0000807	[illegible]	Estimation	[illegible]	null
CO_320:0000882	SubTol_FldEst_1to9	SUB	null	null	Standard CIAT, FLAR	IRIS:3, SES:86	IRRI	Jeffrey Detras	null	EN	Rice	CO_320:0000067	submergence tolerance	Abiotic stress	[illegible]	null	SubTol	null	Submergence	Tolerance	null	[illegible]	CO_320:0000408	submergence survival assessment	Estimation	[illegible]	null
CO_320:0000883	ThripDam_Est_0to9	THRIPS	null	null	null	IRIS:74, SES:69	IRRI	Jeffrey Detras	null	EN	Rice	CO_320:0000210	thrip damage	Biotic stress	[illegible]	thrips	ThripDam	null	Thrip	Damage	null	TO:0000154	CO_320:0000390	thrips damage assessment	Estimation	[illegible]	null
CO_320:0000884	TillNo_Comp_1to9	TI	null	null	Standard CIAT, FLAR	SES:2	IRRI	Julie Pasuquin	null	EN	Rice	CO_320:0000079	Tiller number per plant	Agronomical	[illegible]	Tillering	TillNo	TilPlt	Tiller	Number per plant	null	TO:0000346	CO_320:0000314	Average - Tiller number per plant	Computation	[illegible]	null
CO_320:0000885	UDInc_Est_0to9	UDBATTA_DISEASE	null	null	null	IRIS:181, SES:41	IRRI	Jeffrey Detras	null	EN	Rice	CO_320:0000194	udbatta disease	Biotic stress	[illegible]	udbatta disease	UDInc	null	udbatta disease	Incidence	null	TO:0000045, IRIS:1123	CO_320:0000364	udbatta disease incidence estimation	Estimation	[illegible]	null
CO_320:0000886	UfraDam_Est_0to9	UFRA	null	null	null	IRIS:215, SES:43	IRRI	Jeffrey Detras	null	EN	Rice	CO_320:0000187	ufra	Biotic stress	[illegible]	ufra	UfraDam	null	Ufra	Incidence	null	TO:0000121, IRIS:1116	CO_320:0000368	ufra incidence estimation	Estimation	[illegible]	null
CO_320:0000887	PlntVg_SdEst_1to9	VG	null	null	Standard CIAT, FLAR	IRIS:698, SES:1	IRRI	Jeffrey Detras	null	EN	Rice	CO_320:0000077	vigor	Agronomical	[illegible]	null	PlntVg	null	Plant	Vigor	null	TO:0000296, IRIS:1007	CO_320:0000336	seedling vigor	Estimation	Assess the vigor of seedlings	null
CO_320:0000888	PlntVg_VegEst_1to9	VG	null	null	Standard CIAT, FLAR	SES:1	IRRI	Jeffrey Detras	null	EN	Rice	CO_320:0000077	vigor	Agronomical	[illegible]	null	PlntVg	null	Plant	Vigor	null	TO:0000296, IRIS:1007	CO_320:0000312	vegetative vigor	Estimation	[illegible]	null
CO_320:0000889	WBPH_EstGH_0to9	WBPH	null	null	null	IRIS:41, SES:62	IRRI	Jeffrey Detras	null	EN	Rice	CO_320:0000211	whitebacked planthopper damage	Biotic stress	[illegible]	whitebacked planthopper	WBPH	null	Whitebacked planthopper	Damage	null	TO:0000205	CO_320:0000374	[illegible]	Estimation	[illegible]	null
CO_320:0000890	WBPH_EstFld_0to9	WBPH	null	null	null	IRIS:198, SES:62	IRRI	Jeffrey Detras	null	EN	Rice	CO_320:0000211	whitebacked planthopper damage	Biotic stress	[illegible]	whitebacked planthopper	WBPH	null	Whitebacked planthopper	Damage	null	TO:0000205	CO_320:0000808	[illegible]	Estimation	[illegible]	null
CO_320:0000891	YellowDwarfSev_Est_0to9	YELLOW_DWARF	null	null	null	SES:36	IRRI	Jeffrey Detras	null	EN	Rice	CO_320:0000515	yellow dwarf	Biotic stress	[illegible]	null	YellowDwarfSev	null	Yellow dwarf	Severity	null	TO:0000292	CO_320:0000809	yellow dwarf disease assessment	Estimation	[illegible]	null
CO_320:0000892	YellowMottleSev_Est_0to9	YELLOW_MOTTLE	null	null	null	IRIS:174, SES:36	IRRI	Jeffrey Detras	null	EN	Rice	CO_320:0000192	yellow mottle disease	Biotic stress	[illegible]	[illegible]	YellowMottleSev	null	Yellow mottle	Severity	null	TO:0000088, IRIS:1121	CO_320:0000356	yellow mottle disease assessment	Estimation	[illegible]	null
CO_320:0000893	ZnDefInj_Est_1to9	ZDIF	null	null	pending curation	IRIS:11, SES:74	IRRI	Jeffrey Detras	null	EN	Rice	CO_320:0000069	zinc deficiency injury	Abiotic stress	[illegible]	zinc deficiency	ZnDefInj	null	Zinc deficiency	Injury	pending curation	TO:0000351, IRIS:1217	CO_320:0000396	zinc deficiency injury assessment	Estimation	[illegible]	null
CO_320:0000988	HdR_Comp_Perc	HDR	null	null	pending curation	IRIS:108	IRRI	Jeffrey Detras	null	EN	Rice	CO_320:0000108	head rice	Quality	[illegible]	head rice percent	HdR	null	Head rice	Proportion	pending curation	TO:0000222, IRIS:1257	CO_320:0000471	head rice proportion	Computation	[illegible]	null
CO_320:0000989	HeatSens_Est_0to9	HTOL	null	null	pending curation	IRIS:78, SES:76	IRRI	Jeffrey Detras	null	EN	Rice	CO_320:0000062	heat injury	Abiotic stress	[illegible]	heat tolerance	HeatSens	null	Heat	Sensitivity	pending curation	TO:0000259, IRIS:1210	CO_320:0000400	flowering heat injury	Estimation	[illegible]	null

[illegible]

CO_321:0001265	PrecoNote_E_text	PRECO_text	Nursery/Trial evaluation	[illegible]	null	BMS_29144	CIMMYT	Rosemary Shrestha; Julian Pietragalla	25-oct.-2015	EN	Wheat	CO_321:0000687	Leaf rust notes	Biotic stress traits	[illegible]	null	PrecoNote	BrwRustInc, LRInc	Leaf	Leaf rust notes	NA	NA	CO_321:0000708	Note Estimation	Estimation	Notes taken in the field.	null
CO_321:0001266	PrecoResp_E_0to4	PRECO_0_4__RESP	Nursery/Trial evaluation	[illegible]	null	BMS_22764	CIMMYT	Rosemary Shrestha; Julian Pietragalla	25-oct.-2015	EN	Wheat	CO_321:0000178	Leaf rust plant response	Biotic stress traits	[illegible]	null	PrecoResp	BwRustResp, LRResp	Leaf	Leaf rust plant response	NA	NA	CO_321:0000706	PrecoResp Estimation	Estimation	[illegible]	null
CO_321:0001267	PrecoResp_E_1to4	PRECO_1_4__RESP	Nursery/Trial evaluation	[illegible]	null	BMS_22763	CIMMYT	Rosemary Shrestha; Julian Pietragalla	25-oct.-2015	EN	Wheat	CO_321:0000178	Leaf rust plant response	Biotic stress traits	[illegible]	null	PrecoResp	BwRustResp, LRResp	Leaf	Leaf rust plant response	NA	NA	CO_321:0000706	PrecoResp Estimation	Estimation	[illegible]	null
CO_321:0001268	PrecoResp_E_1to8	PRECO_1_8__ADLTRESP	Nursery/Trial evaluation	[illegible]	null	BMS_29143	CIMMYT	Rosemary Shrestha; Julian Pietragalla	23-sept.-2014	EN	Wheat	CO_321:0000178	Leaf rust plant response	Biotic stress traits	[illegible]	null	PrecoResp	BwRustResp, LRResp	Leaf	Leaf rust plant response	NA	NA	CO_321:0000360	[illegible]	Estimation	[illegible]	null
CO_321:0001269	PrecoSdlgResp_E_0to8	PRECO_0_8__SEEDRESP	Nursery/Trial evaluation	DS11-DS15	null	BMS_29142	CIMMYT	Rosemary Shrestha; Julian Pietragalla	23-sept.-2014	EN	Wheat	CO_321:0000901	Leaf rust seedling response	Biotic stress traits	[illegible]	null	PrecoSdlgResp	BwRustSdlgResp, LRSdlgResp	Seedling	Leaf rust seedling response	NA	NA	CO_321:0000604	[illegible]	Estimation	[illegible]	null
CO_321:0001270	PrecoSev_E_pct	PRECO_pct__SEV	Nursery/Trial evaluation	[illegible]	null	BMS_22760	CIMMYT	Rosemary Shrestha; Julian Pietragalla	23-sept.-2014	EN	Wheat	CO_321:0000902	Leaf rust severity	Biotic stress traits	[illegible]	null	PrecoSev	BwRustSev, LRSev	Leaf	Leaf rust severity	NA	NA	CO_321:0000693	Rust severity Estimation	Estimation	[illegible]	null
CO_321:0001271	PrecoSev_E_0to12	PRECO_0_12__SEV	Nursery/Trial evaluation	[illegible]	null	BMS_22759	CIMMYT	Rosemary Shrestha; Julian Pietragalla	25-oct.-2015	EN	Wheat	CO_321:0000902	Leaf rust severity	Biotic stress traits	[illegible]	null	PrecoSev	BwRustSev, LRSev	Leaf	Leaf rust severity	NA	NA	CO_321:0000324	Modified cobb scale for severity	Estimation	[illegible]	null
CO_321:0001272	StomaCond_M_mmolm2s	STMCOND_mmol_m2_s	Research-intensive characterization	DS12-DS81	null	BMS_29177	CIMMYT	Rosemary Shrestha; Julian Pietragalla	23-sept.-2014	EN	Wheat	CO_321:0000018	Leaf stomatal conductance	Physiological traits	[illegible]	null	StomaCond	Cond, GS, STMCOND	Leaf	Stomatal conductance	NA	NA	CO_321:0000240	StomaCond Measurement	Measurement	[illegible]	null
CO_321:0001273	StomaFrq_Ct_stomamm2	STMFRQ_num, STMFRQ	Research-intensive characterization	DS12-DS81	null	BMS_29179	CIMMYT	Rosemary Shrestha; Julian Pietragalla	23-sept.-2014	EN	Wheat	CO_321:0000143	Leaf stomatal frequency	Morphological traits	[illegible]	null	StomaFrq	null	Leaf	Stomatal frequency	NA	NA	CO_321:0000349	StomaFrq Counting	Counting	[illegible]	null
CO_321:0001274	StomaRes_M_sm	STMRES_s_m	Research-intensive characterization	DS12-DS81	null	BMS_29181	CIMMYT	Rosemary Shrestha; Julian Pietragalla	23-sept.-2014	EN	Wheat	CO_321:0000063	Leaf stomatal resistance	Physiological traits	[illegible]	null	StomaRes	Res, STMRES	Leaf	Stomatal resistance	NA	NA	CO_321:0000285	StomaRes Measurement	Measurement	[illegible]	null
CO_321:0001275	StomaSiz_M_um	STMSIZ_um	Research-intensive characterization	DS12-DS81	null	BMS_29182	CIMMYT	Rosemary Shrestha; Julian Pietragalla	23-sept.-2014	EN	Wheat	CO_321:0000142	Leaf stomatal size	Morphological traits	Stomata size.	null	StomaSiz	STMSIZ	Leaf	Stomatal size	NA	NA	CO_321:0000348	StomaSiz Measurement	Measurement	Standard method for stomatal size.	null
CO_321:0001276	LeafThk_M_um	LEAFTHK_mm	Research-intensive characterization	null	null	BMS_29113	CIMMYT	Rosemary Shrestha; Julian Pietragalla	23-sept.-2014	EN	Wheat	CO_321:0000047	Leaf thickness	Morphological traits	Measurement of leaf thickness.	null	LeafThk	null	Leaf	Thickness	NA	NA	CO_321:0000267	LeafThk Measurement	Measurement	Standard method for leaf thickness.	null
CO_321:0001277	LeafTNecro_E_pct	LEAFTNECR_pct	Nursery/Trial evaluation	[illegible]	null	BMS_29114	CIMMYT	Rosemary Shrestha; Julian Pietragalla	23-sept.-2014	EN	Wheat	CO_321:0000175	Leaf tip necrosis incidence	Morphological traits	[illegible]	null	LeafTNecro	LEAFTNECR	Leaf	Tip necrosis	NA	NA	CO_321:0000361	LeafTNecro Estimation	Estimation	[illegible]	null
CO_321:0001278	LeafWid_M_mm	LEAFWID_mm	Research-intensive characterization	null	null	BMS_29115	CIMMYT	Rosemary Shrestha; Julian Pietragalla	23-sept.-2014	EN	Wheat	CO_321:0000049	Leaf width	Morphological traits	Width of leaf.	null	LeafWid	null	Leaf	Width	NA	NA	CO_321:0000269	LeafWid Measurement	Measurement	Standard method for leaf width.	null
CO_321:0001279	Lodg_E_0to9pcttext	LODG_0_9text	Trial evaluation	After the event	Obsolete/legacy	BMS_22904	CIMMYT	Rosemary Shrestha; Julian Pietragalla	25-oct.-2015	EN	Wheat	CO_321:0000167	Lodging incidence	Agronomical traits	Indicates incidence of lodged plants.	null	Lodg	LOD	Plant	Lodging incidence	NA	NA	CO_321:0000355	Lodg Estimation	Estimation	[illegible]	null
CO_321:0001280	Lodg_E_pct	LODG_pct	Trial evaluation	After the event	null	BMS_18060	CIMMYT	Rosemary Shrestha; Julian Pietragalla	23-sept.-2014	EN	Wheat	CO_321:0000167	Lodging incidence	Agronomical traits	Indicates incidence of lodged plants.	null	Lodg	LOD	Plant	Lodging incidence	NA	NA	CO_321:0000355	Lodg Estimation	Estimation	[illegible]	null
CO_321:0001281	Lodg_E_0to5pct	LODG_0_5	Trial evaluation	After the event	null	BMS_22906	CIMMYT	Rosemary Shrestha; Julian Pietragalla	25-oct.-2015	EN	Wheat	CO_321:0000167	Lodging incidence	Agronomical traits	Indicates incidence of lodged plants.	null	Lodg	LOD	Plant	Lodging incidence	NA	NA	CO_321:0000355	Lodg Estimation	Estimation	[illegible]	null
CO_321:0001282	Lodg_E_0to9pct	LODG_0_9	Trial evaluation	After the event	null	BMS_29117	CIMMYT	Rosemary Shrestha; Julian Pietragalla	25-oct.-2015	EN	Wheat	CO_321:0000167	Lodging incidence	Agronomical traits	Indicates incidence of lodged plants.	null	Lodg	LOD	Plant	Lodging incidence	NA	NA	CO_321:0000355	Lodg Estimation	Estimation	[illegible]	null
CO_321:0001283	Lodg_E_1to5	LODG_1_5, LodgInc_N_T_S_M_V	Trial evaluation	After the event	null	BMS_22681	CIMMYT	Rosemary Shrestha; Julian Pietragalla	25-oct.-2015	EN	Wheat	CO_321:0000167	Lodging incidence	Agronomical traits	Indicates incidence of lodged plants.	null	Lodg	LOD	Plant	Lodging incidence	NA	NA	CO_321:0000355	Lodg Estimation	Estimation	[illegible]	null
CO_321:0001284	Lodg_E_1to9	LODG_1_9	Trial evaluation	After the event	null	BMS_22905	CIMMYT	Rosemary Shrestha; Julian Pietragalla	25-oct.-2015	EN	Wheat	CO_321:0000167	Lodging incidence	Agronomical traits	Indicates incidence of lodged plants.	null	Lodg	LOD	Plant	Lodging incidence	NA	NA	CO_321:0000355	Lodg Estimation	Estimation	[illegible]	null
CO_321:0001285	UtritInc_E_pct	UTRIT_pct__INC	Nursery/Trial evaluation	From DS75	null	BMS_22840	CIMMYT	Rosemary Shrestha; Julian Pietragalla	25-oct.-2015	EN	Wheat	CO_321:0000921	Loose smut incidence	Biotic stress traits	[illegible]	null	UtritInc	UTRIT	Spike	Loose smut incidence	NA	NA	CO_321:0000325	UtritInc Estimation	Estimation	[illegible]	null
CO_321:0001286	UtritInc_E_1to9	UTRIT_1_9__INC	Nursery/Trial evaluation	From DS75	null	BMS_29201	CIMMYT	Rosemary Shrestha; Julian Pietragalla	23-sept.-2014	EN	Wheat	CO_321:0000921	Loose smut incidence	Biotic stress traits	[illegible]	null	UtritInc	UTRIT	Spike	Loose smut incidence	NA	NA	CO_321:0000325	UtritInc Estimation	Estimation	[illegible]	null
CO_321:0001287	Mat_relat_1to6	RELTMT_1_6, RELTMT	Nursery/Trial evaluation	[illegible]	null	BMS_22696	CIMMYT	Rosemary Shrestha; Julian Pietragalla	25-oct.-2015	EN	Wheat	CO_321:0000022	Maturity time	Phenological traits	[illegible]	null	Mat	Mt, M, Maturity	Plant	Maturity time	NA	NA	CO_321:0000243	Mat DS87 relative Estimation	Estimation	[illegible]	null
CO_321:0001288	Mat_date_jd	MATD_jd, MATD, MATDATE	Nursery/Trial evaluation	[illegible]	Obsolete/legacy	BMS_22680	CIMMYT	Rosemary Shrestha; Julian Pietragalla	25-oct.-2015	EN	Wheat	CO_321:0000022	Maturity time	Phenological traits	[illegible]	null	Mat	Mt, M, Maturity	Plant	Maturity time	NA	NA	CO_321:0000842	Mat DS87 date Estimation	Estimation	[illegible]	null
CO_321:0001289	Mat_date_ymd	MATD_yyyymmdd, MATD, MATDATE	Nursery/Trial evaluation	[illegible]	null	BMS_22678	CIMMYT	Rosemary Shrestha; Julian Pietragalla	25-oct.-2015	EN	Wheat	CO_321:0000022	Maturity time	Phenological traits	[illegible]	null	Mat	Mt, M, Maturity	Plant	Maturity time	NA	NA	CO_321:0000842	Mat DS87 date Estimation	Estimation	[illegible]	null
CO_321:0001290	Mat_dto_day	DTM_days, DTM, DAYSMT, MAT	Nursery/Trial evaluation	[illegible]	null	BMS_22677	CIMMYT	Rosemary Shrestha; Julian Pietragalla	23-sept.-2014	EN	Wheat	CO_321:0000022	Maturity time	Phenological traits	[illegible]	null	Mat	Mt, M, Maturity	Plant	Maturity time	NA	NA	CO_321:0000232	Mat DS87 DT Computation	Computation	[illegible]	xxxx
CO_321:0001291	Mat_relat_1to5	RELTMT_1_5, RELTMT	Nursery/Trial evaluation	[illegible]	null	BMS_29155	CIMMYT	Rosemary Shrestha; Julian Pietragalla	23-sept.-2014	EN	Wheat	CO_321:0000022	Maturity time	Phenological traits	[illegible]	null	Mat	Mt, M, Maturity	Plant	Maturity time	NA	NA	CO_321:0000243	Mat DS87 relative Estimation	Estimation	[illegible]	null
CO_321:0001292	HtereInc_E_pct	HTERE_pct__INC	Nursery/Trial evaluation	[illegible]	null	BMS_22818	CIMMYT	Rosemary Shrestha; Julian Pietragalla	25-oct.-2015	EN	Wheat	CO_321:0000170	Net blotch incidence	Biotic stress traits	[illegible]	null	HtereInc	null	Leaf	Net blotch incidence	NA	NA	CO_321:0000357	HtereInc Estimation	Estimation	[illegible]	null
CO_321:0001293	HtereSev_E_00to99	HTERE_00_99__SEV	Nursery/Trial evaluation	[illegible]	null	BMS_22815	CIMMYT	Rosemary Shrestha; Julian Pietragalla	23-sept.-2014	EN	Wheat	CO_321:0000406	Net blotch severity	Biotic stress traits	[illegible]	null	HtereSev	null	Leaf	Net blotch severity	NA	NA	CO_321:0000690	Double digit Estimation	Estimation	[illegible]	null
CO_321:0001294	NodeNO_Ct_node	NODENO_num	Descriptor	DS61-DS75	null	BMS_29123	CIMMYT	Rosemary Shrestha; Julian Pietragalla	23-sept.-2014	EN	Wheat	CO_321:0000051	Node number	Morphological traits	Number of nodes in the main stem.	null	NodeN	null	Stem	Node number	NA	NA	CO_321:0000271	NodeN Counting	Counting	Standard method for node number.	null
CO_321:0001295	PedGlau_E_0to9	PEDGLAU_0_9	Research-intensive characterization	DS61-DS75	null	BMS_29129	CIMMYT	Rosemary Shrestha; Julian Pietragalla	23-sept.-2014	EN	Wheat	CO_321:0000054	Peduncle glaucousness	Morphological traits	Glaucousness in peduncle.	null	PedGlau	null	Peduncle	Glaucousness	NA	NA	CO_321:0000274	PedGlau Estimation	Estimation	[illegible]	null
CO_321:0001296	PedLng_M_cm	PEDLNG_cm	Research-intensive characterization	DS61-DS75	null	BMS_23053	CIMMYT	Rosemary Shrestha; Julian Pietragalla	23-sept.-2014	EN	Wheat	CO_321:0000052	Peduncle length	Morphological traits	Length of peduncle.	null	PedLng	null	Peduncle	Length	NA	NA	CO_321:0000272	PedLng Measurement	Measurement	[illegible]	null
CO_321:0001297	NeckBreak_E_pct	NBREAK_pct	Nursery/Trial evaluation	null	null	BMS_29121	CIMMYT	Rosemary Shrestha; Julian Pietragalla	23-sept.-2014	EN	Wheat	CO_321:0000050	Peduncle neck break	Agronomical traits	[illegible]	null	NeckBreak	NBreak	Peduncle	Neck break	NA	NA	CO_321:0000270	NeckBreak Estimation	Estimation	Standard method for neck break.	null
CO_321:0001298	PedPubDen_E_0to9	PEDPBD_0_9	Research-intensive characterization	DS61-DS75	null	BMS_29130	CIMMYT	Rosemary Shrestha; Julian Pietragalla	23-sept.-2014	EN	Wheat	CO_321:0000053	Peduncle pubescence density	Morphological traits	Peduncle hairiness density.	null	PedPubDen	PEDPBD	Peduncle	Pubescence density	NA	NA	CO_321:0000273	PedPubDen Estimation	Estimation	[illegible]	null
CO_321:0001299	ErlVig_E_1to5	ERLVIG_1_5	Trial evaluation	DS13-DS23	null	BMS_22682	CIMMYT	Rosemary Shrestha; Julian Pietragalla	23-sept.-2014	EN	Wheat	CO_321:0000009	Plant early vigour	Agronomical traits	[illegible]	Seedling vigour, Plant early vigor	ErlVig	ERLVIG	Plant	Early vigour	NA	NA	CO_321:0000233	ErlVig Estimation	Estimation	[illegible]	null
CO_321:0001300	GrwHabit_E_1to5	GHABIT_1_5	Descriptor	DS25-DS30	null	BMS_23213	CIMMYT	Rosemary Shrestha; Julian Pietragalla	23-sept.-2014	EN	Wheat	CO_321:0000015	Plant growth habit	Agronomical traits	[illegible]	null	GrwHabit	GHabit	Plant	Growth habit	NA	NA	CO_321:0000238	GrwHabit Estimation	Estimation	[illegible]	null
CO_321:0001301	PH_M_cm	PH_cm	Nursery/Trial evaluation	DS75-DS89	null	BMS_18020	CIMMYT	Rosemary Shrestha; Julian Pietragalla	23-sept.-2014	EN	Wheat	CO_321:0000020	Plant height	Agronomical traits	[illegible]	null	PH	PlntHt, PHt	Plant	Height	NA	NA	CO_321:0000241	PH Measurement	Measurement	[illegible]	null
CO_321:0001302	PH_M_m	PH_m	Nursery/Trial evaluation	DS75-DS89	null	BMS_22673	CIMMYT	Rosemary Shrestha; Julian Pietragalla	25-oct.-2015	EN	Wheat	CO_321:0000020	Plant height	Agronomical traits	[illegible]	null	PH	PlntHt, PHt	Plant	Height	NA	NA	CO_321:0000241	PH Measurement	Measurement	[illegible]	null
CO_321:0001303	PH_E_1to5	RELTPH_1_5	Nursery/Trial evaluation	DS75-DS89	null	BMS_22672	CIMMYT	Rosemary Shrestha; Julian Pietragalla	23-sept.-2014	EN	Wheat	CO_321:0000020	Plant height	Agronomical traits	[illegible]	null	PH	PlntHt, PHt	Plant	Height	NA	NA	CO_321:0000245	PH Estimation	Estimation	[illegible]	null
CO_321:0001304	HNecro_E_0to1	HNECRO_0_1	Nursery/Trial evaluation	[illegible]	null	BMS_29096	CIMMYT	Rosemary Shrestha; Julian Pietragalla	23-sept.-2014	EN	Wheat	CO_321:0000186	Plant hybrid necrosis incidence	Agronomical traits	Observation of hybrid necrosis.	Dwarf clumping	HNecro	null	Plant	Hybrid necrosis incidence	NA	NA	CO_321:0000367	HNecro Estimation	Estimation	[illegible]	null
CO_321:0001305	PhotopResp_E_1to9	PHOTORESPO_1_9	Descriptor	DS65	null	BMS_29136	CIMMYT	Rosemary Shrestha; Julian Pietragalla	25-oct.-2015	EN	Wheat	CO_321:0000672	Plant photoperiod response	Physiological traits	[illegible]	Daylength sensitivity	PhotopResp	PhotoRespo	Plant	Photoperiod response	NA	NA	CO_321:0000846	PhotopResp Estimation	Estimation	[illegible]	null
CO_321:0001306	PlntStnd_Calc_Plntha	PLNTSTND_pl_ha	Trial evaluation	DS10-DS89	null	BMS_29138	CIMMYT	Rosemary Shrestha; Julian Pietragalla	25-oct.-2015	EN	Wheat	CO_321:0000187	Plant stand	Agronomical traits	Plant population or plant density.	[illegible]	PlntStnd	PStand	Plant	Stand	NA	NA	CO_321:0000955	PlntStnd Computation	Computation	Calculate number of plant per area.	xxxx
CO_321:0001307	PlntStnd_Calc_Plntm2	PLNTSTND_pl_m2	Trial evaluation	DS10-DS89	null	BMS_29140	CIMMYT	Rosemary Shrestha; Julian Pietragalla	25-oct.-2015	EN	Wheat	CO_321:0000187	Plant stand	Agronomical traits	Plant population or plant density.	[illegible]	PlntStnd	PStand	Plant	Stand	NA	NA	CO_321:0000955	PlntStnd Computation	Computation	Calculate number of plant per area.	xxxx
CO_321:0001308	PlntStnd_Ct_Plntm	PLNTSTND_pl_m	Trial evaluation	DS10-DS89	null	BMS_29139	CIMMYT	Rosemary Shrestha; Julian Pietragalla	25-oct.-2015	EN	Wheat	CO_321:0000187	Plant stand	Agronomical traits	Plant population or plant density.	[illegible]	PlntStnd	PStand	Plant	Stand	NA	NA	CO_321:0000368	PlntStnd Counting	Counting	[illegible]	null
CO_321:0001309	PlntStnd_Ct_PlntPlot	PLNTSTND_pl_plot	Trial evaluation	DS10-DS89	null	BMS_18070	CIMMYT	Rosemary Shrestha; Julian Pietragalla	23-sept.-2014	EN	Wheat	CO_321:0000187	Plant stand	Agronomical traits	Plant population or plant density.	[illegible]	PlntStnd	PStand	Plant	Stand	NA	NA	CO_321:0000368	PlntStnd Counting	Counting	[illegible]	null
CO_321:0001310	VgP_calc_day	VEGPERIOD_days	Research-intensive characterization	DS10-DS31	null	BMS_29202	CIMMYT	Rosemary Shrestha; Julian Pietragalla	25-oct.-2015	EN	Wheat	CO_321:0000670	Plant vegetative period	Phenological traits	[illegible]	null	VgP	VegPeriod, VegP, VP	Plant	Vegetative period	NA	NA	CO_321:0001429	VgP Computation	Computation	[illegible]	xxxx
CO_321:0001311	VgP_calc_pct	VEGPERIOD_pct	Research-intensive characterization	DS10-DS31	null	BMS_29203	CIMMYT	Rosemary Shrestha; Julian Pietragalla	25-oct.-2015	EN	Wheat	CO_321:0000670	Plant vegetative period	Phenological traits	[illegible]	null	VgP	VegPeriod, VegP, VP	Plant	Vegetative period	NA	NA	CO_321:0001429	VgP Computation	Computation	[illegible]	xxxx
CO_321:0001312	PlntVig_E_1to5	PLNTVIG_1_5	Trial evaluation	DS13-DS81	null	BMS_18080	CIMMYT	Rosemary Shrestha; Julian Pietragalla	25-oct.-2015	EN	Wheat	CO_321:0000680	Plant vigour	Agronomical traits	Capacity of the plant to grow.	Plant vigour	PlntVig	Vg	Plant	Vigour	NA	NA	CO_321:0001428	PlntVig Estimation	Estimation	[illegible]	null
CO_321:0001313	EgramInc_E_0to9pcttext	EGRAM_0_9text__INC	Nursery/Trial evaluation	[illegible]	Obsolete/legacy	BMS_22804	CIMMYT	Rosemary Shrestha; Julian Pietragalla	25-oct.-2015	EN	Wheat	CO_321:0000937	Powdery mildew incidence	Biotic stress traits	[illegible]	null	EgramInc	EGRAM	Leaf	Powdery mildew incidence	NA	NA	CO_321:0000330	EgramInc Estimation	Estimation	[illegible]	null
CO_321:0001314	EgramInc_E_pct	EGRAM_pct__INC	Nursery/Trial evaluation	[illegible]	null	BMS_22805	CIMMYT	Rosemary Shrestha; Julian Pietragalla	25-oct.-2015	EN	Wheat	CO_321:0000937	Powdery mildew incidence	Biotic stress traits	[illegible]	null	EgramInc	EGRAM	Leaf	Powdery mildew incidence	NA	NA	CO_321:0000330	EgramInc Estimation	Estimation	[illegible]	null
CO_321:0001315	EgramInc_E_0to5	EGRAM_0_5__INC	Nursery/Trial evaluation	[illegible]	null	BMS_22807	CIMMYT	Rosemary Shrestha; Julian Pietragalla	25-oct.-2015	EN	Wheat	CO_321:0000937	Powdery mildew incidence	Biotic stress traits	[illegible]	null	EgramInc	EGRAM	Leaf	Powdery mildew incidence	NA	NA	CO_321:0000330	EgramInc Estimation	Estimation	[illegible]	null
CO_321:0001316	EgramInc_E_1to5	EGRAM_1_5__INC	Nursery/Trial evaluation	[illegible]	null	BMS_22810	CIMMYT	Rosemary Shrestha; Julian Pietragalla	25-oct.-2015	EN	Wheat	CO_321:0000937	Powdery mildew incidence	Biotic stress traits	[illegible]	null	EgramInc	EGRAM	Leaf	Powdery mildew incidence	NA	NA	CO_321:0000330	EgramInc Estimation	Estimation	[illegible]	null

[illegible]

CO_331:0000692	FrHab_Et_0to7	FLWHAB	Characterizing stocks	RG	Standard AVRDC,IBPBR,CIP	Huaman1991:Def_4.3.1	CIP, FAO, IPGRI	Z. Huaman	26/04/2016	EN	Sweetpotato	CO_331:0000328	Flowering habit	Morphological trait	[illegible]	null	FrHab	Flower	Number	Recommended	Huaman1991:Def_4.3.1	CO_331:0000444	[illegible]	Estimation	Visual estimation	null
CO_331:0000693	FrSiz_Ms_cm	FLESIZ	Characterizing stocks	RG	Standard AVRDC,IBPBR,CIP	Huaman1991:Def_4.3.3	CIP, FAO, IPGRI	Z. Huaman	26/04/2016	EN	Sweetpotato	CO_331:0000329	Flower size	Morphological trait	Flower size	null	FrSiz	Flower	Size	Recommended	Huaman1991:Def_4.3.3	CO_331:0000445	[illegible]	Measurement	null	null
CO_331:0000694	FrShL_Et_3to7	FLLMB	Characterizing stocks	RG	Standard AVRDC,IBPBR,CIP	Huaman1991:Def_4.3.4	CIP, FAO, IPGRI	Z. Huaman	26/04/2016	EN	Sweetpotato	CO_331:0000330	Shape of limb	Morphological trait	Shape of limb	null	FrShL	Limb flower	Shape	Recommended	Huaman1991:Def_4.3.4	CO_331:0000446	[illegible]	Estimation	Visual estimation	null
CO_331:0000695	FrSepEql_Et_1to2	SEPLEQ	Characterizing stocks	RG	Standard AVRDC,IBPBR,CIP	Huaman1991:Def_4.3.5	CIP, FAO, IPGRI	Z. Huaman	26/04/2016	EN	Sweetpotato	CO_331:0000331	Equality of sepal length	Morphological trait	Equality of sepal length	null	FrSepEql	Sepal	Length	Recommended	Huaman1991:Def_4.3.5	CO_331:0000447	Equality of sepal length	Estimation	Visual estimation	null
CO_331:0000696	FrSepNVn_Ct_perSpl	SEPVNM	Characterizing stocks	RG	Standard AVRDC,IBPBR,CIP	Huaman1991:Def_4.3.6	CIP, FAO, IPGRI	Z. Huaman	26/04/2016	EN	Sweetpotato	CO_331:0000332	Number of sepal veins	Morphological trait	[illegible]	null	FrSepNVn	Sepal	Number veins	Recommended	Huaman1991:Def_4.3.6	CO_331:0000448	[illegible]	Estimation	Visual estimation	null
CO_331:0000697	FrSepShp_Et_1to9	SEPSHP	Characterizing stocks	RG	Standard AVRDC,IBPBR,CIP	Huaman1991:Def_4.3.7	CIP, FAO, IPGRI	Z. Huaman	26/04/2016	EN	Sweetpotato	CO_331:0000333	Sepal shape	Morphological trait	Sepal shape	null	FrSepShp	Sepal	Shape	Recommended	Huaman1991:Def_4.3.7	CO_331:0000449	Observation of Sepal shape	Estimation	Visual estimation	null
CO_331:0000698	FrSepApx_Et_1to7	SEPAPX	Characterizing stocks	RG	Standard AVRDC,IBPBR,CIP	Huaman1991:Def_4.3.8	CIP, FAO, IPGRI	Z. Huaman	26/04/2016	EN	Sweetpotato	CO_331:0000334	Sepal apex	Morphological trait	Sepal apex	null	FrSepApx	Sepal	Apex	Recommended	Huaman1991:Def_4.3.8	CO_331:0000450	Observation of Sepal apex	Estimation	Visual estimation	null
CO_331:0000699	FrSepPub_Et_0to7	SEPPUB	Characterizing stocks	RG	Standard AVRDC,IBPBR,CIP	Huaman1991:Def_4.3.9	CIP, FAO, IPGRI	Z. Huaman	26/04/2016	EN	Sweetpotato	CO_331:0000335	Sepal pubescence	Morphological trait	[illegible]	null	FrSepPub	Sepal	Pubescence	Recommended	Huaman1991:Def_4.3.9	CO_331:0000451	Observation of Sepal pubescence	Estimation	Visual estimation	null
CO_331:0000700	FrSepCol_Et_3to7	SEPCOL	Characterizing stocks	RG	Standard AVRDC,IBPBR,CIP	Huaman1991:Def_4.3.10	CIP, FAO, IPGRI	Z. Huaman	26/04/2016	EN	Sweetpotato	CO_331:0000336	Sepal color	Morphological trait	[illegible]	null	FrSepCol	Sepal	Color	Recommended	Huaman1991:Def_4.3.10	CO_331:0000452	Observation of Sepal color	Estimation	Visual estimation	null
CO_331:0000701	FrStgCol_Et_1to9	STGCOL	Characterizing stocks	RG	Standard AVRDC,IBPBR,CIP	Huaman1991:Def_4.3.11	CIP, FAO, IPGRI	Z. Huaman	42492	EN	Sweetpotato	CO_331:0000337	Color of stigma	Morphological trait	Color of stigma	null	FrStgCol	Stigma	Color	Recommended	Huaman1991:Def_4.3.11	CO_331:0000453	Observation of Stigma color	Estimation	Visual estimation	null
CO_331:0000702	FrStyCol_Et_1to9	STYCOL	Characterizing stocks	RG	Standard AVRDC,IBPBR,CIP	Huaman1991:Def_4.3.12	CIP, FAO, IPGRI	Z. Huaman	42492	EN	Sweetpotato	CO_331:0000338	Color of style	Morphological trait	Color of style	null	FrStyCol	Style	Color	Recommended	Huaman1991:Def_4.3.12	CO_331:0000454	Observation of Style color	Estimation	Visual estimation	null
CO_331:0000703	FrStgExt_Et_1to7	STGEXT	Characterizing stocks	RG	Standard AVRDC,IBPBR,CIP	Huaman1991:Def_4.3.13	CIP, FAO, IPGRI	Z. Huaman	42492	EN	Sweetpotato	CO_331:0000339	Stigma exertion	Morphological trait	[illegible]	null	FrStgExt	Stigma	exertion	Recommended	Huaman1991:Def_4.3.13	CO_331:0000455	Observation of Stigma exertion	Estimation	Visual estimation	null
CO_331:0000704	FrSdCp_Et_0to7	SEDCAP	Characterizing stocks	RG	Standard AVRDC,IBPBR,CIP	Huaman1991:Def_4.3.14	CIP, FAO, IPGRI	Z. Huaman	42492	EN	Sweetpotato	CO_331:0000340	Seed capsule set	Morphological trait	Seed capsule set	null	FrSdCp	Seed	capsule	Recommended	Huaman1991:Def_4.3.14	CO_331:0000456	Observation of Seed capsule set	Estimation	Visual estimation	null
CO_331:0000705	RtFrm_Et_1to7	RTFORM	Characterizing stocks	SD	Standard AVRDC,IBPBR,CIP	Huaman1991:Def_6.1.1	CIP, FAO, IPGRI	Z. Huaman	42492	EN	Sweetpotato	CO_331:0000341	Storage root formation	Morphological trait	[illegible]	null	RtFrm	Root	formation	Recommended	Huaman1991:Def_6.1.1	CO_331:0000457	[illegible]	Estimation	Visual estimation	null
CO_331:0000706	RtStk_Et_0to9	RTSTFM	Characterizing stocks	SD	Standard AVRDC,IBPBR,CIP	Huaman1991:Def_6.1.2	CIP, FAO, IPGRI	Z. Huaman	42492	EN	Sweetpotato	CO_331:0000342	Storage root stalk	Morphological trait	[illegible]	null	RtStk	Root	talk	Recommended	Huaman1991:Def_6.1.2	CO_331:0000458	Observation of Storage root stalk	Estimation	Visual estimation	null
CO_331:0000707	RtShV_Et_3to7	RTSHPV	Characterizing stocks	RB	Standard AVRDC,IBPBR,CIP	Huaman1991:Def_6.1.4	CIP, FAO, IPGRI	Z. Huaman	42492	EN	Sweetpotato	CO_331:0000343	Variability of storage root shape	Morphological trait	Variability of storage root shape	null	RtShV	Root	Shape variability	Recommended	Huaman1991:Def_6.1.4	CO_331:0000459	[illegible]	Estimation	Visual estimation	null
CO_331:0000708	RtSzV_Et_3to7	RTSIZV	Characterizing stocks	RB	Standard AVRDC,IBPBR,CIP	Huaman1991:Def_6.1.5	CIP, FAO, IPGRI	Z. Huaman	42492	EN	Sweetpotato	CO_331:0000344	Variability of storage root size	Morphological trait	Variability of storage root size	null	RtSzV	Root	Size variability	Recommended	Huaman1991:Def_6.1.5	CO_331:0000460	[illegible]	Estimation	Visual estimation	null
CO_331:0000709	RtKQl_Et_3to7	RTKQL	Evaluation in Trials	RB	Standard AVRDC,IBPBR,CIP	Huaman1991:Def_6.2.7	CIP, FAO, IPGRI	Z. Huaman	42492	EN	Sweetpotato	CO_331:0000345	Keeping quality of storage roots	Morphological trait	Keeping quality of storage roots	null	RtKQl	Root	quality	Recommended	Huaman1991:Def_6.2.7	CO_331:0000461	[illegible]	Estimation	Visual estimation	null
CO_331:0000710	RtBlCu_Et_1to9	RTBCOLD	Evaluation in Trials	RB	Standard AVRDC,IBPBR,CIP	Huaman1991:Def_6.2.9.1	CIP, FAO, IPGRI	Z. Huaman	42492	EN	Sweetpotato	CO_331:0000346	Consistency of boiled storage root	Morphological trait	Consistency of boiled storage root	null	RtBlCu	Root boiled	Consistency	Recommended	Huaman1991:Def_6.2.9.1	CO_331:0000462	[illegible]	Estimation	Visual estimation	null
CO_331:0000711	RtBlCd_Et_0to9	RTBCOLU	Evaluation in Trials	RB	Standard AVRDC,IBPBR,CIP	Huaman1991:Def_6.2.9.2	CIP, FAO, IPGRI	Z. Huaman	42492	EN	Sweetpotato	CO_331:0000347	[illegible]	Morphological trait	[illegible]	null	RtBlCd	Root boiled	color	Recommended	Huaman1991:Def_6.2.9.2	CO_331:0000463	[illegible]	Estimation	Visual estimation	null
CO_331:0000712	RnDrt_Et_1to9	DROUGHT	Evaluation in Trials	RG	Standard AVRDC,IBPBR,CIP	Huaman1991:Def_7.1	CIP, FAO, IPGRI	Z. Huaman	42492	EN	Sweetpotato	CO_331:0000348	Reaction to drought	Abiotic stress trait	[illegible]	null	RnDrt	Plant	Drought tolerance	Recommended	Huaman1991:Def_7.1	CO_331:0000464	[illegible]	Estimation	[illegible]	null
CO_331:0000713	RnFld_Et_1to9	FLOOD	Evaluation in Trials	RG	Standard AVRDC,IBPBR,CIP	Huaman1991:Def_7.2	CIP, FAO, IPGRI	Z. Huaman	42492	EN	Sweetpotato	CO_331:0000349	Reaction to flooding	Abiotic stress trait	[illegible]	waterlogging response	RnFld	Plant	Flooding tolerance	Recommended	Huaman1991:Def_7.2	CO_331:0000465	[illegible]	Estimation	[illegible]	null
CO_331:0000714	RnHeat_Et_1to9	HEAT	Evaluation in Trials	RG	Standard AVRDC,IBPBR,CIP	Huaman1991:Def_7.3	CIP, FAO, IPGRI	Z. Huaman	42492	EN	Sweetpotato	CO_331:0000350	Reaction to heat	Abiotic stress trait	[illegible]	null	RnHeat	Plant	Heat tolerance	Recommended	Huaman1991:Def_7.3	CO_331:0000466	[illegible]	Estimation	[illegible]	null
CO_331:0000715	RnSlt_Et_1to9	SALINITY	Evaluation in Trials	RG	Standard AVRDC,IBPBR,CIP	Huaman1991:Def_7.4	CIP, FAO, IPGRI	Z. Huaman	42492	EN	Sweetpotato	CO_331:0000351	Reaction to salinity	Abiotic stress trait	[illegible]	null	RnSlt	Plant	Salinity tolerance	Recommended	Huaman1991:Def_7.4	CO_331:0000467	[illegible]	Estimation	[illegible]	null
CO_331:0000716	RnShd_Et_1to9	SHADE	Evaluation in Trials	RG	Standard AVRDC,IBPBR,CIP	Huaman1991:Def_7.5	CIP, FAO, IPGRI	Z. Huaman	42492	EN	Sweetpotato	CO_331:0000352	Reaction to shade	Abiotic stress trait	[illegible]	null	RnShd	Plant	Shade tolerance	Recommended	Huaman1991:Def_7.5	CO_331:0000468	[illegible]	Estimation	[illegible]	null
CO_331:0000717	RnSph_Et_1to9	SOILPH	Evaluation in Trials	RG	Standard AVRDC,IBPBR,CIP	Huaman1991:Def_7.6	CIP, FAO, IPGRI	Z. Huaman	42492	EN	Sweetpotato	CO_331:0000353	Reaction to soil	Abiotic stress trait	[illegible]	null	RnSph	Plant	Soil tolerance	Recommended	Huaman1991:Def_7.6	CO_331:0000469	[illegible]	Estimation	[illegible]	null
CO_331:0000718	RnSTp_Et_1to9	SOILTEMP	Evaluation in Trials	RG	Standard AVRDC,IBPBR,CIP	Huaman1991:Def_7.7	CIP, FAO, IPGRI	Z. Huaman	42492	EN	Sweetpotato	CO_331:0000354	Reaction to high soil temperature	Abiotic stress trait	[illegible]	null	RnSTp	Plant	Temperature soiltolerance	Recommended	Huaman1991:Def_7.7	CO_331:0000470	[illegible]	Estimation	[illegible]	null
CO_331:0000719	RnWISPW_Et_1to9	WISPWV	Evaluation in Trials	RG	Standard AVRDC,IBPBR,CIP	Huaman1991:Def_8.1.2	CIP, FAO, IPGRI	Z. Huaman	42492	EN	Sweetpotato	CO_331:0000355	[illegible]	Biotic stress trait	[illegible]	null	RnWISPW	Plant	Reaction to west indian weevil	Recommended	Huaman1991:Def_8.1.2	CO_331:0000471	[illegible]	Estimation	[illegible]	null
CO_331:0000720	RnSSPW_Et_1to9	STSPWV	Evaluation in Trials	RG	Standard AVRDC,IBPBR,CIP	Huaman1991:Def_8.1.3	CIP, FAO, IPGRI	Z. Huaman	42492	EN	Sweetpotato	CO_331:0000356	[illegible]	Biotic stress trait	[illegible]	null	RnSSPW	Plant	Reaction to Striped weevil	Recommended	Huaman1991:Def_8.1.3	CO_331:0000472	[illegible]	Estimation	[illegible]	null
CO_331:0000721	RnSPWW_Et_1to9	SPWW	Evaluation in Trials	RG	Standard AVRDC,IBPBR,CIP	Huaman1991:Def_8.1.4	CIP, FAO, IPGRI	Z. Huaman	42492	EN	Sweetpotato	CO_331:0000357	Reaction to Sweet potato wire worms	Biotic stress trait	Reaction to Sweet potato wire worms	null	RnSPWW	Plant	Reaction to Sweet potato wire worms	Recommended	Huaman1991:Def_8.1.4	CO_331:0000473	[illegible]	Estimation	[illegible]	null
CO_331:0000722	RnWW_Et_1to9	WIREWORMS	Evaluation in Trials	RG	Standard AVRDC,IBPBR,CIP	Huaman1991:Def_8.1.5	CIP, FAO, IPGRI	Z. Huaman	42492	EN	Sweetpotato	CO_331:0000358	Reaction to Wire worms	Biotic stress trait	Reaction to Wire worms	null	RnWW	Plant	Reaction to wire worms	Recommended	Huaman1991:Def_8.1.5	CO_331:0000474	[illegible]	Estimation	[illegible]	null
CO_331:0000723	RnSPFB_Et_1to9	SPFB	Evaluation in Trials	RG	Standard AVRDC,IBPBR,CIP	Huaman1991:Def_8.1.6	CIP, FAO, IPGRI	Z. Huaman	42492	EN	Sweetpotato	CO_331:0000359	Reaction to Sweet potato flea beetles	Biotic stress trait	Reaction to Sweet potato flea beetles	null	RnSPFB	Plant	Reaction to Sweet potato flea beetles	Recommended	Huaman1991:Def_8.1.6	CO_331:0000475	[illegible]	Estimation	[illegible]	null
CO_331:0000724	RnFB_Et_1to9	FLEABEETLE	Evaluation in Trials	RG	Standard AVRDC,IBPBR,CIP	Huaman1991:Def_8.1.7	CIP, FAO, IPGRI	Z. Huaman	42492	EN	Sweetpotato	CO_331:0000360	Reaction to flea beetles	Biotic stress trait	Reaction to flea beetles	null	RnFB	Plant	Reaction to flea beetles	Recommended	Huaman1991:Def_8.1.7	CO_331:0000476	[illegible]	Estimation	[illegible]	null
CO_331:0000725	RnSPLB_Et_1to9	SPFB	Evaluation in Trials	RG	Standard AVRDC,IBPBR,CIP	Huaman1991:Def_8.1.8	CIP, FAO, IPGRI	Z. Huaman	42492	EN	Sweetpotato	CO_331:0000361	Reaction to Sweet potato leaf beetles	Biotic stress trait	Reaction to Sweet potato leaf beetles	null	RnSPLB	Plant	Reaction to Sweet potato leaf beetles	Recommended	Huaman1991:Def_8.1.8	CO_331:0000477	[illegible]	Estimation	[illegible]	null
CO_331:0000726	RnBTL_Et_1to9	BEETLES	Evaluation in Trials	RG	Standard AVRDC,IBPBR,CIP	Huaman1991:Def_8.1.9	CIP, FAO, IPGRI	Z. Huaman	42492	EN	Sweetpotato	CO_331:0000362	Reaction to Beetles	Biotic stress trait	Reaction to Beetles	null	RnBTL	Plant	Reaction to Beetles	Recommended	Huaman1991:Def_8.1.9	CO_331:0000478	[illegible]	Estimation	[illegible]	null
CO_331:0000727	RnGrbW_Et_1to9	GRUBW	Evaluation in Trials	RG	Standard AVRDC,IBPBR,CIP	Huaman1991:Def_8.1.10	CIP, FAO, IPGRI	Z. Huaman	42492	EN	Sweetpotato	CO_331:0000363	Reaction to Grubworm	Biotic stress trait	[illegible]	null	RnGrbW	Root	Reaction to Grub worm	Recommended	Huaman1991:Def_8.1.10	CO_331:0000479	[illegible]	Estimation	[illegible]	null
CO_331:0000728	RnHrnw_Et_1to9	HORNW	Evaluation in Trials	RG	Standard AVRDC,IBPBR,CIP	Huaman1991:Def_8.1.11	CIP, FAO, IPGRI	Z. Huaman	42492	EN	Sweetpotato	CO_331:0000364	Reaction to Hornworm	Biotic stress trait	[illegible]	null	RnHrnw	Root	Reaction to Horn worm	Recommended	Huaman1991:Def_8.1.11	CO_331:0000480	[illegible]	Estimation	[illegible]	null
CO_331:0000729	RnAph_Et_1to9	APHIDS	Evaluation in Trials	RG	Standard AVRDC,IBPBR,CIP	Huaman1991:Def_8.1.12	CIP, FAO, IPGRI	Z. Huaman	42492	EN	Sweetpotato	CO_331:0000365	Reaction to Aphids	Biotic stress trait	[illegible]	null	RnAph	Plant	Reaction to Aphids	Recommended	Huaman1991:Def_8.1.12	CO_331:0000481	[illegible]	Estimation	[illegible]	null
CO_331:0000730	RnSPWF_Et_1to9	SPWF	Evaluation in Trials	RG	Standard AVRDC,IBPBR,CIP	Huaman1991:Def_8.1.13	CIP, FAO, IPGRI	Z. Huaman	42492	EN	Sweetpotato	CO_331:0000366	Reaction to Sweet potato white fly	Biotic stress trait	[illegible]	null	RnSPWF	Plant	Reaction to Sweet potato white fly	Recommended	Huaman1991:Def_8.1.13	CO_331:0000482	[illegible]	Estimation	[illegible]	null
[illegible]																										

CO_325:0000585	BlkLfStrkSwtLfNb_Ct_grd2	null	[illegible]	[illegible]	null	null	Bioversity International	Rhiannon Crichton	null		EN	Banana	CO_325:0000584	[illegible]	Biotic stress	[illegible]	[illegible]	null		Plant	[illegible]	null	null	CO_325:0010184	[illegible]	Computation	[illegible]	null
CO_325:0000587	BlkLfStrkSwtLfNb_Ct_grd3	null	[illegible]	[illegible]	null	null	Bioversity International	Rhiannon Crichton	null		EN	Banana	CO_325:0000586	[illegible]	Biotic stress	[illegible]	[illegible]	null		Plant	[illegible]	null	null	CO_325:0010185	[illegible]	Computation	[illegible]	null
CO_325:0000589	BlkLfStrkSwtLfNb_Ct_grd4	null	[illegible]	[illegible]	null	null	Bioversity International	Rhiannon Crichton	null		EN	Banana	CO_325:0000588	[illegible]	Biotic stress	[illegible]	[illegible]	null		Plant	[illegible]	null	null	CO_325:0010186	[illegible]	Computation	[illegible]	null
CO_325:0000591	BlkLfStrkSwtLfNb_Ct_grd5	null	[illegible]	[illegible]	null	null	Bioversity International	Rhiannon Crichton	null		EN	Banana	CO_325:0000590	[illegible]	Biotic stress	[illegible]	[illegible]	null		Plant	[illegible]	null	null	CO_325:0010187	[illegible]	Computation	[illegible]	null
CO_325:0000593	BlkLfStrkSwtLfNb_Ct_grd6	null	[illegible]	[illegible]	null	null	Bioversity International	Rhiannon Crichton	null		EN	Banana	CO_325:0000592	[illegible]	Biotic stress	[illegible]	[illegible]	null		Plant	[illegible]	null	null	CO_325:0010188	[illegible]	Computation	[illegible]	null
CO_325:0000595	CrmDiscDistTlstSckFW_M_cm	null	[illegible]	Harvest	null	null	Bioversity International	Inge Van den Bergh	null		EN	Banana	CO_325:0000594	[illegible]	Biotic stress	[illegible]	null	null		Pseudostem of tallest sucker	Distance of discolouration	null	null	CO_325:0000594	[illegible]	Measurement	[illegible]	null
CO_325:0000596	YellowPres_Est_0or1	null	[illegible]	[illegible]	null	null	Bioversity International	Inge Van den Bergh		41313	EN	Banana	CO_325:0000087	[illegible]	Biotic stress	[illegible]	null	YL		Leaf	Presence/absence of yellowing	null	null	CO_325:0010190	[illegible]	Estimation	Visual assessment.	null
CO_325:0000597	PStmSplitPres_Est_0or1	null	[illegible]	[illegible]	null	null	Bioversity International	Inge Van den Bergh		41313	EN	Banana	CO_325:0000090	[illegible]	Biotic stress	[illegible]	null	SP		Pseudostem base	Presence/absence of splitting	null	null	CO_325:0010191	[illegible]	Estimation	Visual assessment.	null
CO_325:0000598	LfChgPres_Est_0or1	null	[illegible]	[illegible]	null	null	Bioversity International	Inge Van den Bergh		41313	EN	Banana	CO_325:0000093	[illegible]	Biotic stress	[illegible]	null	CNL		Leaf	Presence/absence of changes	null	null	CO_325:0010192	[illegible]	Estimation	Visual assessment.	null
CO_325:0000599	PetColpPres_Est_0or1	null	[illegible]	[illegible]	null	null	Bioversity International	Inge Van den Bergh		41313	EN	Banana	CO_325:0000096	[illegible]	Biotic stress	[illegible]	null	PC		Petiole	Presence/absence of collapse	null	null	CO_325:0010171	[illegible]	Estimation	Visual assessment.	null
CO_325:0000601	YlLfPercFW_Comp_pct	null	[illegible]	[illegible]	null	null	Bioversity International	Rhiannon Crichton	null		EN	Banana	CO_325:0000600	[illegible]	Biotic stress	[illegible]	null	null		Plant	[illegible]	null	null	CO_325:0010194	[illegible]	Computation	[illegible]	null
CO_325:0000603	SptPsdStmPercFW_Comp_pct	null	[illegible]	[illegible]	null	null	Bioversity International	Rhiannon Crichton	null		EN	Banana	CO_325:0000602	[illegible]	Biotic stress	[illegible]	null	null		Plant	[illegible]	null	null	CO_325:0010195	[illegible]	Computation	[illegible]	null
CO_325:0000605	ChgNwLfPercFW_Comp_pct	null	[illegible]	[illegible]	null	null	Bioversity International	Rhiannon Crichton	null		EN	Banana	CO_325:0000604	[illegible]	Biotic stress	[illegible]	null	null		Plant	[illegible]	null	null	CO_325:0010196	[illegible]	Computation	[illegible]	null
CO_325:0000607	PtlClpFW_Comp_pct	null	[illegible]	[illegible]	null	null	Bioversity International	Rhiannon Crichton	null		EN	Banana	CO_325:0000606	[illegible]	Biotic stress	[illegible]	null	null		Plant	[illegible]	null	null	CO_325:0010197	[illegible]	Computation	[illegible]	null
CO_325:0000609	VitCCnt_M_	null	Evaluation - fruit quality	Post-harvest	null	null	IITA	[illegible]	null		EN	Banana	CO_325:0000608	Vitamin C content	Biochemical	[illegible]	null	null		Fruit pulp	Vitamin C content	null	null	CO_325:0010200	Vitamin C - measurement	Measurement	?	null
CO_325:0000611	WvlAdltPrTrp_Ct_weevil	null	Evaluation - weevils	[illegible]	;	IITA**	Tendo Ssali	null		null	null	EN	Banana	CO_325:0000610	[illegible]	Biotic stress	[illegible]		null	null	Banana weevil trap	Number of banana weevil adults	null	null	CO_325:0010201	Number of adult weevils - counting	Counting	
CO_325:0000613	TrpNum_Ct_weeviltrap	null	Evaluation - weevils	[illegible]	;	IITA**	Tendo Ssali	null		null	null	EN	Banana	CO_325:0000612	Number of banana weevil traps used	Biotic stress	How many banana weevil traps used.		null	null	Banana weevil traps	How many	null	null	CO_325:0010202	[illegible]	Counting	
CO_325:0000615	WvlAdlt_Ct_weevil	null	Evaluation - weevils	[illegible]	;	IITA**	Tendo Ssali	null		null	null	EN	Banana	CO_325:0000614	[illegible]	Biotic stress	[illegible]		null	null	Banana weevil trap	[illegible]	null	null	CO_325:0010201	Number of adult weevils - counting	Counting	
CO_325:0000617	WvlLvPrTrp_Ct_weevil	null	Evaluation - weevils	[illegible]	;	IITA**	Tendo Ssali	null		null	null	EN	Banana	CO_325:0000616	[illegible]	Biotic stress	[illegible]		null	null	Banana weevil trap	Number of banana weevil larvae	null	null	CO_325:0010203	Number of weevil larvae - counting	Counting	
CO_325:0000619	WvlLv_Ct_weevil	null	Evaluation - weevils	[illegible]	;	IITA**	Tendo Ssali	null		null	null	EN	Banana	CO_325:0000618	[illegible]	Biotic stress	[illegible]		null	null	Banana weevil trap	[illegible]	null	null	CO_325:0010203	Number of weevil larvae - counting	Counting	
CO_325:0000621	WvlAdltMsrd_Ct_weevil	null	Evaluation - weevils	Detached corm Assay	null	null	IITA	Tendo Ssali	null		EN	Banana	CO_325:0000620	[illegible]	Biotic stress	[illegible]	null	null		Banana weevil adult	Number of adult weevils measured	null	null	CO_325:0010204	Number of adult larvae - counting	Counting	Visual counting method	null
CO_325:0000623	WvlLvMsrd_Ct_weevil	null	Evaluation - weevils	Detached corm Assay	null	null	IITA	Tendo Ssali	null		EN	Banana	CO_325:0000622	[illegible]	Biotic stress	[illegible]	null	null		Banana weevil larvae	[illegible]	null	null	CO_325:0010203	Number of weevil larvae - counting	Counting	Visual counting method	null
CO_325:0000625	WvlAdltHdCps_M_ÁM	null	Evaluation - weevils	Detached corm Assay	null	null	IITA	Tendo Ssali	null		EN	Banana	CO_325:0000624	[illegible]	Biotic stress	[illegible]	null	null		Banana weevil adult	Head capsule width	null	null	CO_325:0010205	[illegible]	Measurement	Measurement under microscope	null
CO_325:0000627	AvgWvlAdltHdCps_M_ÁM	null	Evaluation - weevils	Detached corm Assay	null	null	IITA	Tendo Ssali	null		EN	Banana	CO_325:0000626	[illegible]	Biotic stress	[illegible]	null	null		Banana weevil adult	Average head capsule width	null	null	CO_325:0010205	[illegible]	Measurement	Measurement under microscope	null
CO_325:0000629	WvlAdltBdLgt_M_ÁM	null	Evaluation - weevils	Detached corm Assay	null	null	IITA	Tendo Ssali	null		EN	Banana	CO_325:0000628	Banana weevil adult body length	Biotic stress	[illegible]	null	null		Banana weevil adult	Body length	null	null	CO_325:0010206	Adult weevil body - measurement	Measurement	Electronic weighing method	null
CO_325:0000631	AvgWvlAdltBdLgt_M_ÁM	null	Evaluation - weevils	Detached corm Assay	null	null	IITA	Tendo Ssali	null		EN	Banana	CO_325:0000630	[illegible]	Biotic stress	[illegible]	null	null		Banana weevil adult	Average body length	null	null	CO_325:0010206	Adult weevil body - measurement	Measurement	Electronic weighing method	null
CO_325:0000633	WvlAdltBdWght_M_mg	null	Evaluation - weevils	Detached corm Assay	null	null	IITA	Tendo Ssali	null		EN	Banana	CO_325:0000632	Banana weevil adult body weight	Biotic stress	[illegible]	null	null		Banana weevil adult	Body weight	null	null	CO_325:0010206	Adult weevil body - measurement	Measurement	Electronic weighing method	null
CO_325:0000635	AvgWvlAdltBdWght_M_mg	null	Evaluation - weevils	Detached corm Assay	null	null	IITA	Tendo Ssali	null		EN	Banana	CO_325:0000634	[illegible]	Biotic stress	[illegible]	null	null		Banana weevil adult	Average body weight	null	null	CO_325:0010206	Adult weevil body - measurement	Measurement	Electronic weighing method	null
CO_325:0000637	WvlLvHdCps_M_ÁM	null	Evaluation - weevils	Detached corm Assay	null	null	IITA	Tendo Ssali	null		EN	Banana	CO_325:0000636	[illegible]	Biotic stress	[illegible]	null	null		Banana weevil larvae	Head capsule width	null	null	CO_325:0010207	[illegible]	Measurement	Measurement under microscope	null
CO_325:0000639	AvgWvlLvHdCps_M_ÁM	null	Evaluation - weevils	Detached corm Assay	null	null	IITA	Tendo Ssali	null		EN	Banana	CO_325:0000638	[illegible]	Biotic stress	[illegible]	null	null		Banana weevil larvae	Average head capsule width	null	null	CO_325:0010207	[illegible]	Measurement	Measurement under microscope	null
CO_325:0000641	WvlLvBdLgt_M_ÁM	null	Evaluation - weevils	Detached corm Assay	null	null	IITA	Tendo Ssali	null		EN	Banana	CO_325:0000640	Banana weevil larvae body length	Biotic stress	[illegible]	null	null		Banana weevil larvae	Body length	null	null	CO_325:0010208	[illegible]	Measurement	Measurement under microscope	null
CO_325:0000643	AvgWvlLvBdLgt_M_ÁM	null	Evaluation - weevils	Detached corm Assay	null	null	IITA	Tendo Ssali	null		EN	Banana	CO_325:0000642	[illegible]	Biotic stress	[illegible]	null	null		Banana weevil larvae	Average body length	null	null	CO_325:0010208	[illegible]	Measurement	Measurement under microscope	null
CO_325:0000645	WvlLvBdWght_M_mg	null	Evaluation - weevils	Detached corm Assay	null	null	IITA	Tendo Ssali	null		EN	Banana	CO_325:0000644	Banana weevil larvae body weight	Biotic stress	[illegible]	null	null		Banana weevil larvae	Body weight	null	null	CO_325:0010209	[illegible]	Measurement	Measurement under microscope	null
CO_325:0000647	AvgWvlLvBdWght_M_mg	null	Evaluation - weevils	Detached corm Assay	null	null	IITA	Tendo Ssali	null		EN	Banana	CO_325:0000646	[illegible]	Biotic stress	[illegible]	null	null		Banana weevil larvae	Average body weight	null	null	CO_325:0010209	[illegible]	Measurement	Measurement under microscope	null
CO_325:0000649	DmgIdxInn_M_%	null	Evaluation - weevils	Harvest, or up to 14 days afterwards	null	null	IITA	Tendo Ssali	null		EN	Banana	CO_325:0000648	[illegible]	Biotic stress	[illegible]	Damage index-X-outer	null		Corm	Damage percent X-upper-outer	null	null	CO_325:0010210	[illegible]	Measurement	[illegible]	null
CO_325:0000651	DmgIdxOut_M_%	null	Evaluation - weevils	Harvest, or up to 14 days afterwards	null	null	IITA	Tendo Ssali	null		EN	Banana	CO_325:0000650	[illegible]	Biotic stress	[illegible]	Damage index-X-inner	null		Corm	Damage percent X-upper-inner	null	null	CO_325:0010211	[illegible]	Measurement	[illegible]	null
CO_325:0000661	temp35	null	[illegible]	null	null	null	Bioversity International	Rhiannon Crichton	null		EN	Banana	CO_325:0000063	[illegible]	Biotic stress	[illegible]	[illegible]	DOT		Leaf	[illegible]	null	null	CO_325:0000064	[illegible]	Computation	[illegible]	null
CO_325:0000662	FrtExtLng_M_cm	null	Evaluation - agronomic	Harvest	null	null	Bioversity International	Rhiannon Crichton	null		EN	Banana	CO_325:0000051	Finger external length	Agronomical	[illegible]	Finger dorsal length	fingerextlength		Finger	External length	null	null	CO_325:0000052	Finger external length - measurement	Measurement	[illegible]	null
CO_325:0000664	temp11	null	Evaluation - agronomic	Shooting	null	null	Bioversity International	Rhiannon Crichton	null		EN	Banana	CO_325:0000663	[illegible]	Agronomical	[illegible]	[illegible]	plantcircumf75cm		Plant	[illegible]	null	null	CO_325:0010023	[illegible]	Measurement	[illegible]	null
CO_325:0000666	temp12	null	Evaluation - agronomic	Shooting	null	null	Bioversity International	Rhiannon Crichton	null		EN	Banana	CO_325:0000665	[illegible]	Agronomical	[illegible]	[illegible]	plantcircumf20cm		Plant	[illegible]	null	null	CO_325:0010023	[illegible]	Measurement	[illegible]	null
CO_325:0000668	temp13	null	Evaluation - agronomic	Shooting	null	null	Bioversity International	Rhiannon Crichton	null		EN	Banana	CO_325:0000667	Plant circumference at collar	Agronomical	[illegible]	[illegible]	plantcircumfcollar		Plant	Circumference at collar	null	null	CO_325:0010243	[illegible]	Measurement	[illegible]	null
CO_325:0000670	temp14	null	Evaluation - agronomic	Calculated later - harvest	null	null	Bioversity International	Rhiannon Crichton	null		EN	Banana	CO_325:0000314	Rachis weight	Agronomical	The weight of the rachis.	null	rachisweight		Rachis	Weight	null	null	CO_325:0010253	Rachis weight - computation	Computation	[illegible]	null
CO_325:0000671	temp15	null	Evaluation - agronomic	Harvest	null	null	Bioversity International	Rhiannon Crichton	null		EN	Banana	CO_325:0000316	Total finger weight	Agronomical	[illegible]	Total fruit weight, Total hand weight	totalfingerweight		Hands	Combined weight	null	null	CO_325:0010254	Total finger weight - measurement	Measurement	[illegible]	null
CO_325:0000672	temp16	null	Evaluation - agronomic	Calculated later - harvest	null	null	Bioversity International	Rhiannon Crichton	null		EN	Banana	CO_325:0000316	Total finger weight	Agronomical	[illegible]	Total fruit weight, Total hand weight	totalfingerweight		Hands	Combined weight	null	null	CO_325:0010255	[illegible]	Computation	[illegible]	null
CO_325:0000676	temp10	null	[illegible]	[illegible]	null	null	Bioversity International	Rhiannon Crichton	null		EN	Banana	CO_325:0000675	[illegible]	Agronomical	[illegible]	null	tallsuckerfunctleaves		Tallest sucker	Number of functional leaves	null	null	CO_325:0010275	[illegible]	Counting	[illegible]	null
CO_325:0000678	temp42	null	Evaluation - weevils	Harvest, or up to 14 days afterwards	null	null	IITA	Tendo Ssali	null		EN	Banana	CO_325:0000677	[illegible]	Biotic stress	[illegible]	Damage index-X-inner	null		Corm	Damage percent X-lower-inner	null	null	CO_325:0010289	[illegible]	Measurement	[illegible]	null
CO_325:0000680	temp43	null	Evaluation - weevils	Harvest, or up to 14 days afterwards	null	null	IITA	Tendo Ssali	null		EN	Banana	CO_325:0000679	[illegible]	Biotic stress	[illegible]	Damage index-X-outer	null		Corm	Damage percent X-lower-outer	null	null	CO_325:0010290	[illegible]	Measurement	[illegible]	null
CO_325:0000682	temp44	null	Evaluation - weevils	[illegible]	null	null	IITA	Tendo Ssali	null		EN	Banana	CO_325:0000681	[illegible]	Biotic stress	[illegible]	Damage index-X-average	null		Corm	Damage percent X-average	null	null	CO_325:0010291	[illegible]	Computation	[illegible]	null
CO_325:0000683	temp41	null	Evaluation - weevils	Any as required	null	null	Bioversity International	Rhiannon Crichton	null		EN	Banana	CO_325:0000660	[illegible]	Biotic stress	[illegible]	null	null		Corm	Date of weevil damage assessment	null	null	CO_325:0010303	[illegible]	Estimation	Record the date of event.	null
CO_325:0000685	temp22	null	Evaluation - agronomic	Any as required	null	null	Bioversity International	Rhiannon Crichton	null		EN	Banana	CO_325:0000684	Hand rank of 2nd-most distal hand	Agronomical	[illegible]	null	handid2ndmostdistal		Second-most distal hand	Rank	null	null	CO_325:0010037	Hand rank - counting	Counting	[illegible]	null
CO_325:0000687	temp8	null	Evaluation - agronomic	Any as required	null	null	Bioversity International	Rhiannon Crichton	null		EN	Banana	CO_325:0000686	Start date and time of data collection	Agronomical	[illegible]	null	datestarttimedatacoll		Plant	[illegible]	null	null	CO_325:0010325	[illegible]	Estimation	Record the date and time of event.	null
CO_325:0000689	temp9	null	Evaluation - agronomic	Any as required	null	null	Bioversity International	Rhiannon Crichton	null		EN	Banana	CO_325:0000688	End date and time of data collection	Agronomical	[illegible]	null	dateendtimedatacoll		Plant	[illegible]	null	null	CO_325:0010326	[illegible]	Estimation	Record the date and time of event.	null
[illegible]																												

CO_322:0001027	BwSpotSev_E_1to9	null	Pedigree nurseries/trials	After anthesis	null	NA	CIMMYT
CO_322:0001028	BwSpotSev_E_pct	null	Pedigree nurseries/trials	After anthesis	null	NA	CIMMYT
CO_322:0001029	CRustSev_E_1to9	null	Pedigree nurseries/trials	After anthesis	null	NA	CIMMYT
CO_322:0001030	CRustSev_E_pct	null	Pedigree nurseries/trials	After anthesis	null	NA	CIMMYT
CO_322:0001032	CSmutSev_E_pct	null	Pedigree nurseries/trials	After anthesis	null	NA	CIMMYT
CO_322:0001033	CSmutSev_E_1to9	null	Pedigree nurseries/trials	After anthesis	null	NA	CIMMYT
CO_322:0001034	CSSSev_E_1to9	null	Pedigree nurseries/trials	After anthesis	null	NA	CIMMYT
CO_322:0001035	CSSSev_E_pct	null	Pedigree nurseries/trials	After anthesis	null	NA	CIMMYT
CO_322:0001036	CLSSev_E_pct	null	Pedigree nurseries/trials	After anthesis	null	NA	CIMMYT
CO_322:0001037	CLSSev_E_1to9	null	Pedigree nurseries/trials	After anthesis	null	NA	CIMMYT
CO_322:0001038	DMSev_E_1to9	null	Pedigree nurseries/trials	After anthesis	null	NA	CIMMYT
CO_322:0001039	DMSev_E_pct	null	Pedigree nurseries/trials	After anthesis	null	NA	CIMMYT
CO_322:0001040	FusERSev_E_1to9	null	Pedigree nurseries/trials	Physiological maturity (R6 stage)	null	NA	CIMMYT
CO_322:0001041	FusERSev_E_pct	null	Pedigree nurseries/trials	Physiological maturity (R6 stage)	null	NA	CIMMYT
CO_322:0001042	FgramERSev_E_1to9	null	Pedigree nurseries/trials	Physiological maturity (R6 stage)	null	NA	CIMMYT
CO_322:0001043	FgramERSev_E_pct	null	Pedigree nurseries/trials	Physiological maturity (R6 stage)	null	NA	CIMMYT
CO_322:0001044	FmonERSev_E_1to9	null	Pedigree nurseries/trials	Physiological maturity (R6 stage)	null	NA	CIMMYT
CO_322:0001045	FmonERSev_E_pct	null	Pedigree nurseries/trials	Physiological maturity (R6 stage)	null	NA	CIMMYT
CO_322:0001046	FmonGRSev_E_pct	null	Pedigree nurseries/trials	null	null	NA	CIMMYT
CO_322:0001047	FmonGRSev_E_1to9	null	Pedigree nurseries/trials	null	null	NA	CIMMYT
CO_322:0001048	GLSSev_E_pct	null	Pedigree nurseries/trials	After anthesis	null	NA	CIMMYT
CO_322:0001049	GLSSev_E_1to9	null	Pedigree nurseries/trials	After anthesis	null	NA	CIMMYT
CO_322:0001050	HSmutSev_E_1to9	null	Pedigree nurseries/trials	After anthesis	null	NA	CIMMYT
CO_322:0001051	HSmutSev_E_pct	null	Pedigree nurseries/trials	After anthesis	null	NA	CIMMYT
CO_322:0001052	CSCMSev_E_1to9	null	Pedigree nurseries/trials	After anthesis	null	NA	CIMMYT
CO_322:0001053	CSCMSev_E_pct	null	Pedigree nurseries/trials	After anthesis	null	NA	CIMMYT
CO_322:0001056	DMVSev_Ct_plntPlot	null	Pedigree nurseries/trials	Physiological maturity (R6 stage)	null	NA	CIMMYT
CO_322:0001057	MLNSev_E_1to9	null	Pedigree nurseries/trials	null	null	NA	CIMMYT
CO_322:0001058	MLNSev_E_pct	null	Pedigree nurseries/trials	null	null	NA	CIMMYT
CO_322:0001059	MSVSev_E_1to9	null	Pedigree nurseries/trials	null	null	NA	CIMMYT
CO_322:0001060	MSVSev_E_pct	null	Pedigree nurseries/trials	null	null	NA	CIMMYT
CO_322:0001061	BmaySev_E_1to9	null	Pedigree nurseries/trials	After anthesis	null	NA	CIMMYT
CO_322:0001062	BmaySev_E_pct	null	Pedigree nurseries/trials	After anthesis	null	NA	CIMMYT
CO_322:0001063	PERSev_E_1to9	null	Pedigree nurseries/trials	At harvest	null	NA	CIMMYT
CO_322:0001064	PERSev_E_pct	null	Pedigree nurseries/trials	At harvest	null	NA	CIMMYT
CO_322:0001065	PLSSev_E_1to9	null	Pedigree nurseries/trials	3 weeks after silk emergence	null	NA	CIMMYT
CO_322:0001066	PLSSev_E_pct	null	Pedigree nurseries/trials	3 weeks after silk emergence	null	NA	CIMMYT
CO_322:0001067	PpolySev_E_1to9	null	Pedigree nurseries/trials	After anthesis	null	NA	CIMMYT
CO_322:0001068	PpolySev_E_pct	null	Pedigree nurseries/trials	After anthesis	null	NA	CIMMYT
CO_322:0001069	RustSev_E_1to9	null	Pedigree nurseries/trials	After anthesis	null	NA	CIMMYT
CO_322:0001070	RustSev_E_pct	null	Pedigree nurseries/trials	After anthesis	null	NA	CIMMYT
CO_322:0001071	DMSSev_E_1to9	null	Pedigree nurseries/trials	After anthesis	null	NA	CIMMYT
CO_322:0001072	DMSSev_E_pct	null	Pedigree nurseries/trials	After anthesis	null	NA	CIMMYT
CO_322:0001073	SmaySev_E_1to9	null	Pedigree nurseries/trials	At harvest	null	NA	CIMMYT
CO_322:0001074	SmaySev_E_pct	null	Pedigree nurseries/trials	At harvest	null	NA	CIMMYT
CO_322:0001075	TSCSev_E_1to9	null	Pedigree nurseries/trials	After anthesis	null	NA	CIMMYT
CO_322:0001076	TSCSev_E_pct	null	Pedigree nurseries/trials	After anthesis	null	NA	CIMMYT
CO_322:0001077	TRustSev_E_1to9	null	Pedigree nurseries/trials	After anthesis	null	NA	CIMMYT
CO_322:0001078	TRustSev_E_pct	null	Pedigree nurseries/trials	After anthesis	null	NA	CIMMYT
CO_322:0001079	EturcSev_E_1to9	null	Pedigree nurseries/trials	After anthesis	null	NA	CIMMYT
CO_322:0001080	EturcSev_E_pct	null	Pedigree nurseries/trials	After anthesis	null	NA	CIMMYT
CO_322:0001081	AntAnCol_E_1to9	null	Pedigree nurseries/trials	null	null	NA	CIMMYT

[illegible]

CO_327:0000148	DEm_Est_JDay	null	Trials and nurseries	Seedling stage	Recommended	NA	ICRISAT-Niger	C Tom Hash	16-Dec-12	EN	Pearl millet	CO_327:0000003	Date of emergence	Phenological traits	[illegible]	NA	DEm		Plant	Date of emergence	Recommended	NA	CO_327:0000004	Date of emergence - Method	Estimation	Record the emergence date
CO_327:0000149	DHd_Est_JDay	DSOHd	Trials and nurseries	Flowering stage	Recommended	NA	ICRISAT-Niger	C Tom Hash	16-Dec-12	EN	Pearl millet	CO_327:0000006	Date of heading	Phenological traits	[illegible]	NA	DHd		Plant	Date of heading	Recommended	NA	CO_327:0000007	Date of 50% heading - Method	Estimation	[illegible]
CO_327:0000150	DBl_Est_JDay	DSOBl	Trials and nurseries	Flowering stage	Recommended	NA	ICRISAT-Niger	C Tom Hash	16-Dec-12	EN	Pearl millet	CO_327:0000009	Date of bloom	Phenological traits	[illegible]	NA	DBl		Plant	Date of bloom	Recommended	NA	CO_327:0000010	Date of 50% bloom - Method	Estimation	[illegible]
CO_327:0000151	DAn_Est_JDay	DSOAn	Trials and nurseries	Flowering stage	Recommended	NA	ICRISAT-Niger	C Tom Hash	16-Dec-12	EN	Pearl millet	CO_327:0000012	Date of anthesis	Phenological traits	[illegible]	NA	DAn		Plant	Date of anthesis	Recommended	NA	CO_327:0000013	Date of 50% anthesis - Method	Estimation	[illegible]
CO_327:0000152	DPhysMat_Est_JDay	null	Trials and nurseries	Maturity	Recommended	NA	ICRISAT-Niger	C Tom Hash	16-Dec-12	EN	Pearl millet	CO_327:0000015	Date of physiological maturity	Phenological traits	[illegible]	NA	DPhysMat		Plant	Date of physiological maturity	Recommended	NA	CO_327:0000016	[illegible]	Estimation	[illegible]
CO_327:0000153	DHar_Est_JDay	null	Trials and nurseries	Maturity	null	NA	ICRISAT-Niger	C Tom Hash	16-Dec-12	EN	Pearl millet	CO_327:0000018	Date of harvest	Phenological traits	Date when plot harvested	NA	DHar		Plant	Date of harvest	null	NA	CO_327:0000019	Date of harvest - Method	Estimation	Record the harvest date
CO_327:0000154	NSH_Count_H	null	Trials and nurseries	Seedling stage	Obsolete	NA	ICRISAT-Niger	C Tom Hash	16-Dec-12	EN	Pearl millet	CO_327:0000021	Number of seedling hills	Agronomical traits	[illegible]	NA	NSH		Hill	Number	Obsolete	NA	CO_327:0000022	Number of seedling hills - Method	Counting	[illegible]
CO_327:0000155	NSPlt_CountThin_PltPlot	null	Trials and nurseries	Seedling stage	Obsolete	NA	ICRISAT-Niger	C Tom Hash	16-Dec-12	EN	Pearl millet	CO_327:0000024	Number of seedling plants	Agronomical traits	Number of total plants after thinning	NA	NSPlt		Seedling	Number	Obsolete	NA	CO_327:0000025	Number of seedlings - Method	Counting	[illegible]
CO_327:0000156	SVig_Est_1to5	null	Trials and nurseries	Seedling stage	Recommended	NA	ICRISAT-Niger	C Tom Hash	16-Dec-12	EN	Pearl millet	CO_327:0000027	Seedling vigor	Morphological traits	[illegible]	NA	SVig		Seedling	Biomass	Recommended	NA	CO_327:0000028	Seedling vigor - Method	Estimation	[illegible]
CO_327:0000157	VegVig_Est_1to5	null	Trials and nurseries	Vegetative growth Stage	Recommended	NA	ICRISAT-Niger	C Tom Hash	16-Dec-12	EN	Pearl millet	CO_327:0000030	Vegetative vigor	Morphological traits	[illegible]	NA	VegVig		Plant	Biomass	Recommended	NA	CO_327:0000031	Vegetative vigor - Method	Estimation	[illegible]
CO_327:0000158	PlNo_CountDMI_PltPlot	null	Trials and nurseries	Vegetative growth Stage	Obsolete	NA	ICRISAT-Niger	C Tom Hash	16-Dec-12	EN	Pearl millet	CO_327:0000033	Plant number per plot	Agronomical traits	[illegible]	NA	PlNo		Seedling	Number per plot	Obsolete	NA	CO_327:0000034	[illegible]	Counting	[illegible]
CO_327:0000159	HNo_Count_H	null	Trials and nurseries	Vegetative growth Stage	Obsolete	NA	ICRISAT-Niger	C Tom Hash	16-Dec-12	EN	Pearl millet	CO_327:0000036	Hill number per plot	Agronomical traits	[illegible]	NA	HNo		null	null	Obsolete	NA	CO_327:0000037	Hill number per plot - Method	Counting	[illegible]
CO_327:0000160	DMP_Count_Plnt	null	Trials and nurseries	Vegetative growth Stage	Recommended	NA	ICRISAT-Niger	C Tom Hash	16-Dec-12	EN	Pearl millet	CO_327:0000039	[illegible]	Biotic Stress traits	[illegible]	NA	DMP		Downy mildew infected plant	Number	Recommended	NA	CO_327:0000040	[illegible]	Counting	[illegible]
CO_327:0000161	DMH_Count_Plnt	null	Trials and nurseries	Vegetative growth Stage	Recommended	NA	ICRISAT-Niger	C Tom Hash	16-Dec-12	EN	Pearl millet	CO_327:0000042	Downy mildew infected hill number	Biotic Stress traits	[illegible]	NA	DMH		Downy mildew infected pockets	Number	Recommended	NA	CO_327:0000043	[illegible]	Counting	[illegible]
CO_327:0000162	BlaRS_Est_1to9	null	Trials and nurseries	Grain-filling stage	Recommended	NA	ICRISAT-Niger	C Tom Hash	16-Dec-12	EN	Pearl millet	CO_327:0000045	Plant reaction to blast	Biotic Stress traits	[illegible]	NA	BlaRS		Leaf	Reaction to blast	Recommended	NA	CO_327:0000046	Blast reaction score - Method	Estimation	[illegible]
CO_327:0000163	ErgProp_Comp_pct	Erg%	Trials and nurseries	Grain-filling stage	Recommended	NA	ICRISAT-Niger	C Tom Hash	16-Dec-12	EN	Pearl millet	CO_327:0000048	Ergot infection proportion	Biotic Stress traits	[illegible]	NA	ErgProp		Egrot sclerotia infected grain	Proportion	Recommended	NA	CO_327:0000049	Ergot infection percentage - Method	Computation	[illegible]
CO_327:0000164	RusRS_Est_1to9	null	Trials and nurseries	Grain-filling stage	Recommended	NA	ICRISAT-Niger	C Tom Hash	16-Dec-12	EN	Pearl millet	CO_327:0000051	Plant reaction to rust	Biotic Stress traits	[illegible]	NA	RusRS		Leaf	Reaction to rust	Recommended	NA	CO_327:0000052	Rust reaction score - Method	Estimation	[illegible]
CO_327:0000165	SmuProp_Comp_pct	Smu%	Trials and nurseries	Grain-filling stage	Recommended	NA	ICRISAT-Niger	C Tom Hash	16-Dec-12	EN	Pearl millet	CO_327:0000054	Smut infection proportion	Biotic Stress traits	[illegible]	NA	SmuProp		Smut sori infected grain	Proportion	Recommended	NA	CO_327:0000055	Smut infection percentage - Method	Computation	[illegible]
CO_327:0000166	PlHt_Av_cm	null	Trials and nurseries	Grain-filling stage	Recommended	NA	ICRISAT-Niger	C Tom Hash	16-Dec-12	EN	Pearl millet	CO_327:0000057	Plant height	Morphological traits	[illegible]	NA	PlHt		Plant	Height	Recommended	NA	CO_327:0000058	Plant height - Method	Computation	[illegible]
CO_327:0000167	PanLen_Av_cm	null	Trials and nurseries	Grain-filling stage	Recommended	NA	ICRISAT-Niger	C Tom Hash	16-Dec-12	EN	Pearl millet	CO_327:0000060	Panicle length	Morphological traits	[illegible]	NA	PanLen		Panicle	Length	Recommended	NA	CO_327:0000061	Panicle length - Method	Computation	[illegible]
CO_327:0000168	PanDia_Av_mm	null	Trials and nurseries	Grain-filling stage	Recommended	NA	ICRISAT-Niger	C Tom Hash	16-Dec-12	EN	Pearl millet	CO_327:0000063	Panicle diameter	Morphological traits	Diameter of the panicle	NA	PanDia		Panicle	Diameter	Recommended	NA	CO_327:0000064	Panicle diameter - Method	Computation	[illegible]
CO_327:0000169	PanGir_Av_mm	null	Trials and nurseries	Grain-filling stage	Recommended	NA	ICRISAT-Niger	C Tom Hash	16-Dec-12	EN	Pearl millet	CO_327:0000066	Panicle girth	Morphological traits	Girth of the panicle	NA	PanGir		Panicle	Girth	Recommended	NA	CO_327:0000067	Panicle girth - Method	Computation	[illegible]
CO_327:0000170	PanEx_Av_cm	null	Trials and nurseries	Grain-filling stage	Recommended	NA	ICRISAT-Niger	C Tom Hash	16-Dec-12	EN	Pearl millet	CO_327:0000069	Panicle exertion	Morphological traits	[illegible]	NA	PanEx		Panicle	Exertion	Recommended	NA	CO_327:0000070	Panicle exertion - Method	Computation	[illegible]
CO_327:0000171	NHarPl_Count_Plnt	null	Trials and nurseries	Maturity	Obsolete	NA	ICRISAT-Niger	C Tom Hash	16-Dec-12	EN	Pearl millet	CO_327:0000072	Number of harvested plants	Agronomical traits	[illegible]	NA	NHarPl		null	null	Obsolete	NA	CO_327:0000073	[illegible]	Counting	[illegible]
CO_327:0000172	NHarH_Count_H	null	Trials and nurseries	Maturity	Obsolete	NA	ICRISAT-Niger	C Tom Hash	16-Dec-12	EN	Pearl millet	CO_327:0000075	Number of harvested hills	Agronomical traits	[illegible]	NA	NHarH		null	null	Obsolete	NA	CO_327:0000076	Number of harvested hills - Method	Counting	[illegible]
CO_327:0000173	NEmPan_Count_Pan	null	Trials and nurseries	Maturity	Recommended	NA	ICRISAT-Niger	C Tom Hash	16-Dec-12	EN	Pearl millet	CO_327:0000078	[illegible]	Agronomical traits	[illegible]	NA	NEmPan		Empty panicle	Number	Recommended	NA	CO_327:0000079	[illegible]	Counting	[illegible]
CO_327:0000174	NHarPan_Count_Pan	null	Trials and nurseries	Maturity	null	NA	ICRISAT-Niger	C Tom Hash	16-Dec-12	EN	Pearl millet	CO_327:0000081	Number of harvested panicles	Agronomical traits	[illegible]	NA	NHarPan		Harvested panicle	Number	null	NA	CO_327:0000082	[illegible]	Counting	[illegible]
CO_327:0000175	NOTPan_Count_Pan	null	Trials and nurseries	Maturity	Recommended	NA	ICRISAT-Niger	C Tom Hash	16-Dec-12	EN	Pearl millet	CO_327:0000084	Number of off-type panicles	Agronomical traits	[illegible]	NA	NOTPan		Harvested off-type panicle	Number	Recommended	NA	CO_327:0000085	[illegible]	Counting	[illegible]
CO_327:0000176	AS_Appr_1to5	null	[illegible]	Maturity	Obsolete	NA	ICRISAT-Niger	C Tom Hash	16-Dec-12	EN	Pearl millet	CO_327:0000087	Agronomic appreciation score	Agronomical traits	Plant type desirability	NA	AS		Plant	Appreciation	Obsolete	NA	CO_327:0000088	[illegible]	Estimation	[illegible]
CO_327:0000177	FAS_FarmAppr_1to5	null	[illegible]	Maturity	Obsolete	NA	ICRISAT-Niger	C Tom Hash	16-Dec-12	EN	Pearl millet	CO_327:0000090	Farmer appreciation score	Agronomical traits	[illegible]	NA	FAS		Plant	Farmer appreciation	Obsolete	NA	CO_327:0000091	Farmer appreciation score - Method	Estimation	[illegible]
CO_327:0000178	FPS_FarmPref_1to3	null	Trials and nurseries	Maturity	Obsolete	NA	ICRISAT-Niger	C Tom Hash	16-Dec-12	EN	Pearl millet	CO_327:0000093	Farmer preference score	Agronomical traits	Farmer choice of the entry	NA	FPS		Plant	Farmer preference	Obsolete	NA	CO_327:0000094	Farmer preference score - Method	Estimation	[illegible]
CO_327:0000179	PanComp_Est_1to7	null	Trials and nurseries	Maturity	Recommended	NA	INERA-Burkina Faso	Drabo Inoussa	4-Mar-14	EN	Pearl millet	CO_327:0000096	Panicle compactness	Morphological traits	Compactness of the panicle	NA	PanComp		Panicle	Compactness	Recommended	NA	CO_327:0000097	Panicle compactness - Method	Estimation	[illegible]
CO_327:0000180	BriPre_Est_0or1	null	Trials and nurseries	Maturity	null	NA	INERA-Burkina Faso	Drabo Inoussa	4-Mar-14	EN	Pearl millet	CO_327:0000099	Bristle presence	Morphological traits	[illegible]	NA	BriPre		Bristle	Presence	null	NA	CO_327:0000100	Bristle presence - Method	Estimation	[illegible]
CO_327:0000181	BriLen_Av_cm	null	nurseries	Maturity	null	NA	INERA-Burkina Faso	Drabo Inoussa	4-Mar-14	EN	Pearl millet	CO_327:0000102	Bristle length	Morphological traits	Length of the bristel	NA	BriLen		Bristle	Length	null	NA	CO_327:0000103	Bristle length - Method	Computation	[illegible]
CO_327:0000182	BriDen_Est_1to5	null	Trials and nurseries	Maturity	null	NA	INERA-Burkina Faso	Drabo Inoussa	4-Mar-14	EN	Pearl millet	CO_327:0000105	Bristle density	Morphological traits	[illegible]	NA	BriDen		Bristle	Density	null	NA	CO_327:0000106	Bristle density - Method	Estimation	[illegible]
CO_327:0000183	PolFer_Comp_pct	null	Trials and nurseries	Flowering stage	null	NA	INERA-Burkina Faso	Drabo Inoussa	4-Mar-14	EN	Pearl millet	CO_327:0000108	Pollen fertility	Fertility traits	Proportion of fertile pollen	NA	PolFer		Fertile pollen	Proportion	null	NA	CO_327:0000109	Pollen fertility - Method	Computation	[illegible]
CO_327:0000184	SDSet_Comp_pct	null	Trials and nurseries	Maturity	Recommended	NA	INERA-Burkina Faso	Drabo Inoussa	4-Mar-14	EN	Pearl millet	CO_327:0000111	Seed set	Fertility traits	[illegible]	NA	SDSet		Seed	Set	Recommended	NA	CO_327:0000112	Seed set - Method	Computation	[illegible]
CO_327:0000185	StrTol_Count_StrigaPlot	null	Trials and nurseries	Vegetative growth Stage	null	NA	INERA-Burkina Faso	Drabo Inoussa	4-Mar-14	EN	Pearl millet	CO_327:0000114	Striga tolerance	Biotic Stress traits	Number of striga plant in the field	NA	StrTol		Striga	Population density	null	NA	CO_327:0000115	Striga tolerance - Method	Counting	Number of striger counted per plot
CO_327:0000186	HMD_Comp_pct	null	Trials and nurseries	Maturity	null	NA	INERA-Burkina Faso	Drabo Inoussa	4-Mar-14	EN	Pearl millet	CO_327:0000117	Head miner damage	Biotic Stress traits	[illegible]	NA	HMD		[illegible]	Proportion	null	NA	CO_327:0000118	Head miner damage - Method	Computation	[illegible]
CO_327:0000187	StBD	null	Trials and nurseries	Vegetative growth Stage	null	NA	INERA-Burkina Faso	Drabo Inoussa	4-Mar-14	EN	Pearl millet	CO_327:0000120	Stem borer damage	Biotic Stress traits	[illegible]	NA	StBD		null	null	null	NA	CO_327:0000121	Stem borer damage - Method	null	null
CO_327:0000188	FLLen_Av_	null	Trials and nurseries	head emerging	Recommended	NA	INERA-Burkina Faso	Drabo Inoussa	4-Mar-14	EN	Pearl millet	CO_327:0000123	Flag leaf length	Morphological traits	Flag leaf length	NA	FLLen		Flag leaf	Length	Recommended	NA	CO_327:0000124	Flag leaf length - Method	Computation	[illegible]
CO_327:0000189	FLDia_Av_	null	Trials and nurseries	head emerging	Recommended	NA	INERA-Burkina Faso	Drabo Inoussa	4-Mar-14	EN	Pearl millet	CO_327:0000126	Flag leaf dimeter	Morphological traits	Flag leaf diameter	NA	FLDia		Flag leaf	Diameter	Recommended	NA	CO_327:0000127	Flag leaf dimeter - Method	Computation	[illegible]
CO_327:0000190	StDia_Av_	null	Trials and nurseries	Maturity	null	NA	INERA-Burkina Faso	Drabo Inoussa	4-Mar-14	EN	Pearl millet	CO_327:0000129	Stem diameter	Morphological traits	Stem diameter	NA	StDia		Stem	Diameter	null	NA	CO_327:0000130	Stem diameter - Method	Computation	[illegible]
CO_327:0000191	SDCol_Est_	null	Trials and nurseries	Maturity	null	NA	INERA-Burkina Faso	Drabo Inoussa	4-Mar-14	EN	Pearl millet	CO_327:0000132	Seed color	Morphological traits	The color of the seed	NA	SDCol		Seed	Color	null	NA	CO_327:0000133	Seed color - Method	Estimation	Visually estimate the seed color
CO_327:0000192	BTN_Count_Till	null	Trials and nurseries	Maturity	Recommended	NA	INERA-Burkina Faso	Drabo Inoussa	4-Mar-14	EN	Pearl millet	CO_327:0000135	Basal tiller number	Agronomical traits	[illegible]	NA	BTN		Basal tiller	Number	Recommended	NA	CO_327:0000136	Basal tiller number - Method	Counting	Count the number of basal tillers
CO_327:0000193	NTN_Count_Till	null	Trials and nurseries	Maturity	null	NA	INERA-Burkina Faso	Drabo Inoussa	4-Mar-14	EN	Pearl millet	CO_327:0000138	Nodal tiller number	Agronomical traits	[illegible]	NA	NTN		Nodal tiller	Number	null	NA	CO_327:0000139	Nodal tiller number - Method	Counting	Count the number of nodal tillers
CO_327:0000194	GW_Meas_g	null	Trials and nurseries	Maturity	Recommended	NA	INERA-Burkina Faso	Drabo Inoussa	4-Mar-14	EN	Pearl millet	CO_327:0000141	Grain weight	Agronomical traits	[illegible]	NA	GW		Grain	Weight	Recommended	NA	CO_327:0000142	Grain weight - Method	Measurement	[illegible]
CO_327:0000195	HGW_Meas_g100gr	null	null	Maturity	Recommended	NA	INERA-Burkina Faso	Drabo Inoussa	4-Mar-14	EN	Pearl millet	CO_327:0000144	100 grain weight	Agronomical traits	weight of 100 grains	NA	HGW		100 grains	Weight	Recommended	NA	CO_327:0000145	100 grain weight - Method	Measurement	[illegible]
CO_327:0000197	PlntPerf_Appr_1to5	AS_Est_1to5	[illegible]	Maturity	Recommended	NA	ICRISAT-Niger	C Tom Hash	16-Dec-12	EN	Pearl millet	CO_327:0000196	Overall plant performance	Agronomical traits	Overall plant performance	NA	PlntPerf		Plant	Overall performance	Recommended	NA	CO_327:0000088	[illegible]	Estimation	[illegible]
CO_327:0000198	PlntPerf_FarmAppr_1to5	FAS_Est_1to5	[illegible]	Maturity	Recommended	NA	ICRISAT-Niger	C Tom Hash	16-Dec-12	EN	Pearl millet	CO_327:0000196	Overall plant performance	Agronomical traits	Overall plant performance	NA	PlntPerf		Plant	Overall performance	Recommended	NA	CO_327:0000091	Farmer appreciation score - Method	Estimation	[illegible]
CO_327:0000199	PlntPerf_FarmPref_1to3	FPS_Est_1to3	Trials and nurseries	Maturity	Recommended	NA	ICRISAT-Niger	C Tom Hash	16-Dec-12	EN	Pearl millet	CO_327:0000196	Overall plant performance	Agronomical traits	Overall plant performance	NA	PlntPerf		Plant	Overall performance	Recommended	NA	CO_327:0000094	Farmer preference score - Method	Estimation	[illegible]
CO_337:0000500	B_E_1to5	B_1_5, B	Characterization	Harvest	null	BMS_22447	ICRISAT/ISRA/DARS	NA	null	EN	Groundnut	CO_337:0000030	Pod beak	Morphological traits	Tip of the pod.	null	B	null	Pod	Beak	null	NA	CO_337:0000110	Pod beak - Method	Estimation	Pod beak assessment.
[illegible]																										

CO_333:1000324	PerispVol	Perisperm Volume	NA	NA	NA	GEVES/INRA	Daphné Verdelet	26/09/2017	EN	Sugar_beet	CO_333:0000026	Seed	Morphological	null	NA	null	null	Seed	null	NA	NA	CO_333:2000303	[illegible]	Measurement	[illegible]	null
CO_333:1000325	SeedCoatVol	Seed Coat Volume	NA	NA	NA	GEVES/INRA	Daphné Verdelet	26/09/2017	EN	Sugar_beet	CO_333:0000026	Seed	Morphological	null	NA	null	null	Seed	null	NA	NA	CO_333:2000306	Measurement of seed coat volume	Measurement	[illegible]	null
CO_333:1000329	SeedVol	Seed Volume	NA	NA	NA	GEVES/INRA	Daphné Verdelet	26/09/2017	EN	Sugar_beet	CO_333:0000026	Seed	Morphological	null	NA	null	null	Seed	null	NA	NA	CO_333:2000310	Measurement of seed volume	Measurement	[illegible]	null
CO_333:1000333	BolStart	Bolting Start	NA	NA	NA	International Data Base for Beta	Daphné Verdelet	26/09/2017	EN	Sugar_beet	CO_333:0000021	Seed Bearer	Morphological	null	NA	null	null	Seed Bearer	null	NA	NA	CO_333:2000315	Observation of Bolting Start	Estimation	[illegible]	null
CO_333:1000334	BractDistrib	Bract Distribution	NA	NA	NA	International Data Base for Beta	Daphné Verdelet	26/09/2017	EN	Sugar_beet	CO_333:0000021	Seed Bearer	Morphological	null	NA	null	null	Seed Bearer	null	NA	NA	CO_333:2000316	Observation of Bract Distribution	Estimation	[illegible]	null
CO_333:1000335	FlorweringTime	Flowering Time	NA	NA	NA	International Data Base for Beta	Daphné Verdelet	26/09/2017	EN	Sugar_beet	CO_333:0000021	Seed Bearer	Morphological	null	NA	null	null	Seed Bearer	null	NA	NA	CO_333:2000317	Observation of flowering time	Estimation	Stages of seedbearer flowering	null
CO_333:1000336	FloweringEnd	Flowering End	NA	NA	NA	International Data Base for Beta	Daphné Verdelet	26/09/2017	EN	Sugar_beet	CO_333:0000021	Seed Bearer	Morphological	null	NA	null	null	Seed Bearer	null	NA	NA	CO_333:2000318	Observation of the Flowering End	Estimation	Date of the seedbearer flowering end	null
CO_333:1000337	FLoweringStart	Flowering Start	NA	NA	NA	International Data Base for Beta	Daphné Verdelet	26/09/2017	EN	Sugar_beet	CO_333:0000021	Seed Bearer	Morphological	null	NA	null	null	Seed Bearer	null	NA	NA	CO_333:2000319	Observation of the Flowering Start	Estimation	[illegible]	null
CO_333:1000338	FLWRPATB	Flowering Pattern ? Between	NA	NA	NA	GRIN	Daphné Verdelet	26/09/2017	EN	Sugar_beet	CO_333:0000021	Seed Bearer	Morphological	null	NA	null	null	Seed Bearer	null	NA	NA	CO_333:2000320	[illegible]	Estimation	null	null
CO_333:1000339	FLWRPATW	Flowering Pattern ? Within	NA	NA	NA	GRIN	Daphné Verdelet	26/09/2017	EN	Sugar_beet	CO_333:0000021	Seed Bearer	Morphological	null	NA	null	null	Seed Bearer	null	NA	NA	CO_333:2000321	[illegible]	Estimation	null	null
CO_333:1000340	SeedMaturity	Seed Maturity	NA	NA	NA	International Data Base for Beta	Daphné Verdelet	26/09/2017	EN	Sugar_beet	CO_333:0000021	Seed Bearer	Morphological	null	NA	null	null	Seed Bearer	null	NA	NA	CO_333:2000322	Observation of seed maturity	Estimation	null	null
CO_333:1000341	SeedShattering	Seed Shattering	NA	NA	NA	International Data Base for Beta	Daphné Verdelet	26/09/2017	EN	Sugar_beet	CO_333:0000021	Seed Bearer	Morphological	null	NA	null	null	Seed Bearer	null	NA	NA	CO_333:2000323	Observation of seed shattering	Estimation	null	null
CO_333:1000342	StemColor	Stem Color	NA	NA	NA	International Data Base for Beta	Daphné Verdelet	26/09/2017	EN	Sugar_beet	CO_333:0000021	Seed Bearer	Morphological	null	NA	null	null	Seed Bearer	null	NA	NA	CO_333:2000324	Observation of Stem Color	Estimation	Color of the inflorescence stem	null
CO_333:1000343	DeltaTempHyp	Hypocotyl Temperature Delta	NA	NA	NA	GEVES/INRA	Daphné Verdelet	26/09/2017	EN	Sugar_beet	CO_333:0000027	Seedling	Morphological	null	NA	null	null	Seedling	null	NA	NA	CO_333:2000330	[illegible]	Computation	[illegible]	null
CO_333:1000344	DeltaTempRad	Radicle Temperature Delta	NA	NA	NA	GEVES/INRA	Daphné Verdelet	26/09/2017	EN	Sugar_beet	CO_333:0000027	Seedling	Morphological	null	NA	null	null	Seedling	null	NA	NA	CO_333:2000331	[illegible]	Computation	[illegible]	null
CO_333:1000345	Tb Seedling	Seedling Growth Base Temperature	NA	NA	NA	GEVES/INRA	Daphné Verdelet	26/09/2017	EN	Sugar_beet	CO_333:0000027	Seedling	Morphological	null	NA	null	null	Seedling	null	NA	NA	CO_333:2000335	[illegible]	Computation	[illegible]	null
CO_333:1000348	DeltaTempCot	Cotyledon Temperature Delta	NA	NA	NA	GEVES/INRA	Daphné Verdelet	26/09/2017	EN	Sugar_beet	CO_333:0000027	Seedling	Morphological	null	NA	null	null	Seedling	null	NA	NA	CO_333:2000329	[illegible]	Measurement	[illegible]	null
CO_333:1000349	HypSpd	[illegible]	NA	NA	NA	GEVES/INRA	Daphné Verdelet	26/09/2017	EN	Sugar_beet	CO_333:0000027	Seedling	Morphological	null	NA	null	null	Seedling	null	NA	NA	CO_333:2000332	[illegible]	Measurement	[illegible]	null
CO_333:1000351	RadSpd	[illegible]	NA	NA	NA	GEVES/INRA	Daphné Verdelet	26/09/2017	EN	Sugar_beet	CO_333:0000027	Seedling	Morphological	null	NA	null	null	Seedling	null	NA	NA	CO_333:2000334	[illegible]	Measurement	[illegible]	null
CO_333:1000352	SeedlingBcyanin	Seedling Betacyanin Content	NA	NA	NA	International Data Base for Beta	Daphné Verdelet	26/09/2017	EN	Sugar_beet	CO_333:0000027	Seedling	Morphological	null	NA	null	null	Seedling	null	NA	NA	CO_333:2000341	No method name found	null	null	null
CO_333:1000353	NICHLOROPPL	Nirs - Chlorophyll Quantity In Plot	NA	NA	NA	Institut Technique de la Betterave	Daphné Verdelet	26/09/2017	EN	Sugar_beet	CO_333:0000028	Photosynthesis	Physiological	null	NA	null	null	Leaf	null	NA	NA	CO_333:2000337	Quantity of chlorophyll in canopy	Estimation	[illegible]	null
CO_333:1000355	NINIPL	Nirs - Nitrogen Quantity In Plot	NA	NA	NA	Institut Technique de la Betterave	Daphné Verdelet	26/09/2017	EN	Sugar_beet	CO_333:0000029	Plant composition	Physiological	null	NA	null	null	Leaf	null	NA	NA	CO_333:2000339	Quantity of nitrogen in canopy	Estimation	[illegible]	null
CO_333:1000356	LANILE	Lab - Nitrogen Quantity In Leaf	NA	NA	NA	[illegible]	Daphné Verdelet	26/09/2017	EN	Sugar_beet	CO_333:0000029	Plant composition	Physiological	null	NA	null	null	Leaf	null	NA	NA	CO_333:2000338	Measurement of nitrogen in leaves	Measurement	[illegible]	null
CO_333:1000361	GERM	Germity	NA	NA	NA	Florimond Desprez	Daphné Verdelet	26/09/2017	EN	Sugar_beet	CO_333:0000030	Seed Quality	Quality	null	NA	null	null	Seed	null	NA	NA	CO_333:2000341	Percentage of monogerm seeds	Measurement	null	null
CO_333:1000365	KNa	K + Na	NA	NA	NA	Florimond Desprez	Daphné Verdelet	26/09/2017	EN	Sugar_beet	CO_333:0000031	Technological quality trait	Quality	null	NA	null	null	Root	null	NA	NA	CO_333:2000356	[illegible]	Computation	[illegible]	mM K + mM Na
CO_333:1000371	BETAN	Betaine NIR	NA	NA	NA	Florimond Desprez	Daphné Verdelet	26/09/2017	EN	Sugar_beet	CO_333:0000031	Technological quality trait	Quality	null	NA	null	null	Root	null	NA	NA	CO_333:2000354	Betain content predicted by FT-NIR	Estimation	[illegible]	null
CO_333:1000380	BETAH	Betaine HPLC	NA	NA	NA	Florimond Desprez	Daphné Verdelet	26/09/2017	EN	Sugar_beet	CO_333:0000031	Technological quality trait	Quality	null	NA	null	null	Root	null	NA	NA	CO_333:2000353	[illegible]	Measurement	[illegible]	null
CO_333:1000381	LABAN	[illegible]	NA	NA	NA	[illegible]	Daphné Verdelet	26/09/2017	EN	Sugar_beet	CO_333:0000031	Technological quality trait	Quality	null	NA	null	null	Root	null	NA	NA	CO_333:2000357	[illegible]	Measurement	[illegible]	null
CO_333:1000382	LABGLC	Lab - Glucose Content Of Beet Mush	NA	NA	NA	Institut Technique de la Betterave	Daphné Verdelet	26/09/2017	EN	Sugar_beet	CO_333:0000031	Technological quality trait	Quality	null	NA	null	null	Root	null	NA	NA	CO_333:2000359	[illegible]	Measurement	[illegible]	null
CO_333:1000383	LABK	[illegible]	NA	NA	NA	[illegible]	Daphné Verdelet	26/09/2017	EN	Sugar_beet	CO_333:0000031	Technological quality trait	Quality	null	NA	null	null	Root	null	NA	NA	CO_333:2000360	[illegible]	Measurement	[illegible]	null
CO_333:1000384	LABNA	[illegible]	NA	NA	NA	[illegible]	Daphné Verdelet	26/09/2017	EN	Sugar_beet	CO_333:0000031	Technological quality trait	Quality	null	NA	null	null	Root	null	NA	NA	CO_333:2000361	[illegible]	Measurement	[illegible]	null
CO_333:1000385	LABSC	Lab- Sugar Content Of Beets Mush	NA	NA	NA	Institut Technique de la Betterave	Daphné Verdelet	26/09/2017	EN	Sugar_beet	CO_333:0000031	Technological quality trait	Quality	null	NA	null	null	Root	null	NA	NA	CO_333:2000362	[illegible]	Measurement	[illegible]	null

[illegible]

CO_358:0000547	SCY (lb/acre)	SCY_M_lb_acre	trial evaluation	NA	standard for CottonGen	NA	CottonGen	Jing Yu	Jing Yu	20-May-19	English	cotton	CO_358:0000039	seed cotton yield	Agronomical traits	[illegible]	null	SCY	seed cotton	yield	standard for CottonGen	NA	CO_358:0000546	SCY - Measurement	Measurement	[illegible]	null
CO_358:0000549	leaf BCONC (mg/kg)	BCONC_Lf_M_mg_kg	QTL evaluation	NA	standard for CottonGen	NA	CottonGen	Jing Yu	Jing Yu	20-May-19	English	cotton	CO_358:0000006	calcium concentration	Biochemical traits	[illegible]	null	CACONC	plant	calcium concentration	standard for CottonGen	NA	CO_358:0000548	BCONC - Measurement	Measurement	[illegible]	null
CO_358:0000552	lint color (1-8)	LINTCL_E_1to8	CN germplasm evaluation	NA	standard for CottonGen	NA	CottonGen	Jing Yu	Jing Yu	20-May-19	English	cotton	CO_358:0000023	lint color	Morphological traits	Coloration of mature fiber	null	LINTCL	fiber	color	standard for CottonGen	NA	CO_358:0000550	LINTCL - Estimation	Estimation	[illegible]	null
CO_358:0000554	petiole BCONC (mg/kg)	BCONC_petiole_M_mg_kg	QTL evaluation	NA	standard for CottonGen	NA	CottonGen	Jing Yu	Jing Yu	20-May-19	English	cotton	CO_358:0000006	calcium concentration	Biochemical traits	[illegible]	null	CACONC	plant	calcium concentration	standard for CottonGen	NA	CO_358:0000553	BCONC - Measurement	Measurement	[illegible]	null
CO_358:0000556	root BCONC (mg/kg)	BCONC_Rt_M_mg_kg	QTL evaluation	NA	standard for CottonGen	NA	CottonGen	Jing Yu	Jing Yu	20-May-19	English	cotton	CO_358:0000006	calcium concentration	Biochemical traits	[illegible]	null	CACONC	plant	calcium concentration	standard for CottonGen	NA	CO_358:0000555	BCONC - Measurement	Measurement	[illegible]	null
CO_358:0000559	lint color (1-7,9)	LINTCL_E_1to7_9	NCGC germplasm evaluation	NA	standard for CottonGen	NA	CottonGen	Jing Yu	Jing Yu	20-May-19	English	cotton	CO_358:0000023	lint color	Morphological traits	Coloration of mature fiber	null	LINTCL	fiber	color	standard for CottonGen	NA	CO_358:0000557	LINTCL - Estimation	Estimation	[illegible]	null
CO_358:0000561	seed BCONC (mg/kg)	BCONC_Sd_M_mg_kg	QTL evaluation	NA	standard for CottonGen	NA	CottonGen	Jing Yu	Jing Yu	20-May-19	English	cotton	CO_358:0000006	calcium concentration	Biochemical traits	[illegible]	null	CACONC	plant	calcium concentration	standard for CottonGen	NA	CO_358:0000560	BCONC - Measurement	Measurement	[illegible]	null
CO_358:0000563	stem BCONC (mg/kg)	BCONC_stem_M_mg_kg	QTL evaluation	NA	standard for CottonGen	NA	CottonGen	Jing Yu	Jing Yu	20-May-19	English	cotton	CO_358:0000006	calcium concentration	Biochemical traits	[illegible]	null	CACONC	plant	calcium concentration	standard for CottonGen	NA	CO_358:0000562	BCONC - Measurement	Measurement	[illegible]	null
CO_358:0000566	lint color (1-5)	LINTCL_E_1to5	UZ germplasm evaluation	NA	standard for CottonGen	NA	CottonGen	Jing Yu	Jing Yu	20-May-19	English	cotton	CO_358:0000023	lint color	Morphological traits	Coloration of mature fiber	null	LINTCL	fiber	color	standard for CottonGen	NA	CO_358:0000564	LINTCL - Estimation	Estimation	[illegible]	null
CO_358:0000569	LY (g/boll)	LY_M_g_boll	QTL evaluation	NA	standard for CottonGen	NA	CottonGen	Jing Yu	Jing Yu	20-May-19	English	cotton	CO_358:0000025	lint yield	Agronomical traits	[illegible]	null	LY	fiber	yield	standard for CottonGen	NA	CO_358:0000567	LY - Measurement	Measurement	lint weight per boll in grams	null
CO_358:0000571	leaf CACONC (mg/kg)	CACONC_Lf_M_mg_kg	QTL evaluation	NA	standard for CottonGen	NA	CottonGen	Jing Yu	Jing Yu	20-May-19	English	cotton	CO_358:0000006	calcium concentration	Biochemical traits	[illegible]	null	CACONC	plant	calcium concentration	standard for CottonGen	NA	CO_358:0000570	CACONC - Measurement	Measurement	[illegible]	null
CO_358:0000573	petiole CACONC (mg/kg)	CACONC_petiole_M_mg_kg	QTL evaluation	NA	standard for CottonGen	NA	CottonGen	Jing Yu	Jing Yu	20-May-19	English	cotton	CO_358:0000006	calcium concentration	Biochemical traits	[illegible]	null	CACONC	plant	calcium concentration	standard for CottonGen	NA	CO_358:0000572	CACONC - Measurement	Measurement	[illegible]	null
CO_358:0000575	root CACONC (mg/kg)	CACONC_Rt_M_mg_kg	QTL evaluation	NA	standard for CottonGen	NA	CottonGen	Jing Yu	Jing Yu	20-May-19	English	cotton	CO_358:0000006	calcium concentration	Biochemical traits	[illegible]	null	CACONC	plant	calcium concentration	standard for CottonGen	NA	CO_358:0000574	CACONC - Measurement	Measurement	[illegible]	null
CO_358:0000577	seed CACONC (mg/kg)	CACONC_Sd_M_mg_kg	QTL evaluation	NA	standard for CottonGen	NA	CottonGen	Jing Yu	Jing Yu	20-May-19	English	cotton	CO_358:0000006	calcium concentration	Biochemical traits	[illegible]	null	CACONC	plant	calcium concentration	standard for CottonGen	NA	CO_358:0000576	CACONC - Measurement	Measurement	[illegible]	null
CO_358:0000579	stem CACONC (mg/kg)	CACONC_stem_M_mg_kg	QTL evaluation	NA	standard for CottonGen	NA	CottonGen	Jing Yu	Jing Yu	20-May-19	English	cotton	CO_358:0000006	calcium concentration	Biochemical traits	[illegible]	null	CACONC	plant	calcium concentration	standard for CottonGen	NA	CO_358:0000578	CACONC - Measurement	Measurement	[illegible]	null
CO_358:0000581	leaf CUCONC (mg/kg)	CUCONC_Lf_M_mg_kg	QTL evaluation	NA	standard for CottonGen	NA	CottonGen	Jing Yu	Jing Yu	20-May-19	English	cotton	CO_358:0000007	copper concentration	Biochemical traits	[illegible]	null	CUCONC	plant	copper concentration	standard for CottonGen	NA	CO_358:0000580	CUCONC - Measurement	Measurement	[illegible]	null
CO_358:0000583	petiole CUCONC (mg/kg)	CUCONC_petiole_M_mg_kg	QTL evaluation	NA	standard for CottonGen	NA	CottonGen	Jing Yu	Jing Yu	20-May-19	English	cotton	CO_358:0000007	copper concentration	Biochemical traits	[illegible]	null	CUCONC	plant	copper concentration	standard for CottonGen	NA	CO_358:0000582	CUCONC - Measurement	Measurement	[illegible]	null
CO_358:0000585	root CUCONC (mg/kg)	CUCONC_Rt_M_mg_kg	QTL evaluation	NA	standard for CottonGen	NA	CottonGen	Jing Yu	Jing Yu	20-May-19	English	cotton	CO_358:0000007	copper concentration	Biochemical traits	[illegible]	null	CUCONC	plant	copper concentration	standard for CottonGen	NA	CO_358:0000584	CUCONC - Measurement	Measurement	[illegible]	null
CO_358:0000587	seed CUCONC (mg/kg)	CUCONC_Sd_M_mg_kg	QTL evaluation	NA	standard for CottonGen	NA	CottonGen	Jing Yu	Jing Yu	20-May-19	English	cotton	CO_358:0000007	copper concentration	Biochemical traits	[illegible]	null	CUCONC	plant	copper concentration	standard for CottonGen	NA	CO_358:0000586	CUCONC - Measurement	Measurement	[illegible]	null
CO_358:0000589	stem CUCONC (mg/kg)	CUCONC_stem_M_mg_kg	QTL evaluation	NA	standard for CottonGen	NA	CottonGen	Jing Yu	Jing Yu	20-May-19	English	cotton	CO_358:0000007	copper concentration	Biochemical traits	[illegible]	null	CUCONC	plant	copper concentration	standard for CottonGen	NA	CO_358:0000588	CUCONC - Measurement	Measurement	[illegible]	null
CO_358:0000591	leaf FECONC (mg/kg)	FECONC_Lf_M_mg_kg	QTL evaluation	NA	standard for CottonGen	NA	CottonGen	Jing Yu	Jing Yu	20-May-19	English	cotton	CO_358:0000019	iron concentration	Biochemical traits	[illegible]	null	FECONC	plant	iron concentration	standard for CottonGen	NA	CO_358:0000590	FECONC - Measurement	Measurement	[illegible]	null
CO_358:0000593	petiole FECONC (mg/kg)	FECONC_petiole_M_mg_kg	QTL evaluation	NA	standard for CottonGen	NA	CottonGen	Jing Yu	Jing Yu	20-May-19	English	cotton	CO_358:0000019	iron concentration	Biochemical traits	[illegible]	null	FECONC	plant	iron concentration	standard for CottonGen	NA	CO_358:0000592	FECONC - Measurement	Measurement	[illegible]	null
CO_358:0000595	root FECONC (mg/kg)	FECONC_Rt_M_mg_kg	QTL evaluation	NA	standard for CottonGen	NA	CottonGen	Jing Yu	Jing Yu	20-May-19	English	cotton	CO_358:0000019	iron concentration	Biochemical traits	[illegible]	null	FECONC	plant	iron concentration	standard for CottonGen	NA	CO_358:0000594	FECONC - Measurement	Measurement	[illegible]	null
CO_358:0000597	seed FECONC (mg/kg)	FECONC_Sd_M_mg_kg	QTL evaluation	NA	standard for CottonGen	NA	CottonGen	Jing Yu	Jing Yu	20-May-19	English	cotton	CO_358:0000019	iron concentration	Biochemical traits	[illegible]	null	FECONC	plant	iron concentration	standard for CottonGen	NA	CO_358:0000596	FECONC - Measurement	Measurement	[illegible]	null
CO_358:0000599	stem FECONC (mg/kg)	FECONC_stem_M_mg_kg	QTL evaluation	NA	standard for CottonGen	NA	CottonGen	Jing Yu	Jing Yu	20-May-19	English	cotton	CO_358:0000019	iron concentration	Biochemical traits	[illegible]	null	FECONC	plant	iron concentration	standard for CottonGen	NA	CO_358:0000598	FECONC - Measurement	Measurement	[illegible]	null
CO_358:0000601	leaf KCONC (mg/kg)	KCONC_Lf_M_mg_kg	QTL evaluation	NA	standard for CottonGen	NA	CottonGen	Jing Yu	Jing Yu	20-May-19	English	cotton	CO_358:0000037	potassium concentration	Biochemical traits	[illegible]	null	KCONC	plant	potassium concentration	standard for CottonGen	NA	CO_358:0000600	KCONC - Measurement	Measurement	[illegible]	null
CO_358:0000603	petiole KCONC (mg/kg)	KCONC_petiole_M_mg_kg	QTL evaluation	NA	standard for CottonGen	NA	CottonGen	Jing Yu	Jing Yu	20-May-19	English	cotton	CO_358:0000037	potassium concentration	Biochemical traits	[illegible]	null	KCONC	plant	potassium concentration	standard for CottonGen	NA	CO_358:0000602	KCONC - Measurement	Measurement	[illegible]	null
CO_358:0000605	root KCONC (mg/kg)	KCONC_Rt_M_mg_kg	QTL evaluation	NA	standard for CottonGen	NA	CottonGen	Jing Yu	Jing Yu	20-May-19	English	cotton	CO_358:0000037	potassium concentration	Biochemical traits	[illegible]	null	KCONC	plant	potassium concentration	standard for CottonGen	NA	CO_358:0000604	KCONC - Measurement	Measurement	[illegible]	null
CO_358:0000607	seed KCONC (mg/kg)	KCONC_Sd_M_mg_kg	QTL evaluation	NA	standard for CottonGen	NA	CottonGen	Jing Yu	Jing Yu	20-May-19	English	cotton	CO_358:0000037	potassium concentration	Biochemical traits	[illegible]	null	KCONC	plant	potassium concentration	standard for CottonGen	NA	CO_358:0000606	KCONC - Measurement	Measurement	[illegible]	null
CO_358:0000609	stem KCONC (mg/kg)	KCONC_stem_M_mg_kg	QTL evaluation	NA	standard for CottonGen	NA	CottonGen	Jing Yu	Jing Yu	20-May-19	English	cotton	CO_358:0000037	potassium concentration	Biochemical traits	[illegible]	null	KCONC	plant	potassium concentration	standard for CottonGen	NA	CO_358:0000608	KCONC - Measurement	Measurement	[illegible]	null
CO_358:0000611	leaf MGCONC (mg/kg)	MGCONC_Lf_M_mg_kg	QTL evaluation	NA	standard for CottonGen	NA	CottonGen	Jing Yu	Jing Yu	20-May-19	English	cotton	CO_358:0000026	magnesium concentration	Biochemical traits	[illegible]	null	MGCONC	plant	magnesium concentration	standard for CottonGen	NA	CO_358:0000610	MGCONC - Measurement	Measurement	[illegible]	null
CO_358:0000613	petiole MGCONC (mg/kg)	MGCONC_petiole_M_mg_kg	QTL evaluation	NA	standard for CottonGen	NA	CottonGen	Jing Yu	Jing Yu	20-May-19	English	cotton	CO_358:0000026	magnesium concentration	Biochemical traits	[illegible]	null	MGCONC	plant	magnesium concentration	standard for CottonGen	NA	CO_358:0000612	MGCONC - Measurement	Measurement	[illegible]	null
CO_358:0000615	root MGCONC (mg/kg)	MGCONC_Rt_M_mg_kg	QTL evaluation	NA	standard for CottonGen	NA	CottonGen	Jing Yu	Jing Yu	20-May-19	English	cotton	CO_358:0000026	magnesium concentration	Biochemical traits	[illegible]	null	MGCONC	plant	magnesium concentration	standard for CottonGen	NA	CO_358:0000614	MGCONC - Measurement	Measurement	[illegible]	null
CO_358:0000617	seed MGCONC (mg/kg)	MGCONC_Sd_M_mg_kg	QTL evaluation	NA	standard for CottonGen	NA	CottonGen	Jing Yu	Jing Yu	20-May-19	English	cotton	CO_358:0000026	magnesium concentration	Biochemical traits	[illegible]	null	MGCONC	plant	magnesium concentration	standard for CottonGen	NA	CO_358:0000616	MGCONC - Measurement	Measurement	[illegible]	null
CO_358:0000619	stem MGCONC (mg/kg)	MGCONC_stem_M_mg_kg	QTL evaluation	NA	standard for CottonGen	NA	CottonGen	Jing Yu	Jing Yu	20-May-19	English	cotton	CO_358:0000026	magnesium concentration	Biochemical traits	[illegible]	null	MGCONC	plant	magnesium concentration	standard for CottonGen	NA	CO_358:0000618	MGCONC - Measurement	Measurement	[illegible]	null
CO_358:0000621	leaf MNCONC (mg/kg)	MNCONC_Lf_M_mg_kg	QTL evaluation	NA	standard for CottonGen	NA	CottonGen	Jing Yu	Jing Yu	20-May-19	English	cotton	CO_358:0000027	manganese concentration	Biochemical traits	[illegible]	null	MNCONC	plant	manganese concentration	standard for CottonGen	NA	CO_358:0000620	MNCONC - Measurement	Measurement	[illegible]	null
CO_358:0000623	petiole MNCONC (mg/kg)	MNCONC_petiole_M_mg_kg	QTL evaluation	NA	standard for CottonGen	NA	CottonGen	Jing Yu	Jing Yu	20-May-19	English	cotton	CO_358:0000027	manganese concentration	Biochemical traits	[illegible]	null	MNCONC	plant	manganese concentration	standard for CottonGen	NA	CO_358:0000622	MNCONC - Measurement	Measurement	[illegible]	null
CO_358:0000625	root MNCONC (mg/kg)	MNCONC_Rt_M_mg_kg	QTL evaluation	NA	standard for CottonGen	NA	CottonGen	Jing Yu	Jing Yu	20-May-19	English	cotton	CO_358:0000027	manganese concentration	Biochemical traits	[illegible]	null	MNCONC	plant	manganese concentration	standard for CottonGen	NA	CO_358:0000624	MNCONC - Measurement	Measurement	[illegible]	null
CO_358:0000627	seed MNCONC (mg/kg)	MNCONC_Sd_M_mg_kg	QTL evaluation	NA	standard for CottonGen	NA	CottonGen	Jing Yu	Jing Yu	20-May-19	English	cotton	CO_358:0000027	manganese concentration	Biochemical traits	[illegible]	null	MNCONC	plant	manganese concentration	standard for CottonGen	NA	CO_358:0000626	MNCONC - Measurement	Measurement	[illegible]	null
CO_358:0000629	stem MNCONC (mg/kg)	MNCONC_stem_M_mg_kg	QTL evaluation	NA	standard for CottonGen	NA	CottonGen	Jing Yu	Jing Yu	20-May-19	English	cotton	CO_358:0000027	manganese concentration	Biochemical traits	[illegible]	null	MNCONC	plant	manganese concentration	standard for CottonGen	NA	CO_358:0000628	MNCONC - Measurement	Measurement	[illegible]	null

[illegible]

Method.reference	Scale.ID	Scale.name	Scale.class	Decimal.places	Lower.limit	Upper.limit	Scale.Xref	Category.1	Category.2
[illegible]	CO_336:0000002	day	Duration					NA	NA
[illegible]	CO_336:0000005	1-5 infection score	Ordinal					1=0% lesions on plants	[illegible]
[illegible]	CO_336:0000008	1-5 infection score	Ordinal					1=0% lesions on plants	[illegible]
[illegible]	CO_336:0000011	1-5 infection score	Ordinal					1=0% virus symptom seen	[illegible]
[illegible]	CO_336:0000014	1-5 infection score	Ordinal					1=0% leaf spot seen	[illegible]
[illegible]	CO_336:0000017	1 to 5 percentage scale	Ordinal					1=0% nodules	[illegible]
[illegible]	CO_336:0000020	1-5 infection score	Ordinal					1=0% root knot galling	[illegible]
[illegible]	CO_336:0000023	day	Numerical					NA	NA
[illegible]	CO_336:0000026	cm	Numerical					NA	NA
[illegible]	CO_336:0000029	cm	Numerical					NA	NA
[illegible]	CO_336:0000032	1 to 5 percentage scale	Ordinal					1= Almost all plants erect	[illegible]
[illegible]	CO_336:0000035	Plant	Numerical					NA	NA
[illegible]	CO_336:0000038	g/plot	Numerical					NA	NA
[illegible]	CO_336:0000041	g/plot	Numerical					NA	NA
[illegible]	CO_336:0000044	1 to 5 percentage scale	Ordinal					1=0% pods shattered	[illegible]
[illegible]	CO_336:0000044	1 to 5 percentage scale	Ordinal					1=0% pods shattered	[illegible]
[illegible]	CO_336:0000047	Percentage	Numerical		0	100		NA	NA
[illegible]	CO_336:0000050	Percentage	Numerical		0	100		NA	NA
[illegible]	CO_336:0000053	Percentage	Numerical		0	100		NA	NA
[illegible]	CO_336:0000056	Undefined scale	Undefined		0	100		NA	NA
[illegible]	CO_336:0000059	1 to 5 branching scale	Ordinal					1=rarely branching	[illegible]
[illegible]	CO_336:0000062	day	Duration					NA	NA
[illegible]	CO_336:0000065	1 to 9 color scale	Nominal					1=B=blue	[illegible]
[illegible]	CO_336:0000068	mm	Numerical					NA	NA
[illegible]	CO_336:0000071	mmdd	Date		0	100		NA	NA
[illegible]	CO_336:0000074	Undefined scale	Undefined		0	100		NA	NA
[illegible]	CO_336:0000077	SPAD unit	Numerical					NA	NA
[illegible]	CO_336:0000080	Undefined scale	Undefined		0	100		NA	NA
[illegible]	CO_336:0000083	Undefined scale	Undefined		0	100		NA	NA
[illegible]	CO_336:0000086	Ratio	Numerical		0	100		NA	NA
[illegible]	CO_336:0000089	day	Duration					NA	NA
[illegible]	CO_336:0000092	1 to 4 color scale	Ordinal					1=Bl=Black	4=G=Grey
[illegible]	CO_336:0000095	1 to 12 color scale	Ordinal					1=Bf= Buff	4=Br= Brown
[illegible]	CO_336:0000098	1 to 5 quality scale	Ordinal					1=Good	4=bad
[illegible]	CO_336:0000101	Undefined scale	Undefined		0	100		NA	NA
[illegible]	CO_336:0000104	Undefined scale	Undefined		0	100		NA	NA
[illegible]	CO_336:0000107	Percentage	Numerical		0	100		NA	NA
[illegible]	CO_336:0000110	Percentage	Numerical		0	100		NA	NA
[illegible]	CO_336:0000113	Percentage	Numerical		0	100		NA	NA
[illegible]	CO_336:0000116	Percentage	Numerical		0	100		NA	NA
[illegible]	CO_336:0000119	Percentage	Numerical		0	100		NA	NA
[illegible]	CO_336:0000122	Percentage	Numerical		0	100		NA	NA
[illegible]	CO_336:0000125	1 or 2 type scale	Nominal					2=indeterminate termination	[illegible]
[illegible]	CO_336:0000128	1 to 5 percentage scale	Ordinal					1=0-20% defoliated	[illegible]
[illegible]	CO_336:0000131	1 to 5 percentage scale	Ordinal					1=0-20% defoliated	[illegible]
[illegible]	CO_336:0000134	1 to 5 percentage scale	Ordinal					1=no pods shattered	[illegible]
[illegible]	CO_336:0000134	1 to 5 percentage scale	Ordinal					1=no pods shattered	[illegible]
[illegible]	CO_336:0000137	1 to 4 color scale	Nominal					1=T=Tawny	[illegible]
[illegible]	CO_336:0000143	1 to 13 maturity scale	Nominal					0=maturity group 0 (extreme North)	[illegible]
[illegible]	CO_336:0000146	1 to 10 percentage scale	Ordinal					0=less than 10% seed discolored	[illegible]
[illegible]	CO_336:0000149	Percentage	Numerical		0	100		NA	NA
[illegible]	[illegible]	[illegible]	[illegible]	[illegible]	[illegible]	[illegible]	[illegible]	[illegible]	[illegible]

Dixon et al 2010 Fukuda et al 2010	CO_334:0100434	7 pt scale	Ordinal			1 =
Dixon et al 2010 Fukuda et al 2010	CO_334:0100490	Plant	Numerical			null
Dixon et al 2010 Fukuda et al 2010	CO_334:0100491	Root	Numerical			null
Dixon et al 2010 Fukuda et al 2010	CO_334:0100492	kg	Numerical	0	100	null
Dixon et al 2010 Fukuda et al 2010	CO_334:0100535	t/ha	Numerical	0	100	null
Dixon et al 2010 Fukuda et al 2010	CO_334:0100536	t/ha	Numerical	0	50	null
Dixon et al 2010 Fukuda et al 2010	CO_334:0100493	kg	Numerical	0	100	null
Dixon et al 2010 Fukuda et al 2010	CO_334:0100538	t/ha	Numerical	0	100	null
Dixon et al 2010 Fukuda et al 2010	CO_334:0100509	cm	Numerical	0	500	null
Dixon et al 2010 Fukuda et al 2010	CO_334:0100494	7pt scale	Ordinal			1 =
Dixon et al 2010 Fukuda et al 2010	CO_334:0100495	6pt scale	Nominal			1 = Conical
Dixon et al 2010 Fukuda et al 2010	CO_334:0100498	3pt scale	Nominal			1 = White or cream
Dixon et al 2010 Fukuda et al 2010	CO_334:0100499	8pt scale	Ordinal			0 = absent or sessile
Dixon et al 2010 Fukuda et al 2010	CO_334:0100551	7pt scale	Nominal			1 =
Dixon et al 2010 Fukuda et al 2010	CO_334:0100550	4 pt scale	Ordinal			0 = Absent
Dixon et al 2010 Fukuda et al 2010	CO_334:0100554	8pt scale	Nominal			0 = Absent
Dixon et al 2010 Fukuda et al 2010	CO_334:0100544	8pt scale	Nominal			0 = Absence
Dixon et al 2010 Fukuda et al 2010	CO_334:0100473	5 pt scale	Ordinal			1 = No symptom
Dixon et al 2010 Fukuda et al 2010	CO_334:0100474	ratio	Numerical	0	1	null
Dixon et al 2010 Fukuda et al 2010	CO_334:0100531	5 pt scale	Ordinal			1 = No symptom
Dixon et al 2010 Fukuda et al 2010	CO_334:0100532	ratio	Numerical	0	1	null
Dixon et al 2010 Fukuda et al 2010	CO_334:0100529	5 pt scale	Ordinal			1 = No symptom
Dixon et al 2010 Fukuda et al 2010	CO_334:0100530	ratio	Numerical	0	1	null
Dixon et al 2010 Fukuda et al 2010	CO_334:0100549	7pt scale	Ordinal			0 = no petiole
Dixon et al 2010 Fukuda et al 2010	CO_334:0100572	ug/g	Numerical	0	500	null
Dixon et al 2010 Fukuda et al 2010	CO_334:0100511	7pt scale	Ordinal			1 = no stress
Dixon et al 2010 Fukuda et al 2010	CO_334:0100573	days	Duration			null
Dixon et al 2010 Fukuda et al 2010	CO_334:0100515	8pt scale	Ordinal			0 = Absent
Dixon et al 2010 Fukuda et al 2010	CO_334:0100516	2pt scale	Nominal			1 = Present
Dixon et al 2010 Fukuda et al 2010	CO_334:0100570	3pt scale	Nominal			1 = Tending toward vertical
Dixon et al 2010 Fukuda et al 2010	CO_334:0100517	3pt scale	Nominal			1 = White or cream
Dixon et al 2010 Fukuda et al 2010	CO_334:0100518	3pt scale	Nominal			1 =
Dixon et al 2010 Fukuda et al 2010	CO_334:0100571	7pt scale	Nominal			1 =
Dixon et al 2010 Fukuda et al 2010	CO_334:0100569	4pt scale	Nominal			1 = White or cream
Dixon et al 2010 Fukuda et al 2010	CO_334:0100561	5pt scale	Nominal			1 = White
Dixon et al 2010 Fukuda et al 2010	CO_334:0100562	5pt scale	Nominal			1 = White
Dixon et al 2010 Fukuda et al 2010	CO_334:0100560	5pt scale	Nominal			1 = White
Dixon et al 2010 Fukuda et al 2010	CO_334:0100559	5pt scale	Nominal			1 = White or cream
Dixon et al 2010 Fukuda et al 2010	CO_334:0100563	3pt scale	Nominal			1 = Cream
Dixon et al 2010 Fukuda et al 2010	CO_334:0100507	4pt scale	Nominal			1 = Silver
Dixon et al 2010 Fukuda et al 2010	CO_334:0100568	5pt scale	Nominal			1 = Brown
Dixon et al 2010 Fukuda et al 2010	CO_334:0100497	4pt scale	Nominal			1 = White or cream
Dixon et al 2010 Fukuda et al 2010	CO_334:0100583	Pct (%)	Numerical	0	100	null
Dixon et al 2010 Fukuda et al 2010	CO_334:0100584	Pct (%)	Numerical	0	100	null
Dixon et al 2010 Fukuda et al 2010	CO_334:0100579	Pct (%)	Numerical	0	100	null
Dixon et al 2010 Fukuda et al 2010	CO_334:0100576	ug/g	Numerical	0	500	null
Dixon et al 2010 Fukuda et al 2010	CO_334:0100520	Pct (%)	Numerical	0	100	null
[illegible]	[illegible]	[illegible]	[illegible]	[illegible]	[illegible]	[illegible]

IRIS:186, 186SES:36	CO_320:0000607	ragged stunt disease scale	Ordinal			IRIS_SCALE:62	1= No symptom observed	[illegible]	[illegible]	[illegible]	[illegible]	null	null	null	null
IRIS:213, SES:50	CO_320:0000608	rat damage incidence scale	Ordinal			null	0= No damage observed	9= 26-100%	1= Less than 5%	5= 6-25%	null	null	null	null	null
IRIS:534, RO:7.3.36	CO_320:0000285	rhizome formation scale 1	Nominal			null	1= Vegetative crown	[illegible]	5= Strong rhizomes and no tubers	2= Vegetative crown and stolon	[illegible]	6= Strong rhizomes with tubers	null	null	null
SES:68	CO_320:0000389	rice bug damage scale	Ordinal			null	0= No injury	5= 8-15%	7= 12-25%	1= Less than 3%	3= 4-7%	9= 26-100%	null	null	null
SES:62	CO_320:0000609	[illegible]	Ordinal			null	0= No injury	[illegible]	[illegible]	[illegible]	3= Some damage/leaf discoloration	9= All plants dead	null	null	null
IRIS:124, SES:62	CO_320:0000610	rice delphacid injury scale field	Ordinal			null	0= No damage	[illegible]	[illegible]	[illegible]	3= Some damage/leaf discoloration	9= All plants dead	null	null	null
IRIS:1, SES:36	CO_320:0000909	rice tungro disease scale	Ordinal			null	1= No symptom observed	[illegible]	[illegible]	[illegible]	[illegible]	null	null	null	null
IRIS:2, SES:36	CO_320:0000910	%	Numerical			null	null	null	null	null	null	null	null	null	null
IRIS:64, SES:67	CO_320:0000387	rice whorl maggot damage scale	Ordinal			null	0= No injury	5= 1/3 to 1/2 of leaves injured	[illegible]	1= Less than 2 leaves/hill injured	[illegible]	[illegible]	null	null	null
IRIS:5, SES:71	CO_320:0000611	salt injury scale	Ordinal			null	1= Growth and tillering nearly normal	[illegible]	9= Almost all plants dead or dying	[illegible]	[illegible]	null	null	null	null
IRIS:146, RO:8.1.2	CO_320:0000612	sensed aroma cooked scale	Ordinal			null	0= Non-scented	1= Lightly scented	2= Scented	null	null	null	null	null	null
IRIS:860, SES:129, UPOV:65	CO_320:0000613	sensed aroma uncooked scale	Ordinal			null	0= Non-scented	1= Lightly scented	2= Scented	null	null	null	null	null	null
[illegible]	CO_320:0000421	secondary panicle branching scale	Nominal			null	0= Absent	3= Clustered	1= Light	2= Heavy	null	null	null	null	null
null	CO_320:0000614	%	Numerical			null	null	null	null	null	null	null	null	null	null
RO:7.2.1	CO_320:0000615	ISO Date (yyyy-mm-dd)	Date			null	null	null	null	null	null	null	null	null	null
RO:7.3.2, SES:91	CO_320:0000233	cm	Numerical			null	null	null	null	null	null	null	null	null	null
RO:7.3.2, SES:91	CO_320:0000228	seedling height scale	Ordinal			null	3= Short <30 cm	5= Intermediate ~45 cm	7= Tall >60 cm	null	null	null	null	null	null
IRIS:45, SES:37	CO_320:0000355	sheath blight relative height scale	Ordinal			null	0= No infection observed	3= 20-30%	4=	[illegible]	2=	7= 46-65%	8=	5= 31-45%	6=
IRIS:54, SES:38	CO_320:0000361	sheath rot incidence scale	Ordinal			null	0= No disease observed	3= 1-5%	4=	1= Less than 1%	2=	7= 26-50%	8=	5= 6-25%	6=
IRIS:694, RO:7.5.14, SES:9	CO_320:0000303	spikelet fertility scale RO	Ordinal			null	1= Completely sterile 0%	4= Fertile 75-90%	5= Highly fertile >90%	2= Highly sterile 1-49%	3= Partly sterile 50-74%	null	null	null	null
IRIS:694, RO:7.5.14, SES:9	CO_320:0000482	spikelet fertility scale SES	Ordinal			null	1= Highly fertile >90%	7= Highly sterile <50% to trace	9= Completely sterile 0%	3= Fertile 75-89%	5= Partly sterile 50-74%	null	null	null	null
IRIS:694, RO:7.5.14, SES:9	CO_320:0000482	spikelet fertility scale SES	Ordinal			null	1= Highly fertile >90%	7= Highly sterile <50% to trace	9= Completely sterile 0%	3= Fertile 75-89%	5= Partly sterile 50-74%	null	null	null	null
IRIS:197, SES:218	CO_320:0001288	spikelet sterility scale	Ordinal			IRIS_SCALE:192	1= Completely 100% sterile	7= Partially sterile (70%-94.9%)	[illegible]	3= Highly sterile (99%-99.9%)	5= Sterile (95%-98.9%)	null	null	null	null
IRIS:42, SES:63	CO_320:0000379	[illegible]	Ordinal			null	0= No injury	5= 21-30%	7= 31-60%	1= 1-10%	3= 11-20%	9= 61% and above	null	null	null
IRIS:62, SES:63	CO_320:0000623	stem borer deadheart damage scale	Ordinal			null	0= No injury	5= 21-30%	7= 31-60%	1= 1-10%	3= 11-20%	9= 61% and above	null	null	null
SES:63	CO_320:0000616	stem borer whitehead damage scale	Ordinal			null	0= No damage	5= 11-15%	7= 16-25%	1= 1-5%	3= 6-10%	9= 26% and above	null	null	null
SES:63	CO_320:0000617	stem borer deepwater damage scale	Ordinal			null	0= No damage	5= 21-30%	7= 31-60%	1= 1-10%	3= 11-20%	9= 61% and above	null	null	null
IRIS:34, SES:85	CO_320:0000407	stem elongation scale	Ordinal			null	[illegible]	[illegible]	[illegible]	[illegible]	[illegible]	null	null	null	null
SES:42	CO_320:0000367	stem rot incidence scale	Ordinal			null	0= No disease observed	3= 1-5%	4=	1= Less than 1%	2=	7= 26-50%	8=	5= 6-25%	6=
[illegible]	CO_320:0000301	sterile lemma color scale cult	Nominal			null	1= 020= Straw	4= 080= Purple	2= 040= Gold	3= 070= Red	null	null	null	null	null
RO:7.5.13	CO_320:0000907	sterile lemma color scale wild	Nominal			null	1= 011= Whitish	4= 060= Green	5= 061= Light green	2= 020= Straw	3= 040= Gold	8= 080= Purple	9= 082= Reddish purple	6= 062= Yellowish green	7= 070= Red
IRIS:325, RO:7.5.10, UPOV:51	CO_320:0000224	sterile lemma length scale	Ordinal			null	3= Short	9= Extra long	5= Medium	7= Long	null	null	null	null	null
IRIS:325, RO:7.5.11, UPOV:51	CO_320:0000618	longer sterile lemma length scale	Ordinal			null	3= Short	9= Extra long	5= Medium	7= Long	null	null	null	null	null
IRIS:325, RO:7.5.10, UPOV:51	CO_320:0000223	mm	Numerical			null	null	null	null	null	null	null	null	null	null
IRIS:529, RO:7.5.12	CO_320:0000299	Sterile lemma shape scale	Nominal			null	0= Absent	3= Triangular (and very small)	1= Linear(long and slender)	2= Subulate or setaceous	null	null	null	null	null
[illegible]	CO_320:0000226	stigma color scale 1	Nominal			null	1= 010= White	4= 081= Light purple	5= 080= Purple	2= 061= Light green	3= 030= Yellow	null	null	null	null
IRIS:165, SES:25	CO_320:0000335	stigma exsertion scale	Ordinal			null	1= above 70%	7= 11-20%	9= 0-10%	3= 41-70%	5= 21-40%	null	null	null	null
SES:86	CO_320:0000619	submergence relative survival scale	Ordinal			null	[illegible]	7= 50-74%	9= 0-49%	3= 95-99%	5= 75-94%	null	null	null	null
IRIS:3, SES:86	CO_320:0000409	submergence survival scale	Ordinal			null	1= 100% of plants survive	7= 50-74%	9= 0-49%	3= 95-99%	5= 75-94%	null	null	null	null
IRIS:74, SES:69	CO_320:0000391	thrips damage scale	Ordinal			null	[illegible]	[illegible]	[illegible]	[illegible]	[illegible]	null	null	null	null
SES:2	CO_320:0000315	Tillering scale	Ordinal			null	[illegible]	7= Few: 5-9 tillers/plant	9= Very few: fewer than 5 tillers/plant	3= Many: 20-25 tillers/plant	5= Medium: 10-19 tillers/plant	null	null	null	null
IRIS:181, SES:41	CO_320:0000365	udbatta disease incidence scale	Ordinal			null	0= No disease observed	3=	4=	1= Less than 1%	2=	7=	8=	5= 1-25%	6=
IRIS:215, SES:43	CO_320:0000369	ufra incidence scale	Ordinal			null	0= 0%	5= 41-60%	7= 61-80%	1= 1-2%	3= 21-40%	9= 81-100%	null	null	null
IRIS:698, SES:1	CO_320:0000337	seedling vigor scale	Ordinal			null	1= Extra vigorous, very fast growing	7= Weak, plants somewhat stunted	[illegible]	3= Vigorous, fast growing	5= Normal	null	null	null	null
SES:1	CO_320:0000313	vegetative vigor scale	Ordinal			null	[illegible]	[illegible]	[illegible]	[illegible]	5= Normal, most plants with 4-leaves	null	null	null	null
IRIS:41, SES:62	CO_320:0000375	[illegible]	Ordinal			null	0= No damage	[illegible]	[illegible]	1= Very slight damage	[illegible]	9= All plants dead	null	null	null
IRIS:198, SES:62	CO_320:0000620	[illegible]	Ordinal			null	0= No injury	[illegible]	[illegible]	1= Very slight injury	[illegible]	9= All plants dead	null	null	null
SES:36	CO_320:0000621	yellow dwarf disease scale	Ordinal			null	[illegible]	[illegible]	[illegible]	[illegible]	[illegible]	null	null	null	null
IRIS:174, SES:36	CO_320:0000357	yellow mottle disease scale	Ordinal			null	1= No symptom observed	[illegible]	[illegible]	[illegible]	[illegible]	null	null	null	null
IRIS:11, SES:74	CO_320:0000397	zinc deficiency injury scale	Ordinal			null	[illegible]	[illegible]	[illegible]	[illegible]	[illegible]	9= Almost all plants dead or dying	null	null	null
IRIS:108	CO_320:0000472	%	Numerical			null	null	null	null	null	null	null	null	null	null
IRIS:78, SES:76	CO_320:0000401	flowering heat injury scale	Ordinal			null	1= More than 80%	7= 11-40%	9= Less than 11%	3= 61-80%	5= 41-60%	null	null	null	null

[illegible]

Roelfs et al., 1992	CO_321:0000443	text	Text				null	null	null	null	null	null	null	null	null
Roelfs et al., 1992	CO_321:0000768	0-4 PRECO scale	Ordinal	1			[illegible]	[illegible]	[illegible]	[illegible]	[illegible]	null	null	null	null
Roelfs et al., 1992	CO_321:0000773	1-4 PRECO scale	Ordinal	1			[illegible]	[illegible]	[illegible]	[illegible]	null	null	null	null	null
[illegible]	CO_321:0000473	1-8 rust field response scale	Ordinal	1			0= no visible infection (0)	[illegible]	[illegible]	[illegible]	[illegible]	[illegible]	[illegible]	[illegible]	null
[illegible]	CO_321:0000606	0-8 Rust infection type	Ordinal	1			[illegible]	[illegible]	[illegible]	[illegible]	[illegible]	[illegible]	[illegible]	[illegible]	2= Heterogeneous, ordered distribution of variable-sized uredinia, with larger uredinia at leaf base (2)
[illegible]	CO_321:0000410	%	Numerical	1	0	100	null	null	null	null	null	null	null	null	null
[illegible]	CO_321:0000753	0-12 Modified Cobb Scale	Ordinal	1			0= 0% (no uredia)	10= 10% (3.7% occupied by uredia)	20= 20% (7.4% occupied by uredia)	1= 1% (0.37% occupied by uredia)	5= 5% (1.85% occupied by uredia)	50= 50% (18.5% occupied by uredia)	60= 60% (22.2% occupied by uredia)	30= 30% (11.1% occupied by uredia)	40= 40% (14.8% occupied by uredia)
Pask et al., 2012 (Ch. 2)	CO_321:0000429	mmol/m2/s	Numerical	0			null	null	null	null	null	null	null	null	null
null	CO_321:0000442	stoma/mm2	Numerical	1			null	null	null	null	null	null	null	null	null
Pask et al., 2012 (Ch. 2)	CO_321:0000431	s/m	Numerical	1	0	200	null	null	null	null	null	null	null	null	null
null	CO_321:0000415	?/m	Numerical		0.1	0.5	null	null	null	null	null	null	null	null	null
null	CO_321:0000415	?/m	Numerical		0.1	0.5	null	null	null	null	null	null	null	null	null
null	CO_321:0000410	%	Numerical	1	0	100	null	null	null	null	null	null	null	null	null
null	CO_321:0000428	mm	Numerical	1	0.1	5	null	null	null	null	null	null	null	null	null
[illegible]	CO_321:0000727	0-9 percentage scale + text	Text	1			null	null	null	null	null	null	null	null	null
[illegible]	CO_321:0000410	%	Numerical	1	0	100	null	null	null	null	null	null	null	null	null
[illegible]	CO_321:0000807	0-5 percentage scale	Ordinal	1			0= No visible affection	3= 60% coverage	4= 80% coverage	1= 20% coverage	2= 40% coverage	5= 100% coverage	null	null	null
[illegible]	CO_321:0000446	0-9 percentage scale	Ordinal	1			0= No visible affection	3= 30% coverage	4= 40% coverage	1= 10% coverage	2= 20% coverage	7= 70% coverage	8= 80% coverage	5= 50% coverage	6= 60% coverage
[illegible]	CO_321:0000457	1-5 DMG scale	Ordinal	1			1= none (N)	4= moderate (M)	5= very severe (V)	2= trace (T)	3 = slight (S)	null	null	null	null
[illegible]	CO_321:0000465	1-9 incidence scale	Ordinal	1			1= none	4= low to medium	5= medium	2= very low	3= low	8= very high	9= severe	6= medium to high	7= high
Wilcoxson and Saari, 1996	CO_321:0000410	%	Numerical	1	0	100	null	null	null	null	null	null	null	null	null
Wilcoxson and Saari, 1996	CO_321:0000465	1-9 incidence scale	Ordinal	1			1= none	4= low to medium	5= medium	2= very low	3= low	8= very high	9= severe	6= medium to high	7= high
Zadoks et al., 1974; Pask et al., 2012	CO_321:0000856	1-6 Phenological relative scale	Ordinal				1= very early (VE)	4= normal, similar to local check (N)	5= late (L)	2= early (E)	3= early to normal (E-N)	6= very late (VL)	null	null	null
Zadoks et al., 1974; Pask et al., 2012	CO_321:0000855	Julian date (JD)	Time				null	null	null	null	null	null	null	null	null
Zadoks et al., 1974; Pask et al., 2012	CO_321:0000850	yyyymmdd	Time				null	null	null	null	null	null	null	null	null
Zadoks et al., 1974; Pask et al., 2012	CO_321:0000434	day	Duration	0	40	240	null	null	null	null	null	null	null	null	null
Zadoks et al., 1974; Pask et al., 2012	CO_321:0000460	1-5 Phenological relative scale	Ordinal	0			VE= very early (VE)	L= late (L)	VL= very late (VL)	E= early (E)	M= moderate (M)	null	null	null	null
[illegible]	CO_321:0000410	%	Numerical	1	0	100	null	null	null	null	null	null	null	null	null
[illegible]	CO_321:0000470	Double digit (00-99)	Ordinal	1			[illegible]	[illegible]	[illegible]	[illegible]	20= 20	[illegible]	[illegible]	[illegible]	21= 20% plant height affected, 10% coverage
null	CO_321:0000437	node	Numerical	1	1	15	null	null	null	null	null	null	null	null	null
null	CO_321:0000450	0-9 density scale	Ordinal	1			0= absent	3= sparse	4= sparse to intermediate	1= very sparse	2= very sparse to sparse	7= dense	8= dense to very dense	5= intermediate	6= intermediate to dense
null	CO_321:0000411	cm	Numerical	1	0	2.5	null	null	null	null	null	null	null	null	null
null	CO_321:0000410	%	Numerical	1	0	100	null	null	null	null	null	null	null	null	null
null	CO_321:0000450	0-9 density scale	Ordinal	1			0= absent	3= sparse	4= sparse to intermediate	1= very sparse	2= very sparse to sparse	7= dense	8= dense to very dense	5= intermediate	6= intermediate to dense
null	CO_321:0000462	1-5 scoring scale	Ordinal	1			1= very poor (VP)	4= good (G)	5= very good (VG)	2= poor (P)	3= fair (F)	null	null	null	null
null	CO_321:0000458	1-5 GHABIT scale	Ordinal	1			E= erect (E)	S= spreading (S)	P= Prostrate (P)	SE= semi-erect (SE)	SS= semi-spreading (SS)	null	null	null	null
[illegible]	CO_321:0000411	cm	Numerical	1	0	2.5	null	null	null	null	null	null	null	null	null
[illegible]	CO_321:0000813	m	Numerical	2	0	2.5	null	null	null	null	null	null	null	null	null
[illegible]	CO_321:0000461	1-5 RELTPH scale	Ordinal	0			VS= very short (VS)	T= tall (T)	VT= very tall (VT)	S= short (S)	M= medium (M)	null	null	null	null
null	CO_321:0000599	0-1 absent-present	Nominal				1= present	0= absent	null	null	null	null	null	null	null
null	CO_321:0000857	1-9 PHOTORESP scale	Ordinal	1			0= insensitive	3= low	4= low to intermediate	1= very low	2= very low to low	7= moderate	8= moderate to high	5= intermediate	6= intermediate to moderate
null	CO_321:0000814	plant/ha	Numerical	0			null	null	null	null	null	null	null	null	null
null	CO_321:0000817	plant/m2	Numerical	0			null	null	null	null	null	null	null	null	null
null	CO_321:0000815	plant/m	Numerical	0			null	null	null	null	null	null	null	null	null
null	CO_321:0000816	plant/plot	Numerical	0			null	null	null	null	null	null	null	null	null
Zadoks et al., 1974; Pask et al., 2012	CO_321:0000434	day	Duration	0	40	240	null	null	null	null	null	null	null	null	null
Zadoks et al., 1974; Pask et al., 2012	CO_321:0000410	%	Numerical	1	0	100	null	null	null	null	null	null	null	null	null
null	CO_321:0000462	1-5 scoring scale	Ordinal	1			1= very poor (VP)	4= good (G)	5= very good (VG)	2= poor (P)	3= fair (F)	null	null	null	null
[illegible]	CO_321:0000727	0-9 percentage scale + text	Text	1			null	null	null	null	null	null	null	null	null
[illegible]	CO_321:0000410	%	Numerical	1	0	100	null	null	null	null	null	null	null	null	null
[illegible]	CO_321:0000782	0-5 incidence scale	Ordinal	1			0= none	3= medium	4= high	1= very low	2= low	5= severe	null	null	null
[illegible]	CO_321:0000788	1-5 incidence scale	Ordinal	1			1= none	4= high	5= severe	2= low	3= medium	null	null	null	null

[illegible]

Huaman1991:Def_4.3.1	CO_331:0000562	FlrHab 4 pt. Scale	Ordinal					0 = None	7 = Profuse	3 = Sparse	5 = Moderate	null	null	null	null	null
Huaman1991:Def_4.3.3	CO_331:0000563	cm	Continuous		2	0	10	null	null	null	null	null	null	null	null	null
Huaman1991:Def_4.3.4	CO_331:0000564	FlrShL 3 pt. Scale	Ordinal					3 = Semi-stellate	5 = Pentagonal	7 = Rounded	null	null	null	null	null	null
Huaman1991:Def_4.3.5	CO_331:0000565	SepEql 2 pt. Scale	Ordinal					2 = Equal	1 = Outer two shorter	null	null	null	null	null	null	null
Huaman1991:Def_4.3.6	CO_331:0000566	number	Continuous		0	0		null	null	null	null	null	null	null	null	null
Huaman1991:Def_4.3.7	CO_331:0000567	SepShp 5 pt. Scale	Ordinal					1 = Ovate	7 = Oblong	9 = Lanceolate	3 = Elliptic	5 = Obovate	null	null	null	null
Huaman1991:Def_4.3.8	CO_331:0000568	SepApx 4 pt. Scale	Ordinal					1 = Acute	7 = Caudate	3 = Obtuse	5 = Acuminate	null	null	null	null	null
Huaman1991:Def_4.3.9	CO_331:0000569	SepPub 4 pt. Scale	Ordinal					0 = Absent	7 = Heavy	3 = Sparse	5 = Moderate	null	null	null	null	null
Huaman1991:Def_4.3.10	CO_331:0000570	SepCol 7 pt. Scale	Ordinal					1 = Green	5 = Green with purple areas	6 = Some sepals green, others purple	2 = Green with purple edge	3 = Green with purple spots	9 = Totally pigmented - dark purple	null	null	null
Huaman1991:Def_4.3.11	CO_331:0000571	StgCol 3 pt. Scale	Ordinal					1 = White	5 = Pale purple	9 = Purple	null	null	null	null	null	null
Huaman1991:Def_4.3.12	CO_331:0000572	StyCol 5 pt. Scale	Ordinal					1 = White	[illegible]	9 = Purple	3 = White with purple at the base	5 = White with purple at the top	null	null	null	null
Huaman1991:Def_4.3.13	CO_331:0000573	StgExt 4 pt. Scale	Ordinal					[illegible]	[illegible]	3 = Same height as highest anther	5 = Slighty exerted	null	null	null	null	null
Huaman1991:Def_4.3.14	CO_331:0000574	SedCap 5 pt. Scale	Ordinal					0 = None	5 = Moderate	7 = Profuse	1 = Scarce	3 = Sparse	null	null	null	null
Huaman1991:Def_6.1.1	CO_331:0000575	StrFor 4 pt. Scale	Ordinal					1 = Closed cluster	7 = Very dispersed	3 = Open cluster	5 = Dispersed	null	null	null	null	null
Huaman1991:Def_6.1.2	CO_331:0000576	StrStk 6 pt. Scale	Ordinal					0 = Sessile or absent	5 = Intermediate (6-8 cm)	7 = Long (9-12 cm)	1 = Very short (<2 cm)	3 = Short (2-5 cm)	9 = Very long (>12 cm)	null	null	null
Huaman1991:Def_6.1.4	CO_331:0000577	VRtsSh 3 pt. Scale	Ordinal					3 = Uniform	5 = Slightly variable	7 = Moderately variable	null	null	null	null	null	null
Huaman1991:Def_6.1.5	CO_331:0000578	VRtsSiz 3 pt. Scale	Ordinal					3 = Uniform	5 = Slightly variable	7 = Moderately variable	null	null	null	null	null	null
Huaman1991:Def_6.2.7	CO_331:0000579	KQtyRts 3 pt. Scale	Ordinal					3 = Poor	5 = Medium	7 = Good	null	null	null	null	null	null
Huaman1991:Def_6.2.9.1	CO_331:0000580	CBolRts 9 pt. Scale	Ordinal					1 = Watery	4 = Soft	5 = Slighty hard	2 = Extremely soft	3 = Very soft	8 = Very hard	9 = Very hard and non-cooked	6 = Moderately hard	7 = Hard
Huaman1991:Def_6.2.9.2	CO_331:0000581	UColBRts 10 pt. Scale	Ordinal					0 = None	3 = Slighty green or grey	4 = Green	1 = Some beige	2 = Much beige	7 = Beige and grey	8 = Beige and purple	5 = Grey	6 = Beige and green
Huaman1991:Def_7.1	CO_331:0000582	RctDro 5 pt. Scale	Ordinal					1 = Very low	7 = High	9 = Very High	3 = Low	5 = Intermediate	null	null	null	null
Huaman1991:Def_7.2	CO_331:0000583	RctFlo 5 pt. Scale	Ordinal					1 = Very low	7 = High	9 = Very High	3 = Low	5 = Intermediate	null	null	null	null
Huaman1991:Def_7.3	CO_331:0000584	RctHeat 5 pt. Scale	Ordinal					1 = Very low	7 = High	9 = Very High	3 = Low	5 = Intermediate	null	null	null	null
Huaman1991:Def_7.4	CO_331:0000585	RctSty 5 pt. Scale	Ordinal					1 = Very low	7 = High	9 = Very High	3 = Low	5 = Intermediate	null	null	null	null
Huaman1991:Def_7.5	CO_331:0000586	RctShd 5 pt. Scale	Ordinal					1 = Very low	7 = High	9 = Very High	3 = Low	5 = Intermediate	null	null	null	null
Huaman1991:Def_7.6	CO_331:0000587	RctSph 5 pt. Scale	Ordinal					1 = Very low	7 = High	9 = Very High	3 = Low	5 = Intermediate	null	null	null	null
Huaman1991:Def_7.7	CO_331:0000588	RctSTp 5 pt. Scale	Ordinal					1 = Very low	7 = High	9 = Very High	3 = Low	5 = Intermediate	null	null	null	null
Huaman1991:Def_8.1.2	CO_331:0000589	RcWSPW 5 pt. Scale	Ordinal					1 = Very low	7 = High	9 = Very High	3 = Low	5 = Intermediate	null	null	null	null
Huaman1991:Def_8.1.3	CO_331:0000590	RcSSPW 5 pt. Scale	Ordinal					1 = Very low	7 = High	9 = Very High	3 = Low	5 = Intermediate	null	null	null	null
Huaman1991:Def_8.1.4	CO_331:0000591	RcSPWW 5 pt. Scale	Ordinal					1 = Very low	7 = High	9 = Very High	3 = Low	5 = Intermediate	null	null	null	null
Huaman1991:Def_8.1.5	CO_331:0000592	RcWW 5 pt. Scale	Ordinal					1 = Very low	7 = High	9 = Very High	3 = Low	5 = Intermediate	null	null	null	null
Huaman1991:Def_8.1.6	CO_331:0000593	RcSPFB 5 pt. Scale	Ordinal					1 = Very low	7 = High	9 = Very High	3 = Low	5 = Intermediate	null	null	null	null
Huaman1991:Def_8.1.7	CO_331:0000594	RcFB 5 pt. Scale	Ordinal					1 = Very low	7 = High	9 = Very High	3 = Low	5 = Intermediate	null	null	null	null
Huaman1991:Def_8.1.8	CO_331:0000595	RcSPLB 5 pt. Scale	Ordinal					1 = Very low	7 = High	9 = Very High	3 = Low	5 = Intermediate	null	null	null	null
Huaman1991:Def_8.1.9	CO_331:0000596	RcBTL 5 pt. Scale	Ordinal					1 = Very low	7 = High	9 = Very High	3 = Low	5 = Intermediate	null	null	null	null
Huaman1991:Def_8.1.10	CO_331:0000597	RcGrbW 5 pt. Scale	Ordinal					1 = Very low	7 = High	9 = Very High	3 = Low	5 = Intermediate	null	null	null	null
Huaman1991:Def_8.1.11	CO_331:0000598	RcHmw 5 pt. Scale	Ordinal					1 = Very low	7 = High	9 = Very High	3 = Low	5 = Intermediate	null	null	null	null
Huaman1991:Def_8.1.12	CO_331:0000599	RcAph 5 pt. Scale	Ordinal					1 = Very low	7 = High	9 = Very High	3 = Low	5 = Intermediate	null	null	null	null
Huaman1991:Def_8.1.13	CO_331:0000600	RcSPWF 5 pt. Scale	Ordinal					1 = Very low	7 = High	9 = Very High	3 = Low	5 = Intermediate	null	null	null	null
Huaman1991:Def_8.1.14	CO_331:0000601	RcSPMth 5 pt. Scale	Ordinal					1 = Very low	7 = High	9 = Very High	3 = Low	5 = Intermediate	null	null	null	null
Huaman1991:Def_8.1.15	CO_331:0000602	RcMth 5 pt. Scale	Ordinal					1 = Very low	7 = High	9 = Very High	3 = Low	5 = Intermediate	null	null	null	null
Huaman1991:Def_8.1.16	CO_331:0000603	RcSPSB 5 pt. Scale	Ordinal					1 = Very low	7 = High	9 = Very High	3 = Low	5 = Intermediate	null	null	null	null
Huaman1991:Def_8.1.17	CO_331:0000604	RcIns 5 pt. Scale	Ordinal					1 = Very low	7 = High	9 = Very High	3 = Low	5 = Intermediate	null	null	null	null
Huaman1991:Def_8.2.2	CO_331:0000605	RcRFN 5 pt. Scale	Ordinal					1 = Very low	7 = High	9 = Very High	3 = Low	5 = Intermediate	null	null	null	null
Huaman1991:Def_8.2.3	CO_331:0000606	RcStN 5 pt. Scale	Ordinal					1 = Very low	7 = High	9 = Very High	3 = Low	5 = Intermediate	null	null	null	null
Huaman1991:Def_8.2.4	CO_331:0000607	RcBRR 5 pt. Scale	Ordinal					1 = Very low	7 = High	9 = Very High	3 = Low	5 = Intermediate	null	null	null	null
Huaman1991:Def_8.2.5	CO_331:0000608	RcRLN 5 pt. Scale	Ordinal					1 = Very low	7 = High	9 = Very High	3 = Low	5 = Intermediate	null	null	null	null
Huaman1991:Def_8.2.6	CO_331:0000609	RcON 5 pt. Scale	Ordinal					1 = Very low	7 = High	9 = Very High	3 = Low	5 = Intermediate	null	null	null	null
Huaman1991:Def_8.3.1	CO_331:0000610	RcWR 5 pt. Scale	Ordinal					1 = Very low	7 = High	9 = Very High	3 = Low	5 = Intermediate	null	null	null	null
Huaman1991:Def_8.3.2	CO_331:0000611	RcFRS 5 pt. Scale	Ordinal					1 = Very low	7 = High	9 = Very High	3 = Low	5 = Intermediate	null	null	null	null
Huaman1991:Def_8.3.3	CO_331:0000612	RcFRR 5 pt. Scale	Ordinal					1 = Very low	7 = High	9 = Very High	3 = Low	5 = Intermediate	null	null	null	null
Huaman1991:Def_8.3.4	CO_331:0000613	RcSBC 5 pt. Scale	Ordinal					1 = Very low	7 = High	9 = Very High	3 = Low	5 = Intermediate	null	null	null	null
Huaman1991:Def_8.3.5	CO_331:0000614	RcBR 5 pt. Scale	Ordinal					1 = Very low	7 = High	9 = Very High	3 = Low	5 = Intermediate	null	null	null	null
Huaman1991:Def_8.3.6	CO_331:0000615	RcScrf 5 pt. Scale	Ordinal					1 = Very low	7 = High	9 = Very High	3 = Low	5 = Intermediate	null	null	null	null
Huaman1991:Def_8.3.7	CO_331:0000616	RcSR 5 pt. Scale	Ordinal					1 = Very low	7 = High	9 = Very High	3 = Low	5 = Intermediate	null	null	null	null

[illegible]

null	CO_325:0000071	leaf	Numerical	null			0	50	null	null
null	CO_325:0000071	leaf	Numerical	null			0	50	null	null
null	CO_325:0000071	leaf	Numerical	null			0	50	null	null
null	CO_325:0000071	leaf	Numerical	null			0	50	null	null
null	CO_325:0000071	leaf	Numerical	null			0	50	null	null
null	CO_325:0100088	5 pt corm discolouration scale	Nominal	null	null				[illegible]	[illegible]
null	CO_325:0100089	Presence absence 0	Nominal	null	null				1 = present	0 = absent
null	CO_325:0100089	Presence absence 0	Nominal	null	null				1 = present	0 = absent
null	CO_325:0100089	Presence absence 0	Nominal	null	null				1 = present	0 = absent
null	CO_325:0100089	Presence absence 0	Nominal	null	null				1 = present	0 = absent
null	CO_325:0000080	%	Numerical		1		0	100	null	null
null	CO_325:0000080	%	Numerical		1		0	100	null	null
null	CO_325:0000080	%	Numerical		1		0	100	null	null
null	CO_325:0000080	%	Numerical		1		0	100	null	null
null	CO_325:0100097	Ex: g/100 mL juice????	Numerical	null	null				null	null
[illegible]	null	null	CO_325:0100098	weevil	Numerical				null	null
[illegible]	null	null	CO_325:0100098	weevil	Numerical				null	null
[illegible]	null	null	CO_325:0100098	weevil	Numerical				null	null
Visual counting method	null	null	CO_325:0100098	weevil	Numerical				null	null
Visual counting method	null	null	CO_325:0100098	weevil	Numerical				null	null
null	CO_325:0100098	weevil	Numerical	null	null				null	null
null	CO_325:0100098	weevil	Numerical	null	null				null	null
null	CO_325:0100099	ÂM	Numerical	null	null				null	null
null	CO_325:0100099	ÂM	Numerical	null	null				null	null
null	CO_325:0100099	ÂM	Numerical	null	null				null	null
null	CO_325:0100099	ÂM	Numerical	null	null				null	null
null	CO_325:0100100	mg	Numerical	null	null				null	null
null	CO_325:0100100	mg	Numerical	null	null				null	null
null	CO_325:0100099	ÂM	Numerical	null	null				null	null
null	CO_325:0100099	ÂM	Numerical	null	null				null	null
null	CO_325:0100099	ÂM	Numerical	null	null				null	null
null	CO_325:0100099	ÂM	Numerical	null	null				null	null
null	CO_325:0100100	mg	Numerical	null	null				null	null
null	CO_325:0100100	mg	Numerical	null	null				null	null
null	CO_325:0000080	%	Numerical		1		0	100	null	null
null	CO_325:0000080	%	Numerical		1		0	100	null	null
null	CO_325:0000002	day	Duration	null			0	1000	null	null
null	CO_325:0000011	cm	Numerical		1		0	100	null	null
null	CO_325:0000011	cm	Numerical		1		0	100	null	null
null	CO_325:0000011	cm	Numerical		1		0	100	null	null
null	CO_325:0000011	cm	Numerical		1		0	100	null	null
null	CO_325:0100105	kg	Numerical		2		0.01	10	null	null
null	CO_325:0000035	kg	Numerical		2		0.01	10	null	null
null	CO_325:0000035	kg	Numerical		2		0.01	10	null	null
null	CO_325:0000068	leaf	Numerical		0		0	20	null	null
null	CO_325:0000080	%	Numerical		1		0	100	null	null
null	CO_325:0000080	%	Numerical		1		0	100	null	null
null	CO_325:0000080	%	Numerical		1		0	100	null	null
null	CO_325:0000213	yyyy/mm/dd	Time	null	null				null	null
null	CO_325:0000318	hand rank	Numerical		0		1	20	null	null
null	CO_325:0100126	dd/mm/yyyy hh:mm:ss	Time	null	null				null	null
null	CO_325:0100126	dd/mm/yyyy hh:mm:ss	Time	null	null				null	null
null	CO_325:0100127	photograph	Photo	null	null				null	null
null	CO_325:0100127	photograph	Photo	null	null				null	null
null	CO_325:0100127	photograph	Photo	null	null				null	null
null	CO_325:0100127	photograph	Photo	null	null				null	null
null	CO_325:0100127	photograph	Photo	null	null				null	null
null	CO_325:0100127	photograph	Photo	null	null				null	null
null	CO_325:0100127	photograph	Photo	null	null				null	null
null	CO_325:0100127	photograph	Photo	null	null				null	null
[illegible]	null	null	CO_325:0100128	Presence absence 0	Nominal				null	null
null	CO_325:0100000	dd/mm/yyyy	Time	null	null				null	null
null	CO_325:0100130	open answer	Text	null	null				null	null
null	CO_325:0100130	open answer	Text	null	null				null	null
null	CO_325:0100130	open answer	Text	null	null				null	null

[illegible]

[illegible]	CO_322:0000981	1-9 disease scoring scale	Ordinal			1= no infection	4= low to medium infection	5= medium infection	2= very low infection	3= low infection	8= very high infection	9= severe infection	6= medium to high infection	7= high infection
[illegible]	CO_322:0000350	%	Numerical	0	1	null	null	null	null	null	null	null	null	null
[illegible]	CO_322:0000981	1-9 disease scoring scale	Ordinal			1= no infection	4= low to medium infection	5= medium infection	2= very low infection	3= low infection	8= very high infection	9= severe infection	6= medium to high infection	7= high infection
[illegible]	CO_322:0000350	%	Numerical	0	1	null	null	null	null	null	null	null	null	null
[illegible]	CO_322:0000350	%	Numerical	0	1	null	null	null	null	null	null	null	null	null
[illegible]	CO_322:0000981	1-9 disease scoring scale	Ordinal			1= no infection	4= low to medium infection	5= medium infection	2= very low infection	3= low infection	8= very high infection	9= severe infection	6= medium to high infection	7= high infection
[illegible]	CO_322:0000981	1-9 disease scoring scale	Ordinal			1= no infection	4= low to medium infection	5= medium infection	2= very low infection	3= low infection	8= very high infection	9= severe infection	6= medium to high infection	7= high infection
[illegible]	CO_322:0000350	%	Numerical	0	1	null	null	null	null	null	null	null	null	null
[illegible]	CO_322:0000350	%	Numerical	0	1	null	null	null	null	null	null	null	null	null
[illegible]	CO_322:0000981	1-9 disease scoring scale	Ordinal			1= no infection	4= low to medium infection	5= medium infection	2= very low infection	3= low infection	8= very high infection	9= severe infection	6= medium to high infection	7= high infection
[illegible]	CO_322:0000981	1-9 disease scoring scale	Ordinal			1= no infection	4= low to medium infection	5= medium infection	2= very low infection	3= low infection	8= very high infection	9= severe infection	6= medium to high infection	7= high infection
[illegible]	CO_322:0000350	%	Numerical	0	1	null	null	null	null	null	null	null	null	null
[illegible]	CO_322:0000981	1-9 disease scoring scale	Ordinal			1= no infection	4= low to medium infection	5= medium infection	2= very low infection	3= low infection	8= very high infection	9= severe infection	6= medium to high infection	7= high infection
[illegible]	CO_322:0000350	%	Numerical	0	1	null	null	null	null	null	null	null	null	null
[illegible]	CO_322:0000981	1-9 disease scoring scale	Ordinal			1= no infection	4= low to medium infection	5= medium infection	2= very low infection	3= low infection	8= very high infection	9= severe infection	6= medium to high infection	7= high infection
[illegible]	CO_322:0000350	%	Numerical	0	1	null	null	null	null	null	null	null	null	null
[illegible]	CO_322:0000981	1-9 disease scoring scale	Ordinal			1= no infection	4= low to medium infection	5= medium infection	2= very low infection	3= low infection	8= very high infection	9= severe infection	6= medium to high infection	7= high infection
[illegible]	CO_322:0000350	%	Numerical	0	1	null	null	null	null	null	null	null	null	null
null	CO_322:0000350	%	Numerical	0	1	null	null	null	null	null	null	null	null	null
null	CO_322:0000981	1-9 disease scoring scale	Ordinal			1= no infection	4= low to medium infection	5= medium infection	2= very low infection	3= low infection	8= very high infection	9= severe infection	6= medium to high infection	7= high infection
[illegible]	CO_322:0000350	%	Numerical	0	1	null	null	null	null	null	null	null	null	null
[illegible]	CO_322:0000981	1-9 disease scoring scale	Ordinal			1= no infection	4= low to medium infection	5= medium infection	2= very low infection	3= low infection	8= very high infection	9= severe infection	6= medium to high infection	7= high infection
null	CO_322:0000981	1-9 disease scoring scale	Ordinal			1= no infection	4= low to medium infection	5= medium infection	2= very low infection	3= low infection	8= very high infection	9= severe infection	6= medium to high infection	7= high infection
null	CO_322:0000350	%	Numerical	0	1	null	null	null	null	null	null	null	null	null
[illegible]	CO_322:0000981	1-9 disease scoring scale	Ordinal			1= no infection	4= low to medium infection	5= medium infection	2= very low infection	3= low infection	8= very high infection	9= severe infection	6= medium to high infection	7= high infection
[illegible]	CO_322:0000350	%	Numerical	0	1	null	null	null	null	null	null	null	null	null
[illegible]	CO_322:0000234	1-5 disease scoring scale	Ordinal			[illegible]	[illegible]	[illegible]	[illegible]	[illegible]	null	null	null	null
[illegible]	CO_322:0000981	1-9 disease scoring scale	Ordinal			1= no infection	4= low to medium infection	5= medium infection	2= very low infection	3= low infection	8= very high infection	9= severe infection	6= medium to high infection	7= high infection
[illegible]	CO_322:0000350	%	Numerical	0	1	null	null	null	null	null	null	null	null	null
[illegible]	CO_322:0000981	1-9 disease scoring scale	Ordinal			1= no infection	4= low to medium infection	5= medium infection	2= very low infection	3= low infection	8= very high infection	9= severe infection	6= medium to high infection	7= high infection
[illegible]	CO_322:0000350	%	Numerical	0	1	null	null	null	null	null	null	null	null	null
[illegible]	CO_322:0000981	1-9 disease scoring scale	Ordinal			1= no infection	4= low to medium infection	5= medium infection	2= very low infection	3= low infection	8= very high infection	9= severe infection	6= medium to high infection	7= high infection
[illegible]	CO_322:0000350	%	Numerical	0	1	null	null	null	null	null	null	null	null	null
[illegible]	CO_322:0000981	1-9 disease scoring scale	Ordinal			1= no infection	4= low to medium infection	5= medium infection	2= very low infection	3= low infection	8= very high infection	9= severe infection	6= medium to high infection	7= high infection
[illegible]	CO_322:0000350	%	Numerical	0	1	null	null	null	null	null	null	null	null	null
[illegible]	CO_322:0000981	1-9 disease scoring scale	Ordinal			1= no infection	4= low to medium infection	5= medium infection	2= very low infection	3= low infection	8= very high infection	9= severe infection	6= medium to high infection	7= high infection
[illegible]	CO_322:0000350	%	Numerical	0	1	null	null	null	null	null	null	null	null	null
[illegible]	CO_322:0000981	1-9 disease scoring scale	Ordinal			1= no infection	4= low to medium infection	5= medium infection	2= very low infection	3= low infection	8= very high infection	9= severe infection	6= medium to high infection	7= high infection
[illegible]	CO_322:0000350	%	Numerical	0	1	null	null	null	null	null	null	null	null	null
[illegible]	CO_322:0000981	1-9 disease scoring scale	Ordinal			1= no infection	4= low to medium infection	5= medium infection	2= very low infection	3= low infection	8= very high infection	9= severe infection	6= medium to high infection	7= high infection
[illegible]	CO_322:0000350	%	Numerical	0	1	null	null	null	null	null	null	null	null	null
[illegible]	CO_322:0000981	1-9 disease scoring scale	Ordinal			1= no infection	4= low to medium infection	5= medium infection	2= very low infection	3= low infection	8= very high infection	9= severe infection	6= medium to high infection	7= high infection
[illegible]	CO_322:0000350	%	Numerical	0	1	null	null	null	null	null	null	null	null	null
[illegible]	CO_322:0000981	1-9 disease scoring scale	Ordinal			1= no infection	4= low to medium infection	5= medium infection	2= very low infection	3= low infection	8= very high infection	9= severe infection	6= medium to high infection	7= high infection
[illegible]	CO_322:0000350	%	Numerical	0	1	null	null	null	null	null	null	null	null	null
[illegible]	CO_322:0000981	1-9 disease scoring scale	Ordinal			1= no infection	4= low to medium infection	5= medium infection	2= very low infection	3= low infection	8= very high infection	9= severe infection	6= medium to high infection	7= high infection
[illegible]	CO_322:0000350	%	Numerical	0	1	null	null	null	null	null	null	null	null	null
[illegible]	CO_322:0000981	1-9 disease scoring scale	Ordinal			1= no infection	4= low to medium infection	5= medium infection	2= very low infection	3= low infection	8= very high infection	9= severe infection	6= medium to high infection	7= high infection
[illegible]	CO_322:0000350	%	Numerical	0	1	null	null	null	null	null	null	null	null	null
null	CO_322:0000982	1-9 Anthocyanin coloration scale	Ordinal			1= absent or very weak	4= weak to medium	5= medium	2= very weak to weak	3= weak	8= strong to very strong	9= very strong	6= medium to strong	7= strong
null	CO_322:0000983	1-2 absent-present scale	Ordinal			2= present	1= absent	null	null	null	null	null	null	null
null	CO_322:0000982	1-9 Anthocyanin coloration scale	Ordinal			1= absent or very weak	4= weak to medium	5= medium	2= very weak to weak	3= weak	8= strong to very strong	9= very strong	6= medium to strong	7= strong
null	CO_322:0001088	1-3 Cob color scale	Ordinal			2= light purple	3= dark purple	1= white	null	null	null	null	null	null
[illegible]														

null	CO_327:0000005	Julian Days	Date				null	null	null	null	null	null	null	null	null
null	CO_327:0000008	Julian Days	Date				null	null	null	null	null	null	null	null	null
null	CO_327:0000011	Julian Days	Date				null	null	null	null	null	null	null	null	null
null	CO_327:0000014	Julian Days	Date				null	null	null	null	null	null	null	null	null
null	CO_327:0000017	Julian Days	Date				null	null	null	null	null	null	null	null	null
null	CO_327:0000020	Julian Days	Date				null	null	null	null	null	null	null	null	null
null	CO_327:0000023	Hill	Numerical				null	null	null	null	null	null	null	null	null
null	CO_327:0000026	Plant/plot	Numerical				null	null	null	null	null	null	null	null	null
[illegible]	CO_327:0000029	Vigor scale	Ordinal				1= Very Bad Vigor	4 = Good Vigor	5 = Excellent Vigor	2 = Bad Vigor	3 = Acceptable vigor	null	null	null	null
null	CO_327:0000032	Vigor scale	Ordinal				1= Very Bad Vigor	4 = Good Vigor	5 = Excellent Vigor	2 = Bad Vigor	3 = Acceptable vigor	null	null	null	null
null	CO_327:0000035	Plant/plot	Numerical				null	null	null	null	null	null	null	null	null
null	CO_327:0000038	Hill	Numerical				null	null	null	null	null	null	null	null	null
null	CO_327:0000041	Plant	Numerical				null	null	null	null	null	null	null	null	null
null	CO_327:0000044	Plant	Numerical				null	null	null	null	null	null	null	null	null
null	CO_327:0000047	bBlast reaction score	Ordinal				[illegible]	[illegible]	[illegible]	[illegible]	[illegible]	[illegible]	[illegible]	[illegible]	7=41-50% leaf area covered with large coalescing necrotic lesions, S
[illegible]	CO_327:0000050	%	Numerical		0	100	null	null	null	null	null	null	null	null	null
null	CO_327:0000053	Score (1-9)	Ordinal				[illegible]	[illegible]	[illegible]	[illegible]	[illegible]	[illegible]	[illegible]	[illegible]	7=41-50% leaf area covered with large coalescing necrotic lesions, S
[illegible]	CO_327:0000056	%	Numerical		0	100	null	null	null	null	null	null	null	null	null
[illegible]	CO_327:0000059	cm	Numerical		0	600	null	null	null	null	null	null	null	null	null
null	CO_327:0000062	cm	Numerical		0	150	null	null	null	null	null	null	null	null	null
null	CO_327:0000065	mm	Numerical		0	100	null	null	null	null	null	null	null	null	null
null	CO_327:0000068	mm	Numerical		0	200	null	null	null	null	null	null	null	null	null
[illegible]	CO_327:0000071	cm	Numerical		-50	50	null	null	null	null	null	null	null	null	null
null	CO_327:0000074	Plant	Numerical				null	null	null	null	null	null	null	null	null
null	CO_327:0000077	Hill	Numerical				null	null	null	null	null	null	null	null	null
null	CO_327:0000080	Panicle	Numerical				null	null	null	null	null	null	null	null	null
null	CO_327:0000083	Panicle	Numerical				null	null	null	null	null	null	null	null	null
null	CO_327:0000086	Panicle	Numerical				null	null	null	null	null	null	null	null	null
null	CO_327:0000089	Agronomic appreciation score	Ordinal				1 = Very poor	4 = Good	5 = Very good	2 = Poor	3 = Acceptable	null	null	null	null
null	CO_327:0000092	Farmer preference score	Ordinal				1 = Bad	4 = Good	5 = Very good	2 = With some problem	3 = Acceptable	null	null	null	null
null	CO_327:0000095	Preference score	Ordinal				1 = Variety to reject	2 = Need to test again	[illegible]	null	null	null	null	null	null
[illegible]	CO_327:0000098	Panicle compactness score	Ordinal				3= loose	5= intermediate	7= compact	null	null	null	null	null	null
null	CO_327:0000101	Presence-Absence	Nominal				0= absent	1= present	null	null	null	null	null	null	null
[illegible]	CO_327:0000104	cm	Numerical		0		null	null	null	null	null	null	null	null	null
null	CO_327:0000107	Bristle density scale	Ordinal				1 = Very poor	4 = Good	5 = Very good	2 = Poor	3 = Acceptable	null	null	null	null
[illegible]	CO_327:0000110	%	Numerical		0	100	null	null	null	null	null	null	null	null	null
[illegible]	CO_327:0000113	%	Numerical		0	100	null	null	null	null	null	null	null	null	null
null	CO_327:0000116	Striga/plot	Numerical				null	null	null	null	null	null	null	null	null
null	CO_327:0000119	%	Numerical		0	100	null	null	null	null	null	null	null	null	null
null	CO_327:0000122	No scale name found	null				null	null	null	null	null	null	null	null	null
null	CO_327:0000125	cm	Numerical				null	null	null	null	null	null	null	null	null
null	CO_327:0000128	cm	Numerical				null	null	null	null	null	null	null	null	null
[illegible]	CO_327:0000131	mm	Numerical				null	null	null	null	null	null	null	null	null
[illegible]	CO_327:0000134	Color	Numerical				null	null	null	null	null	null	null	null	null
[illegible]	CO_327:0000137	Tiller	Numerical				null	null	null	null	null	null	null	null	null
[illegible]	CO_327:0000140	Tiller	Numerical				null	null	null	null	null	null	null	null	null
[illegible]	CO_327:0000143	Number	Numerical				null	null	null	null	null	null	null	null	null
[illegible]	CO_327:0000146	g/100 grains	Numerical				null	null	null	null	null	null	null	null	null
null	CO_327:0000089	Agronomic appreciation score	Ordinal				1 = Very poor	4 = Good	5 = Very good	2 = Poor	3 = Acceptable	null	null	null	null
null	CO_327:0000092	Farmer preference score	Ordinal				1 = Bad	4 = Good	5 = Very good	2 = With some problem	3 = Acceptable	null	null	null	null
null	CO_327:0000095	Preference score	Ordinal				1 = Variety to reject	2 = Need to test again	[illegible]	null	null	null	null	null	null
NA	CO_337:0000111	1-5 Pod beak score	Ordinal	1			1= A= Absent	4= P= Prominent	5= VP= Very Prominent	2= Sl= Slight	3= M= Moderate	null	null	null	null
[illegible]	[illegible]	[illegible]	[illegible]	[illegible]	[illegible]	[illegible]	[illegible]	[illegible]	[illegible]	[illegible]	[illegible]	[illegible]	[illegible]	[illegible]	[illegible]

NA	CO_333:3000084	mm3	Numerical			null	null
NA	CO_333:3000084	mm3	Numerical			null	null
NA	CO_333:3000084	mm3	Numerical			null	null
NA	CO_333:3000091	Date	Time			null	null
NA	CO_333:3000089	Bract Distribution Scale	Ordinal	0	9	[0] absent	[1] some pres[illegible]
NA	CO_333:3000090	Flowering Time scale	Ordinal	1	7	[1] rosette stadium	[2] starting to[illegible]
NA	CO_333:3000091	Date	Time			null	null
NA	CO_333:3000091	Date	Time			null	null
NA	CO_333:3000092	[illegible]	Nominal	1	2	[1] Synchronous	[2] Asynchron[illegible]
NA	CO_333:3000093	[illegible]	Nominal	1	2	[1] Determinate	[2] Indetermin[illegible]
NA	CO_333:0000340	No scale name found	null			null	null
NA	CO_333:3000094	Seed Shattering scale	Ordinal	1	9	[1] very little	[9] very much[illegible]
NA	CO_333:3000095	Stem Color scale	Ordinal	1	3	[1] white	[2] yellow / lig[illegible]
NA	UO:0000027	°C	Numerical			null	null
NA	UO:0000027	°C	Numerical			null	null
NA	UO:0000027	°C	Numerical			null	null
NA	UO:0000027	°C	Numerical			null	null
NA	CO_333:3000096	mm/h	Numerical			null	null
NA	CO_333:3000096	mm/h	Numerical			null	null
NA	CO_333:3000097	[illegible]	Ordinal	1	5	[1] little	[5] moderate[illegible]
NA	CO_333:3000098	µg/cm² leaf	Numerical			null	null
NA	CO_333:3000099	kg/cm² leaf	Numerical			null	null
NA	CO_333:3000102	mEq/100g of fresh matter	Numerical			null	null
NA	CO_333:3000100	Germity scale	Ordinal	1	3	[1] Monogerm	[illegible]
NA	CO_333:3000102	mEq/100g of fresh matter	Numerical			null	null
NA	CO_333:3000101	g/100g of fresh matter	Numerical			null	null
NA	CO_333:3000101	g/100g of fresh matter	Numerical			null	null
NA	CO_333:3000102	mEq/100g of fresh matter	Numerical			null	null
NA	CO_333:3000102	mEq/100g of fresh matter	Numerical			null	null
NA	CO_333:3000102	mEq/100g of fresh matter	Numerical			null	null
NA	CO_333:3000102	mEq/100g of fresh matter	Numerical			null	null
NA	null	null	null			null	null
[illegible]	CO_335:0000149	5 categories (1 -5)	Categorical	null		[illegible]	1=Determinat[illegible]
CIAT 1987	CO_335:0000157	1 to 9	Categorical	null		[illegible]	1= No visible[illegible]
CIAT 1987	CO_335:0000159	1 to 9	Categorical	null		[illegible]	1= No visible[illegible]
[illegible]	CO_335:0000161	1 to 9	Categorical	null		[illegible]	1= No visible[illegible]
CIAT 1987	CO_335:0000163	1 to 9	Categorical	null		[illegible]	1= No visible[illegible]
CIAT 1987	CO_335:0000165	1 to 9	Categorical	null		[illegible]	1= No visible[illegible]
[illegible]	CO_335:0000167	1 to 9	Categorical	null		[illegible]	1= No visible[illegible]
CIAT 1987, Beebe 2012 (p.c.)	CO_335:0000169	1 to 9	Categorical	null		[illegible]	[illegible]
CIAT 1987, Duncan 2011	CO_335:0000171	1 to 9	Categorical	null		[illegible]	1= No visible[illegible]
CIAT 1987, Duncan 2011	CO_335:0000173	1 to 9	Categorical	null		[illegible]	1= No visible[illegible]
CIAT 1987, Duncan 2011	CO_335:0000175	1 to 9	Categorical	null		[illegible]	1= No visible[illegible]
CIAT 1987	CO_335:0000177	1 to 9	Categorical	null		[illegible]	1= No visible[illegible]
CIAT 1987	CO_335:0000179	1 to 9	Categorical	null		[illegible]	1= No visible[illegible]
CIAT 1987	CO_335:0000181	1 to 9	Categorical	null		[illegible]	1= No visible[illegible]
CIAT 1987	CO_335:0000183	1 to 9	Categorical	null		[illegible]	1= No visible[illegible]
CIAT 1987	CO_335:0000185	1 to 9	Categorical	null		[illegible]	1= No visible[illegible]
CIAT 1987	CO_335:0000187	1 to 9	Categorical	null		[illegible]	[illegible]
CIAT 1987	CO_335:0000189	1 to 9	Categorical	null		[illegible]	[illegible]
Beebe 2012 (p.c.)	CO_335:0000191	[illegible]	Categorical	null		null	null
CIAT 1987	CO_335:0000193	1 to 9	Categorical	null		[illegible]	1= No visible[illegible]
CIAT 1987	CO_335:0000195	1 to 9	Categorical	null		[illegible]	1= No visible[illegible]
Beebe et al. 2010	CO_335:0000197	kg/ha	Continuous		10000	null	null
Beebe et al. 2010	CO_335:0000199	°C	Continuous		50	null	null

[illegible]

null	CO_358:0000068	lb/acre	Numerical	3	0		null	null	NA	NA	NA	NA	NA	NA	NA
null	CO_358:0000069	mg/kg	Numerical	3	2		null	null	NA	NA	NA	NA	NA	NA	NA
null	CO_358:0000551	lint color CN scale	Nominal				8= dark green	null	NA	NA	NA	NA	NA	NA	NA
null	CO_358:0000069	mg/kg	Numerical	3	2		null	null	NA	NA	NA	NA	NA	NA	NA
null	CO_358:0000069	mg/kg	Numerical	3	2		null	null	NA	NA	NA	NA	NA	NA	NA
null	CO_358:0000558	lint color NCGC scale	Nominal				9= seg/off type	null	NA	NA	NA	NA	NA	NA	NA
null	CO_358:0000069	mg/kg	Numerical	3	2		null	null	NA	NA	NA	NA	NA	NA	NA
null	CO_358:0000069	mg/kg	Numerical	3	2		null	null	NA	NA	NA	NA	NA	NA	NA
null	CO_358:0000565	lint color UZ scale	Nominal				null	null	NA	NA	NA	NA	NA	NA	NA
null	CO_358:0000568	g/boll	Numerical		0		null	null	NA	NA	NA	NA	NA	NA	NA
null	CO_358:0000069	mg/kg	Numerical	3	2		null	null	NA	NA	NA	NA	NA	NA	NA
null	CO_358:0000069	mg/kg	Numerical	3	2		null	null	NA	NA	NA	NA	NA	NA	NA
null	CO_358:0000069	mg/kg	Numerical	3	2		null	null	NA	NA	NA	NA	NA	NA	NA
null	CO_358:0000069	mg/kg	Numerical	3	2		null	null	NA	NA	NA	NA	NA	NA	NA
null	CO_358:0000069	mg/kg	Numerical	3	2		null	null	NA	NA	NA	NA	NA	NA	NA
null	CO_358:0000069	mg/kg	Numerical	3	2		null	null	NA	NA	NA	NA	NA	NA	NA
null	CO_358:0000069	mg/kg	Numerical	3	2		null	null	NA	NA	NA	NA	NA	NA	NA
null	CO_358:0000069	mg/kg	Numerical	3	2		null	null	NA	NA	NA	NA	NA	NA	NA
null	CO_358:0000069	mg/kg	Numerical	3	2		null	null	NA	NA	NA	NA	NA	NA	NA
null	CO_358:0000069	mg/kg	Numerical	3	2		null	null	NA	NA	NA	NA	NA	NA	NA
null	CO_358:0000069	mg/kg	Numerical	3	2		null	null	NA	NA	NA	NA	NA	NA	NA
null	CO_358:0000069	mg/kg	Numerical	3	2		null	null	NA	NA	NA	NA	NA	NA	NA
null	CO_358:0000069	mg/kg	Numerical	3	2		null	null	NA	NA	NA	NA	NA	NA	NA
null	CO_358:0000069	mg/kg	Numerical	3	2		null	null	NA	NA	NA	NA	NA	NA	NA
null	CO_358:0000069	mg/kg	Numerical	3	2		null	null	NA	NA	NA	NA	NA	NA	NA
null	CO_358:0000069	mg/kg	Numerical	3	2		null	null	NA	NA	NA	NA	NA	NA	NA
null	CO_358:0000069	mg/kg	Numerical	3	2		null	null	NA	NA	NA	NA	NA	NA	NA
null	CO_358:0000069	mg/kg	Numerical	3	2		null	null	NA	NA	NA	NA	NA	NA	NA
null	CO_358:0000069	mg/kg	Numerical	3	2		null	null	NA	NA	NA	NA	NA	NA	NA
null	CO_358:0000069	mg/kg	Numerical	3	2		null	null	NA	NA	NA	NA	NA	NA	NA
null	CO_358:0000069	mg/kg	Numerical	3	2		null	null	NA	NA	NA	NA	NA	NA	NA
null	CO_358:0000069	mg/kg	Numerical	3	2		null	null	NA	NA	NA	NA	NA	NA	NA
null	CO_358:0000069	mg/kg	Numerical	3	2		null	null	NA	NA	NA	NA	NA	NA	NA
null	CO_358:0000069	mg/kg	Numerical	3	2		null	null	NA	NA	NA	NA	NA	NA	NA
null	CO_358:0000069	mg/kg	Numerical	3	2		null	null	NA	NA	NA	NA	NA	NA	NA
null	CO_358:0000069	mg/kg	Numerical	3	2		null	null	NA	NA	NA	NA	NA	NA	NA
null	CO_358:0000069	mg/kg	Numerical	3	2		null	null	NA	NA	NA	NA	NA	NA	NA
null	CO_358:0000069	mg/kg	Numerical	3	2		null	null	NA	NA	NA	NA	NA	NA	NA
null	CO_358:0000069	mg/kg	Numerical	3	2		null	null	NA	NA	NA	NA	NA	NA	NA
null	CO_358:0000069	mg/kg	Numerical	3	2		null	null	NA	NA	NA	NA	NA	NA	NA
null	CO_358:0000069	mg/kg	Numerical	3	2		null	null	NA	NA	NA	NA	NA	NA	NA
null	CO_358:0000069	mg/kg	Numerical	3	2		null	null	NA	NA	NA	NA	NA	NA	NA
null	CO_358:0000635	petal color CN scale	Nominal				8= purple	null	NA	NA	NA	NA	NA	NA	NA
null	CO_358:0000638	petal color NCGC scale	Nominal				8= pink	9= seg/off type	NA	NA	NA	NA	NA	NA	NA
null	CO_358:0000641	petal color UZ scale	Nominal				null	null	NA	NA	NA	NA	NA	NA	NA
[illegible]	CO_358:0000645	mg/g	Numerical	3	0		null	null	NA	NA	NA	NA	NA	NA	NA
null	CO_358:0000648	petal spot NCGC scale	Ordinal				null	null	NA	NA	NA	NA	NA	NA	NA
null	CO_358:0000651	petal spot UZ scale	Ordinal				null	null	NA	NA	NA	NA	NA	NA	NA
null	CO_358:0000069	mg/kg	Numerical	3	2		null	null	NA	NA	NA	NA	NA	NA	NA
null	CO_358:0000656	m	Numerical		0		null	null	NA	NA	NA	NA	NA	NA	NA
null	CO_358:0000659	PlotHomo UZ Scale	Ordinal				null	null	NA	NA	NA	NA	NA	NA	NA
null	CO_358:0000662	PlotHomo NCGC scale	Text				null	null	NA	NA	NA	NA	NA	NA	NA
null	CO_358:0000665	plot maturity CN scale	Ordinal				null	null	NA	NA	NA	NA	NA	NA	NA
null	CO_358:0000668	Plot maturity NCGC scale	Ordinal				null	null	NA	NA	NA	NA	NA	NA	NA
null	CO_358:0000671	pollen color CN scale	Nominal				null	null	NA	NA	NA	NA	NA	NA	NA
null	CO_358:0000674	pollen color NCGC scale	Nominal				null	null	NA	NA	NA	NA	NA	NA	NA
null	CO_358:0000677	pollen color UZ scale	Nominal				null	null	NA	NA	NA	NA	NA	NA	NA
null	CO_358:0000680	plant shape CN scale	Nominal				null	null	NA	NA	NA	NA	NA	NA	NA
null	CO_358:0000683	plant shape NCGC scale	Nominal				null	null	NA	NA	NA	NA	NA	NA	NA
null	CO_358:0000686	plant shape UZ scale	Nominal				null	null	NA	NA	NA	NA	NA	NA	NA
null	CO_358:0000689	PS sym branch type CN scale	Code				null	null	NA	NA	NA	NA	NA	NA	NA
null	CO_358:0000069	mg/kg	Numerical	3	2		null	null	NA	NA	NA	NA	NA	NA	NA
null	CO_358:0000694	seed oil content CN scale	Ordinal				null	null	NA	NA	NA	NA	NA	NA	NA
null	CO_358:0000069	mg/kg	Numerical	3	2		null	null	NA	NA	NA	NA	NA	NA	NA
null	CO_358:0000699	seed protein content CN scale	Ordinal				null	null	NA	NA	NA	NA	NA	NA	NA
null	CO_358:0000069	mg/kg	Numerical	3	2		null	null	NA	NA	NA	NA	NA	NA	NA
null	CO_358:0000069	mg/kg	Numerical	3	2		null	null	NA	NA	NA	NA	NA	NA	NA
null	CO_358:0000069	mg/kg	Numerical	3	2		null	null	NA	NA	NA	NA	NA	NA	NA
null	CO_358:0000069	mg/kg	Numerical	3	2		null	null	NA	NA	NA	NA	NA	NA	NA
null	CO_358:0000069	mg/kg	Numerical	3	2		null	null	NA	NA	NA	NA	NA	NA	NA
null	CO_358:0000069	mg/kg	Numerical	3	2		null	null	NA	NA	NA	NA	NA	NA	NA
null	CO_358:0000069	mg/kg	Numerical	3	2		null	null	NA	NA	NA	NA	NA	NA	NA
null	CO_358:0000069	mg/kg	Numerical	3	2		null	null	NA	NA	NA	NA	NA	NA	NA
null	CO_358:0000069	mg/kg	Numerical	3	2		null	null	NA	NA	NA	NA	NA	NA	NA
null	CO_358:0000069	mg/kg	Numerical	3	2		null	null	NA	NA	NA	NA	NA	NA	NA
null	CO_358:0000069	mg/kg	Numerical	3	2		null	null	NA	NA	NA	NA	NA	NA	NA
null	CO_358:0000069	mg/kg	Numerical	3	2		null	null	NA	NA	NA	NA	NA	NA	NA
null	CO_358:0000069	mg/kg	Numerical	3	2		null	null	NA	NA	NA	NA	NA	NA	NA
null	CO_358:0000729	fuzz color NCGC scale	Nominal				7= off white	9= seg/off type	NA	NA	NA	NA	NA	NA	NA
null	CO_358:0000732	fuzz color UZ scale	Nominal				null	null	NA	NA	NA	NA	NA	NA	NA
null	CO_358:0000735	fuzz grade CN scale	Ordinal				null	null	NA	NA	NA	NA	NA	NA	NA
null	CO_358:0000738	fuzz grade NCGC scale	Ordinal				null	null	NA	NA	NA	NA	NA	NA	NA
null	CO_358:0000069	mg/kg	Numerical	3	2		null	null	NA	NA	NA	NA	NA	NA	NA
null	CO_358:0000069	mg/kg	Numerical	3	2		null	null	NA	NA	NA	NA	NA	NA	NA
null	CO_358:0000069	mg/kg	Numerical	3	2		null	null	NA	NA	NA	NA	NA	NA	NA
null	CO_358:0000070	mm	Numerical	2	0		null	null	NA	NA	NA	NA	NA	NA	NA
null	CO_358:0000070	mm	Numerical	2	0		null	null	NA	NA	NA	NA	NA	NA	NA
null	CO_358:0000070	mm	Numerical	2	0		null	null	NA	NA	NA	NA	NA	NA	NA
doi: 10.1007/s00438-014-0930-5	CO_358:0000070	mm	Numerical	2	0		null	null	NA	NA	NA	NA	NA	NA	NA
null	CO_358:0000070	mm	Numerical	2	0		null	null	NA	NA	NA	NA	NA	NA	NA
null	CO_358:0000070	mm	Numerical	2	0		null	null	NA	NA	NA	NA	NA	NA	NA
null	CO_358:0000070	mm	Numerical	2	0		null	null	NA	NA	NA	NA	NA	NA	NA
[illegible]	CO_358:0000071	mm2/mm3	Numerical	0	0		null	null	NA	NA	NA	NA	NA	NA	NA
[illegible]	CO_358:0000071	mm2/mm3	Numerical	0	0		null	null	NA	NA	NA	NA	NA	NA	NA
null	CO_358:0000072	number	Numerical	0	0		null	null	NA	NA	NA	NA	NA	NA	NA
doi: 10.1111/pbi.12722	CO_358:0000072	number	Numerical	0	0		null	null	NA	NA	NA	NA	NA	NA	NA
null	CO_358:0000072	number	Numerical	0	0		null	null	NA	NA	NA	NA	NA	NA	NA
doi: 10.1007/s10681-014-1150-0	CO_358:0000072	number	Numerical	0	0		null	null	NA	NA	NA	NA	NA	NA	NA
null	CO_358:0000072	number	Numerical	0	0		null	null	NA	NA	NA	NA	NA	NA	NA
null	CO_358:0000072	number	Numerical	0	0		null	null	NA	NA	NA	NA	NA	NA	NA
null	CO_358:0000072	number	Numerical	0	0		null	null	NA	NA	NA	NA	NA	NA	NA
null	CO_358:0000790	VWR CN scale	Ordinal				null	null	NA	NA	NA	NA	NA	NA	NA
null	CO_358:0000072	number	Numerical	0	0		null	null	NA	NA	NA	NA	NA	NA	NA
null	CO_358:0000072	number	Numerical	0	0		null	null	NA	NA	NA	NA	NA	NA	NA
null	CO_358:0000073	rating	Numerical	1	1	7	null	null	NA	NA	NA	NA	NA	NA	NA
null	CO_358:0000073	rating	Numerical	1	1	7	null	null	NA	NA	NA	NA	NA	NA	NA
null	CO_358:0000074	rating	Numerical	1	1	5	null	null	NA	NA	NA	NA	NA	NA	NA
null	CO_358:0000074	rating	Numerical	1	1	5	null	null	NA	NA	NA	NA	NA	NA	NA
null	CO_358:0000075	ratio	Numerical		0		null	null	NA	NA	NA	NA	NA	NA	NA
null	CO_358:0000075	ratio	Numerical		0		null	null	NA	NA	NA	NA	NA	NA	NA
null	CO_358:0000075	ratio	Numerical		0		null	null	NA	NA	NA	NA	NA	NA	NA
NCVT	CO_358:0000813	mN/tex	Numerical	2	0		null	null	NA	NA	NA	NA	NA	NA	NA
null	CO_358:0000075	ratio	Numerical		0		null	null	NA	NA	NA	NA	NA	NA	NA
null	CO_358:0000819	read	Numerical	1	0		null	null	NA	NA	NA	NA	NA	NA	NA
null	CO_358:0000075	ratio	Numerical		0		null	null	NA	NA	NA	NA	NA	NA	NA
null	CO_358:0000076	unit	Numerical		0		null	null	NA	NA	NA	NA	NA	NA	NA
null	CO_358:0000827	?m	Numerical		0		null	null	NA	NA	NA	NA	NA	NA	NA
null	CO_358:0000076	unit	Numerical		0		null	null	NA	NA	NA	NA	NA	NA	NA
null	CO_356:4000003	Calendar date	Time				null	NA	NA	NA	NA	NA	NA	NA	NA
null	CO_356:4000004	day/Chasselas	Duration				null	NA	NA	NA	NA	NA	NA	NA	NA
null	CO_356:4000004	day/Chasselas	Duration				null	NA	NA	NA	NA	NA	NA	NA	NA
null	CO_356:4000005	week/Chasselas	Duration				null	NA	NA	NA	NA	NA	NA	NA	NA
null	CO_356:4000005	week/Chasselas	Duration				null	NA	NA	NA	NA	NA	NA	NA	NA
null	CO_356:4000003	Calendar date	Time				null	NA	NA	NA	NA	NA	NA	NA	NA
null	CO_356:4000003	Calendar date	Time				null	NA	NA	NA	NA	NA	NA	NA	NA
null	CO_356:4000001	%v/v	Numerical				null	NA	NA	NA	NA	NA	NA	NA	NA
[illegible]	CO_356:4000028	S1_9_by2	Nominal		1	9	1-3-5-7-9	NA	NA	NA	NA	NA	NA	NA	NA
[illegible]	CO_356:4000031	S1_6	Nominal		1	6	null	NA	NA	NA	NA	NA	NA	NA	NA
[illegible]	CO_356:4000023	S1_3	Nominal		1	3	1/02/2003	NA	NA	NA	NA	NA	NA	NA	NA
[illegible]	CO_356:4000023	S1_3	Nominal		1	3	1/02/2003	NA	NA	NA	NA	NA	NA	NA	NA
[illegible]	CO_356:4000023	S1_3	Nominal		1	3	1/02/2003	NA	NA	NA	NA	NA	NA	NA	NA
[illegible]	CO_356:4000022	S1_2	Nominal		1	2	1-Feb	NA	NA	NA	NA	NA	NA	NA	NA
[illegible]	CO_356:4000028	S1_9_by2	Nominal		1	9	1-3-5-7-9	NA	NA	NA	NA	NA	NA	NA	NA
[illegible]	CO_356:4000023	S1_3	Nominal		1	3	1/02/2003	NA	NA	NA	NA	NA	NA	NA	NA
[illegible]	CO_356:4000028	S1_9_by2	Nominal		1	9	1-3-5-7-9	NA	NA	NA	NA	NA	NA	NA	NA
[illegible]	CO_356:4000028	S1_9_by2	Nominal		1	9	1-3-5-7-9	NA	NA	NA	NA	NA	NA	NA	NA
[illegible]	CO_356:4000028	S1_9_by2	Nominal		1	9	1-3-5-7-9	NA	NA	NA	NA	NA	NA	NA	NA
[illegible]	CO_356:4000033	S3_5_7	Nominal		3	7	3/05/2007	NA	NA	NA	NA	NA	NA	NA	NA
[illegible]	CO_356:4000025	S1_5	Nominal		1	5	1-2-3-4-5	NA	NA	NA	NA	NA	NA	NA	NA
[illegible]	CO_356:4000021	S1_10	Nominal		1	10	1-Oct	NA	NA	NA	NA	NA	NA	NA	NA
[illegible]	CO_356:4000028	S1_9_by2	Nominal		1	9	1-3-5-7-9	NA	NA	NA	NA	NA	NA	NA	NA
[illegible]	CO_356:4000032	S1_9	Nominal		1	9	1-Sep	NA	NA	NA	NA	NA	NA	NA	NA
[illegible]	CO_356:4000028	S1_9_by2	Nominal		1	9	1-3-5-7-9	NA	NA	NA	NA	NA	NA	NA	NA
null	CO_356:4000022	S1_2	Nominal		1	2	1-Feb	NA	NA	NA	NA	NA	NA	NA	NA
null	CO_356:4000022	S1_2	Nominal		1	2	1-Feb	NA	NA	NA	NA	NA	NA	NA	NA
[illegible]	CO_356:4000028	S1_9_by2	Nominal		1	9	1-3-5-7-9	NA	NA	NA	NA	NA	NA	NA	NA
[illegible]	CO_356:4000028	S1_9_by2	Nominal		1	9	1-3-5-7-9	NA	NA	NA	NA	NA	NA	NA	NA
[illegible]	CO_356:4000028	S1_9_by2	Nominal		1	9	1-3-5-7-9	NA	NA	NA	NA	NA	NA	NA	NA
[illegible]	[illegible]	[illegible]	[illegible]	[illegible]	[illegible]	[illegible]	[illegible]	[illegible]	[illegible]	[illegible]	[illegible]	[illegible]	[illegible]	[illegible]	[illegible]
