# Supplementary material for: Challenges for FAIR-compliant description and comparison of crop phenotype data with standardized controlled vocabularies
Source: Database (Oxford). 2021 May 15;2021:baab028. doi: 10.1093/database/baab028 (PMC8122365; doi:10.1093/database/baab028)
Supplement: baab028_Supp [file baab028_supp.zip › SupplementalTable2.pdf]

Count of Trait.name	Banana	Barley	Brachiaria	Brassica	Cassava	Castorbean	Chickpea	Common Bean	Cotton	Cowpea	Faba bean	Groundnut	Lentil	Maize	Mungbean	Oat	Pearl millet	Pigeonpea	Potato	Rice	Sorghum	Soybean	Sugar beet	Sugar Kelp	Sweetpotato	Vitis	Wheat	Yam	Grand Total
Plant height	1	1	1		1	1	1	1	1	1	1		1	1	1	1	1	1		1	1	1					1		20
Harvest index		1					1	1		1		1	1					1		1					1		1		10
Drought Tolerance		1	1			1	1		1				1		1							1					1		9
Seed weight				1			1	1		1		1	1		1			1				1							9
Flowering time		1				1				1	1	1			1						1	1							8
Growth habit		1					1	1		1	1	1	1		1														8
Leaf chlorophyll content							1		1		1	1			1			1				1					1		8
Maturity time		1				1				1				1	1						1	1					1		8
Salinity tolerance		1				1	1		1				1		1				1								1		8
Seed shape						1	1	1		1	1	1			1														8
Grain protein content		1								1			1	1		1					1						1		7
Plant stand		1		1			1							1	1			1									1		7
Seed protein content							1		1		1	1			1			1				1							7
Seed yield							1		1		1	1			1			1				1							7
Stem Color					1	1			1					1				1									1	1	7
stem diameter									1	1	1				1		1										1	1	7
Cold tolerance		1					1						1		1					1							1		6
Emergence time		1				1					1	1			1												1		6
Flower color							1	1		1								1				1			1				6
Grain yield										1				1		1				1	1						1		6
Leaf color					1				1			1		1							1							1	6
leaf length			1			1								1						1							1	1	6
Leaf shape						1			1			1						1	1									1	6
Lodging incidence											1			1	1	1				1							1		6
Peduncle length		1								1					1					1	1						1		6
Pod length							1	1			1				1			1				1							6
Root length			1		1		1					1	1							1									6
Canopy height							1			1		1								1		1							5
Grain weight														1			1			1	1						1		5
leaf width			1											1						1							1	1	5
Petiole length					1	1									1										1			1	5
Plant vigour		1								1		1						1									1		5
Seed color					1	1	1					1					1												5
Seed length						1		1		1	1				1														5
Seed width						1		1		1	1				1														5
Chlorophyll content			1							1			1	1															4
Cooking time							1	1		1			1																4
Grain color														1		1					1						1		4
Heat tolerance													1		1				1								1		4
leaf area	1					1						1														1			4
Plant vigor														1					1		1							1	4
Pod number				1			1								1							1							4
Seed oil content						1			1			1										1							4
Stomatal conductance								1					1	1						1									4
Amylose Content					1															1					1				3
Aphid damage										1				1													1		3
Aphid resistance									1				1		1														3
awn length		1																		1							1		3
Biomass yield				1			1																		1				3
Canopy temperature								1						1													1		3
Days to 50% flowering							1		1				1																3
Flag leaf length		1															1			1									3
Fusarium wilt resistance							1		1				1																3
Grain Iron content		1												1													1		3
grain length														1						1							1		3
Grain moisture content		1																			1						1		3
Grain number		1												1													1		3
Grain test weight		1												1													1		3
grain thickness														1						1							1		3
grain width														1						1							1		3
Grain Zinc content		1												1													1		3
Heading time		1																			1						1		3
Internode length											1				1													1	3
Leaf carbon isotope discrimination								1				1															1		3
Leaf dry weight	1					1	1																						3
Leaf number	1					1																1							3
Leaf weight					1					1		1																	3
maturity																				1				1		1			3
Nodulation											1				1							1							3
panicle length																	1			1	1								3
Plant growth habit			1																1								1		3
Plant lodging incidence		1		1																	1								3
Pod borer resistance							1						1		1														3
Pod curvature								1		1					1														3
Pod shattering				1							1											1							3
Pods per plant							1	1										1											3
Protein Content					1																			1	1				3
Root length density			1				1						1																3
Seed Calcium content				1							1				1														3
Seed Copper content				1							1				1														3
Seed Iron content				1							1				1														3
Seed Magnesium content				1							1				1														3
Seed Manganese content				1							1				1														3
Seed Potassium content				1							1				1														3
Seed Selenium content				1							1				1														3
Seed Sodium content				1							1				1														3
Seed Zinc content				1							1				1														3
Seedling vigor														1			1				1								3
Seedling vigour											1				1			1											3
Seeds per pod							1	1										1											3
Specific leaf area							1					1															1		3
stem borer damage														1			1			1									3
Stem pigmentation		1						1				1																	3
Time to maturity							1						1															1	3
Transpiration efficiency	1						1					1																	3
Winter survival													1			1											1		3
Acid soil tolerance															1												1		2
Agronomic score																1											1		2
Aluminium tolerance			1			1																							2
anther colour	1																			1									2
Anthesis time														1													1		2
Ascochyta blight resistance							1						1																2
Ash Content					1																			1					2
awn color																				1							1		2
awn presence		1																		1									2
bird damage																				1							1		2
Boron tolerance		1																									1		2
Branch length											1				1														2
Branch number															1													1	2
Branching habit					1							1																	2
Branching pattern						1							1																2
Bruchid resistance													1		1														2
Canopy temperature depression								1																			1		2
Canopy width												1	1																2
Collar rot resistance							1						1																2
Date of harvest	1																1												2
Days to first flower							1											1											2
Drought susceptibility		1									1																		2
Effective tiller number		1																			1								2
Endosperm color														1							1								2
Fiber content																						1			1				2
First pod position											1				1														2
Flag leaf area																				1							1		2
Flag leaf width		1																		1									2
Flower number			1																			1							2
Flowering					1																					1			2
Flowering period duration											1				1														2
Frost damage						1																					1		2
Fusarium wilt incidence																		1									1		2
Glume color																					1						1		2
glume opening																				1	1								2
Grain carbon isotope discrimination								1																			1		2
Grain hardness																					1						1		2
Grain lysine content														1													1		2
Grain Nitrogen content		1												1															2
Grain number per spike		1																									1		2
Grain plumpness																1											1		2
Grain size																					1						1		2
Grain starch content		1												1															2
Grain sucrose content										1				1															2
Grain yield per plant										1										1									2
Growth pattern										1					1														2
Growth stage								1																			1		2
Hail damage				1																							1		2

Harvest time													1										1							2
High temperature susceptibility		1										1																		2
High temperature tolerance						1	1																						2
Insect damage																						1					1		2
Iron concentration									1	1																		2
Iron Content					1																				1				2
Leaf area index								1																		1		2
Leaf colour										1					1												2
Leaf intercellular carbon dioxide concentration																				1						1		2
Leaf Lobe Number						1																		1				2
Leaf Potassium content				1								1																2
Leaf pubescence															1			1										2
Leaf rolling severity														1												1		2
Leaf size									1			1																2
Leaf Sodium content				1								1																2
Leaf Vein Color					1																1							2
Main stem diameter						1																1						2
Maturity group																		1				1						2
Net blotch severity		1																								1		2
Node number															1											1		2
Nodule number										1												1						2
Number of clusters																		1							1			2
Number of emerged plants																			1								1	2
Number of harvested hills																	1				1							2
Number of harvested plants																	1		1									2
Number of leaves																									1		1	2
Number of plants harvested	1																							1				2
Number of plants planted	1																							1				2
Number of seedling hills																	1				1							2
Oil yield				1		1																						2
Panicle density																				1	1							2
Panicle exertion																	1				1							2
panicle threshability																				1	1							2
pericarp colour		1																		1								2
Photoperiod response								1							1													2
Photosynthetic efficiency			1					1																				2
Plant aspect			1											1														2
plant dry weight	1					1																							2
Plant early vigour		1																								1		2
plant fresh weight	1					1																							2
Plant leafiness			1												1													2
Plant number											1										1							2
Plant pigmentation							1			1																			2
Plant pubescence							1						1																2
Plant shape					1				1																				2
Plant Type																								1			1	2
Plant uniformity														1					1									2
Plant width							1	1																				2
Plants Harvested														1								1						2
Ploidy					1														1									2
Pod color																		1				1						2
Pod colour										1	1																		2
Pod constriction												1			1													2
Pod pubescence															1			1										2
Pod size												1						1										2
Pod thickness											1				1													2
Pod weight							1						1																2
Pod width								1		1																			2
Pod yield										1		1																2
Pods per plot								1		1																			2
Powdery mildew incidence																1										1		2
Powdery Mildew susceptibility		1										1																		2
Reproductive period																						1				1		2
Root biomass			1									1																2
Root diameter												1								1								2
Root dry weight	1						1																						2
Root knot nematode resistance							1												1									2
Root rot incidence						1										1												2
Root surface area							1													1								2
Root volume							1													1								2
Rust severity												1		1															2
Salinity susceptibility		1										1																		2
Seed diameter							1						1																2
seed germination																		1		1								2
Seed index									1																		1	2
Seed linolenic acid content									1													1						2
Seed moisture content																		1				1						2
Seed number				1											1													2
Seed oleic acid content									1													1						2
Seed palmitic acid content									1													1						2
Seed Phosphorus content				1							1																		2
Seed quality																						1	1					2
Seed shattering											1				1													2
Seed size								1				1																2
Seed stearic acid content									1													1						2
Seed width length ratio											1				1													2
Seed yield per plant							1						1																2
Seedling number					1																1							2
Seeds per plant								1										1										2
Senescence														1					1									2
Sepal Color					1																				1				2
Sorghum downy mildew incidence														1							1							2
Spike density		1																								1		2
Spike length		1																								1		2
Spike number		1																								1		2
Spike type		1																								1		2
Spot blotch severity		1																								1		2
Stem dry weight							1													1								2
Stem height					1																						1	2
Stem length																						1					1		2
Stem potassium content												1										1							2
Stem rust severity																1										1		2
Stem sodium content												1										1							2
Stigma Color					1															1								2
stigma exsertion									1											1								2
Storage root cortex thickness																								1				2
Storage Root Shape					1																				1				2
Storage Root Size					1																				1				2
Storage root stalk																								1				2
Straw yield																				1						1		2
Striga plant number														1							1							2
Terminal leaflet length										1					1													2
Terminal leaflet shape										1					1													2
Terminal leaflet width										1					1													2
Thousand grain weight		1														1													2
Tiller number		1																								1		2
Time to flowering							1						1																2
Tuber appearance																			1								1	2
Tuber dormancy period																			1								1	2
Tuber dry matter content																			1								1	2
Tuber shape																			1								1	2
Tuber size																			1								1	2
Virus Incidence										1																		1	2
Virus Severity										1																		1	2
Zinc concentration									1	1																		2
Zinc Content					1																				1				2
100 grain weight																	1											1
2.5 span length									1																				1
3rd order branching density										1																			1
50 span length									1																				1
50% sprout emergence time																											1	1
Abaxial Leaf Vein Pigmentation																								1				1
abortion pattern																				1								1
Aboveground biomass at maturity																										1		1
Aboveground biomass yield dry weight bas		1																										1
Aboveground biomass yield fresh weight ba		1																										1
Abscisic Acid Content					1																								1
Accession ID	1																											1
Acid detergent fiber content			1																									1
Acid soil susceptibility											1																		1
adventitious root growth angle										1																			1
Aeneolamia reducta adult damage			1																									1
Aeneolamia reducta adult number			1																									1
Aeneolamia reducta antibiosis			1																									1
Aeneolamia reducta egg number			1																									1

Aeneolamia reducta nymph damage			1																						1
Aeneolamia varia adult damage			1																						1
Aeneolamia varia adult number			1																						1
Aeneolamia varia antibiosis			1																						1
Aeneolamia varia egg number			1																						1
Aeneolamia varia nymph damage			1																						1
Aerenchyma area																	1								1
Aerenchyma diameter																	1								1
Aerenchyma number																	1								1
Aflatoxin content											1														1
Agronomic appreciation score														1											1
Agronomic water efficiency						1																			1
Al content																				1					1
Alcidodes sp. damage																					1				1
Alcohol																						1			1
Alectra								1																	1
Alectra - Date of First Alectra Emergence								1																	1
Alectra- Alectra infection								1																	1
Alectra- Height of Alectra								1																	1
Aleurone color											1														1
alkali injury																	1								1
ALPERPLT								1																	1
Alternaria blight disease susceptibility															1										1
Alternaria blight resistance					1																				1
Alternaria leaf blight plant response																							1		1
Aluminum tolerance																							1		1
Amylopectin Content				1																					1
Amylose Amylopectin Ratio				1																					1
Angular Leaf Spot in field						1																			1
Angular Leaf Spot in greenhouse						1																			1
Angular Leaf Spot on pods in field						1																			1
Annual crop cycle proportion	1																								1
Ant damage																							1		1
Anther Anthocyanin coloration											1														1
Anther Color				1																					1
Anther extrusion																							1		1
anther length																	1								1
Anther pigmentation																1									1
Anthesis silking interval											1														1
Anthesis thermal time																							1		1
anthocyanin below apiculus																	1								1
Anthocyanin content																					1				1
Anthocyanin Pigmentation				1																					1
Anthracnose damage								1																	1
Anthracnose leaf blight incidence											1														1
Anthracnose on leaves in field						1																			1
Anthracnose on leaves in greenhouse						1																			1
Anthracnose on pods in field						1																			1
Anthracnose resistance										1															1
Apex shape of the terminal leaflets																1									1
Aphid incidence								1																	1
Aphid severity								1																	1
Aphid susceptibility									1																1
Aphids susceptibility		1																							1
Aphis						1																			1
Apical Branching				1																					1
Apical leaflet shape																1									1
Apical leaflet size																1									1
Apical primary branches number per plant					1																				1
Apical Pubescence				1																					1
Apical secondary branches number per plant					1																				1
apiculus anthocyanin																	1								1
apiculus color																	1								1
apiculus shape																	1								1
Appearance of flower																					1				1
Appearance of hearbarium																					1				1
Appearance of leaves																					1				1
Appearance of plant																					1				1
Appearance of roots																					1				1
Appearance of seeds																					1				1
Arealometer A							1																		1
Arealometer AH							1																		1
Army worm damage											1														1
Armyworm damage																							1		1
As content																				1					1
Ascochyta blight on leaves in field						1																			1
Ascochyta blight on leaves in greenhouse						1																			1
Ascochyta blight on pods in field						1																			1
Ascochyta blight severity									1																1
Ascochyta blight susceptibility									1																1
Ascorbic Acid				1																					1
Asparagine content																					1				1
Aspergillus ear rot severity											1														1
Aspergillus flavus ear rot severity											1														1
Attachment of testa								1																	1
auricle anthocyanin																	1								1
auricle color																	1								1
Auricle pigmentation		1																							1
Avenanthramide content													1												1
Average actual annual yield	1																								1
Average commercial root weight																					1				1
Average finger circumference	1																								1
Average finger external length	1																								1
Average finger internal length	1																								1
Average finger lateral diameter	1																								1
Average finger peel thickness	1																								1
Average finger weight	1																								1
Average fruit pulp lateral diamete	1																								1
Average hand weight	1																								1
Average number of fingers in han	1																								1
Average of marketable tuber weight																1									1
Average of tuber weight																1									1
Average percentage damage to th	1																								1
Average potential annual yield	1																								1
Average tuber weight																								1	1
Average weigth of big size tuber																								1	1
Awn colour		1																							1
awn distribution																	1								1
Awn texture		1																							1
awn thickness																	1								1
Awn tip pigmentation presence		1																							1
Awn type		1																							1
awning																	1								1
B content																				1					1
B crypto activity				1																					1
Bacteria pustule								1																	1
Bacterial blight (X. vignicola) severity								1																	1
bacterial blight damage																	1								1
Bacterial disease																			1						1
Bacterial leaf blight incidence																							1		1
Bacterial leaf blight severity																							1		1
Bacterial leaf spot resistance												1													1
bacterial leaf streak damage																	1								1
Bacterial leaf streak severity																							1		1
Bacterial leaf stripe severity											1														1
Bacterial Pustule																		1							1
Bacterial stalk rot incidence											1														1
Bacterial wilt resistance																1									1
bakanae disease																	1								1
Banana weevil adult average bod	1																								1
Banana weevil adult average bod	1																								1
Banana weevil adult average hea	1																								1
Banana weevil adult body length	1																								1
Banana weevil adult body weight	1																								1
Banana weevil adult head capsule	1																								1
Banana weevil larvae average boc	1																								1
Banana weevil larvae average boc	1																								1
Banana weevil larvae average hea	1																								1
Banana weevil larvae body length	1																								1
Banana weevil larvae body weigh	1																								1
Banana weevil larvae head capsul	1																								1
Banded leaf and sheath blight severity											1														1
Barley Helminthosporium leaf stripe incidence																							1		1
Barley leaf rust coefficient of infection																							1		1
Barley leaf rust incidence																							1		1

Barley leaf rust severity																								1		1
Barley scald incidence																								1		1
Barley scald severity																								1		1
Barley stripe mosaic incidence																								1		1
Barley yellow dwarf incidence																								1		1
Barley yellow dwarf virus incidence														1												1
Barley yellow dwarf virus severity														1												1
Barley Yellow Dwarf Virus susceptibility		1																								1
basal leaf sheath anthocyanin																	1									1
basal leaf sheath color																	1									1
Basal primary branches number per plant						1																				1
basal root growth angle									1																	1
Basal root growth angle in field							1																			1
Basal root growth angle in greenhouse							1																			1
Basal root length							1																			1
Basal root whorl number							1																			1
Basal roots number							1																			1
Basal secondary branches number per plant						1																				1
Basal tiller number															1											1
Base shape of the terminal leaflets																1										1
Basic Vegetative Phase																		1								1
Bean Common Mosaic Necrosis Virus							1																			1
Bean Golden Yellow Mosaic Virus in field							1																			1
Bean Golden Yellow Mosaic Virus in greenhouse							1																			1
Bean Stem Maggot							1																			1
Beet armyworm																			1							1
Beginning of flowering time (BBCH 61)				1																						1
Beginning of pod development time (BBCH 71)				1																						1
Beginning of pod maturity time (BBCH 81)				1																						1
Bemesia tabaci nymphs black					1																					1
Bemesia tabaci nymphs severity					1																					1
Bemesia tabaci nymphs yellow					1																					1
Berry hilum																							1			1
Berry pedicel																							1			1
Berry skin																							1			1
Berry: bloom																							1			1
Berry: color of skin																							1			1
Berry: ease of detachment from pedicel																							1			1
Berry: firmness of flesh																							1			1
Berry: formation of seeds																							1			1
Berry: intensity of the anthocyanin coloration of flesh																							1			1
Berry: juiciness of flesh																							1			1
Berry: length																							1			1
Berry: length of seeds																							1			1
Berry: particularity of flavor																							1			1
Berry: shape																							1			1
Berry: transversal ridges on dorsal side of seeds																							1			1
Berry: uniformity of color of skin																							1			1
Berry: uniformity of size																							1			1
Berry: weight of seeds																							1			1
Berry: width																							1			1
Beta carotene					1																					1
Beta Carotenoid Retention					1																					1
Beta-carotene content																						1				1
Beta-glucan content														1												1
Big size tuber length																									1	1
Big size tuber weight per plant																									1	1
Big size tuber width																									1	1
Big size tuber yield adjusted																									1	1
Big size tubers per plant																									1	1
Big size tubers per plot																									1	1
Big size tubers weight per plot																									1	1
Big size yam yield																									1	1
Biological yield per area												1														1
Biological yield per plant												1														1
Biomass											1															1
Biomass at Maturity									1																	1
Biomass harvest time			1																							1
Biomass production rate																								1		1
Biomass yield per plant						1																				1
Biotic attack annotation				1																						1
Black bundle incidence													1													1
Black leaf streak disease developr	1																									1
Black leaf streak disease evolutior	1																									1
Black leaf streak disease incubatic	1																									1
Black leaf streak disease severity	1																									1
Black leaf streak disease severity	1																									1
Black point incidence																								1		1
Black Rust coefficient of infection		1																								1
Black Rust plant response		1																								1
Black Rust severity		1																								1
Black Rust severity and response		1																								1
Black Rust susceptibility		1																								1
Blackeye cowpea mosaic virus									1																	1
Blackeye cowpea mosaic virus severity									1																	1
Blade length																					1					1
Blade width																					1					1
Block ID	1																									1
Blotches colour at the petiole bas	1																									1
Blotches relative area at petiole b	1																									1
boll color								1																		1
boll gossypol glands								1																		1
boll nectaries								1																		1
boll number								1																		1
boll open rate								1																		1
boll pitting								1																		1
boll point								1																		1
boll shape								1																		1
boll size								1																		1
Bolting																				1						1
Booting initiation thermal time																								1		1
Booting thermal time																								1		1
Booting time																								1		1
Boron deficiency												1														1
Boron toxicity tolerance												1														1
Botrytis grey mold resistance						1																				1
Bract apex shape	1																									1
Bract behaviour before falling	1																									1
bract color								1																		1
Bract imbrication at apex of male	1																									1
bract nectaries								1																		1
bract shape								1																		1
bract teeth number								1																		1
bract teeth size								1																		1
bract type								1																		1
Branch height					1																					1
Branch number per plant										1																1
Branching Angle					1																					1
Branching density									1																	1
branching density 10-15									1																	1
branching density 5-10									1																	1
Branching Levels					1																					1
Bread crumb structure																								1		1
Bread loaf volume																								1		1
Breakage incidence														1												1
Bristle density															1											1
Bristle length															1											1
Bristle presence															1											1
Broken groats														1												1
Brown blotch									1																	1
brown planthopper damage																	1									1
brown rice protein content																	1									1
Brown rice rate																	1									1
Brown Rust coefficient of infection		1																								1
Brown Rust plant response		1																								1
Brown Rust severity		1																								1
Brown Rust severity and response		1																								1
Brown Rust susceptibility		1																								1
brown spot damage																	1									1
Brown spot severity													1													1
Brown stripe downy mildew incidence													1													1
Bruchid damage							1																			1
Bud necrosis disease incidence											1															1
Budbreak																							1			1
Bulbil shape																									1	1

Bulbil weight																							1	1
Bulbil yield																							1	1
Bunch at harvest photograph	1																							1
Bunch length																					1			1
Bunch maturity stage at harvest	1																							1
Bunch position	1																							1
Bunch shape	1																							1
Bunch weight	1																							1
Bunch: density																					1			1
Bunch: length of peduncle of primary bunch																					1			1
Bunch: lignification of peduncle																					1			1
Bunch: number of wings of the primary bunch																					1			1
Bunch: shape																					1			1
Bunch: width																					1			1
Busseola damage												1												1
Busseola incidence												1												1
C content																			1					1
Ca content																			1					1
calcium concentration								1																1
Calcium content																				1				1
Calculated number of fingers in b	1																							1
Calyx color															1									1
Calyx colour													1											1
Calyx lobe length									1															1
Canopy architecture																							1	1
Canopy biomass at R8							1																	1
Canopy green normalized difference vegetation index																						1		1
Canopy light interception																						1		1
Canopy normalized difference vegetation index																						1		1
Canopy normalized pheophytinization index																						1		1
Canopy normalized water index 1																						1		1
Canopy normalized water index 2																						1		1
Canopy normalized water index 3																						1		1
Canopy normalized water index 4																						1		1
Canopy photochemical reflectance index																						1		1
Canopy ratio analysis of reflectance spectra carotenoid																						1		1
Canopy ratio analysis of reflectance spectra chlorophyll a																						1		1
Canopy ratio analysis of reflectance spectra chlorophyll b																						1		1
Canopy red normalized difference vegetation index																						1		1
Canopy simple ratio																						1		1
Canopy stay green																						1		1
Canopy structural independent pigment index																						1		1
canopy type								1																1
Canopy water index																						1		1
Capacity for re-growth									1															1
Capsule shattering incidence					1																			1
Capsule spineness					1																			1
Capsule wall hardness					1																			1
Capsule yield					1																			1
Carbohydrate / fiber content																			1					1
Carbon isotope discrimination						1																		1
Carotenoid content				1																				1
Caruncle Color				1																				1
caryopsis length																1								1
caryopsis shape																1								1
caryopsis width																1								1
case worm damage																1								1
Cassava anthracnose disease incidence				1																				1
Cassava anthracnose disease severity				1																				1
Cassava bacterial blight incidence				1																				1
Cassava bacterial blight severity				1																				1
Cassava brown streak disease leaf incidence				1																				1
Cassava brown streak disease leaf severity				1																				1
Cassava brown streak disease root incidence				1																				1
Cassava brown streak disease root severity				1																				1
Cassava green mite incidence				1																				1
Cassava green mite severity				1																				1
Cassava Mealy Bug Incidence				1																				1
Cassava Mealy Bug Severity				1																				1
Cassava mosaic disease incidence				1																				1
Cassava mosaic disease severity				1																				1
Cause of damage	1																							1
Cause of death	1																							1
Cd content																			1					1
Central Leaf Shape				1																				1
Cercospora									1															1
Cercospora Leaf spot																		1						1
Cercospora leaf spot resistance													1											1
chalkiness of endosperm																1								1
Charcoal rot incidence												1												1
Chilo damage												1												1
Chips color															1									1
Chips oil absorption rate															1									1
Chlorosis																		1						1
Chlorotic leaf biomass		1																						1
Chocolate spot severity										1														1
Chocolate spot susceptibility										1														1
chromosome number																1								1
Cis 9 activity				1																				1
Cl content																			1					1
Climatic incidents annotation			1																					1
Cluster number													1											1
Co content																			1					1
Cob glumes Anthocyanin coloration												1												1
Cob glumes color												1												1
Cob sucrose content												1												1
Cohesiveness of pounded yam																							1	1
Cold stress resistance																		1						1
coleoptile anthocyanin																1								1
Coleoptile color																	1							1
Coleoptile length																						1		1
coletotrichum resistance								1																1
collar anthocyanin																1								1
collar color																1								1
Collar rot incidence											1													1
Colletotrichum blight resistance						1																		1
Color of boiled roots																				1				1
Color Of Boiled Tuberous Roots				1																				1
color of dry pod									1															1
Color of leave																				1				1
Color of stigma																				1				1
Color of style																				1				1
Colour line along edge of petiole	1																							1
Colour of bract external face	1																							1
Colour of bract internal face	1																							1
Column ID	1																							1
Common Bacterial Blight on leaves in field							1																	1
Common Bacterial Blight on leaves in greenhouse							1																	1
Common Bacterial Blight on pods in field							1																	1
Common bunt spike incidence																						1		1
Common root rot incidence																						1		1
Common rust severity												1												1
Common smut incidence												1												1
Common smut severity												1												1
Compound tepal main colour	1																							1
Concentration of bound terpenols																					1			1
Concentration of bound terpenols in the pulp																					1			1
Concentration of bound terpenols in the skins																					1			1
Concentration of free and bound terpenols																					1			1
Concentration of free and bound terpenols in the pulp																					1			1
Concentration of free and bound terpenols in the skins																					1			1
Concentration of free terpenols																					1			1
Concentration of free terpenols in the pulp																					1			1
Concentration of free terpenols in the skins																					1			1
Concentrations of free and bound alpha-terpineol																					1			1
Concentrations of free and bound citronellol																					1			1
Concentrations of free and bound geraniol																					1			1
Concentrations of free and bound linalool																					1			1
Concentrations of free and bound nerol																					1			1
Consistency of boiled storage root																				1				1
Cookie spread factor																						1		1
Cooking quality of dhal														1										1
Cooking quality of seed														1										1
copper concentration								1																1
Corm on tuber																							1	1

Corm separability from tuber																									1	1
Corm size																									1	1
Corm type																									1	1
Corn stunt incidence											1															1
Corn stunt spiroplasma severity											1															1
Corolla colour												1														1
Corolla shape																1										1
cotton bollworm resistance							1																			1
Cotyledon colour										1																1
Cowpea aphid-borne mosaic virus severity								1																		1
Cr content																					1					1
Crazy roots presence																									1	1
Crazy top downy mildew incidence											1															1
Critical photoperiod																		1								1
Crop cycle ID	1																									1
Crop establishment																				1						1
Crop ground cover																								1		1
Crop growth rate																								1		1
Crop problems annotation			1																							1
Crossing date																									1	1
Crown rust infection type													1													1
Crown rust severity													1													1
Crude protein content		1																								1
Cu content																					1					1
culm angle																	1									1
Culm area																	1									1
Culm degree of deformation																	1									1
Culm diameter - 1st internode																	1									1
Culm diameter - 2nd internode																	1									1
culm internode anthocyanin																	1									1
culm internode color																	1									1
culm internode underlying colour																	1									1
culm length																	1									1
Culm major diameter - 2nd internode																	1									1
Culm minor diameter - 2nd internode																	1									1
culm node anthocyanin																	1									1
culm node underlying colour																	1									1
culm number																	1									1
Culm partial porosity																	1									1
Culm porosity																	1									1
Culm specific porosity																	1									1
culm strength																	1									1
Culm total porosity																	1									1
Culm wall area																	1									1
Culm wall thickness																	1									1
Cultivar group									1																	1
Curvularia leaf spot severity											1															1
Cyanidin content																						1				1
Cyst nematode damage																								1		1
Cyst nematode resistance					1																					1
Cytoplasmic male sterility																		1								1
daily rate of water loss	1																									1
Damage description	1																									1
Damage photograph	1																									1
Damage to periphery of peeled cc	1																									1
Date of anthesis														1												1
Date of bloom														1												1
Date of damage	1																									1
Date of data collection	1																									1
Date of death	1																									1
Date of emergence														1												1
Date of first external disease sym	1																									1
Date of first youngest leaf spotte	1																									1
Date of flowering	1																									1
Date of heading														1												1
Date of physiological maturity														1												1
Date of planting	1																									1
Date of recognising off-type	1																									1
Date of shooting	1																									1
Date of sowing														1												1
Date of Striga emergence								1																		1
Date of tagged cigar leaf unrolling	1																									1
Date of tagged leaf presenting bla	1																									1
Date of tagged leaf presenting bla	1																									1
Date of weevil damage to corm a	1																									1
days flowering to boll open							1																			1
Days to 50 % flowering															1											1
Days to 50% anthesis				1																						1
days to 50% boll opening							1																			1
Days to 50% branching				1																						1
days to 50% emergence							1																			1
Days to 75 % maturity															1											1
Days to first anthesis				1																						1
Days to first flowering										1																1
Days to first inflorescence				1																						1
days to flowering						1																				1
days to physiological maturity						1																				1
Dead leaf biomass at R8						1																				1
Dead plant number								1																		1
Dead plant proportion								1																		1
Death density	1																									1
Death photograph	1																									1
Death proportion	1																									1
Decorticated grain weight																		1								1
Deep of eyes of roots																						1				1
Defoliation																			1							1
Dehiscence type						1																				1
Deoxynivalenol concentration													1													1
Diabrotica								1																		1
Diabrotica damage											1															1
Diameter of internodes																							1			1
Diatraea damage											1															1
Dietary fiber content													1													1
Disc Color				1																						1
DISEASES								1																		1
Distance of internal discolouratio	1																									1
Distance of internal discolouratio	1																									1
Distribution of Secondary Flesh color																						1				1
Distribution of secondary sprout color																1										1
Distribution of secondary tuber flesh color																1										1
Distribution of secondary tuber skin color																1										1
Diverse damages																				1						1
DO 420																							1			1
DO 520																							1			1
DO 620																							1			1
Dots on seed coat					1																					1
Double ridge time																								1		1
Dough alveograph extensibility index																								1		1
Dough alveograph P/G																								1		1
Dough alveograph P/L																								1		1
Dough alveograph value G																								1		1
Dough alveograph value L																								1		1
Dough alveograph value P																								1		1
Dough alveograph value W																								1		1
Dough Mixograph mixing time																								1		1
Dough mixograph torque																								1		1
Dough mixograph type																								1		1
Downy mildew																			1							1
Downy mildew infected hill number														1												1
Downy mildew infected plant number														1												1
Downy mildew severity											1															1
Drought induced senescence								1																		1
drought injury																	1									1
Drought intensity index						1																				1
drought recovery																	1									1
Drought stress																				1						1
Drought stress susceptibility															1											1
Drought susceptibility index						1																				1
Drought/Heat stress																				1						1
Dry fodder weight								1																		1
Dry matter content																					1					1
Dry matter digestibility								1																		1
Dry matter yield																	1									1
dry plant growth	1																									1
Dry root rot resistance					1																					1

Dry weight of flag leaf and flag leaf-1																1								1
Dry weight per shoot																					1			1
Dwarf bunt incidence																						1		1
Dwarfism	1																							1
DYSTRGEM									1															1
Ear aspect													1											1
Ear bad husk cover incidence													1											1
Ear damage													1											1
Ear diameter													1											1
Ear growth													1											1
Ear growth rate													1											1
Ear harvested number													1											1
Ear height													1											1
Ear husk cover													1											1
Ear leaf chlorophyll content													1											1
Ear leaf length													1											1
Ear leaf senescence time													1											1
Ear leaf weight													1											1
Ear length													1											1
Ear number													1											1
Ear position													1											1
Ear rot incidence													1											1
Ear shape													1											1
Ear shelling ratio													1											1
Ear weight													1											1
Ear width													1											1
Early growth vigour												1												1
Early leaf spot severity											1													1
Early pod development time (BBCH 73)				1																				1
Early shattering																		1						1
Earworm damage													1											1
Earworm incidence													1											1
Ease of Harvest					1																			1
Ease Of Peeling Root Cortex					1																			1
Elongated internode number																1								1
Emerging cotyledon color							1																	1
Empty panicle number																	1							1
End date and time of data collect	1																							1
End of flowering time (BBCH 69)				1																				1
Endosperm hardness													1											1
Endosperm Lysine content													1											1
Endosperm protein content													1											1
Endosperm texture																	1							1
Endosperm Tryptophan content													1											1
endosperm type																1								1
Equality of sepal length																				1				1
Ergot infection proportion														1										1
Established plants																							1	1
Evapotranspiration							1																	1
Exposed leaf area																					1			1
Extent of internal discolouration i	1																							1
Extent of internal discolouration i	1																							1
Eye colour									1															1
Eye pattern									1															1
Eyespot plant response																						1		1
Faba bean necrotic Yellow virus susceptibility										1														1
Faba bean yellow mosaic virus susceptibility										1														1
false smut																1								1
Farmer appreciation score														1										1
Farmer preference score														1										1
Fe content																			1					1
Female flower number					1																			1
Female flowering intensity																							1	1
Female Stamenoids					1																			1
fertility restoration																1								1
fiber color grade								1																1
Fiber cooked																				1				1
fiber density								1																1
fiber elongation								1																1
fiber fineness								1																1
fiber immature content								1																1
fiber length								1																1
fiber length by number								1																1
fiber length by weight								1																1
fiber mature content								1																1
fiber nep count								1																1
fiber nep size								1																1
fiber number per seed								1																1
fiber perimeter								1																1
fiber quality score								1																1
fiber spin consistency index								1																1
fiber strength								1																1
fiber uniformity								1																1
Fibre Content					1																			1
Fibrousness of pounded yam																							1	1
filament color								1																1
Filled double pod number						1																		1
Filled grain weight																1								1
Filled seed proportion			1																					1
Filled spikelet density																1								1
Filled spikelet proportion																1								1
Filled spikelets per panicle																1								1
Final plant stand											1													1
Finger circumference	1																							1
Finger external length	1																							1
Finger internal length	1																							1
Finger lateral diameter	1																							1
Finger peel thickness	1																							1
Finger weight	1																							1
First flower																		1						1
First flowers time (BBCH 60)				1																				1
first fruit branch position								1																1
first fruit branch position by node								1																1
First inter-node time (BBCH 31)				1																				1
First node detectable time																						1		1
First sprout emergence time																							1	1
flag leaf angle																1								1
Flag leaf attitude		1																						1
Flag leaf chlorophyll content																1								1
Flag leaf dimeter														1										1
Flag leaf dry weight																1								1
Flag leaf lamina length																						1		1
Flag leaf lamina width																						1		1
Flag leaf ligulation time																	1							1
Flag leaf relative size																1								1
Flag leaf senescence																						1		1
Flag leaf specific area																1								1
Flag leaf temperature																1								1
Flag leaf weight																	1							1
Flag leaf-1 area																1								1
Flag leaf-1 dry weight																1								1
Flag leaf-1 length																1								1
Flag leaf-1 specific leaf area																1								1
Flag leaf-1 width																1								1
Flag smut plant response																						1		1
Flesh Color (anthocyanins)																				1				1
Flesh Color (carotenoids)																				1				1
Flesh color after cooking															1									1
Flesh darkening after cooking															1									1
Flooding tolerance																		1						1
Floral abscision									1															1
Flour color																						1		1
Flour falling number																						1		1
Flour gluten content																						1		1
Flour gluten type																						1		1
Flour protein content																						1		1
Flour SDS sedimentation																						1		1
Flour SDS sedimentation index																						1		1
Flour yellowness																						1		1
Flour yield																						1		1
Floury leaf spot							1																	1
Flower crescent color											1													1
Flower form																		1						1
Flower length									1															1
Flower number per pod										1														1

Flower size																			1				1
Flower standard color						1																	1
Flower standard petal color										1													1
Flower thrip damage								1															1
Flower wings color						1																	1
Flower: sexual organs																				1			1
flowering date														1									1
Flowering degree													1										1
Flowering duration					1																		1
Flowering habit																			1				1
Flowering intensity																						1	1
Flowering pattern												1											1
Flowering pigment pattern								1															1
Flowering time (BBCH 62-65)			1																				1
Fodder biomass at Flowering								1															1
Fodder Yield																1							1
Foliage senescence time																						1	1
Foliage total yield																			1				1
Foliar anthracnose response															1								1
Foliar anthracnose severity															1								1
Foliar disease development																					1		1
Foliar thrip damage								1															1
Forage dry matter																					1		1
French fries color													1										1
Fresh biomass at flowering								1															1
Fresh fodder weight								1															1
fresh plant growth	1																						1
Fresh pod								1															1
Fresh seed dormancy										1													1
Fresh Shoot Weight				1																			1
Fresh weight																		1					1
Frog Skin Disease				1																			1
Frogeye leafspot																1							1
Frost susceptibility									1														1
Fructose content																			1				1
Fruit apex point	1																						1
fruit branch internode length							1																1
fruit branch node number							1																1
Fruit color													1										1
Fruit developed number																						1	1
Fruit Exocarp Texture				1																			1
Fruit index																						1	1
Fruit length	1																						1
Fruit pedicel length	1																						1
Fruit pulp colour	1																						1
Fruit pulp lateral diameter	1																						1
Fruit set number																						1	1
Fruit set presence				1																			1
Fruit shape													1										1
Fruit shape of curve	1																						1
Fruit smashed pulp aroma/smell	1																						1
Fruit smashed pulp colour	1																						1
Fruit smashed pulp smoothness-n	1																						1
Fruit smashed pulp taste	1																						1
Fruit smashed pulp texture	1																						1
Fruit unsmashed pulp colour	1																						1
Fruit unsmashed pulp smoothnes:	1																						1
Fruit unsmashed pulp taste	1																						1
Fruit unsmashed pulp texture	1																						1
Fruiting efficiency																					1		1
fruiting type							1																1
Fruits harvested number																						1	1
Fufu Content				1																			1
functional leaf number	1																						1
Fungal disease																	1						1
Fungal disease presence											1												1
Fusarium ear rot severity											1												1
Fusarium graminearum ear rot severity											1												1
Fusarium graminearum incidence																					1		1
Fusarium graminearum plant response																					1		1
Fusarium graminearum severity																					1		1
Fusarium graminearum stalk rot incidence											1												1
Fusarium head blight AUDPC																					1		1
Fusarium head blight DON content																					1		1
Fusarium head blight grain incidence																					1		1
Fusarium head blight incidence												1											1
Fusarium Head Blight severity		1																					1
Fusarium head blight spike incidence																					1		1
Fusarium head blight spike response																					1		1
Fusarium head blight spike weight																					1		1
Fusarium head blight spikelet incidence																					1		1
Fusarium leaf blotch incidence																					1		1
Fusarium leaf blotch severity																					1		1
Fusarium moniliforme ear rot severity											1												1
Fusarium moniliforme stalk rot incidence											1												1
Fusarium moniliforme stalk rot severity											1												1
Fusarium solani in field						1																	1
Fusarium solani in greenhouse						1																	1
Fusarium stalk rot incidence											1												1
Fusarium wilt photograph	1																						1
Fusarium wilt Race 3 damage								1															1
Fusarium wilt Race 4 damage								1															1
Fusion of pedicels	1																						1
Ga content																		1					1
gall midge damage														1									1
Gari Content				1																			1
Gari fibre				1																			1
Gari sampled weight				1																			1
Gari shaft weight				1																			1
Gari starch				1																			1
Gari taste				1																			1
Gari weight after drying				1																			1
Gari weight after grinding				1																			1
gel consistency														1									1
gelatinization temperature														1									1
General disease severity										1													1
General Outline of the Leaf																			1				1
Genotype ID	1																						1
Genotype name	1																						1
Genotype purpose	1																						1
Geometric mean of 100 seed weight						1																	1
Geometric mean of days to physiological maturity						1																	1
Geometric mean of seed yield						1																	1
Germination time																1							1
gin turnout							1																1
Glucose content																			1				1
Glume adhesion															1								1
Glume awnedness															1								1
Glume glaucousness																					1		1
Glume length																					1		1
Glume pubescence density																					1		1
Glume pubescence length																					1		1
Glume width																					1		1
Glutenin Glu-A1 composition																					1		1
Glutenin Glu-A3 composition																					1		1
Glutenin Glu-B1 composition																					1		1
Glutenin Glu-B3 composition																					1		1
Glutenin Glu-D1 composition																					1		1
Glutenin Glu-D3 composition																					1		1
Glyphosate susceptibility at flowering stage									1														1
Glyphosate susceptibility at vegetative stage									1														1
gossypol free coutent							1																1
gossypol minus coutent							1																1
gossypol plus coutent							1																1
Grain 15N natural abundance						1																	1
Grain abscisic acid content											1												1
Grain amylopectin content																					1		1
Grain amylose content															1								1
Grain anthracnose severity															1								1
Grain appearance																					1		1
Grain ash content											1												1
Grain Beta Glucan content		1																					1
Grain colour		1																					1
Grain covering															1								1
Grain crease width		1																					1

Grain decortication yield																			1							1
Grain density																			1							1
Grain Deoxynivalenol content		1																							1
Grain desirability																			1							1
grain discoloration																		1								1
Grain dry matter basis yield																			1							1
Grain dry matter content																			1							1
grain elongation																		1								1
Grain end use type																								1	1
Grain filling index							1																		1
Grain filling period																								1	1
Grain filling period duration		1																							1
Grain filling rate																		1								1
Grain germination																								1	1
Grain germination capacity		1																							1
Grain germinative energy		1																							1
Grain glucose content												1													1
Grain harvest index																			1							1
Grain hullness		1																							1
Grain husk content		1																							1
Grain length to width ratio																		1								1
Grain lipid content																			1							1
Grain lustre																			1							1
Grain moisture												1													1
Grain mold severity																			1							1
Grain number per panicle																			1							1
Grain number per spikelet																								1	1
Grain oil content												1													1
Grain Phosphorous content																								1	1
Grain plumpness fraction 2.2 mm		1																							1
Grain plumpness fraction 2.5 mm		1																							1
Grain plumpness fraction 2.8 mm		1																							1
Grain proline content												1													1
Grain provitaminA content												1													1
Grain retention																								1	1
Grain rotation																			1							1
Grain roundness																			1							1
Grain row arrangement												1													1
Grain row number												1													1
Grain screenings																								1	1
Grain shape		1																							1
Grain shape in dorsal view																			1							1
Grain shape in profile view																			1							1
Grain standardized yield																			1							1
Grain subcoat presence																			1							1
Grain sugar content												1													1
Grain texture												1													1
Grain Tryptophan content												1													1
Grain type												1													1
Grain upper surface shape												1													1
Grain viability												1													1
Grain vitreousness																								1	1
Grain weight loss by maize weevil												1													1
Grain weight per panicle																			1							1
Grain weight per plant																1									1
Grain weight per plot																1									1
Grain weight per spike																								1	1
Grain yellow berry incidence																								1	1
Grain yield adjusted weight basis		1																							1
Grain yield at harvest																			1							1
Grain yield dry weight basis		1																							1
Grain yield factor																								1	1
Grain yield fresh weight basis		1																							1
Grain yield per plot								1																	1
Grain yield production rate																								1	1
grassy stunt 1 disease																		1								1
grassy stunt 2 disease																		1								1
Gray leaf spot severity												1													1
Gray mold resistance					1																				1
Gray mold severity					1																				1
Green leaf area			1																						1
Green leaf biomass			1																						1
Green Leaf Biomass proportion			1																						1
Green leaf number																		1								1
green leafhopper damage																		1								1
Green leaves number																			1							1
Green pod yield									1																1
Groat color														1											1
Groat content														1											1
Groat oil content														1											1
Groat protein content														1											1
Ground colour of seed testa											1														1
Ground Cover																						1			1
Growing season																						1			1
Growth class																								1	1
Growth of axillary shoots																							1		1
Growth rate																					1				1
Growth type class		1																							1
Growth vigor						1																			1
GRPODKGH								1																	1
H content																					1				1
Hairiness				1																					1
Hairiness on mainstem										1															1
Hairiness on mature leaflet										1															1
Half-filled grain weight																		1								1
Half-filled spikelet densty																		1								1
Half-filled spikelet weight per area																		1								1
Half-filled spikelets per panicle																		1								1
Half-filled spikelets proportion																		1								1
Halo blight on leaves in field							1																		1
Halo blight on leaves in greenhouse							1																		1
Halo blight on pods in field							1																		1
hand rank	1																								1
Hand rank of 2nd-most distal han	1																								1
Hand weight	1																								1
Harvest date								1																	1
Harvest density	1																								1
Harvest proportion	1																								1
Harvested leaf weight								1																	1
Head milled rice rate																		1								1
Head miner damage															1										1
head rice																		1								1
Head smut incidence												1													1
Head smut severity												1													1
HEAD WEIGHT								1																	1
Heading date														1											1
Heading thermal time																								1	1
heat injury																		1								1
Heat stress																				1					1
Heat stress resistance																			1						1
height increase of pseudostem	1																								1
Height of lowest pods																			1						1
Height of the lowest pod											1														1
Helminthosporiose disease presence																			1							1
Helminthosporium species incidence																								1	1
Helminthosporium species legacy																								1	1
Helminthosporium species severity																								1	1
Herbicide damage																								1	1
Herbicide sensitivity																			1						1
Herbicide tolerance											1														1
Hessian fly damage																								1	1
Hessian Fly susceptibility		1																							1
High soil moisture susceptibility		1																							1
High temperature stress susceptibility																1									1
Hill number per plot															1										1
Hilum color																			1						1
hoja blanca disease																		1								1
Hundred seed dry weight					1																				1
Hundred seed weight									1																1
Hypocotyl colour													1												1
Hypocotyl length								1																	1
Hypocotyl pigmentation							1																		1
I content																					1				1
Imizathapyr susceptibility at flowering stage									1																1
Imizathapyr susceptibility at vegetative stage									1																1

Immature Leaf Color																							1				1
Immature pod colour															1												1
Immature pod pigmentation										1																	1
In content																						1					1
In vitro dry matter digestibility			1																								1
In vitro dry matter digestibility of neutral detergent			1																								1
increase of leaf area	1																										1
Index of non-spotted leaves	1																										1
Infertile spike number																									1		1
Infertile tiller number																									1		1
Inflorescence initiation time																										1	1
Inflorescence number																	1										1
Inflorescence type																										1	1
Inflorescence: insertion of 1st inflorescence																								1			1
Initial Vigor					1																						1
In-season aboveground biomass																									1		1
Insect pest damage												1															1
INSECTS										1																	1
Intensity of Predominant Skin color																							1				1
Iron (Fe) deficiency													1														1
iron toxicity injury																		1									1
Java downy mildew incidence														1													1
K content																						1					1
Karnal bunt incidence																									1		1
keel anthocyanin																		1									1
Keeping quality of storage roots																							1				1
kernel smut																		1									1
kneeing ability																		1									1
Lafun Content					1																						1
Large grain borer damage														1													1
Late blight AUDPC																	1										1
Late blight relative AUDPC																	1										1
Late blight resistance																	1										1
Late Blight severity																	1										1
Late blight susceptibility																	1										1
Late leaf spot severity												1															1
Late Shattering Score																				1							1
Late wilt incidence														1													1
Lateral leaflet shape																	1										1
Lateral root density				1																							1
Lateral root number				1																							1
Lateral root shape																				1							1
Lateral root size																				1							1
Lateral spikelet fertility		1																									1
Lateral spikelet presence		1																									1
Latex Production in Storage Roots																							1				1
Leaf																					1						1
Leaf 15N natural abundance								1																			1
Leaf abaxial surface color																	1										1
Leaf abaxial surface trichome density																	1										1
Leaf abscisic acid content														1													1
Leaf adaxial surface color																	1										1
Leaf adaxial surface trichome density																	1										1
Leaf Aluminium content				1																							1
leaf angle																		1									1
Leaf apex shape																										1	1
Leaf area in field								1																			1
Leaf area in greenhouse								1																			1
leaf area to plant area ratio	1																										1
Leaf Arsenic content				1																							1
Leaf ash content														1													1
Leaf Balium content				1																							1
Leaf beetle damage incidence																										1	1
Leaf beetle damage severity																										1	1
Leaf biomass			1																								1
Leaf biomass at R8								1																			1
Leaf biomass dry matter basis yield																			1								1
Leaf biomass dry matter content																			1								1
Leaf biomass moisture content																			1								1
Leaf biomass yield at harvest																			1								1
leaf blade anthocyanin																		1									1
leaf blade color																		1									1
leaf blade greenness																		1									1
leaf blade pubescence																		1									1
Leaf blast damage																		1									1
Leaf blotch severity																1											1
Leaf Boron content				1																							1
Leaf Cadmium content				1																							1
Leaf Caesium content				1																							1
Leaf Calcium content				1																							1
Leaf carotenoid content											1																1
Leaf Chlorophyll a content											1																1
Leaf Chlorophyll b content											1																1
leaf chlorosis	1																										1
Leaf Chromium content				1																							1
Leaf Copper content				1																							1
Leaf density																										1	1
Leaf dry weight per plant																		1									1
Leaf electron transport rate																									1		1
Leaf erectness														1													1
leaf fresh weight	1																										1
Leaf glaucousness																									1		1
leaf gossypol glands									1																		1
Leaf hairiness																										1	1
Leaf harvest										1																	1
Leaf intercellular to ambient carbon dioxide ratio																									1		1
Leaf Iron content				1																							1
Leaf lamina dry weight						1																					1
Leaf lamina leaf width						1																					1
Leaf lamina length						1																					1
Leaf lamina partitioning index																									1		1
Leaf Lead content				1																							1
Leaf Life span					1																						1
Leaf light and carbon dioxide-saturated net photosynthesis																									1		1
Leaf light-saturated net photosynthesis																									1		1
Leaf lobe length					1																						1
Leaf Lobe Position					1																						1
Leaf Lobe Type																							1				1
Leaf lobe width					1																						1
Leaf Lobes Number					1																						1
Leaf loss								1																			1
Leaf Magnesium content				1																							1
Leaf Manganese content				1																							1
leaf margin pubescence																		1									1
Leaf marking										1																	1
Leaf maximal fluorescence intensity																									1		1
Leaf maximum efficiency of PSII photochemistry																									1		1
Leaf midrib color																			1								1
Leaf miner fly resistance																	1										1
Leaf miner resistance							1																				1
Leaf minimal fluorescence intensity																									1		1
Leaf Molybdenum content				1																							1
leaf nectaries									1																		1
Leaf net photosynthesis rate																									1		1
Leaf Nickel content				1																							1
Leaf persistence								1																			1
Leaf Phosphorus content				1																							1
Leaf position																										1	1
Leaf proline content														1													1
Leaf protein content										1																	1
Leaf pubescence density																									1		1
Leaf pubescence length																									1		1
Leaf quantum yield of carbon dioxide fixation																									1		1
Leaf quantum yield of photosystem II																									1		1
leaf relative water content	1																										1
Leaf Retention					1																						1
Leaf rolling incidence																									1		1
Leaf Rubidium content				1																							1
Leaf rust coefficient of infection																									1		1
Leaf rust incidence																									1		1
Leaf rust infestation										1																	1
Leaf rust legacy																									1		1
Leaf rust notes																									1		1
Leaf rust plant response																									1		1
Leaf rust seedling response																									1		1

Leaf rust severity																										1	1
Leaf scab infestation										1																	1
leaf scald damage																			1								1
Leaf Scald severity		1																									1
Leaf Scald susceptibility		1																									1
Leaf scar distance					1																						1
Leaf scar height					1																						1
Leaf scar number					1																						1
Leaf Scar Prominence					1																						1
Leaf Selenium content				1																							1
leaf senescence																			1								1
Leaf sheath partitioning index																										1	1
Leaf sheath thickness																			1								1
Leaf Silver content				1																							1
Leaf spectral reflectance 550 nm																										1	1
Leaf spectral reflectance 675 nm																										1	1
Leaf spectral reflectance 750/550 nm																										1	1
Leaf spectral reflectance 750/700 nm																										1	1
Leaf spot infestation										1																	1
Leaf spot photograph	1																										1
Leaf steady-state fluorescence																										1	1
Leaf stem ratio										1																	1
Leaf stomatal conductance																										1	1
Leaf stomatal frequency																										1	1
Leaf stomatal resistance																										1	1
Leaf stomatal size																										1	1
Leaf Stripe incidence		1																									1
Leaf Stripe susceptibility		1																									1
Leaf Strontium content				1																							1
Leaf sucrose content														1													1
Leaf Sulphur content				1																							1
Leaf temperature																			1								1
Leaf texture										1																	1
Leaf thickness																										1	1
Leaf tier susceptibility																	1										1
Leaf tip necrosis incidence																										1	1
Leaf Titanium content				1																							1
Leaf to stem weight ratio																				1							1
leaf to whole plant mass ratio	1																										1
Leaf total number																			1								1
Leaf transpiration rate																										1	1
Leaf trichome length															1												1
Leaf type							1																				1
Leaf Uranium content				1																							1
Leaf Vanadium content				1																							1
leaf variable fluorescence																										1	1
Leaf water index ratio											1																1
Leaf width to length ratio			1																								1
Leaf Zinc content				1																							1
leaffolder damage																			1								1
Leafhopper								1																			1
Leafhopper (Empoasca) damage										1																	1
Leaflet margin							1																				1
Leaflet shape																					1						1
leaves formed per pseudostem he	1																										1
Leaves per plant																								1			1
lemma and palea color																			1								1
lemma and palea color ornementation																			1								1
lemma and palea pubescence																			1								1
Lemma colour		1																									1
lemma phenol reaction																			1								1
Lemma surface type		1																									1
Length of internodes																									1		1
Length of pods										1																	1
Length of stolons																		1									1
Length to Diameter Ratio of roots																								1			1
Li content																							1				1
ligule color																			1								1
ligule length																			1								1
ligule margin shape																			1								1
ligule pubescence																			1								1
ligule shape																			1								1
lint color									1																		1
lint index									1																		1
lint percent									1																		1
lint visible foreign matter									1																		1
lint yield									1																		1
Lipid content																							1				1
LLS severity on leaf				1																							1
Lobe colour of tip of compound te	1																										1
locule number per boll									1																		1
Lodging																					1						1
Lodging resistance											1																1
Lodging severity																1											1
Lodging susceptibility													1														1
Loose smut incidence																										1	1
Low temperature stress susceptibility																	1										1
Low temperature susceptibility		1																									1
Lygus bug damage										1																	1
M. incognita root knot nematode										1																	1
M. javanica root knot nematode										1																	1
Macrophomina in field								1																			1
Macrophomina in greenhouse								1																			1
Macrophomina Severity										1																	1
magnesium concentration									1																		1
Magnesium content																								1			1
Main inflorescence flower number																		1									1
Main stem dry weight						1																					1
Main stem length						1																					1
Main stem node number						1																					1
Main Stem pigmentation																		1									1
Main underlying colour of pseudc	1																										1
Maize bushy stunt severity														1													1
Maize dwarf mosaic virus incidence														1													1
Maize dwarf mosaic virus severity														1													1
Maize lethal necrosis severity														1													1
Maize rayado fino virus incidence														1													1
Maize rough dwarf virus incidence														1													1
Maize streak virus incidence														1													1
Maize streak virus severity														1													1
Maize stripe virus incidence														1													1
Maize weevil incidence														1													1
Maize weevil number in Grain														1													1
Mal de rio cuarto virus incidence														1													1
Male bud length	1																										1
Male bud shape	1																										1
Male bud shoulder	1																										1
Male fertility																						1					1
Male flower length						1																					1
Male flower number					1																						1
Male rachis appearance	1																										1
Male rachis position	1																										1
Male rachis type	1																										1
Male Sterile					1																						1
male sterility group																			1								1
Malic acid concentration																									1		1
Malt Alpha Amylase activity		1																									1
Malt Beta Glucan content		1																									1
Malt diastatic power		1																									1
Malt friability		1																									1
Malt hot water extract		1																									1
Malt Kolbach index		1																									1
Malt yield		1																									1
Maltose content																								1			1
manganese concentration									1																		1
Margin behaviour on petiole cana	1																										1
Marketable root number					1																						1
Marketable root weight					1																						1
Marketable root yield																								1			1
Marketable tuber weight																		1									1
Marketable tuber weight per plant																		1									1
Marketable tuber yield adjusted																		1									1
Marketable tuber yield no adjusted																		1									1
Marking on standard petal												1															1
Maruca damage										1																	1

Mat ID	1																									1
Mature Leaf Color																						1				1
Mature Leaf Size																						1				1
Mature leaf: angle between N1 and N2 (Code Nos OIV 601 and OIV 602) measured at the first ramification																							1			1
Mature leaf: angle between N2 and N3 (Code Nos OIV 601 and OIV 602) measured at the first ramification																							1			1
Mature leaf: angle between N3 and N4 (Code Nos OIV 601 and OIV 602) measured at the first ramification																							1			1
Mature leaf: angle between N3 and the tangent between petiole point 610																							1			1
Mature leaf: area of anthocyanin coloration of main veins on lower side of blade																							1			1
Mature leaf: area of anthocyanin coloration of main veins on upper side of blade																							1			1
Mature leaf: blistering of upper side of blade																							1			1
Mature leaf: color of the upper side of blade																							1			1
Mature leaf: degree of opening / overlapping of petiole sinus																							1			1
Mature leaf: degree of opening / overlapping of upper lateral sinus																							1			1
Mature leaf: density of erect hairs between the main veins on lower side of blade																							1			1
Mature leaf: density of erect hairs on main veins on lower side of blade																							1			1
Mature leaf: density of erect hairs on petiole																							1			1
Mature leaf: density of prostrate hairs between the main veins on lower side of blade																							1			1
Mature leaf: density of prostrate hairs on main veins on lower side of blade																							1			1
Mature leaf: density of prostrate hairs on petiole																							1			1
Mature leaf: depth of upper lateral sinuses																							1			1
Mature leaf: erect hairs on main veins on upper side of blade																							1			1
Mature leaf: goffering of blade																							1			1
Mature leaf: length between the tooth tip of N2 and the tooth tip of the first secondary vein of N2																							1			1
Mature leaf: length of petiole compared to length of middle vein																							1			1
Mature leaf: length of teeth compared with their width																							1			1
Mature leaf: length of tooth N2																							1			1
Mature leaf: length of tooth N4																							1			1
Mature leaf: length of vein N1																							1			1
Mature leaf: length of vein N2																							1			1
Mature leaf: length of vein N3																							1			1
Mature leaf: length of vein N4																							1			1
Mature leaf: length of vein N5																							1			1
Mature leaf: length petiole sinus to lower lateral leaf sinus																							1			1
Mature leaf: length petiole sinus to upper lateral leaf sinus																							1			1
Mature leaf: number of lobes																							1			1
Mature leaf: number of teeth between the tooth tip of N2 and the tooth tip of the first secondary vein of N2 including the limits																							1			1
Mature leaf: opening/overlapping of petiole sinus																							1			1
Mature leaf: petiole sinus base limited by veins																							1			1
Mature leaf: profile of blade in cross section																							1			1
Mature leaf: prostrate hairs on main veins on upper side of blade																							1			1
Mature leaf: shape of base of petiole sinus																							1			1
Mature leaf: shape of base of upper lateral sinuses																							1			1
Mature leaf: shape of blade																							1			1
Mature leaf: shape of teeth																							1			1
Mature leaf: size of blade																							1			1
Mature leaf: size of teeth in relation to blade size																							1			1
Mature leaf: teeth in the petiole sinus																							1			1
Mature leaf: teeth in the upper lateral sinuses																							1			1
Mature leaf: undulation of blade between main and lateral veins																							1			1
Mature leaf: width of tooth N2																							1			1
Mature leaf: width of tooth N4																							1			1
Mature pod color							1																			1
Mature pod colour													1													1
Mature spike colour		1																								1
Maturity date														1												1
Maydis leaf blight severity												1														1
Mealybug tuber infestation incidence																									1	1
Mealybug tuber infestation severity																									1	1
Mean root diameter							1																			1
Medium size tuber length																									1	1
Medium size tuber weight per plant																									1	1
Medium size tuber width																									1	1
Medium size tubers per plant																									1	1
Medium size tubers per plot																									1	1
Medium size tubers weight per plot																									1	1
Metribuzin susceptibility at flowering stage										1																1
Metribuzin susceptibility at vegetative stage										1																1
Mg content																					1					1
Micro nutrient content											1															1
micronaire								1																		1
Midge incidence																			1							1
Milled rice rate																		1								1
Millipede damage																						1				1
Mineral Deficiencies																				1						1
Mn content																					1					1
Mo content																					1					1
Moisture Content					1																					1
monopodia branch number								1																		1
Monthly leaf emission rate	1																									1
Multiple cob incidence												1														1
Multiple finger weight	1																									1
Multiple hand weight	1																									1
Multiple number of fingers in han	1																									1
must specific pH																							1			1
Must yield																							1			1
N content																					1					1
Na content																					1					1
narrow brown leaf spot damage																		1								1
Nematode lifecycle stage	1																									1
Nematode reproduction factor (n	1																									1
Nematode species	1																									1
Nematodes susceptibility																1										1
Net blotch incidence																								1		1
Net Blotch susceptibility		1																								1
Neutral detergent fiber content			1																							1
Ni content																					1					1
No method name found		1																								1
No of plants with Sclerotinia (out of six)				1																						1
No of plants with Sclerotinia (per plot)				1																						1
No. of fertile pods per plant						1																				1
Nodal tiller number															1											1
Node number in main stem										1																1
Node number of the first tendril above the cotyledon																							1			1
Nodes per vine																						1				1
Nodule weight									1																	1
Nodules number in field							1																			1
Nodules weight in field							1																			1
Non marketable root number					1																					1
Non marketable root weight					1																					1
Non-bearing tiller number		1																								1
Non-harvest density	1																									1
Non-harvest proportion	1																									1
Non-marketable tuber number																	1									1
Non-marketable tuber weight																	1									1
Normalized difference vegetation index												1														1
number of adventitious roots									1																	1
Number of banana weevil adults	1																									1
Number of banana weevil larvae	1																									1
number of basal region roots									1																	1
Number of Bemesia tabaci adult					1																					1
Number of Bemesia tabaci nymphs					1																					1
Number of buds left after pruning																							1			1
Number of bulbil																									1	1
Number of canes																							1			1
Number of commercial storage roots																						1				1
Number of effective nodules in bush bean							1																			1
Number of effective nodules in climbing bean							1																			1
Number of empty or missing panicles															1											1
Number of fertile branches				1																						1
Number of fingers in 2nd hand	1																									1
Number of fingers in 2nd-most di	1																									1
Number of fingers in 3rd hand	1																									1
Number of fingers in hand	1																									1
Number of fingers in outer whorl	1																									1
Number of fingers measured	1																									1
Number of flowering stalks					1																					1
Number of flowers per peduncle						1																				1
Number of forks					1																					1
Number of fruits					1																					1
Number of fruits on mid-hand of	1																									1
Number of fruitset					1																					1
Number of functional leaves	1																									1
Number of functional roots	1																									1
Number of hands in bunch	1																									1
Number of hands measured	1																									1

Number of hands on whole bunc	1																					1
Number of harvested panicles											1											1
Number of hills						1																1
Number of inflorescences (All)																			1			1
Number of inflorescences on non-count position																			1			1
Number of inflorescences on primary shoots																			1			1
Number of inflorescences on secundary shoots																			1			1
Number of inflorescences on spurs																			1			1
Number of inflorescences per primary shoot																			1			1
Number of inflorescences per secundary shoot																			1			1
Number of inflorescences per shoot																			1			1
Number of inflorescences per shoot (all kinds of shoots)																			1			1
Number of inflorescences per shoot on non-count positions																			1			1
Number of inflorescences per shoot on spurs																			1			1
Number of internodes to first branching																					1	1
Number of leaflets per leaf				1																		1
Number of leaves with black leaf	1																					1
Number of leaves with black leaf	1																					1
Number of leaves with black leaf	1																					1
Number of leaves with black leaf	1																					1
Number of leaves with black leaf	1																					1
Number of leaves with black leaf	1																					1
Number of leaves with black leaf	1																					1
Number of Lenticels																		1				1
Number of maiden suckers	1																					1
Number of main branches						1																1
Number of nodes						1																1
Number of non-commercial storage roots																		1				1
Number of non-count positions shoots																			1			1
Number of off-type panicles											1											1
Number of peduncles per plant									1													1
Number of peeper suckers	1																					1
Number of planted stakes			1																			1
Number of plants dead	1																					1
Number of plants established																		1				1
Number of plants not harvested	1																					1
Number of plants per effective plot area					1																	1
Number of plants per m?					1																	1
Number of plants per m2																			1			1
Number of plants with phoma leaf spots		1																				1
Number of plants with storage roots																		1				1
Number of pods per peduncle									1													1
Number of pods per plant									1													1
Number of primary branches												1										1
Number of primary shoots																			1			1
Number of productive branches												1										1
Number of racemes						1																1
Number of rotten stem			1																			1
Number of secondary branches												1										1
Number of secundary shoots																			1			1
Number of seedling plants											1											1
Number of seeds																1						1
Number of seeds per berry																			1			1
Number of seeds per pod									1													1
Number of seeds sown			1																			1
Number of sepal veins																		1				1
Number of shoots on spurs																			1			1
Number of spotted leaves	1																					1
Number of sprouted buds																			1			1
Number of stakes			1																			1
Number of standing leaves	1																					1
Number of Stolons													1									1
Number of storage root damages																		1				1
Number of Striga						1																1
Number of suckers	1																					1
Number of sword suckers	1																					1
number of tap laterals						1																1
Number of terminal branches with inflorescence			1																			1
Number of tubers planted													1									1
Number of water suckers	1																					1
Number of marketable tubers													1									1
Number of marketable tubers per plant													1									1
O content																	1					1
Off-type description	1																					1
Off-type photograph	1																					1
Orobanche index							1															1
Orobanche resistance									1													1
Orobanche shoot number							1															1
Orobanche shoot weight							1															1
Osmotic adjustment										1												1
Osmotic potential										1												1
Outcrossing extent														1								1
Ovary Color			1																			1
Overall appearance of cooked sample																		1				1
Overall bunch quality	1																					1
Overall fruit pendulance quality	1																					1
Overall plant performance											1											1
Overall suckering quality	1																					1
Overall taste of cooked sample																		1				1
Oxidation in Storage Roots																		1				1
Oxygen radical absorbance capacity														1								1
panicle axis attitude														1								1
panicle axis texture														1								1
panicle basal branch number														1								1
panicle blast damage														1								1
panicle blast incidence														1								1
panicle blast severity														1								1
panicle branch arrangement														1								1
Panicle compactness											1											1
Panicle compactness and shape															1							1
Panicle desirability															1							1
Panicle diameter											1											1
panicle distance to basal branch														1								1
Panicle dry matter basis yield															1							1
Panicle dry matter content															1							1
Panicle dry weight per plant														1								1
panicle exsertion														1								1
Panicle girth											1											1
Panicle harvest index															1							1
Panicle harvested number															1							1
Panicle moisture content															1							1
Panicle number															1							1
panicle number per plant														1								1
Panicle off type number															1							1
Panicle selected number															1							1
panicle shattering														1								1
Panicle temperature														1								1
panicle type														1								1
Panicle weight without grain															1							1
Panicle width															1							1
Panicle yield at harvest															1							1
Panicles per hill														1								1
Parthenocarpy	1																					1
Pattern of seed testa									1													1
Pb content																	1					1
Pedicel color													1									1
Peduncle glaucousness																				1		1
Peduncle neck break																				1		1
Peduncle pubescence density																				1		1
Peel weight			1																			1
Peg pigmentation								1														1
Penicillium ear rot severity										1												1
Peonidin content																		1				1
PERCENT PROTEIN						1																1
Percentage damage to lower cros	1																					1
Percentage damage to lower cros	1																					1
Percentage damage to upper cros	1																					1
Percentage damage to upper cros	1																					1
Percentage of berries destroyed by Black rot Black																			1			1
Percentage of berries destroyed by Downy Mildew																			1			1
Percentage of berries with																			1			1
Percentage of berries with grey rot																			1			1
Percentage of berries with noble rot																			1			1
Percentage of berry set																			1			1
Percentage of colored berries																			1			1

Percentage of grain moisture								1																			1
Percentage of marketable roots																							1				1
Percentage of open flowers																								1			1
Percentage of plants with a splitti	1																										1
Percentage of plants with change	1																										1
Percentage of plants with externa	1																										1
Percentage of plants with interna	1																										1
Percentage of plants with petiole	1																										1
Percentage of plants with yellowi	1																										1
Percentage of soft berries																								1			1
Percentage of sprouted buds																								1			1
Pericarp color														1													1
Pericarp thickness																				1							1
Pest and/or disease assessment a	1																										1
Pest and/or disease assessment a	1																										1
Pest and/or disease photograph	1																										1
Pest or disease sample collection	1																										1
Pests																						1					1
petal color									1																		1
petal spot									1																		1
Petiole Color					1																						1
Petiole colour															1												1
Petiole Insertion Angle					1																						1
Petiole margin colour	1																										1
Petiole margins clasping	1																										1
Petiole margins winged	1																										1
Petiole orientation					1																						1
Petiole Pigmentation																							1				1
pH																								1			1
Phaeosphaeria leaf spot severity														1													1
Phenol content																							1				1
Phenotypic Acceptability																			1								1
Philippine downy mildew incidence														1													1
Phoma damage				1																							1
Phoma infestation										1																	1
Phoma leaf spot severity				1																							1
Phoma stem canker base stem score				1																							1
Phoma stem canker severity				1																							1
Phoma stem canker upper stem lesion score				1																							1
phosphorus concentration									1																		1
phosphorus deficiency damage																			1								1
Photochemical radiation index														1													1
photoperiod									1																		1
Photoperiod sensitivity coefficient of the variety based on CERES model																				1							1
Photosynthesis																						1					1
Photosynthetic rate																			1								1
Physiological maturity thermal time																									1		1
Phytium infestation										1																	1
phytochemical compound content							1																				1
Phytophthora blight disease susceptibility																	1										1
Pigment bleeding										1																	1
Pigment simple ratio chlorophyll a																									1		1
Pigment simple ratio chlorophyll b																									1		1
Pigmentation of outer surface of	1																										1
pink bollworm resistance									1																		1
Pistil pigmentation																		1									1
Pith area																			1								1
Pith diameter																			1								1
Pith major diameter																			1								1
Pith minor diameter																			1								1
Place of roots on tubers																										1	1
Plant																						1					1
Plant agronomic appreciation																				1							1
plant anatomy related traits							1																				1
Plant annual yield	1																										1
Plant anthocyanin presence																				1							1
Plant Architecture					1																						1
Plant basal tiller number																				1							1
Plant biomass			1																							1
Plant branch number												1															1
Plant branching																					1						1
Plant chlorosis			1																							1
Plant circumference 100 cm from	1																										1
Plant circumference 20 cm from t	1																										1
Plant circumference 75 cm from t	1																										1
Plant circumference at collar	1																										1
plant color									1																		1
Plant composition																						1					1
Plant crop cycle	1																										1
Plant damage options	1																										1
Plant density	1																										1
Plant diameter			1																							1
Plant dry weight content										1																	1
Plant earliness						1																					1
Plant emergence proportion																		1									1
Plant establishment		1																								1
Plant Fertility																	1										1
Plant groundcover			1																							1
Plant growing period																		1									1
Plant hairiness										1																	1
Plant height to insertion of first tassel branch														1													1
Plant height to ligule of the flag leaf														1													1
Plant height to neck of peduncle	1																										1
Plant hybrid necrosis incidence																									1		1
Plant ID	1																										1
Plant ID manual entry	1																										1
Plant ID QR code	1																										1
Plant infected with Striga										1																	1
Plant leaf weight												1															1
Plant lifecycle stage options	1																										1
Plant nitrogen content											1																1
Plant number per experimental unit							1																				1
Plant number per plot																1											1
Plant peg number												1															1
Plant phenotypic performance			1																							1
Plant photoperiod response																									1		1
Plant pod number												1															1
Plant pod weight												1															1
Plant reaction to blast																1											1
Plant reaction to rust																1											1
plant relative water content	1																										1
Plant reproductive mode			1																							1
Plant seed number												1															1
Plant seed weight												1															1
Plant senescence																	1										1
Plant sex																										1	1
Plant spacing	1																										1
Plant stand at emergence										1																	1
Plant stand at harvest										1																	1
Plant stand establishment											1																1
Plant Stands Harvested					1																						1
Plant state description	1																										1
Plant state options	1																										1
Plant stem weight												1															1
Plant structure													1														1
Plant tillering capacity		1																								1
Plant vegetative growth rate																				1							1
Plant vegetative period																									1		1
plant water loss	1																										1
Plantable stem length					1																						1
Plot																						1					1
plot homogeneity									1																		1
PLOT INFECTED WITH ALECTRA										1																	1
plot maturity									1																		1
Plot measure validity				1																							1
plot plant stand									1																		1
Plot size				1																							1
Plumule length																	1										1
Pod angle											1																1
Pod attachment to peduncle										1																	1
Pod beak												1															1
Pod beak orientation								1																			1
Pod beak position								1																			1
Pod bearing length																	1										1
Pod biomass at R9								1																			1

Pod borers susceptibility																1										1
Pod cross section							1																			1
Pod dehiscence												1														1
Pod development time (BBCH 72-79)				1																						1
Pod filling period												1														1
Pod fly susceptibility																1										1
Pod harvest index							1																			1
Pod Maturity Date																			1							1
Pod maturity time										1																1
Pod maturity time (BBCH 82-99)				1																						1
Pod number per node										1																1
Pod number per plant										1																1
Pod partitioning index							1																			1
Pod per plant									1																	1
Pod production efficiency							1																			1
Pod reticulation											1															1
Pod ridge											1															1
Pod scab infestation									1																	1
Pod seed number											1															1
Pod shedding												1														1
Pod shell thickness											1															1
Pod sucking bug severity									1																	1
Pod wall biomass proportion							1																			1
Pod wall thickness									1																	1
Pod wasp susceptibility																1										1
Pod wax																1										1
Pod weight per plot									1																	1
Pode emergence time									1																	1
pods biomass at R8							1																			1
PODS KG/H									1																	1
Pods per cluster																1										1
Pods per peduncule									1																	1
Podwall biomass at R9							1																			1
pollen color								1																		1
Pollen fertility															1											1
pollen sterility																		1								1
Pollen viability																	1									1
Pollinated florets number																									1	1
Pollinated spikes number																									1	1
Pollination type																									1	1
Polysora rust severity													1													1
Position of the pods in the plant							1																			1
post-harvest physiological deterioration					1																					1
potassium concentration								1																		1
potassium to sodium concentration ratio								1																		1
Potato virus X resistance																	1									1
Potato virus Y resistance																	1									1
Potato leaf roll virus resistance																	1									1
Poundability					1																					1
Powdery mildew legacy																								1		1
Powdery Mildew resistance														1												1
Powdery mildew severity																								1		1
Pratylenchus neglectus damage																								1		1
Pratylenchus thornei damage																								1		1
Predominant Flesh color																						1				1
Predominant Skin color																						1				1
Predominant sprout color																	1									1
Predominant Tuber flesh color																	1									1
Predominant tuber skin color																	1									1
Predominant Vine Color																						1				1
Presence (1) or absence (0) of powdery mildew				1																						1
Presence of bulbil																									1	1
Presence of changes in new leave	1																									1
Presence of petiole collapse cause	1																									1
Presence of pollen					1																					1
Presence of splitting pseudostem	1																									1
Presence of spot on spine base																									1	1
Presence of Tendrils												1														1
Presence of the DNA marker linked to bc-3 gene of resistance to the BCMNV							1																			1
Presence of the DNA marker linked to bgm-1 gene of resistance to the BGYMV							1																			1
Presence of the DNA marker linked to QTL of resistance to the BGYMV							1																			1
Presence of yellowing leaves caus	1																									1
Primary branches number												1														1
Primary lateral leaflet number																	1									1
Primary raceme capsule covered length						1																				1
Primary raceme capsule dry weight						1																				1
Primary raceme capsule number						1																				1
Primary raceme dry weight						1																				1
Primary raceme height						1																				1
Primary raceme length						1																				1
Primary raceme male flower covered length						1																				1
Primary raceme seed dry weight						1																				1
Primary raceme seed number						1																				1
Primary root shape																			1							1
Primary root size																			1							1
Primary seed color							1																			1
Production per effective plot area							1																			1
Productive raceme number						1																				1
Productive tiller number per plant																		1								1
productiveness								1																		1
Productivity																				1						1
projected top area	1																									1
Proportion of emerged plants																									1	1
Proportion of established plants																									1	1
Proportion of female flower					1																					1
Proportion of grains with chalkiness																		1								1
Proportion of Lodged Plants					1																					1
Proportion of off-type			1																							1
Proportion of plant with inflorescence					1																					1
Proportion of plants harvested																	1									1
Prosapia simulans adult damage			1																							1
Prosapia simulans adult number			1																							1
Prosapia simulans antibiosis			1																							1
Prosapia simulans egg number			1																							1
Prosapia simulans nymph damage			1																							1
Pruning fresh weight																							1			1
pseudostem dry weight	1																									1
pseudostem fresh weight	1																									1
Pseudostem height	1																									1
pseudostem height increase per l	1																									1
pseudostem length	1																									1
pseudostem relative water conte	1																									1
pseudostem to whole plant mass	1																									1
Pubescence Color																			1							1
Pubescence form																			1							1
Pubescence form on leaflet																			1							1
Pubescence presence																			1							1
Pubescence tip type																			1							1
Pythium spp. in field							1																			1
Pythium spp. in greenhouse							1																			1
Pythium stalk rot incidence													1													1
R8 Full Maturity																			1							1
Raceme compactness						1																				1
Raceme length covered by female flowers						1																				1
Raceme number						1																				1
Raceme position									1																	1
Raceme shape						1																				1
Rachilla hair length		1																								1
Rachis brittleness		1																								1
Rachis length																								1		1
Rachis weight	1																									1
Radiation use efficiency																								1		1
Radicle length																1										1
ragged stunt disease																		1								1
Rank of marked youngest leaf	1																									1
Rank of oldest standing leaf	1																									1
Rank of previously marked leaf	1																									1
Rapid spike growth period																								1		1
rat damage																		1								1
Rating of external symptoms of F	1																									1
Rating of internal symptoms of Fu	1																									1
ratio root to shoot	1																									1
Ratoon crop cycle	1																									1
Rb content																					1					1
Reaction to Aphids																						1				1

Reaction to Aphis glycines																	1						1
Reaction to Bacterial stem and root rot																			1				1
Reaction to Bacterial wilt																			1				1
Reaction to Beetles																			1				1
Reaction to Black rot																			1				1
Reaction to Brown ring rot																			1				1
Reaction to Charcoal rot																			1				1
Reaction to Diaporthe dry rot																			1				1
Reaction to drought																			1				1
Reaction to Early Blight: (Alternaria spp)																			1				1
Reaction to flea beetles																			1				1
Reaction to flooding																			1				1
Reaction to Foot rot																			1				1
Reaction to Fusarium oxysporum																			1				1
Reaction to Fusarium root rot																			1				1
Reaction to Fusarium surface rot																			1				1
Reaction to Grubworm																			1				1
Reaction to heat																			1				1
Reaction to high soil temperature																			1				1
Reaction to Hornworm																			1				1
Reaction to Java black rot																			1				1
Reaction to leaf spot																			1				1
Reaction to Moth																			1				1
Reaction to Other bacteria																			1				1
Reaction to Other fungi																			1				1
Reaction to Other insects																			1				1
Reaction to Other mycoplasma																			1				1
Reaction to other nematodes																			1				1
Reaction to Other virus																			1				1
Reaction to Pox or soil rot																			1				1
Reaction to Reniform nematode																			1				1
Reaction to Root Knot Nematode Meloidogyne incognita																			1				1
Reaction to Root Knot Nematode Meloidogyne spp																			1				1
Reaction to Root lesion nematode																			1				1
Reaction to salinity																			1				1
Reaction to Scab																			1				1
Reaction to Sclerotial blight and circular spot																			1				1
Reaction to Scurf																			1				1
Reaction to shade																			1				1
Reaction to Soft rot																			1				1
Reaction to soil																			1				1
Reaction to Sting nematode																			1				1
Reaction to Streptomyces Soil Rot																			1				1
Reaction to Striped sweet potato weevil																			1				1
Reaction to Sweet potato feathery mottle virus (SPMV)																			1				1
Reaction to Sweet potato flea beetles																			1				1
Reaction to Sweet potato leaf beetles																			1				1
Reaction to Sweet potato mild mottle virus (SPMMV)																			1				1
Reaction to Sweet potato moth																			1				1
Reaction to Sweet potato stem borer																			1				1
Reaction to Sweet potato vein mottle virus (SPVMV)																			1				1
Reaction to Sweet potato virus disease complex (SPVD complex)																			1				1
Reaction to sweet potato weevil																			1				1
Reaction to Sweet potato white fly																			1				1
Reaction to Sweet potato wire worms																			1				1
Reaction to West Indian sweet potato weevil																			1				1
Reaction to White rust																			1				1
Reaction to Wilt rot																			1				1
Reaction to Wire worms																			1				1
Reaction to Witches broom																			1				1
Red Spider Mite Incidence				1																			1
Red Spider Mite Severity				1																			1
reflectance								1															1
Regularity of plant stand			1																				1
Relative canopy development														1									1
Relative Storage Root Yield																			1				1
Relative water content							1																1
Remains of flower relicts at fruit a	1																						1
Residual sugar																				1			1
Resistance to bacterial blight (X. vignicola)									1														1
Resistance to Botrytis (Cluster)																				1			1
Resistance to Botrytis (Leaf)																				1			1
Resistance to chlorides																				1			1
Resistance to drought																				1			1
Resistance to Eutypa dieback Eutypa																				1			1
Resistance to iron chlorosis																				1			1
Resistance to M. incognita root knot nematode									1														1
Resistance to M. javanica root knot nematode									1														1
Resistance to Oidium (Cluster)																				1			1
Resistance to Oidium (Leaf)																				1			1
Resistance to Plasmopara (Cluster)																				1			1
Resistance to Plasmopara (Leaf)																				1			1
Resistance to Striga gesnerioides									1														1
Rest of leaves weight																1							1
Rhizoctonia in field							1																1
Rhizoctonia in greenhouse							1																1
Rhizoctonia root rot incidence																					1		1
Rhizoctonia severity		1																					1
rhizome and stolon formation															1								1
rice bug damage															1								1
rice delphacid															1								1
rice tungro disease															1								1
rice whorl maggot damage															1								1
Rodent damage																					1		1
Root																		1					1
Root abscisic acid content													1										1
Root aspect		1																					1
Root capacitance													1										1
Root constrictions number				1																			1
Root depth											1												1
Root disease development																					1		1
Root Dry Matter Content				1																			1
Root dry weight/total plant dry weight						1																	1
Root evaluation				1																			1
root fresh weight	1																						1
Root glucose content													1										1
Root knot nematode																	1						1
Root knot nematode incidence																						1	1
Root knot nematode severity																						1	1
Root lesion nematode damage																					1		1
Root lodging incidence													1										1
Root mean diameter		1																					1
Root Neck Length				1																			1
Root nematode density	1																						1
Root nodule weight																	1						1
Root Number				1																			1
Root Position				1																			1
Root proline content													1										1
root relative water content	1																						1
Root rot resistance					1																		1
Root sucrose content													1										1
Root Surface Color				1																			1
Root Surface Texture				1																			1
Root to Shoot Ratio											1												1
root to whole plant mass ratio	1																						1
Root Vigor		1																					1
Root volume in field							1																1
Root volume in greenhouse							1																1
Root Yield				1																			1
Rooting depth						1																	1
Rootstock adventitious root formation																				1			1
Rootstock callus																				1			1
Rootstock canes/ha																				1			1
Rosette disease incidence											1												1
Rotted Storage Roots				1																			1
Rotten root proportion				1																			1
Rotten tuber weight																						1	1
Row ID	1																						1
RtCYld_Cp_tha																			1				1
Russian wheat aphid damage																					1		1
Rust on leaves in field							1																1
Rust on leaves in greenhouse							1																1
Rust on pods in field							1																1
Rust resistance												1											1
Rust susceptibility										1													1

S content																			1				1
salt injury																	1							1
Sawfly damage																							1		1
Scale insect infestation																								1	1
scent																	1							1
Se content																			1				1
Second internode length																	1							1
Secondary branch length				1																				1
Secondary branch number				1																				1
Secondary branches number											1													1
Secondary Flesh color																					1			1
secondary panicle branching																	1							1
Secondary raceme dry weight				1																				1
Secondary raceme length				1																				1
Secondary raceme number				1																				1
Secondary seed color						1																		1
Secondary Skin color																					1			1
Secondary sprout color																1								1
Secondary sprouting																								1	1
Secondary Vine Color																					1			1
Seed																			1					1
Seed appearance and uniformity										1														1
seed arginine content							1																	1
Seed Arsenic content		1																						1
Seed Ash content						1																		1
seed aspartate content							1																	1
Seed base colour															1									1
Seed Bearer																			1					1
Seed biomass at R9						1																		1
Seed Boron content		1																						1
Seed Brassicasterol content		1																						1
Seed brilliance						1																		1
Seed Cadmium content		1																						1
Seed calcium content in field						1																		1
Seed calcium content in greenhouse						1																		1
Seed capsule set																					1			1
Seed Chromium content		1																						1
seed coat fragment							1																	1
seed coat index							1																	1
Seed coat mottling																		1						1
seed coat nep count							1																	1
Seed coat pattern								1																1
seed coat percent							1																	1
Seed Cobalt content		1																						1
Seed color pattern						1																		1
Seed colour													1											1
Seed cooking time									1															1
seed cotton weight per boll							1																	1
seed cotton yield							1																	1
Seed crowding								1																1
seed cysteine content							1																	1
Seed ends										1														1
Seed filling period																		1						1
seed fuzz color							1																	1
seed fuzz grade							1																	1
Seed gall nematode damage																							1		1
Seed germination rate																								1	1
Seed glucosinolate content		1																						1
seed glycine content							1																	1
Seed gross yield	1																							1
Seed harvest time	1																							1
Seed Height						1																		1
Seed hilum shape													1											1
Seed Iron						1																		1
Seed isoflavone content																		1						1
seed isoleucine content							1																	1
seed kernel content							1																	1
seed kernel index							1																	1
Seed Lead content		1																						1
seed leucine content							1																	1
Seed linoleic acid content																		1						1
Seed Lithium content		1																						1
Seed lustre													1											1
Seed lysine content																		1						1
Seed magnesium content in field						1																		1
Seed magnesium content in greenhouse						1																		1
Seed methionine content																		1						1
Seed milling fraction															1									1
Seed Molybdenum content		1																						1
Seed mottling													1											1
Seed net yield	1																							1
Seed Nickel content		1																						1
seed nitrogen content							1																	1
Seed Nitrogen content in field						1																		1
Seed Nitrogen content in greenhouse						1																		1
seed number per acre							1																	1
seed number per boll							1																	1
Seed number per pod									1															1
Seed oil/protein content																		1						1
Seed Oleic / Linoleic acid ratio										1														1
Seed per plant					1																			1
Seed per pod								1																1
seed percent							1																	1
seed phenylalanine content							1																	1
Seed Phosphorus						1																		1
Seed phosphorus content in greenhouse						1																		1
Seed Phosphosrus content													1											1
Seed potassium content in field						1																		1
Seed potassium content in greenhouse						1																		1
Seed production efficiency						1																		1
Seed production per berry																1								1
Seed Protein						1																		1
Seed protein yield		1																						1
Seed Raffinose content																		1						1
Seed ricin content				1																				1
Seed ricinine content				1																				1
Seed Rubidium content		1																						1
seed serine content							1																	1
Seed set														1										1
Seed sound mature proportion										1														1
Seed starchyose content																		1						1
Seed Strontium content		1																						1
Seed Sucrose content																		1						1
Seed Sulphur content		1																						1
Seed surface					1																			1
Seed testa ground colour									1															1
Seed thickness								1																1
seed type							1																	1
seed valine content							1																	1
seed vigor							1																	1
Seed weevil susceptibility									1															1
Seed yield fresh weight basis				1																				1
Seed yield per area											1													1
Seed yield per ha					1																			1
Seed yield per plot					1																			1
Seed Zinc						1																		1
Seedcoat								1																1
Seedcoat color								1																1
seeding date																	1							1
Seedling																			1					1
Seedling disease incidence												1												1
Seedling Establishment rate																								1	1
seedling height																	1							1
Seedling transplant survival rate																								1	1
Seeds germinated																								1	1
Seeds germination end time																								1	1
Seeds germination start time																								1	1
Seeds per plot						1																		1
Seeds set per fruit																								1	1
Seeds sown																								1	1
Selected variety			1																					1
Semolina color																							1		1
Semolina protein content																							1		1
Semolina yellow pigment content																							1		1
Senescenece class																								1	1

Sepal apex																				1				1
Sepal pubescence																				1				1
Sepal shape																				1				1
Septoria blotch incidence																						1		1
Septoria blotch legacy																						1		1
Septoria blotch plant response																						1		1
Septoria blotch severity																						1		1
Septoria nodorum blotch incidence																						1		1
Septoria nodorum blotch legacy																						1		1
Septoria nodorum blotch severity																						1		1
Septoria severity								1																1
Septoria tritici blotch incidence																						1		1
Septoria tritici blotch legacy																						1		1
Septoria tritici blotch plant response																						1		1
Septoria tritici blotch severity																						1		1
Shape of Central Leaf Lobe																				1				1
Shape of limb																				1				1
Sharp eyespot incidence																						1		1
sheath blight															1									1
Sheath pubescence										1														1
sheath rot															1									1
Shelled cob weight										1														1
Shelling index											1													1
Shelling ratio									1															1
Shoot apex compactness				1																				1
Shoot apex size				1																				1
Shoot Ash content in field						1																		1
Shoot Ash content in greenhouse						1																		1
Shoot biomass			1																					1
Shoot biomass in field						1																		1
Shoot biomass in greenhouse						1																		1
Shoot calcium content in field						1																		1
Shoot calcium content in greenhouse						1																		1
Shoot diameter																					1			1
Shoot dry weight					1																			1
Shoot magnesium content in field						1																		1
Shoot magnesium content in greenhouse						1																		1
Shoot Nitrogen content in field						1																		1
Shoot Nitrogen content in greenhouse						1																		1
Shoot phosphorus content in field						1																		1
Shoot phosphorus content in greenhouse						1																		1
Shoot potassium					1																			1
Shoot potassium content in field						1																		1
Shoot potassium content in greenhouse						1																		1
Shoot sodium					1																			1
Shoot: area of the anthocyanin coloration on bud scales																					1			1
Shoot: attitude (before tying)																					1			1
Shoot: color of dorsal side of internodes																					1			1
Shoot: color of dorsal side of nodes																					1			1
Shoot: color of ventral side of internodes																					1			1
Shoot: color of ventral side of nodes																					1			1
Shoot: density of erect hairs on internodes																					1			1
Shoot: density of erect hairs on nodes																					1			1
Shoot: density of prostrate hairs on internodes																					1			1
Shoot: density of prostrate hairs on nodes																					1			1
Shoot: fertility of basal buds (buds 1-3)																					1			1
Shoot: intensity of anthocyanin coloration on bud scales																					1			1
Shoot: length of tendrils																					1			1
Shoot: number of consecutive tendrils																					1			1
Shooting photograph	1																							1
short fiber content							1																	1
Silk Anthocyanin coloration										1														1
Silk growth										1														1
Silk growth rate										1														1
Silk length										1														1
Silking time										1														1
Single berry volume																					1			1
Single berry weight																					1			1
Single cluster weight																					1			1
single leaf length	1																							1
single leaf width	1																							1
Single seed weight																					1			1
Skin Color of roots																				1				1
Skin Texture of roots																				1				1
Small size tuber length																							1	1
Small size tuber weight per plant																							1	1
Small size tuber width																							1	1
Small size tubers per plant																							1	1
Small size tubers per plot																							1	1
Small size tubers weight per plot																							1	1
Smoothness of pounded yam																							1	1
Smut incidence												1												1
Smut infection proportion													1											1
Smut infestation								1																1
Smuts incidence		1																						1
Snail damage																						1		1
Snapback												1												1
SO content																			1					1
sodium concentration							1																	1
Soil																		1						1
Soil acidity stress susceptibility														1										1
Soil acidity susceptibility		1																						1
Soil borne mosaic incidence																						1		1
Soil insect damage																				1				1
Soil salinity stress susceptibility														1										1
Sooty Mould				1																				1
Sooty stripe disease presence																1								1
Sorghum downy mildew severity										1														1
Sorghum rust incidence																1								1
Sorghum shoot fly incidence																1								1
Sorghum stem borer incidence																1								1
Southern blight in field						1																		1
Southern blight in greenhouse						1																		1
Southern blight plant response																						1		1
Sowing time																1								1
Soybean anthracnose																	1							1
Soybean Mosaic Virus																	1							1
Soybean rust																	1							1
SPAD after 7days under stress									1															1
SPAD at harvest (10d)									1															1
SPAD at the start of stress									1															1
SPAD chlorophyll meter reading						1																		1
Spad chlorophyll meter reading in greenhouse						1																		1
Spatial position ID	1																							1
Spatial position status	1																							1
Specific area of flag leaf and flag leaf-1															1									1
Specific root length						1																		1
spider mite resistance							1																	1
Spike attitude		1																						1
Spike awnedness																						1		1
Spike basal sterility presence		1																						1
Spike colour		1																						1
Spike disease development																						1		1
Spike dry weight																						1		1
Spike partitioning index																						1		1
Spike photosynthesis contribution																						1		1
Spike shattering																						1		1
Spike sterility index																						1		1
Spike threshability																						1		1
Spike waxiness		1																						1
spikelet fertility															1									1
Spikelet Fusarium Head Blight incidence		1																						1
Spikelet number																						1		1
Spikelet number per spike		1																						1
Spikelet shattering susceptibility		1																						1
Spikelet sterility															1									1
Spikelets density															1									1
Spikelets per hill															1									1
Spikelets per panicle															1									1
Spines on sprout presence																							1	1
Spines on stem density																							1	1
Spiraling Whitefly Incidence				1																				1
Spiraling Whitefly Severity				1																				1
Spittlebug adult number			1																					1
Spittlebug egg number			1																					1

Splitting of testa								1																1
Spot blotch incidence																						1		1
Spot blotch legacy																						1		1
Spot Blotch susceptibility		1																						1
Springiness of pounded yam																							1	1
Sprout color																							1	1
Sprout count					1																			1
Sprout hairiness																							1	1
Sprout length																							1	1
Sprouting					1																			1
Sprouting ability																					1			1
Sprouting susceptibility		1																						1
Sprouting tolerance																						1		1
Sr content																				1				1
Stalk length					1																			1
Stalk rot incidence												1												1
Stand establishment																						1		1
Stand harvested number																							1	1
Standard germination												1												1
Standard petal colour									1															1
Starch Content					1																			1
Start date and time of data collec	1																							1
Status of fruit development																							1	1
Stay-green																	1							1
Stem ash content																	1							1
Stem biomass			1																					1
Stem biomass at R8						1																		1
Stem biomass at R9						1																		1
Stem Biomass dry matter basis yield																		1						1
Stem biomass dry matter content																		1						1
Stem biomass moisture content																		1						1
Stem biomass reduction						1																		1
Stem biomass yield at harvest																		1						1
Stem cell wall carbohydrate content																	1							1
Stem cortex color					1																			1
Stem diameter - 2nd internode																	1							1
Stem elongated internodes number																		1						1
stem elongation																	1							1
Stem epidermis color					1																			1
Stem extractives content																	1							1
stem gossypol glands							1																	1
Stem hairiness																							1	1
Stem internodes length																		1						1
Stem juice sugar content																		1						1
Stem juice volume																		1						1
Stem juice weight																		1						1
Stem lodging incidence												1												1
Stem major diameter - 2nd internode																	1							1
Stem minor diameter - 2nd internode																	1							1
Stem nodal tiller number																		1						1
stem node number							1																	1
Stem number					1																			1
Stem protein content																	1							1
stem rot																	1							1
Stem rot damage								1																1
Stem rot incidence										1														1
Stem rot susceptibility									1															1
Stem rust coefficient of infection																						1		1
Stem rust incidence													1											1
Stem rust infection type													1											1
Stem rust notes																						1		1
Stem rust plant response																						1		1
Stem rust seedling response																						1		1
Stem solidness																						1		1
Stem starch content																	1							1
Stem strength																						1		1
Stem sucrose content																	1							1
Stem termination type																			1					1
Stem total lignin content																	1							1
Stem total nonstructural carbohydrate content																	1							1
Stem total structural materials content																	1							1
Stem upper node pigmentation		1																						1
Stem wall thickness																						1		1
Stem water soluble carbohydrate																						1		1
Stem weevil resistance											1													1
Stem weight					1																			1
Stem width				1																				1
Stemphylium blight resistance											1													1
Stems per plant																							1	1
Stenocarpella ear rot severity												1												1
Stenocarpella stalk rot incidence												1												1
sterile lemma color																	1							1
sterile lemma length																	1							1
sterile lemma shape																	1							1
Sterility mosaic incidence															1									1
Stewarts wilt incidence												1												1
Stickiness of pounded yam																							1	1
Stigma exertion																					1			1
Stigma shape																1								1
Stipe length																				1				1
Stipule length								1																1
Stipule margin					1																			1
Stipule width								1																1
Stipules Length					1																			1
Stock weight					1																			1
Stomatal resistance																	1							1
Storability																			1					1
Storage Root Appearance																					1			1
Storage Root Attachement																					1			1
Storage Root Constrictions					1																			1
Storage Root Cortex Color					1																			1
Storage root cracking																					1			1
Storage Root Defects																					1			1
Storage Root Diameter					1																			1
Storage root dry matter content																					1			1
Storage root form																					1			1
Storage root formation																					1			1
Storage Root Peduncle					1																			1
Storage Root Periderm Color					1																			1
Storage Root Pulp Color					1																			1
Storage Root Shape (primary)																					1			1
Storage Root Shape (secondary)																					1			1
Storage Root Shape Uniformity																					1			1
Storage root starch content																					1			1
Storage Root Surface Defects																					1			1
Storage root texture																					1			1
Storage root weight					1																			1
Storage Root Yield relative to check																					1			1
Stover Biomass dry matter basis yield																		1						1
Stover biomass dry matter content																		1						1
Stover biomass moisture content																		1						1
Stover biomass yield at harvest																		1						1
Stover metabolised energy																		1						1
Stover nitrogen digestibility																		1						1
Stover organic matter digestibility																		1						1
Straw breakage																						1		1
Straw color																						1		1
Straw dry matter per plant																	1							1
Straw protein content											1													1
Straw strength																						1		1
STRGDRWT								1																1
Striga damage												1												1
Striga- Height of Striga								1																1
Striga incidence																		1						1
Striga infestation								1																1
Striga maximum vigor																		1						1
Striga mean vigor																		1						1
Striga tolerance														1										1
Stripe rust coefficient of infection																						1		1
Stripe rust incidence																						1		1
Stripe rust notes																						1		1
Stripe rust plant response																						1		1
Stripe rust seedling response																						1		1
Stripe rust severity																						1		1
Stripe rust spike severity																						1		1

Stump weight		1																1
submergence tolerance											1							1
Sucrose content														1				1
Sugar															1			1
Sugar Content		1																1
Sugarcane downy mildew incidence									1									1
Sugarcane mosaic virus incidence									1									1
Suitability for Intercropping		1																1
sulfur concentration						1												1
Suni pest damage																1		1
Surface of foliage attacked by Downy Mildew															1			1
Surface of foliage attacked by Powdery Mildew															1			1
Survival index														1				1
Sweetness of boiled storage root flesh														1				1
Swelling capacity		1																1
sympodia branch number						1												1
system water loss	1																	1
system water loss per leaf area	1																	1
Take all incidence																1		1
Tallest sucker height	1																	1
Tallest sucker number of function	1																	1
Tan spot incidence																1		1
Tan spot plant response																1		1
Tan spot seedling response																1		1
Tan spot severity																1		1
Tap root diameter							1											1
tap root diameter at 10							1											1
tap root diameter at 15							1											1
tap root diameter at 5							1											1
Tar spot complex severity									1									1
Tartaric acid concentration															1			1
Tassel Anthocyanin coloration									1									1
Tassel blasting severity									1									1
Tassel branch number									1									1
Tassel branch weight									1									1
Tassel branching type									1									1
Tassel density									1									1
Tassel exertion									1									1
Tassel glumes Anthocyanin coloration									1									1
Tassel glumes base Anthocyanin coloration									1									1
Tassel opening									1									1
Tassel size									1									1
Taste				1														1
Taste Of Boiled Roots		1																1
Taste of pounded yam																	1	1
Technological quality trait													1					1
Terminal spikelet thermal time																1		1
Terminal spikelet time																1		1
Termites infestation							1											1
Tertiary branches number								1										1
Tertiary branches number per plant				1														1
Tertiary raceme dry weight			1															1
Tertiary raceme length			1															1
Tertiary raceme number			1															1
Test weight										1								1
Testa texture							1											1
Thermal time to panicle inititation												1						1
Thousand seed dry weight			1															1
Threshability							1											1
thrip damage											1							1
Thrip damage to podset							1											1
Thrip damage to stipules							1											1
Thrip Resistance							1											1
Tiller density											1							1
Tiller fertility											1							1
Tiller number per hill											1							1
Tiller number per plant											1							1
Tillering									1									1
Tillering capacity																1		1
Tilletia laevis bunt incidence																1		1
Tilletia tritici bunt incidence																1		1
Time from flowering to harvest	1																	1
Time from harvest to death	1																	1
Time from planting to death	1																	1
Time from planting to first young	1																	1
Time from planting to shooting	1																	1
Time from shooting to harvest	1																	1
Time from start of crop cycle to fi	1																	1
Time of autumn coloring of leaves															1			1
Time of beginning of wood maturity															1			1
Time of physiological stage of full maturity of the berry															1			1
Time to 50% flowering																	1	1
Time to 50% foliage senescence																	1	1
Time to bulbil initiation																	1	1
Time to end of flowering																	1	1
Time to first flower							1											1
Time to first leaf emergence																	1	1
Time to first mature pod							1											1
Time to first pod formation								1										1
Time to flower initiation																	1	1
Time to flowering (from emergence)											1							1
Time to flowering (from sowing)											1							1
Time to flowering (from transplanting)											1							1
Time to fruit harvesting																	1	1
Time to fruit maturity																	1	1
Time to fruit setting																	1	1
Time to initial flowering (from sowing)											1							1
Time to initial flowering (from transplanting)											1							1
Time to maturity (from sowing)											1							1
Time to maturity (from transplanting)											1							1
Time to panicle exsertion (from heading)											1							1
Time to pod formation				1														1
Tip shoot weight		1																1
Titratable acidity															1			1
To color												1						1
To consistency												1						1
Tocopherol concentration										1								1
Tocotrienol concentration										1								1
Tolerance to Phylloxera (Leaf)															1			1
Tolerance to Phylloxera (Root)															1			1
Total acid of must															1			1
Total Carotenoid Retention		1																1
Total carotenoids														1				1
Total dry matter per plant											1							1
Total finger weight	1																	1
Total fresh tuber weight per plot																	1	1
total length of all leaves	1																	1
Total Monomeric Anthocyanin content														1				1
total nep count						1												1
Total non-structural carbohydrates in leaves - Field					1													1
Total non-structural carbohydrates in leaves - Greenhouse					1													1
Total non-structural carbohydrates in seed					1													1
Total non-structural carbohydrates in shoot - Field					1													1
Total non-structural carbohydrates in shoot - Greenhouse					1													1
Total non-structural carbohydrates in stem - Field					1													1
Total non-structural carbohydrates in stem - Greenhouse					1													1
Total number of inflorescences/number of primary shoots															1			1
Total number of root														1				1
Total number of shoots															1			1
Total number of shoots per plant															1			1
Total phenolic															1			1
TOTAL PODS							1											1
TOTAL PODS HARVESTED							1											1
Total root biomass per area					1													1
Total root biomass per plant					1													1
Total root length per area					1													1
Total root length per plant					1													1
Total root weight														1				1
TOTAL SEED HARVESTED							1											1
Total Seed Phytates					1													1
Total soluble solids															1			1
Total stem materials content											1							1
Total sugar content														1				1
Total tuber weight per plant																	1	1
Total tuber yield																	1	1

Total tuber yield adjusted																							1	1
Total tubers per plant																							1	1
Total tubers per plot																							1	1
Trans-9 activity				1																				1
Translocation 1BL/1RS																						1		1
Transpiration									1															1
Transpiration rate																1								1
trash content							1																	1
Trial group ID	1																							1
Trial ID	1																							1
trichome density on bract							1																	1
trichome density on leaf							1																	1
trichome density on plant							1																	1
trichome density on stem							1																	1
Tropical rust severity											1													1
True seeds harvested																							1	1
True stem partitioning index																						1		1
Tuber Anthocyanin concentration in dry weight basis															1									1
Tuber Anthocyanin concentration in fresh weight basis															1									1
Tuber Ascorbic acid concentration in dry weight basis															1									1
Tuber Ascorbic acid concentration in fresh weight basis															1									1
Tuber beetle incidence																							1	1
Tuber beetle severity																							1	1
Tuber cooking quality															1									1
Tuber cooking time															1									1
Tuber cracks																							1	1
Tuber depth eyes															1									1
Tuber fiber content															1									1
Tuber flavor after cooking															1									1
Tuber flesh color																							1	1
Tuber flesh cross-section color uniformity																							1	1
Tuber flesh oxidation																							1	1
Tuber flesh oxidation color																							1	1
Tuber flesh oxidation intensity																							1	1
Tuber flesh secondary color															1									1
Tuber flesh texture																							1	1
Tuber fructose content															1									1
Tuber glucose content															1									1
Tuber hairiness																							1	1
Tuber Hydrophilic antioxidant activity in dry weight basis															1									1
Tuber Hydrophilic antioxidant activity in fresh weight basis															1									1
Tuber Iron concentration in dry weight basis															1									1
Tuber Iron concentration in fresh weight basis															1									1
Tuber length																							1	1
Tuber Lipophilic antioxidant activity in dry weight basis															1									1
Tuber Lipophilic antioxidant activity in fresh weight basis															1									1
Tuber maltose content															1									1
Tuber number															1									1
Tuber number per plant															1									1
Tuber protein content															1									1
Tuber skin main color intensity															1									1
Tuber skin secondary color															1									1
Tuber specific gravity															1									1
Tuber sprouting pattern															1									1
Tuber starch content															1									1
Tuber sucrose content															1									1
Tuber surface texture																							1	1
Tuber texture after cooking															1									1
Tuber thorniness																							1	1
Tuber thorniness intensity																							1	1
Tuber uniformity															1									1
Tuber weight															1									1
Tuber weight per plant															1									1
Tuber width																							1	1
Tuber yield no adjusted															1									1
Tuber Zinc concentration in dry weight basis															1									1
Tuber Zinc concentration in fresh weight basis															1									1
Tuberous root cyanide content				1																				1
Tubers planted																							1	1
Turcicum leaf blight severity											1													1
Twining																				1				1
Twining date																		1						1
Twining direction																							1	1
Twining habit																							1	1
Twinning tendency								1																1
Type of planting material	1																							1
Type of tendril										1														1
Typhlodromalus aripo				1																				1
udbatta disease																1								1
ufra																1								1
Undehulled seed												1												1
Undesirable color of boiled storage root																				1				1
Unfilled grain weight																1								1
Unfilled spikelets density																1								1
Unfilled spikelets per panicle																1								1
Unfilled spikelets proportion																1								1
Unfilled spikelets weight per area																1								1
Unusual tuber shape															1									1
upper quartile length by weight							1																	1
Uppermost ear shape											1													1
Variability of storage root shape																				1				1
Variability of storage root size																				1				1
Variety farmer preference																	1							1
Variety group					1																			1
Variety maturity appreciation																	1							1
Variety photoperiod sensitivity coefficient																	1							1
Vascular disease																			1					1
Vegetative vigor													1											1
Vein color																							1	1
Veraison																					1			1
Verticilium wilt presence			1																					1
verticillium wilt disease incidence							1																	1
verticillium wilt disease severity							1																	1
verticillium wilt resistance							1																	1
vigor																1								1
Vigor of shoot growth																					1			1
Vine cutting establishment rate																							1	1
Vine cuttings planted																							1	1
Vine internode diameter																				1				1
Vine internode length																				1				1
Vine Tips Pubescence																				1				1
Vine vigor																				1				1
Viral (stunt) resistance					1																			1
Viral disease																			1					1
Virus symptoms																				1				1
Visual rooting depth						1																		1
Visual selection scores at flowering stage														1										1
Visual selection scores at maturity stage														1										1
Visual selection scores at podding stage														1										1
Vitamin C content	1																							1
Wasps								1																1
waste							1																	1
Water extraction capacity																1								1
Water extraction rate		1																						1
Water index											1													1
Water logging tolerance										1														1
Water use efficiency					1																			1
Waterlogging damage																						1		1
Waterlogging tolerance		1																						1
Web blight damage								1																1
Web blight in field						1																		1
Web blight in greenhouse						1																		1
Weekly leaf emission rate	1																							1
Weevils damage			1																					1
Weight of 100 seeds														1										1
Weight of Alectra								1																1
Weight of mashed cassava cake				1																				1
Weight of non-commercial storage roots																				1				1
Weight of planted tubers																							1	1
Weight of storage root																				1				1
Weight of vines																				1				1
wet tolerance							1																	1
Wheat blast severity																						1		1
White mold in field						1																		1
White mold in greenhouse						1																		1

whitebacked planthopper damage											1							1
Whitefly resistance									1									1
Whole above ground biomass dry matter basis yield												1						1
Whole above ground biomass dry matter content												1						1
Whole above ground biomass moisture content												1						1
Whole above ground biomass yield at harvest												1						1
Whole meal flour ash content																1		1
Whole meal flour color																1		1
Whole meal flour falling number																1		1
Whole meal flour SDS sedimentation																1		1
Whole meal flour yellow pigment content																1		1
Wilting score					1													1
Wing petal colour							1											1
Wing stem shape										1								1
Winter kill damage																1		1
Winter kill susceptibility		1																1
Woody shoot: cross section															1			1
Woody shoot: erect hairs on internodes															1			1
Woody shoot: erect hairs on nodes															1			1
Woody shoot: lenticels															1			1
Woody shoot: main color															1			1
Woody shoot: structure of surface															1			1
Worm damage																1		1
Wort FAN content		1																1
Wort filtration rate		1																1
Wort viscosity		1																1
Yam anthracnose disease incidence																	1	1
Yam anthracnose disease severity																	1	1
Yam tuber dry rot incidence																	1	1
Yam tuber dry rot presence																	1	1
Yam tuber dry rot severity																	1	1
yarn strength						1												1
yellow dwarf											1							1
Yellow mosaic virus resistance									1									1
yellow mottle disease											1							1
Yellow Rust coefficient of infection		1																1
Yellow Rust plant response		1																1
Yellow Rust severity		1																1
Yellow Rust severity and response		1																1
Yellow Rust susceptibility		1																1
yellowness						1												1
Yield															1			1
Yield of total roots														1				1
Yield per day					1													1
Yield per hectare					1													1
Yield per plant					1													1
Yield production efficiency					1													1
Young leaf chlorophyll content								1										1
Young leaf: color of the upper side of blade (4th leaf)															1			1
Young leaf: density of erect hairs between main veins on lower side of blade (4th leaf)															1			1
Young leaf: density of erect hairs on main veins on lower side of blade															1			1
Young leaf: density of prostrate hairs between main veins on lower side of blade (4th leaf)															1			1
Young leaf: density of prostrate hairs on main veins on lower side of blade (4th leaf)															1			1
Young Shoot: aperture of tip															1			1
Young Shoot: density of erect hairs on tip															1			1
Young Shoot: density of prostrate hairs on tip															1			1
Young Shoot: distribution of anthocyanin coloration on prostrate hairs of tip															1			1
Young Shoot: intensity of anthocyanin coloration on prostrate hairs of tip															1			1
Young Stem Growth Habit				1														1
Young Stem Pubescence				1														1
Youngest leaf spotted	1																	1
Zinc content in grain											1							1
zinc deficiency injury											1							1
Zn content													1					1
Zulia carbonaria adult damage			1															1
Zulia carbonaria adult number			1															1
Zulia carbonaria antibiosis			1															1
Zulia carbonaria egg number			1															1
Zulia carbonaria nymph damage			1															1
