# Supplementary material for: Challenges for FAIR-compliant description and comparison of crop phenotype data with standardized controlled vocabularies
Source: Database (Oxford). 2021 May 15;2021:baab028. doi: 10.1093/database/baab028 (PMC8122365; doi:10.1093/database/baab028)
Supplement: baab028_Supp [file baab028_supp.zip › SupplementalTable3.pdf]

	Banan	Barley	Brachi	Brassic	Cassav	Castor	Chickp	Comm	Cottor	Cowpe	Faba b	Grounc	Lentil	Maize	Mungb	Oat	Pearl r	Pigeor	Potatc	Rice	Sorghı	Soybe	Sugar	Sugar l	Sweet	Vitis	Whea	Yam
Banana		0.25	0.29	0.00	0.22	1.74	1.11	0.29	0.24	0.22	0.28	0.56	0.29	0.28	0.28	0.32	0.62	0.30	0.00	0.37	0.20	0.56	0.00	0.00	0.43	0.21	0.16	0.00
Barley	0.25		1.02	0.90	0.33	3.02	3.27	1.28	1.08	2.62	3.81	2.88	3.59	3.03	4.83	1.78	1.12	2.09	0.41	2.27	3.42	1.89	0.00	0.00	0.34	0.00	6.06	0.00
Brachiaria	0.29	1.02		0.00	0.82	2.92	2.63	1.02	0.92	0.82	0.68	1.37	3.76	1.87	2.07	0.93	0.85	0.78	0.55	1.19	0.50	2.00	0.00	0.00	0.00	0.00	1.25	0.89
Brassica	0.00	0.90	0.00		0.00	0.61	2.25	0.36	0.00	0.37	6.32	1.74	0.63	0.34	7.60	0.00	0.00	1.29	0.00	0.00	0.44	1.70	0.00	0.00	0.00	0.00	0.47	0.00
Cassava	0.22	0.33	0.82	0.00		1.62	1.15	0.28	1.22	0.57	0.39	1.95	0.82	0.79	0.78	0.46	0.88	0.84	0.34	0.90	1.29	0.38	0.00	0.91	2.10	0.26	0.39	1.19
Castorbean	1.74	3.02	2.92	0.61	1.62		4.52	1.57	2.73	2.44	3.97	4.70	2.94	1.99	6.76	1.82	1.67	2.27	1.08	0.59	1.47	4.58	0.00	0.00	0.78	0.36	1.99	1.75
Chickpea	1.11	3.27	2.63	2.25	1.15	4.52		4.09	3.40	3.45	4.22	7.32	13.91	0.70	8.59	0.80	1.48	8.16	1.00	2.27	0.46	5.95	0.00	0.00	1.11	0.00	1.91	0.41
Common B	0.29	1.28	0.80	0.36	0.28	1.57	4.09		0.30	3.33	2.26	2.66	2.40	0.78	3.44	0.45	0.43	3.25	0.00	0.66	0.31	1.50	0.00	0.00	0.54	0.00	1.55	0.00
Cotton	0.24	1.08	0.92	0.00	1.22	2.73	3.40	0.30		1.23	2.16	3.06	2.78	0.85	3.51	0.53	1.00	2.83	0.75	0.48	0.70	4.29	0.00	0.00	0.00	0.00	1.24	1.62
Cowpea	0.22	2.62	0.82	0.37	0.57	2.44	3.45	3.33	1.23		3.11	3.59	2.89	1.86	6.30	1.39	0.88	2.84	0.00	1.35	1.94	2.70	0.00	0.00	0.55	0.00	1.77	1.17
Faba bean	0.28	3.81	0.68	6.32	0.39	3.97	4.22	2.26	2.16	3.11		4.38	1.36	0.71	19.50	1.65	1.53	4.20	0.00	0.57	1.40	4.88	0.00	0.00	0.00	0.00	1.21	0.84
Groundnut	0.56	2.88	1.37	1.74	1.95	4.70	7.32	2.66	3.06	3.53	4.38		3.45	0.71	5.73	0.00	0.78	5.67	0.52	1.15	2.35	4.32	0.00	0.00	0.38	0.35	1.46	0.84
Lentil	0.29	3.59	3.76	0.63	0.82	2.94	13.91	2.40	2.78	2.89	1.36	3.45		1.50	6.94	2.83	0.86	2.34	1.10	1.50	1.00	2.01	0.00	0.00	0.40	0.00	2.01	0.45
Maize	0.28	3.03	1.87	0.34	0.79	1.99	0.70	0.78	0.85	1.86	0.71	0.71	1.50		1.43	2.07	1.59	1.14	0.95	2.35	3.58	1.06	0.00	0.00	0.00	0.00	4.31	1.39
Mungbean	0.28	4.83	2.07	7.60	0.78	6.76	8.59	3.44	3.51	6.30	19.50	5.73	6.94	1.43		1.69	1.56	7.14	1.04	1.16	1.89	6.83	0.00	0.00	0.38	0.00	3.41	1.69
Oat	0.32	1.78	0.93	0.00	0.46	1.82	0.80	0.45	0.53	1.39	1.65	0.00	2.83	2.07	1.69		1.11	0.98	0.00	0.97	2.30	0.81	0.00	0.00	0.00	0.00	2.68	0.00
Pearl millet	0.62	1.12	0.85	0.00	0.88	1.67	1.48	0.43	1.00	0.88	1.53	0.78	0.86	1.59	1.56	1.11		0.89	0.61	1.57	3.80	0.75	0.00	0.00	0.00	0.00	0.78	0.48
Pigeonpea	0.30	2.09	0.78	1.29	0.84	2.27	8.16	3.25	2.83	2.84	4.20	5.67	2.34	1.14	7.14	0.98	0.89		0.56	0.91	0.51	6.90	0.00	0.00	0.81	0.37	1.77	0.91
Potato	0.00	0.41	0.55	0.00	0.34	1.08	1.00	0.00	0.75	0.00	0.00	0.52	1.10	0.95	1.04	0.00	0.61	0.56		0.00	0.40	0.00	0.00	0.00	0.00	0.00	0.67	2.93
Rice	0.37	2.27	1.19	0.00	0.90	0.59	2.27	0.66	0.48	1.35	0.57	1.15	1.50	2.35	1.16	0.97	1.57	0.91	0.00		1.99	0.57	0.00	0.32	0.44	0.21	3.00	0.47
Sorghum	0.20	3.42	0.50	0.44	1.29	1.47	0.46	0.31	0.70	1.94	1.40	2.35	1.00	3.58	1.89	2.30	3.80	0.51	0.40	1.99		1.38	0.00	0.00	0.00	0.00	3.00	0.68
Soybean	0.56	1.89	2.00	1.70	0.38	4.58	5.95	1.50	4.29	2.70	4.88	4.32	2.01	1.06	6.83	0.81	0.75	6.90	0.00	0.57	1.38		0.92	0.00	0.74	0.00	1.20	0.00
Sugar beet	0.00	0.00	0.00	0.00	0.00	0.00	0.00	0.00	0.00	0.00	0.00	0.00	0.00	0.00	0.00	0.00	0.00	0.00	0.00	0.00	0.00	0.92		0.00	0.00	0.00	0.00	0.00
Sugar Kelp	0.00	0.00	0.00	0.00	0.91	0.00	0.00	0.00	0.00	0.00	0.00	0.00	0.00	0.00	0.00	0.00	0.00	0.00	0.00	0.32	0.00	0.00	0.00		0.44	0.44	0.00	0.00
Sweetpota	0.43	0.34	0.00	0.36	2.20	0.78	1.11	0.54	0.00	0.55	0.00	0.38	0.40	0.00	0.38	0.00	0.00	0.81	0.00	0.44	0.00	0.74	0.00	0.44		0.00	0.19	0.58
Vitis	0.21	0.00	0.00	0.00	0.26	0.36	0.00	0.00	0.00	0.00	0.00	0.35	0.00	0.00	0.00	0.00	0.00	0.37	0.00	0.21	0.00	0.00	0.00	0.44	0.00		0.00	0.27
Wheat	0.16	6.06	1.25	0.47	0.39	1.99	1.91	1.55	1.24	1.77	1.21	1.46	2.01	4.31	3.41	2.68	0.78	1.77	0.67	3.00	3.00	1.20	0.00	0.00	0.19	0.00		0.81
Yam	0.00	0.00	0.89	0.00	1.19	1.75	0.41	0.00	1.62	1.17	0.84	0.84	0.45	1.39	1.69	0.00	0.48	0.91	2.93	0.47	0.68	0.00	0.00	0.00	0.58	0.27	0.81	
