# Supplementary material for: Challenges for FAIR-compliant description and comparison of crop phenotype data with standardized controlled vocabularies
Source: Database (Oxford). 2021 May 15;2021:baab028. doi: 10.1093/database/baab028 (PMC8122365; doi:10.1093/database/baab028)
Supplement: baab028_Supp [file baab028_supp.zip › SupplementalTable4.pdf]

"Curation;""Variable ID"";""Variable name"";""Variable synonyms"";""Context of use"";""Growth stage"";""Variable status"";""Variable Xref"";""Institution"";""Scientist"";""Date"";""Language"";""Crop"";""Trait ID"";""Trait name"";""Trait class"";""Trait description"";""Trait synonyms"";""Main trait abbreviation"";""Alternative trait abbreviations"";""Entity"";""Attribute"";""Trait status"";""Trait Xref"";""Method ID"";""Method name"";""Method class"";""Method description"";""Formula"";""Method reference"";""Scale ID"";""Scale name"";""Scale class"";""Decimal places"";""Lower limit"";""Upper limit"";""Scale Xref"";""Category 1"";""Category 2"";""Category 3"";""Category 4"";""Category 5"";""Category 6"";""Category 7"";""Category 8"";""Category 9"";""Category 10"";"
";""CO_366:0000021"";""PShelling_Cp_pct"";"""";""Trial evaluation"";""Post-Harvest"";"""";"""";""IBP"";""Julian Pietragalla"";"""";""EN"";""Bambara groundnut"";""CO_366:0000019"";""Pod shelling"";""Agronomical trait"";""The pod shelling."";"""";""PShelling"";"""";""Pod"";""shelling"";"""";"""";""CO_366:0000020"";""Pod shelling - Computation"";""Computation"";""Compute the pod shelling. Recorded within two months after harvest"   average percentage of 10 pods " based on weight of mature seed (at 12% moisture content)."";"""";"""";""CO_366:0000009"";""%"";""Numerical"";"""";""0.0"";""50.0"";"""";"
";""CO_366:0000024"";""SdFat_M_pct"";"""";""Trial evaluation"";""Post-Harvest"";"""";"""";""IBP"   " IPGRI"";""Julian Pietragalla"";"""";""EN"";""Bambara groundnut"";""CO_366:0000022"";""Seed fat content"";""Quality trait"";""The content of fat in seeds."";"""";""SdFat"";"""";""Seed"";""fat content"";"""";"""";""CO_366:0000023"";""Seed fat content - Measurement"";""Measurement"";""Standard chemical method for seed fat content method."";"""";"""";""CO_366:0000009"";""%"";""Numerical"";"""";""0.0"";""50.0"";"""";"
";""CO_366:0000027"";""SdProt_M_pct"";"""";""Trial evaluation"";""Post-Harvest"";"""";"""";""IBP"   " IPGRI"";""Julian Pietragalla"";"""";""EN"";""Bambara groundnut"";""CO_366:0000025"";""Seed protein content"";""Quality trait"";""The content of protein in seeds."";"""";""SdProt"";"""";""Seed"";""protein content"";"""";"""";""CO_366:0000026"";""Seed protein content - Measurement"";""Measurement"";""Standard chemical method for seed protein content method."";"""";"""";""CO_366:0000009"";""%"";""Numerical"";"""";""0.0"";""50.0"";"""";"
";""CO_366:0000031"";""StmHair_E_0to9"";"""";""Nursery evaluation"";""Harvest"";"""";"""";""IBP"   " IPGRI"";""Julian Pietragalla"";"""";""EN"";""Bambara groundnut"";""CO_366:0000028"";""Stem hairiness"";""Morphological trait"";""The stem hairiness."";"""";""StmHair"";"""";""Stem"";""hairiness"";"""";"""";""CO_366:0000029"";""Stem hairiness - Estimation"";""Estimation"";""Observe stem hairiness and rate it. Usually observed at harvest time."";"""";"""";""CO_366:0000030"";""0-9 Density scale"";""Ordinal"";"""";"""";"""";"""";""0= absent"";""1= slightly"";""2= slightly to sparse"";""3= sparse"";""4= sparse to moderately"";""5= moderately"";""6= moderately to dense"";""7= dense"";""8= highly dense"";""9= extremely dense"";"
";""CO_366:0000035"";""NoduCap_E_0to9"";"""";""Nursery evaluation"   " Trial evaluation"";"""";"""";"""";""IBP"   " IPGRI"";""Julian Pietragalla"   " Michael Abberton"";"""";""EN"";""Bambara groundnut"";""CO_366:0000032"";""Nodulation capacity"";""Physiological trait"";""The nodulation capacity of the plant."";"""";""NoduCap"";"""";""Plant"";""nodulation capacity"";"""";"""";""CO_366:0000033"";""Nodulation capacity - Estimation"";""Estimation"";""Observe nodulation capacity and rate it. Usually recorded 10 weeks after planting"   " average capacity of five healthy plants."";"""";"""";""CO_366:0000034"";""0-9 Nodulation capacity scale"";""Ordinal"";"""";"""";"""";"""";""0= none"";""1= very few nodules"";""2= very few to few nodules"";""3= few nodules"";""4= few to moderate nodules"";""5= moderate nodules"";""6= moderate to abundant nodules"";""7= abundant nodules"";""8= abundant to extremely abundant nodules"";""9= extremely abundant nodules"";"
";""CO_366:0000039"";""GH_E_1to3"";"""";""Trial evaluation"";"""";"""";"""";""IBP"   IITA   " IPGRI"";""Julian Pietragalla" Michael Abberton   " Sean Mayes"";"""";""EN"";""Bambara groundnut"";""CO_366:0000036"";""Growth habit"";""Morphological trait"";""A qualitative description of the growth habit of the plant."";"""";""GH"";"""";""Plant"";""growth habit"";"""";"""";""CO_366:0000037"";""Growth habit - Estimation"";""Estimation"";""Growth habit assessment. Usually recorded 10 weeks after planting" " based on the 4th petiole (P)/4th internode (I) length ratio (P/I)."";"""";"""";""CO_366:0000038"";""1-3 Growth habit scale"";""Nominal"";"""";"""";"""";"""";""1= Bunch type (P/I = >9)"";""2= Semibunch type (P/I = 7 - 9)"";""3= Spreading type (open) (P/I = <7)"";"
";""CO_366:0000043"";""SdSh_E_1to3"";"""";""Nursery evaluation" " Trial evaluation"";""Post-Harvest"";"""";"""";""IBP"   IITA   " IPGRI"";""Julian Pietragalla" " Michael Abberton"";"""";""EN"";""Bambara groundnut"";""CO_366:0000040"";""Seed shape"";""Morphological trait"";""The shape of seed."";"""";""SdSh"";"""";""Seed"";""shape"";"""";"""";""CO_366:0000041"";""Seed shape - Estimation"";""Estimation"";""Observe seed shape and rate it. Recorded on the basis of seeds from one-seeded pod"   " within two months after harvest."";"""";"""";""CO_366:0000042"";""1-3 Seed shape scale"";""Nominal"";"""";"""";"""";"""";""1= Round"";""2= Oval"";""99= Other (specify in descriptor Notes)"";"
";""CO_366:0000047"";""TLfltCol_E_1to4"";"""";""Nursery evaluation"";"""";"""";"""";""IBP"   IITA   " IPGRI"";""Julian Pietragalla" " Michael Abberton"";"""";""EN"";""Bambara groundnut"";""CO_366:0000044"";""Terminal leaflet colour"";""Morphological trait"";""The colour of the terminal leaflet."";"""";""TLfltCol"";"""";""Terminal leaflet"";""colour"";"""";"""";""CO_366:0000045"";""Terminal leaflet colour - Estimation"";""Estimation"";""Observe terminal leaflet colour and rate it. Usually observed on fully expanded terminal leaflet."";"""";"""";""CO_366:0000046"";""1-4 Leaflet colour scale"";""Nominal"";"""";"""";"""";"""";""1= Green"";""2= Red"";""3= Purple"";""99= Other (specify in descriptor Notes)"";"
";""CO_366:0000051"";""Ptext_E_1to4"";"""";""Nursery evaluation"   " Trial evaluation"";""Post-Harvest"";"""";"""";""IBP"   IITA   " IPGRI"";""Julian Pietragalla" " Michael Abberton"";"""";""EN"";""Bambara groundnut"";""CO_366:0000048"";""Pod texture"";""Morphological trait"";""The texture of pod."";"""";""Ptext"";"""";""Pod"";""texture"";"""";"""";""CO_366:0000049"";""Pod texture - Estimation"";""Estimation"";""Observe texture and rate it. Recorded within two months after harvest."";"""";"""";""CO_366:0000050"";""1-4 Pod texture scale"";""Nominal"";"""";"""";"""";"""";""1= Smooth"";""2= Little grooves"";""3= Much grooved"";""4= Much folded"";"
";""CO_366:0000055"";""TLfltSh_E_1to5"";"""";""Nursery evaluation"";"""";"""";"""";""IBP"   IITA   " IPGRI"";""Julian Pietragalla" Michael Abberton   " Sean Mayes"";"""";""EN"";""Bambara groundnut"";""CO_366:0000052"";""Terminal leaflet shape"";""Morphological trait"";""The shape of terminal leaflet."";"""";""TLfltSh"";"""";""Terminal leaflet"";""shape"";"""";"""";""CO_366:0000053"";""Terminal leaflet shape - Estimation"";""Estimation"";""Observe the shape of the terminal leaflet and rate it."";"""";"""";""CO_366:0000054"";""1-5 Leaflet shape scale"";""Nominal"";"""";"""";"""";"""";""1= Round"";""2= Oval"";""3= Lanceolate"";""4= Elliptic"";""99= Other (specify in descriptor Notes)"";"
";""CO_366:0000059"";""PSh_E_1to5"";"""";""Nursery evaluation" " Trial evaluation"";""Post-Harvest"";"""";"""";""IBP"   IITA   " IPGRI"";""Julian Pietragalla" " Michael Abberton"";"""";""EN"";""Bambara groundnut"";""CO_366:0000056"";""Pod shape"";""Morphological trait"";""The shape of pod."";"""";""PSh"";"""";""Pod"";""shape"";"""";"""";""CO_366:0000057"";""Pod shape - Estimation"";""Estimation"";""Observe pod shape and rate it. Recorded on the basis of one-seeded pod"   " within two months after harvest."";"""";"""";""CO_366:0000058"";""1-5 Pod shape scale"";""Nominal"";"""";"""";"""";"""";""1= Without point"";""2= Ending in a point" " round on the other side"";""3= Ending in a point"   " with nook on the other side"";""4= Ending in two points on each side"";""99= Other (specify in descriptor Notes)"";"
";""CO_366:0000063"";""PCol_E_1to6"";"""";""Nursery evaluation" " Trial evaluation"";""Post-Harvest"";"""";"""";""IBP" " IPGRI"";""Julian Pietragalla"";"""";""EN"";""Bambara groundnut"";""CO_366:0000060"";""Pod colour"";""Morphological trait"";""The colour of pod."";"""";""PCol"";"""";""Pod"";""colour"";"""";"""";""CO_366:0000061"";""Pod colour - Estimation"";""Estimation"";""Observe pod colour and rate it. Recorded within two months after harvest."";"""";"""";""CO_366:0000062"";""1-6 Pod colour scale"";""Nominal"";"""";"""";"""";"""";""1= Yellowish-brown"";""2= Brown"";""3= Reddish-brown"";""4= Purple"";""5= Black"";""99= Other (specify in descriptor Notes)"";"
";""CO_366:0000066"";""AlSus_E_1to9"";"""";""Research-intensive characterization"";"""";"""";"""";""IBP"   " IPGRI"";""Julian Pietragalla"";"""";""EN"";""Bambara groundnut"";""CO_366:0000064"";""Aluminium susceptibility"";""Abiotic stress trait"";""The plants susceptibility to Aluminium."";"""";""AlSus"";"""";""Plant"";""aluminium susceptibility"";"""";"""";""CO_366:0000065"";""Aluminium susceptibility - Estimation"";""Estimation"";""Observe the growth of plants per plot/entry in a high Aluminium soil and rate the susceptibility."";"""";"""";""CO_366:0000010"";""1-9 Susceptibility scale"";""Ordinal"";"""";"""";"""";"""";""1= Very low or no visible sign of susceptibility"";""2= Very low to low susceptibility"";""3= Low susceptibility"";""4= Low to intermediate susceptibility"";""5= Intermediate susceptibility"";""6= Intermediate to high susceptibility"";""7= High susceptibility"";""8= High to very high susceptibility"";""9= Very high susceptibility"";"
";""CO_366:0000069"";""DroSus_E_1to9"";"""";""Research-intensive characterization"";"""";"""";"""";""IBP"   " IPGRI"";""Julian Pietragalla"";"""";""EN"";""Bambara groundnut"";""CO_366:0000067"";""Drought susceptibility"";""Abiotic stress trait"";""The plants susceptibility to

drought stress."";"""";""DroSus"";"""";""Plant"";""drought susceptibility"";"""";"""";""CO_366:0000068"";""Drought susceptibility -
Estimation"";""Estimation"";""Observe the growth of plants per plot/entry under droughted condition and rate the
susceptibility."";"""";"""";""CO_366:0000010"";""1-9 Susceptibility scale"";""Ordinal"";"""";"""";"""";"""";""1= Very low or no visible sign of
susceptibility"";""2= Very low to low susceptibility"";""3= Low susceptibility"";""4= Low to intermediate susceptibility"";""5= Intermediate
susceptibility"";""6= Intermediate to high susceptibility"";""7= High susceptibility"";""8= High to very high susceptibility"";""9= Very high
susceptibility"";"
";""CO_366:0000072"";""FrostSus_E_1to9"";"""";""Research-intensive characterization"";"""";"""";"""";""IBP"   " IPGRI"";""Julian
Pietragalla"";"""";""EN"";""Bambara groundnut"";""CO_366:0000070"";""Frost damage"";""Abiotic stress trait"";""The plant susceptibility to
frost."";"""";""FrostSus"";"""";""Plant"";""frost susceptibility"";"""";"""";""CO_366:0000071"";""Frost susceptibility -
Estimation"";""Estimation"";""Observe the growth of plants per plot/entry after a frost event and rate the
susceptibility."";"""";"""";""CO_366:0000010"";""1-9 Susceptibility scale"";""Ordinal"";"""";"""";"""";"""";""1= Very low or no visible sign of
susceptibility"";""2= Very low to low susceptibility"";""3= Low susceptibility"";""4= Low to intermediate susceptibility"";""5= Intermediate
susceptibility"";""6= Intermediate to high susceptibility"";""7= High susceptibility"";""8= High to very high susceptibility"";""9= Very high
susceptibility"";"
";""CO_366:0000075"";""HTempSus_E_1to9"";"""";""Research-intensive characterization"";"""";"""";"""";""IBP"   " IPGRI"";""Julian
Pietragalla"";"""";""EN"";""Bambara groundnut"";""CO_366:0000073"";""High temperature susceptibility"";""Abiotic stress trait"";""The plants
susceptibility to high temperature conditions."";"""";""HTempSus"";"""";""Plant"";""high temperature
susceptibility"";"""";"""";""CO_366:0000074"";""High temperature susceptibility - Estimation"";""Estimation"";""Observe the growth of plants per
plot/entry under heat condition and rate the susceptibility."";"""";"""";""CO_366:0000010"";""1-9 Susceptibility
scale"";""Ordinal"";"""";"""";"""";"""";""1= Very low or no visible sign of susceptibility"";""2= Very low to low susceptibility"";""3= Low
susceptibility"";""4= Low to intermediate susceptibility"";""5= Intermediate susceptibility"";""6= Intermediate to high susceptibility"";""7= High
susceptibility"";""8= High to very high susceptibility"";""9= Very high susceptibility"";"
";""CO_366:0000078"";""SaltSus_E_1to9"";"""";""Research-intensive characterization"";"""";"""";"""";""IBP"   " IPGRI"";""Julian
Pietragalla"";"""";""EN"";""Bambara groundnut"";""CO_366:0000076"";""Salinity susceptibility"";""Abiotic stress trait"";""The plants susceptibility to
soil salinity."";"""";""SaltSus"";"""";""Plant"";""salinity susceptibility"";"""";"""";""CO_366:0000077"";""Salinity susceptibility -
Estimation"";""Estimation"";""Observe the growth of plants per plot/entry at the field presenting soil salinity and rate the
susceptibility."";"""";"""";""CO_366:0000010"";""1-9 Susceptibility scale"";""Ordinal"";"""";"""";"""";"""";""1= Very low or no visible sign of
susceptibility"";""2= Very low to low susceptibility"";""3= Low susceptibility"";""4= Low to intermediate susceptibility"";""5= Intermediate
susceptibility"";""6= Intermediate to high susceptibility"";""7= High susceptibility"";""8= High to very high susceptibility"";""9= Very high
susceptibility"";"
";""CO_366:0000081"";""AlteLfSpotSus_E_1to9"";"""";""Nursery evaluation"   " Trial evaluation"";"""";"""";"""";""IBP"";""Julian
Pietragalla"";"""";""EN"";""Bambara groundnut"";""CO_366:0000079"";""Alternaria leaf spot susceptibility"";""Biotic stress trait"";""Alternaria leaf spot
susceptibility caused by the agent Alternaria alternata."";"""";""AlteLfSpotSus"";"""";""Plant"";""Alternaria leaf spot
susceptibility"";"""";"""";""CO_366:0000080"";""Alternaria leaf spot susceptibility - Estimation"";""Estimation"";""Observe Alternaria leaf spot
susceptibility and rate it."";"""";"""";""CO_366:0000010"";""1-9 Susceptibility scale"";""Ordinal"";"""";"""";"""";"""";""1= Very low or no visible sign of
susceptibility"";""2= Very low to low susceptibility"";""3= Low susceptibility"";""4= Low to intermediate susceptibility"";""5= Intermediate
susceptibility"";""6= Intermediate to high susceptibility"";""7= High susceptibility"";""8= High to very high susceptibility"";""9= Very high
susceptibility"";"
";""CO_366:0000084"";""AscBlightSus_E_1to9"";"""";""Nursery evaluation" " Trial evaluation"";"""";"""";"""";""IBP"";""Julian
Pietragalla"";"""";""EN"";""Bambara groundnut"";""CO_366:0000082"";""Ascochyta blight susceptibility"";""Biotic stress trait"";""Ascochyta blight
susceptibility caused by the agent Ascochyta phaseolorum."";"""";""AscBlightSus"";"""";""Plant"";""Ascochyta blight
susceptibility"";"""";"""";""CO_366:0000083"";""Ascochyta blight susceptibility - Estimation"";""Estimation"";""Observe Ascochyta blight susceptibility
and rate it."";"""";"""";""CO_366:0000010"";""1-9 Susceptibility scale"";""Ordinal"";"""";"""";"""";"""";""1= Very low or no visible sign of
susceptibility"";""2= Very low to low susceptibility"";""3= Low susceptibility"";""4= Low to intermediate susceptibility"";""5= Intermediate
susceptibility"";""6= Intermediate to high susceptibility"";""7= High susceptibility"";""8= High to very high susceptibility"";""9= Very high
susceptibility"";"
";""CO_366:0000087"";""BCMVSus_E_1to9"";"""";""Nursery evaluation"   " Trial evaluation"";"""";"""";"""";""IBP"";""Julian
Pietragalla"";"""";""EN"";""Bambara groundnut"";""CO_366:0000085"";""BCMV susceptibility"";""Biotic stress trait"";""Bean common mosaic virus
(BCMV) susceptibility."";"""";""BCMVSus"";"""";""Plant"";""BCMV susceptibility"";"""";"""";""CO_366:0000086"";""BCMV susceptibility -
Estimation"";""Estimation"";""Observe BCMV susceptibility and rate it."";"""";"""";""CO_366:0000010"";""1-9 Susceptibility
scale"";""Ordinal"";"""";"""";"""";"""";""1= Very low or no visible sign of susceptibility"";""2= Very low to low susceptibility"";""3= Low
susceptibility"";""4= Low to intermediate susceptibility"";""5= Intermediate susceptibility"";""6= Intermediate to high susceptibility"";""7= High
susceptibility"";""8= High to very high susceptibility"";""9= Very high susceptibility"";"
";""CO_366:0000090"";""BeanBugSus_E_1to9"";"""";""Nursery evaluation"   " Trial evaluation"";"""";"""";"""";""IBP"";""Julian
Pietragalla"";"""";""EN"";""Bambara groundnut"";""CO_366:0000088"";""Bean bug susceptibility"";""Biotic stress trait"";""Bean bug (Clavigralla
tomentosicollis) susceptibility."";"""";""BeanBugSus"";"""";""Plant"";""Bean bug susceptibility"";"""";"""";""CO_366:0000089"";""Bean bug
susceptibility - Estimation"";""Estimation"";""Observe Bean bug susceptibility and rate it."";"""";"""";""CO_366:0000010"";""1-9 Susceptibility
scale"";""Ordinal"";"""";"""";"""";"""";""1= Very low or no visible sign of susceptibility"";""2= Very low to low susceptibility"";""3= Low
susceptibility"";""4= Low to intermediate susceptibility"";""5= Intermediate susceptibility"";""6= Intermediate to high susceptibility"";""7= High
susceptibility"";""8= High to very high susceptibility"";""9= Very high susceptibility"";"
";""CO_366:0000093"";""BruchidSus_E_1to9"";"""";""Nursery evaluation"   " Trial evaluation"";"""";"""";"""";""IBP"";""Julian
Pietragalla"";"""";""EN"";""Bambara groundnut"";""CO_366:0000091"";""Bruchid susceptibility"";""Biotic stress trait"";""Bruchid (Bruchidius
atrolineatus) susceptibility."";"""";""BruchidSus"";"""";""Plant"";""Bruchid susceptibility"";"""";"""";""CO_366:0000092"";""Bruchid susceptibility -
Estimation"";""Estimation"";""Observe Bruchid susceptibility and rate it."";"""";"""";""CO_366:0000010"";""1-9 Susceptibility
scale"";""Ordinal"";"""";"""";"""";"""";""1= Very low or no visible sign of susceptibility"";""2= Very low to low susceptibility"";""3= Low
susceptibility"";""4= Low to intermediate susceptibility"";""5= Intermediate susceptibility"";""6= Intermediate to high susceptibility"";""7= High
susceptibility"";""8= High to very high susceptibility"";""9= Very high susceptibility"";"
";""CO_366:0000096"";""BSMVSus_E_1to9"";"""";""Nursery evaluation"   " Trial evaluation"";"""";"""";"""";""IBP"";""Julian
Pietragalla"";"""";""EN"";""Bambara groundnut"";""CO_366:0000094"";""BSMV susceptibility"";""Biotic stress trait"";""Bean southern mosaic virus
(BSMV) susceptibility."";"""";""BSMVSus"";"""";""Plant"";""BSMV susceptibility"";"""";"""";""CO_366:0000095"";""BSMV susceptibility -
Estimation"";""Estimation"";""Observe BSMV susceptibility and rate it."";"""";"""";""CO_366:0000010"";""1-9 Susceptibility
scale"";""Ordinal"";"""";"""";"""";"""";""1= Very low or no visible sign of susceptibility"";""2= Very low to low susceptibility"";""3= Low
susceptibility"";""4= Low to intermediate susceptibility"";""5= Intermediate susceptibility"";""6= Intermediate to high susceptibility"";""7= High
susceptibility"";""8= High to very high susceptibility"";""9= Very high susceptibility"";"
";""CO_366:0000099"";""CABMVSus_E_1to9"";"""";""Nursery evaluation"   " Trial evaluation"";"""";"""";"""";""IBP"";""Julian
Pietragalla"";"""";""EN"";""Bambara groundnut"";""CO_366:0000097"";""CABMV susceptibility"";""Biotic stress trait"";""Cowpea aphid-borne mosaic
virus (CABMV) susceptibility."";"""";""CABMVSus"";"""";""Plant"";""CABMV susceptibility"";"""";"""";""CO_366:0000098"";""CABMV
susceptibility - Estimation"";""Estimation"";""Observe CABMV susceptibility and rate it."";"""";"""";""CO_366:0000010"";""1-9 Susceptibility
scale"";""Ordinal"";"""";"""";"""";"""";""1= Very low or no visible sign of susceptibility"";""2= Very low to low susceptibility"";""3= Low
susceptibility"";""4= Low to intermediate susceptibility"";""5= Intermediate susceptibility"";""6= Intermediate to high susceptibility"";""7= High
susceptibility"";""8= High to very high susceptibility"";""9= Very high susceptibility"";"
";""CO_366:0000102"";""CercLfSpotSus_E_1to9"";"""";""Nursery evaluation"" Trial evaluation"";"""";"""";"""";""IBP"   " IITA"";""Julian Pietragalla"

" Michael Abberton"";"""";""EN"";""Bambara groundnut"";""CO_366:0000100"";""Cercospora leaf spot susceptibility"";""Biotic stress
trait"";""Cercospora leaf spot susceptibility caused by the agent Cercospora canescens."";"""";""CercLfSpotSus"";"""";""Plant"";""Cercospora leaf spot
susceptibility"";"""";"""";""CO_366:0000101"";""Cercospora leaf spot susceptibility - Estimation"";""Estimation"";""Observe Cercospora leaf spot
susceptibility and rate it."";"""";"""";""CO_366:0000010"";""1-9 Susceptibility scale"";""Ordinal"";"""";"""";"""";"""";""1= Very low or no visible sign of
susceptibility"";""2= Very low to low susceptibility"";""3= Low susceptibility"";""4= Low to intermediate susceptibility"";""5= Intermediate
susceptibility"";""6= Intermediate to high susceptibility"";""7= High susceptibility"";""8= High to very high susceptibility"";""9= Very high
susceptibility"";"
";""CO_366:0000105"";""ChiBruchidSus_E_1to9"";"""";""Nursery evaluation"" Trial evaluation"";"""";"""";"""";""IBP"";""Julian
Pietragalla"";"""";""EN"";""Bambara groundnut"";""CO_366:0000103"";""Chinese bruchid susceptibility"";""Biotic stress trait"";""Chinese bruchid
(Callosobruchus chinensis) susceptibility."";"""";""ChiBruchidSus"";"""";""Plant"";""Chinese bruchid
susceptibility"";"""";"""";""CO_366:0000104"";""Chinese bruchid susceptibility - Estimation"";""Estimation"";""Observe Chinese bruchid susceptibility
and rate it."";"""";"""";""CO_366:0000010"";""1-9 Susceptibility scale"";""Ordinal"";"""";"""";"""";"""";""1= Very low or no visible sign of
susceptibility"";""2= Very low to low susceptibility"";""3= Low susceptibility"";""4= Low to intermediate susceptibility"";""5= Intermediate
susceptibility"";""6= Intermediate to high susceptibility"";""7= High susceptibility"";""8= High to very high susceptibility"";""9= Very high
susceptibility"";"
";""CO_366:0000108"";""CMVSus_E_1to9"";"""";""Nursery evaluation" " Trial evaluation"";"""";"""";"""";""IBP"";""Julian
Pietragalla"";"""";""EN"";""Bambara groundnut"";""CO_366:0000106"";""CMV susceptibility"";""Biotic stress trait"";""Cucumber mosaic virus (CMV)
susceptibility."";"""";""CMVSus"";"""";""Plant"";""CMV susceptibility"";"""";"""";""CO_366:0000107"";""CMV susceptibility -
Estimation"";""Estimation"";""Observe CMV susceptibility and rate it."";"""";"""";""CO_366:0000010"";""1-9 Susceptibility
scale"";""Ordinal"";"""";"""";"""";"""";""1= Very low or no visible sign of susceptibility"";""2= Very low to low susceptibility"";""3= Low
susceptibility"";""4= Low to intermediate susceptibility"";""5= Intermediate susceptibility"";""6= Intermediate to high susceptibility"";""7= High
susceptibility"";""8= High to very high susceptibility"";""9= Very high susceptibility"";"
";""CO_366:0000111"";""CocoaWvlSus_E_1to9"";"""";""Nursery evaluation" " Trial evaluation"";"""";"""";"""";""IBP"";""Julian
Pietragalla"";"""";""EN"";""Bambara groundnut"";""CO_366:0000109"";""Cocoa weevil susceptibility"";""Biotic stress trait"";""Cocoa weevil (Araecerus
fasciculatus) susceptibility."";"""";""CocoaWvlSus"";"""";""Plant"";""Cocoa weevil susceptibility"";"""";"""";""CO_366:0000110"";""Cocoa weevil
susceptibility - Estimation"";""Estimation"";""Observe Cocoa weevil susceptibility and rate it."";"""";"""";""CO_366:0000010"";""1-9 Susceptibility
scale"";""Ordinal"";"""";"""";"""";"""";""1= Very low or no visible sign of susceptibility"";""2= Very low to low susceptibility"";""3= Low
susceptibility"";""4= Low to intermediate susceptibility"";""5= Intermediate susceptibility"";""6= Intermediate to high susceptibility"";""7= High
susceptibility"";""8= High to very high susceptibility"";""9= Very high susceptibility"";"
";""CO_366:0000114"";""CowpeaWvilSus_E_1to9"";"""";""Nursery evaluation" " Trial evaluation"";"""";"""";"""";""IBP"";""Julian
Pietragalla"";"""";""EN"";""Bambara groundnut"";""CO_366:0000112"";""Cowpea weevil susceptibility"";""Biotic stress trait"";""Cowpea weevil
(Callosobruchus maculatus) susceptibility."";"""";""CowpeaWvilSus"";"""";""Plant"";""Cowpea weevil
susceptibility"";"""";"""";""CO_366:0000113"";""Cowpea weevil susceptibility - Estimation"";""Estimation"";""Observe Cowpea weevil susceptibility
and rate it."";"""";"""";""CO_366:0000010"";""1-9 Susceptibility scale"";""Ordinal"";"""";"""";"""";"""";""1= Very low or no visible sign of
susceptibility"";""2= Very low to low susceptibility"";""3= Low susceptibility"";""4= Low to intermediate susceptibility"";""5= Intermediate
susceptibility"";""6= Intermediate to high susceptibility"";""7= High susceptibility"";""8= High to very high susceptibility"";""9= Very high
susceptibility"";"
";""CO_366:0000117"";""CPMMVSus_E_1to9"";"""";""Nursery evaluation" " Trial evaluation"";"""";"""";"""";""IBP"";""Julian
Pietragalla"";"""";""EN"";""Bambara groundnut"";""CO_366:0000115"";""CPMMV susceptibility"";""Biotic stress trait"";""Cowpea mild mottle virus
(CPMMV) susceptibility."";"""";""CPMMVSus"";"""";""Plant"";""CPMMV susceptibility"";"""";"""";""CO_366:0000116"";""CPMMV susceptibility -
Estimation"";""Estimation"";""Observe CPMMV susceptibility and rate it."";"""";"""";""CO_366:0000010"";""1-9 Susceptibility
scale"";""Ordinal"";"""";"""";"""";"""";""1= Very low or no visible sign of susceptibility"";""2= Very low to low susceptibility"";""3= Low
susceptibility"";""4= Low to intermediate susceptibility"";""5= Intermediate susceptibility"";""6= Intermediate to high susceptibility"";""7= High
susceptibility"";""8= High to very high susceptibility"";""9= Very high susceptibility"";"
";""CO_366:0000120"";""CPMoVSus_E_1to9"";"""";""Nursery evaluation" " Trial evaluation"";"""";"""";"""";""IBP"";""Julian
Pietragalla"";"""";""EN"";""Bambara groundnut"";""CO_366:0000118"";""CPMoV susceptibility"";""Biotic stress trait"";""Cowpea mottle virus
(CPMoV) susceptibility."";"""";""CPMoVSus"";"""";""Plant"";""CPMoV susceptibility"";"""";"""";""CO_366:0000119"";""CPMoV susceptibility -
Estimation"";""Estimation"";""Observe CPMoV susceptibility and rate it."";"""";"""";""CO_366:0000010"";""1-9 Susceptibility
scale"";""Ordinal"";"""";"""";"""";"""";""1= Very low or no visible sign of susceptibility"";""2= Very low to low susceptibility"";""3= Low
susceptibility"";""4= Low to intermediate susceptibility"";""5= Intermediate susceptibility"";""6= Intermediate to high susceptibility"";""7= High
susceptibility"";""8= High to very high susceptibility"";""9= Very high susceptibility"";"
";""CO_366:0000123"";""CPMVSus_E_1to9"";"""";""Nursery evaluation" " Trial evaluation"";"""";"""";"""";""IBP"";""Julian
Pietragalla"";"""";""EN"";""Bambara groundnut"";""CO_366:0000121"";""CPMV susceptibility"";""Biotic stress trait"";""Cowpea mosaic virus (CPMV)
susceptibility."";"""";""CPMVSus"";"""";""Plant"";""CPMV susceptibility"";"""";"""";""CO_366:0000122"";""CPMV susceptibility -
Estimation"";""Estimation"";""Observe CPMV susceptibility and rate it."";"""";"""";""CO_366:0000010"";""1-9 Susceptibility
scale"";""Ordinal"";"""";"""";"""";"""";""1= Very low or no visible sign of susceptibility"";""2= Very low to low susceptibility"";""3= Low
susceptibility"";""4= Low to intermediate susceptibility"";""5= Intermediate susceptibility"";""6= Intermediate to high susceptibility"";""7= High
susceptibility"";""8= High to very high susceptibility"";""9= Very high susceptibility"";"
";""CO_366:0000126"";""DidyLfSpotSus_E_1to9"";"""";""Nursery evaluation" " Trial evaluation"";"""";"""";"""";""IBP"";""Julian
Pietragalla"";"""";""EN"";""Bambara groundnut"";""CO_366:0000124"";""Didymella leaf spot susceptibility"";""Biotic stress trait"";""Didymella leaf
spot susceptibility caused by the agent Didymella pinodes."";"""";""DidyLfSpotSus"";"""";""Plant"";""Didymella leaf spot
susceptibility"";"""";"""";""CO_366:0000125"";""Didymella leaf spot susceptibility - Estimation"";""Estimation"";""Observe Didymella leaf spot
susceptibility and rate it."";"""";"""";""CO_366:0000010"";""1-9 Susceptibility scale"";""Ordinal"";"""";"""";"""";"""";""1= Very low or no visible sign of
susceptibility"";""2= Very low to low susceptibility"";""3= Low susceptibility"";""4= Low to intermediate susceptibility"";""5= Intermediate
susceptibility"";""6= Intermediate to high susceptibility"";""7= High susceptibility"";""8= High to very high susceptibility"";""9= Very high
susceptibility"";"
";""CO_366:0000129"";""FusaWiltSus_E_1to9"";"""";""Nursery evaluation" " Trial evaluation"";"""";"""";"""";""IBP"";""Julian
Pietragalla"";"""";""EN"";""Bambara groundnut"";""CO_366:0000127"";""Fusarium wilt susceptibility"";""Biotic stress trait"";""Fusarium wilt
susceptibility caused by the agent Fusarium oxysporum."";"""";""FusaWiltSus"";"""";""Plant"";""Fusarium wilt
susceptibility"";"""";"""";""CO_366:0000128"";""Fusarium wilt susceptibility - Estimation"";""Estimation"";""Observe Fusarium wilt susceptibility and
rate it."";"""";"""";""CO_366:0000010"";""1-9 Susceptibility scale"";""Ordinal"";"""";"""";"""";"""";""1= Very low or no visible sign of
susceptibility"";""2= Very low to low susceptibility"";""3= Low susceptibility"";""4= Low to intermediate susceptibility"";""5= Intermediate
susceptibility"";""6= Intermediate to high susceptibility"";""7= High susceptibility"";""8= High to very high susceptibility"";""9= Very high
susceptibility"";"
";""CO_366:0000132"";""PeMoVSus_E_1to9"";"""";""Nursery evaluation" " Trial evaluation"";"""";"""";"""";""IBP"";""Julian
Pietragalla"";"""";""EN"";""Bambara groundnut"";""CO_366:0000130"";""PeMoV susceptibility"";""Biotic stress trait"";""Peanut mottle virus (PeMoV)
susceptibility."";"""";""PeMoVSus"";"""";""Plant"";""PeMoV susceptibility"";"""";"""";""CO_366:0000131"";""PeMoV susceptibility -
Estimation"";""Estimation"";""Observe PeMoV susceptibility and rate it."";"""";"""";""CO_366:0000010"";""1-9 Susceptibility
scale"";""Ordinal"";"""";"""";"""";"""";""1= Very low or no visible sign of susceptibility"";""2= Very low to low susceptibility"";""3= Low
susceptibility"";""4= Low to intermediate susceptibility"";""5= Intermediate susceptibility"";""6= Intermediate to high susceptibility"";""7= High
susceptibility"";""8= High to very high susceptibility"";""9= Very high susceptibility"";"

";""CO_366:0000135"";""PhylLfSpotSus_E_1to9"";"""";""Nursery evaluation"" Trial evaluation"";"""";"""";"""";""IBP"";""Julian Pietragalla"";"""";""EN"";""Bambara groundnut"";""CO_366:0000133"";""Phyllosticta leaf spot susceptibility"";""Biotic stress trait"";""Phyllosticta leaf spot susceptibility caused by the agent Phyllosticta voandzeiae."";"""";""PhylLfSpotSus"";"""";""Plant"";""Phyllosticta leaf spot susceptibility"";"""";"""";""CO_366:0000134"";""Phyllosticta leaf spot susceptibility - Estimation"";""Estimation"";""Observe Phyllosticta leaf spot susceptibility and rate it."";"""";"""";""CO_366:0000010"";""1-9 Susceptibility scale"";""Ordinal"";"""";"""";"""";"""";""1= Very low or no visible sign of susceptibility"";""2= Very low to low susceptibility"";""3= Low susceptibility"";""4= Low to intermediate susceptibility"";""5= Intermediate susceptibility"";""6= Intermediate to high susceptibility"";""7= High susceptibility"";""8= High to very high susceptibility"";""9= Very high susceptibility"";"
";""CO_366:0000138"";""PowMildewSus_E_1to9"";"""";""Nursery evaluation"" Trial evaluation"";"""";"""";"""";""IBP"";""Julian Pietragalla"";"""";""EN"";""Bambara groundnut"";""CO_366:0000136"";""Powdery mildew susceptibility"";""Biotic stress trait"";""Powdery mildew susceptibility caused by the agent Sphaerotheca voandzeiae."";"""";""PowMildewSus"";"""";""Plant"";""Powdery mildew susceptibility"";"""";"""";""CO_366:0000137"";""Powdery mildew susceptibility - Estimation"";""Estimation"";""Observe Powdery mildew susceptibility and rate it."";"""";"""";""CO_366:0000010"";""1-9 Susceptibility scale"";""Ordinal"";"""";"""";"""";"""";""1= Very low or no visible sign of susceptibility"";""2= Very low to low susceptibility"";""3= Low susceptibility"";""4= Low to intermediate susceptibility"";""5= Intermediate susceptibility"";""6= Intermediate to high susceptibility"";""7= High susceptibility"";""8= High to very high susceptibility"";""9= Very high susceptibility"";"
";""CO_366:0000141"";""RootKnotSus_E_1to9"";"""";""Nursery evaluation"" Trial evaluation"";"""";"""";"""";""IBP"";""Julian Pietragalla"";"""";""EN"";""Bambara groundnut"";""CO_366:0000139"";""Root knot nematode susceptibility"";""Biotic stress trait"";""Root knot nematode (Meloidogyne spp.) susceptibility."";"""";""RootKnotSus"";"""";""Plant"";""Root knot nematode susceptibility"";"""";"""";""CO_366:0000140"";""Root knot nematode susceptibility - Estimation"";""Estimation"";""Observe Root knot nematode susceptibility and rate it."";"""";"""";""CO_366:0000010"";""1-9 Susceptibility scale"";""Ordinal"";"""";"""";"""";"""";""1= Very low or no visible sign of susceptibility"";""2= Very low to low susceptibility"";""3= Low susceptibility"";""4= Low to intermediate susceptibility"";""5= Intermediate susceptibility"";""6= Intermediate to high susceptibility"";""7= High susceptibility"";""8= High to very high susceptibility"";""9= Very high susceptibility"";"
";""CO_366:0000144"";""SclerRootRotSus_E_1to9"";"""";""Nursery evaluation"" Trial evaluation"";"""";"""";"""";""IBP"";""Julian Pietragalla"";"""";""EN"";""Bambara groundnut"";""CO_366:0000142"";""Sclerotium root rot susceptibility"";""Biotic stress trait"";""Sclerotium root rot susceptibility caused by the agent Sclerotium rolfsii."";"""";""SclerRootRotSus"";"""";""Plant"";""Sclerotium root rot susceptibility"";"""";"""";""CO_366:0000143"";""Sclerotium root rot susceptibility - Estimation"";""Estimation"";""Observe Sclerotium root rot susceptibility and rate it."";"""";"""";""CO_366:0000010"";""1-9 Susceptibility scale"";""Ordinal"";"""";"""";"""";"""";""1= Very low or no visible sign of susceptibility"";""2= Very low to low susceptibility"";""3= Low susceptibility"";""4= Low to intermediate susceptibility"";""5= Intermediate susceptibility"";""6= Intermediate to high susceptibility"";""7= High susceptibility"";""8= High to very high susceptibility"";""9= Very high susceptibility"";"
";""CO_366:0000147"";""SuckingBugSus_E_1to9"";"""";""Nursery evaluation"" Trial evaluation"";"""";"""";"""";""IBP"";""Julian Pietragalla"";"""";""EN"";""Bambara groundnut"";""CO_366:0000145"";""Sucking bug susceptibility"";""Biotic stress trait"";""Sucking bug (Agonoscelis sp.) susceptibility."";"""";""SuckingBugSus"";"""";""Plant"";""Sucking bug susceptibility"";"""";"""";""CO_366:0000146"";""Sucking bug susceptibility - Estimation"";""Estimation"";""Observe Sucking bug susceptibility and rate it."";"""";"""";""CO_366:0000010"";""1-9 Susceptibility scale"";""Ordinal"";"""";"""";"""";"""";""1= Very low or no visible sign of susceptibility"";""2= Very low to low susceptibility"";""3= Low susceptibility"";""4= Low to intermediate susceptibility"";""5= Intermediate susceptibility"";""6= Intermediate to high susceptibility"";""7= High susceptibility"";""8= High to very high susceptibility"";""9= Very high susceptibility"";"
";""CO_366:0000150"";""VNMVSus_E_1to9"";"""";""Nursery evaluation"" Trial evaluation"";"""";"""";"""";""IBP"";""Julian Pietragalla"";"""";""EN"";""Bambara groundnut"";""CO_366:0000148"";""VNMV susceptibility"";""Biotic stress trait"";""Voandzeia necrotic mosaic virus (VNMV) susceptibility."";"""";""VNMVSus"";"""";""Plant"";""VNMV susceptibility"";"""";"""";""CO_366:0000149"";""VNMV susceptibility - Estimation"";""Estimation"";""Observe VNMV susceptibility and rate it."";"""";"""";""CO_366:0000010"";""1-9 Susceptibility scale"";""Ordinal"";"""";"""";"""";"""";""1= Very low or no visible sign of susceptibility"";""2= Very low to low susceptibility"";""3= Low susceptibility"";""4= Low to intermediate susceptibility"";""5= Intermediate susceptibility"";""6= Intermediate to high susceptibility"";""7= High susceptibility"";""8= High to very high susceptibility"";""9= Very high susceptibility"";"
";""CO_366:0000154"";""StmBrnchN_Ct_brnch"";"""";""Trial evaluation"";""Harvest"";"""";"""";""IBP" " IITA"";""Julian Pietragalla"" Michael Abberton"";"""";""EN"";""Bambara groundnut"";""CO_366:0000151"";""Branch number per stem"";""Morphological trait"";""The branches number per stem."";"""";""StmBrnchN"";"""";""Stem"";""branch number"";"""";"""";""CO_366:0000152"";""Branch number - Counting"";""Counting"";""Count branches in a stem. Usually recorded at harvest" " average number of three stems of five healthy plants."";"""";"""";""CO_366:0000153"";""branch"";""Numerical"";"""";"""";"""";"""";"
";""CO_366:0000157"";""PlntH_M_cm"";"""";""Nursery evaluation"" Trial evaluation"";"""";"""";"""";""IBP" IPGRI IITA " UoN"";""Julian Pietragalla" Michael Abberton " Sean Mayes"";"""";""EN"";""Bambara groundnut"";""CO_366:0000155"";""Plant height"";""Morphological trait"";""The height of the plant."";"""";""PlntH"";"""";""Plant"";""height"";"""";"""";""CO_366:0000156"";""Plant height - Measurement"";""Measurement"";""Measure plant height and record it. Measured from the ground level (at the base of the plant) to the tip of the highest point" including the terminal leaflet. Usually recorded 10 weeks after planting " average height of five plants."";"""";"""";""CO_366:0000011"";""cm"";""Numerical"";"""";"""";"""";"""";"
";""CO_366:0000160"";""PlntSpread_M_cm"";"""";""Nursery evaluation"" Trial evaluation"";"""";"""";"""";""IBP" " UoN"";""Julian Pietragalla" " Sean Mayes"";"""";""EN"";""Bambara groundnut"";""CO_366:0000158"";""Plant spread"";""Morphological trait"";""The spread of the plant."";"""";""PlntSpread"";"""";""Plant"";""spread"";"""";"""";""CO_366:0000159"";""Plant spread - Measurement"";""Measurement"";""Measure plant spread and record it. Usually recorded 10 weeks after planting" " average of five plants. Widest length between two opposite points."";"""";"""";""CO_366:0000011"";""cm"";""Numerical"";"""";"""";"""";"""";"
";""CO_366:0000162"";""Emer_Date_ymd"";"""";""Nursery evaluation" " Trial evaluation"";""Emergence"";"""";"""";""IBP"";""Julian Pietragalla"";"""";""EN"";""Bambara groundnut"";""CO_366:0000003"";""Emergence time"";""Phenological trait"";""Emergence time is when the cotyledon appears above the soil surface."";"""";""Emer"";"""";""Plant"";""emergence time"";"""";"""";""CO_366:0000161"";""Emergence date - Estimation"";""Estimation"";""Record date of emergence."";"""";"""";""CO_366:0000012"";""Date (yyyymmdd)"";""Time"";"""";"""";"""";"""";"
";""CO_366:0000164"";""Flw_DateFirst_ymd"";"""";""Nursery evaluation" " Trial evaluation"";""Flowering"";"""";"""";""IBP"";""Julian Pietragalla"";"""";""EN"";""Bambara groundnut"";""CO_366:0000004"";""Flowering time"";""Phenological trait"";""Flowering time."";"""";""Flw"";"""";""Plant"";""flowering time"";"""";"""";""CO_366:0000163"";""First flowering date - Estimation"";""Estimation"";""Record date of first flower."";"""";"""";""CO_366:0000012"";""Date (yyyymmdd)"";""Time"";"""";"""";"""";"""";"
";""CO_366:0000166"";""Flw_DateHalf_ymd"";"""";""Nursery evaluation" " Trial evaluation"";""Flowering"";"""";"""";""IBP"";""Julian Pietragalla"";"""";""EN"";""Bambara groundnut"";""CO_366:0000004"";""Flowering time"";""Phenological trait"";""Flowering time."";"""";""Flw"";"""";""Plant"";""flowering time"";"""";"""";""CO_366:0000165"";""Half flowering date - Estimation"";""Estimation"";""Record date when 50 percent of the plants begun to flower."";"""";"""";""CO_366:0000012"";""Date (yyyymmdd)"";""Time"";"""";"""";"""";"""";"
";""CO_366:0000168"";""Mat_Date_ymd"";"""";""Nursery evaluation" " Trial evaluation"";""Maturity"";"""";"""";""IBP"";""Julian Pietragalla"";"""";""EN"";""Bambara groundnut"";""CO_366:0000005"";""Maturity time"";""Phenological trait"";""Maturity time."";"""";""Mat"";"""";""Plant"";""maturity time"";"""";"""";""CO_366:0000167"";""Maturity date - Estimation"";""Estimation"";""Record date of maturity."";"""";"""";""CO_366:0000012"";""Date (yyyymmdd)"";""Time"";"""";"""";"""";"""";"
";""CO_366:0000170"";""Emer_DT_day"";"""";""Nursery evaluation" " Trial evaluation"";""Emergence"";"""";"""";""IBP" IITA " UoN"";""Julian Pietragalla" Michael Abberton " Sean Mayes"";"""";""EN"";""Bambara groundnut"";""CO_366:0000003"";""Emergence time"";""Phenological trait"";""Emergence time is when the cotyledon appears above the soil surface."";"""";""Emer"";"""";""Plant"";""emergence time"";"""";"""";""CO_366:0000169"";""Days to emergence - Computation"";""Computation"";""Compute number of days required from sowing to

emergence."";""""Emer_Date_ymd"" - ""Planting_date_ymd"""";"""";""CO_366:0000013"";""day"";""Duration"";"""";"""";"""";"""";"
";""CO_366:0000172"";""Flw_DTFirst_day"";"""";""Nursery evaluation" " Trial evaluation"";""Flowering"";"""";"""";""IBP" " IITA"";""Julian
Pietragalla" " Michael Abberton"";"""";""EN"";""Bambara groundnut"";""CO_366:0000004"";""Flowering time"";""Phenological trait"";""Flowering
time."";"""";""Flw"";"""";""Plant"";""flowering time"";"""";"""";""CO_366:0000171"";""Days to first flowering -
Computation"";""Computation"";""Compute number of days required from sowing (or emergence) to first flower."";""""Flw_Date_ymd"" -
""Emer_Date_ymd"" or ""Flw_Date_ymd"" - ""Planting_date_ymd"""";"""";""CO_366:0000013"";""day"";""Duration"";"""";"""";"""";"""";"
";""CO_366:0000174"";""Flw_DTHalf_day"";"""";""Nursery evaluation" " Trial evaluation"";""Flowering"";"""";"""";""IBP"";""Julian
Pietragalla"";"""";""EN"";""Bambara groundnut"";""CO_366:0000004"";""Flowering time"";""Phenological trait"";""Flowering
time."";"""";""Flw"";"""";""Plant"";""flowering time"";"""";"""";""CO_366:0000173"";""Days to half flowering -
Computation"";""Computation"";""Compute number of days required from sowing (or emergence) to when 50 percent of the plants begun to
flower."";""""Flw_DateHalf_ymd"" - ""Emer_Date_ymd"" or ""Flw_DateHalf_ymd"" -
""Planting_date_ymd"""";"""";""CO_366:0000013"";""day"";""Duration"";"""";"""";"""";"""";"
";""CO_366:0000176"";""Mat_DT_day"";"""";""Nursery evaluation" " Trial evaluation"";""Maturity"";"""";"""";""IBP" IPGRI IITA "
UoN"";""Julian Pietragalla" Michael Abberton " Sean Mayes"";"""";""EN"";""Bambara groundnut"";""CO_366:0000005"";""Maturity
time"";""Phenological trait"";""Maturity time."";"""";""Mat"";"""";""Plant"";""maturity time"";"""";"""";""CO_366:0000175"";""Days to maturity -
Computation"";""Computation"";""Compute number of days required from sowing (or emergence) to maturity."";""""Mat_Date_ymd"" -
""Emer_Date_ymd"" or ""Mat_Date_ymd"" - ""Planting_date_ymd"""";"""";""CO_366:0000013"";""day"";""Duration"";"""";"""";"""";"""";"
";""CO_366:0000180"";""PedFlwN_Ct_flw"";"""";""Nursery evaluation" " Trial evaluation"";"""";"""";"""";""IBP" IITA " IPGRI"";""Julian
Pietragalla" " Michael Abberton"";"""";""EN"";""Bambara groundnut"";""CO_366:0000177"";""Flower number per peduncle"";""Morphological
trait"";""The flowers number per peduncle."";"""";""PedFlwN"";"""";""Peduncle"";""flower number"";"""";"""";""CO_366:0000178"";""Flower number -
Counting"";""Counting"";""Count flowers in a sampling unit (plot" plant branch peduncle "
etc)."";"""";"""";""CO_366:0000179"";""flower"";""Numerical"";"""";"""";"""";"""";"
";""CO_366:0000182"";""HSdAdjW_M_g"";"""";""Trial evaluation"";""Post-Harvest"";"""";"""";""IBP" IITA IPGRI " UoN"";""Julian
Pietragalla" Michael Abberton " Sean Mayes"";"""";""EN"";""Bambara groundnut"";""CO_366:0000181"";""Hundred seed adjusted
weight"";""Agronomical trait"";""The adjusted weight (at 12% moisture content) of 100 seeds."";"""";""HSdAdjW"";"""";""Seed"";""adjusted
weight"";"""";"""";""CO_366:0000006"";""Seed Weight - Measurement"";""Measurement"";""Count and weigh seed per sampling unit (single" 100 200
" 1000)."";"""";"""";""CO_366:0000014"";""g"";""Numerical"";"""";"""";"""";"""";"
";""CO_366:0000184"";""HSdDW_M_g"";"""";""Trial evaluation"";""Post-Harvest"";"""";"""";""IBP"";""Julian Pietragalla"";"""";""EN"";""Bambara
groundnut"";""CO_366:0000183"";""Hundred seed dry weight"";""Agronomical trait"";""The dry weight of 100
seeds."";"""";""HSdDW"";"""";""Seed"";""dry weight"";"""";"""";""CO_366:0000006"";""Seed Weight - Measurement"";""Measurement"";""Count and
weigh seed per sampling unit (single" 100 200 " 1000)."";"""";"""";""CO_366:0000014"";""g"";""Numerical"";"""";"""";"""";"""";"
";""CO_366:0000186"";""SdMoist_M_pct"";"""";""Trial evaluation"";""Harvest"";"""";"""";""IBP"";""Julian Pietragalla"";"""";""EN"";""Bambara
groundnut"";""CO_366:0000185"";""Seed moisture content"";""Agronomical trait"";""The moisture content of the
seed."";"""";""SdMoist"";"""";""Seed"";""moisture"";"""";"""";""CO_366:0000007"";""Seed yield adjusted weight basis -
Computing"";""Computation"";""Compute adjusted weight of harvested seed per
area."";"""";"""";""CO_366:0000015"";""g/m2"";""Numerical"";"""";"""";"""";"""";"
";""CO_366:0000187"";""SdYAdjW_Cp_gm2"";"""";""Trial evaluation"";""Harvest"";"""";"""";""IBP"";""Julian Pietragalla"";"""";""EN"";""Bambara
groundnut"";""CO_366:0000001"";""Seed yield adjusted weight basis"";""Agronomical trait"";""The amount in adjusted weight basis (at 12% moisture
content) of seed that was harvested per unit area or plant."";"""";""SdYAdjW"";"""";""Seed"";""yield adjusted weight
basis"";"""";"""";""CO_366:0000007"";""Seed yield adjusted weight basis - Computing"";""Computation"";""Compute adjusted weight of harvested seed
per area."";"""";"""";""CO_366:0000015"";""g/m2"";""Numerical"";"""";"""";"""";"""";"
";""CO_366:0000189"";""SdYFW_M_gplant"";"""";""Trial evaluation"";""Harvest"";"""";"""";""IBP"";""Julian Pietragalla"";"""";""EN"";""Bambara
groundnut"";""CO_366:0000002"";""Seed yield fresh weight basis"";""Agronomical trait"";""The amount in fresh weight basis of seed that was harvested
per unit area or plant."";"""";""SdYFW"";"""";""Seed"";""yield"";"""";"""";""CO_366:0000008"";""Seed yield FW basis -
Measurement"";""Measurement"";""Record fresh weight (FW) of harvested seed per sampling unit (plot" plant branch raceme "
etc)."";"""";"""";""CO_366:0000188"";""g/plant"";""Numerical"";"""";"""";"""";"""";"
";""CO_366:0000191"";""SdYFW_M_gplot"";"""";""Trial evaluation"";""Harvest"";"""";"""";""IBP"";""Julian Pietragalla"";"""";""EN"";""Bambara
groundnut"";""CO_366:0000002"";""Seed yield fresh weight basis"";""Agronomical trait"";""The amount in fresh weight basis of seed that was harvested
per unit area or plant."";"""";""SdYFW"";"""";""Seed"";""yield"";"""";"""";""CO_366:0000008"";""Seed yield FW basis -
Measurement"";""Measurement"";""Record fresh weight (FW) of harvested seed per sampling unit (plot" plant branch raceme "
etc)."";"""";"""";""CO_366:0000190"";""g/plot"";""Numerical"";"""";"""";"""";"""";"
";""CO_366:0000193"";""SdYAdjW_Cp_kgha"";"""";""Trial evaluation"";""Harvest"";"""";"""";""IBP" " IPGRI"";""Julian
Pietragalla"";"""";""EN"";""Bambara groundnut"";""CO_366:0000001"";""Seed yield adjusted weight basis"";""Agronomical trait"";""The amount in
adjusted weight basis (at 12% moisture content) of seed that was harvested per unit area or plant."";"""";""SdYAdjW"";"""";""Seed"";""yield adjusted
weight basis"";"""";"""";""CO_366:0000007"";""Seed yield adjusted weight basis - Computing"";""Computation"";""Compute adjusted weight of
harvested seed per area."";"""";"""";""CO_366:0000192"";""kg/ha"";""Numerical"";"""";"""";"""";"""";"
";""CO_366:0000197"";""PlntLfN_Ct_lf"";"""";""Nursery evaluation" " Trial evaluation"";"""";"""";"""";""IBP" " UoN"";""Julian Pietragalla" "
Sean Mayes"";"""";""EN"";""Bambara groundnut"";""CO_366:0000194"";""Leaf number per plant"";""Morphological trait"";""The leaves number per
plant."";"""";""PlntLfN"";"""";""Plant"";""leaf number"";"""";"""";""CO_366:0000195"";""Leaf number - Counting"";""Counting"";""Count leaves in a
sampling unit (plot" plant branch etc). Usually recorded two weeks after first flowering " average number of 10
plants."";"""";"""";""CO_366:0000196"";""leaf"";""Numerical"";"""";"""";"""";"""";"
";""CO_366:0000200"";""BLng_M_mm"";"""";""Nursery evaluation" " Trial evaluation"";""Flowering"";"""";"""";""IBP" " IITA"";""Julian
Pietragalla" " Michael Abberton"";"""";""EN"";""Bambara groundnut"";""CO_366:0000198"";""Banner length"";""Morphological trait"";""The length
of the banner."";"""";""BLng"";"""";""Banner"";""length"";"""";"""";""CO_366:0000199"";""Banner length -
Measurement"";""Measurement"";""Measure the banner length and record it. Usually recorded as average length of two flowers of five
plants."";"""";"""";""CO_366:0000016"";""mm"";""Numerical"";"""";"""";"""";"""";"
";""CO_366:0000203"";""IntNodeLng_M_mm"";"""";""Trial evaluation"";""Harvest"";"""";"""";""IBP" IITA UoN " IPGRI"";""Julian
Pietragalla" Michael Abberton " Sean Mayes"";"""";""EN"";""Bambara groundnut"";""CO_366:0000201"";""Internode length"";""Morphological
trait"";""The length of internode."";"""";""IntNodeLng"";"""";""Internode"";""length"";"""";"""";""CO_366:0000202"";""Internode length -
Measurement"";""Measurement"";""Measure the internode length and record it. Usually recorded 10 weeks after planting" " average length of fourth
internode of three longest stems of five healthy plants."";"""";"""";""CO_366:0000016"";""mm"";""Numerical"";"""";"""";"""";"""";"
";""CO_366:0000206"";""PedLng_M_mm"";"""";""Nursery evaluation" " Trial evaluation"";"""";"""";"""";""IBP" IPGRI IITA "
UoN"";""Julian Pietragalla" Michael Abberton " Sean Mayes"";"""";""EN"";""Bambara groundnut"";""CO_366:0000204"";""Peduncle
length"";""Morphological trait"";""The length of peduncle."";"""";""PedLng"";"""";""Peduncle"";""length"";"""";"""";""CO_366:0000205"";""Peduncle
length - Measurement"";""Measurement"";""Measure the banner length and record it. Usually recorded at the stage of open flower" " average length of
two peduncles of five healthy plants."";"""";"""";""CO_366:0000016"";""mm"";""Numerical"";"""";"""";"""";"""";"
";""CO_366:0000209"";""PetioLng_M_mm"";"""";""Nursery evaluation" " Trial evaluation"";"""";"""";"""";""IBP" IPGRI IITA "
UoN"";""Julian Pietragalla" Michael Abberton " Sean Mayes"";"""";""EN"";""Bambara groundnut"";""CO_366:0000207"";""Petiole
length"";""Morphological trait"";""The length of the petiole."";"""";""PetioLng"";"""";""Petiole"";""length"";"""";"""";""CO_366:0000208"";""Petiole
length - Measurement"";""Measurement"";""Measure petiole length and record it. Usually recorded 10 weeks after planting" " average length of three
leaves at the fourth node of five healthy plants."";"""";"""";""CO_366:0000016"";""mm"";""Numerical"";"""";"""";"""";"""";"

";""CO_366:0000212"";""PLng_M_mm"";"""";""Trial evaluation"";""Post-Harvest"";"""";"""";""IBP"" IITA IPGRI " UoN"";""Julian Pietragalla" Michael Abberton " Sean Mayes"";"""";""EN"";""Bambara groundnut"";""CO_366:0000210"";""Pod length"";""Morphological trait"";""The length of the pod."";"""";""PLng"";"""";""Pod"";""length"";"""";"""";""CO_366:0000211"";""Pod length - Measurement"";""Measurement"";""Measure pod length and record it. Usually recorded within two months after harvest" " average length of 10 pods."";"""";"""";""CO_366:0000016"";""mm"";""Numerical"";"""";"""";"""";"""";"
";""CO_366:0000215"";""PWid_M_mm"";"""";""Trial evaluation"";""Post-Harvest"";"""";"""";""IBP"" IITA IPGRI " UoN"";""Julian Pietragalla" Michael Abberton " Sean Mayes"";"""";""EN"";""Bambara groundnut"";""CO_366:0000213"";""Pod width"";""Morphological trait"";""The width of the pod."";"""";""PWid"";"""";""Pod"";""width"";"""";"""";""CO_366:0000214"";""Pod width - Measurement"";""Measurement"";""Measure pod width and record it. Usually recorded within two months after harvest" " average width of 10 pods."";"""";"""";""CO_366:0000016"";""mm"";""Numerical"";"""";"""";"""";"""";"
";""CO_366:0000218"";""SdLng_M_mm"";"""";""Trial evaluation"";""Post-Harvest"";"""";"""";""IBP"" IITA IPGRI " UoN"";""Julian Pietragalla" Michael Abberton " Sean Mayes"";"""";""EN"";""Bambara groundnut"";""CO_366:0000216"";""Seed length"";""Morphological trait"";""The length of seeds."";"""";""SdLng"";"""";""Seed"";""length"";"""";"""";""CO_366:0000217"";""Seed length - Measurement"";""Measurement"";""Measure seeds width and record it. Usually recorded within two months after harvest" " average length of 10 seeds."";"""";"""";""CO_366:0000016"";""mm"";""Numerical"";"""";"""";"""";"""";"
";""CO_366:0000221"";""SdWid_M_mm"";"""";""Trial evaluation"";""Post-Harvest"";"""";"""";""IBP"" IITA IPGRI " UoN"";""Julian Pietragalla" Michael Abberton " Sean Mayes"";"""";""EN"";""Bambara groundnut"";""CO_366:0000219"";""Seed width"";""Morphological trait"";""The width of seeds."";"""";""SdWid"";"""";""Seed"";""width"";"""";"""";""CO_366:0000220"";""Seed width - Measurement"";""Measurement"";""Measure seeds length and record it. Usually recorded within two months after harvest" " average length of 10 seeds."";"""";"""";""CO_366:0000016"";""mm"";""Numerical"";"""";"""";"""";"""";"
";""CO_366:0000224"";""TLfltLng_M_mm"";"""";""Nursery evaluation" " Trial evaluation"";"""";"""";"""";""IBP"" IITA IPGRI " UoN"";""Julian Pietragalla" Michael Abberton " Sean Mayes"";"""";""EN"";""Bambara groundnut"";""CO_366:0000222"";""Terminal leaflet length"";""Morphological trait"";""The length of the terminal leaflet."";"""";""TLfltLng"";"""";""Terminal leaflet"";""length"";"""";"""";""CO_366:0000223"";""Terminal leaflet length - Measurement"";""Measurement"";""Measure terminal leaflet length and record it. Usually recorded 10 weeks after planting" " average length of three leaves at the fourth node of five healthy plants."";"""";"""";""CO_366:0000016"";""mm"";""Numerical"";"""";"""";"""";"""";"
";""CO_366:0000227"";""TLfltWid_M_mm"";"""";""Nursery evaluation" " Trial evaluation"";"""";"""";"""";""IBP"" IITA IPGRI " UoN"";""Julian Pietragalla" Michael Abberton " Sean Mayes"";"""";""EN"";""Bambara groundnut"";""CO_366:0000225"";""Terminal leaflet width"";""Morphological trait"";""The width of the terminal leaflet."";"""";""TLfltWid"";"""";""Terminal leaflet"";""width"";"""";"""";""CO_366:0000226"";""Terminal leaflet width - Measurement"";""Measurement"";""Measure terminal leaflet width and record it. Usually recorded 10 weeks after planting" " average width of three leaves at the fourth node of five healthy plants."";"""";"""";""CO_366:0000016"";""mm"";""Numerical"";"""";"""";"""";"""";"
";""CO_366:0000231"";""StmNodeN_Ct_node"";"""";""Trial evaluation"";""Harvest"";"""";"""";""IBP" " IPGRI"";""Julian Pietragalla"";"""";""EN"";""Bambara groundnut"";""CO_366:0000228"";""Node number per stem"";""Morphological trait"";""The nodes number per stem."";"""";""StmNodeN"";"""";""Stem"";""node number"";"""";"""";""CO_366:0000229"";""Node number - Counting"";""Counting"";""Count nodes in a stem. Usually recorded at harvest" " average number of three stems of five healthy plants."";"""";"""";""CO_366:0000230"";""node"";""Numerical"";"""";"""";"""";"""";"
";""CO_366:0000235"";""PlntPN_Ct_pod"";"""";""Trial evaluation"";""Harvest"";"""";"""";""IBP" IITA " UoN"";""Julian Pietragalla" Michael Abberton " Sean Mayes"";"""";""EN"";""Bambara groundnut"";""CO_366:0000232"";""Pod number per plant"";""Agronomical trait"";""The pod number per plant."";"""";""PlntPN"";"""";""Plant"";""pod number"";"""";"""";""CO_366:0000233"";""Pod number per plant - Counting"";""Counting"";""Count pods in a plant. Usually recorded at harvest" " average number of ten healthy plants."";"""";"""";""CO_366:0000234"";""pod"";""Numerical"";"""";"""";"""";"""";"
";""CO_366:0000239"";""PSdN_Cp_sd"";"""";""Trial evaluation"";""Post-Harvest"";"""";"""";""IBP" IITA " IPGRI"";""Julian Pietragalla" " Michael Abberton"";"""";""EN"";""Bambara groundnut"";""CO_366:0000236"";""Seed number per pod"";""Agronomical trait"";""The seed number per pod."";"""";""PSdN"";"""";""Pod"";""seed number"";"""";"""";""CO_366:0000237"";""Seed number per pod - Computation"";""Computation"";""Compute the seed number per pod."";"""";"""";""CO_366:0000238"";""seed"";""Numerical"";"""";"""";"""";"""";"
";""CO_366:0000243"";""PlntStmN_Ct_stm"";"""";""Trial evaluation"";""Harvest"";"""";"""";""IBP" IPGRI IITA " UoN"";""Julian Pietragalla" Michael Abberton " Sean Mayes"";"""";""EN"";""Bambara groundnut"";""CO_366:0000240"";""Stem number per plant"";""Morphological trait"";""The stems number per plant."";"""";""PlntStmN"";"""";""Plant"";""stem number"";"""";"""";""CO_366:0000241"";""Stem number - Counting"";""Counting"";""Count stems in a plant. Usually recorded at harvest" " average number of five healthy plants."";"""";"""";""CO_366:0000242"";""stem"";""Numerical"";"""";"""";"""";"""";"
";""CO_366:0000247"";""ShellThk_M_um"";"""";""Trial evaluation"";""Post-Harvest"";"""";"""";""IBP" IITA IPGRI " UoN"";""Julian Pietragalla" Michael Abberton " Sean Mayes"";"""";""EN"";""Bambara groundnut"";""CO_366:0000244"";""Shell thickness"";""Morphological trait"";""The thickness of the shell."";"""";""ShellThk"";"""";""Shell"";""thickness"";"""";"""";""CO_366:0000245"";""Shell thickness - Measurement"";""Measurement"";""Measure shell thickness and record it. Usually recorded within two months after harvest" " average width of 10 pods."";"""";"""";""CO_366:0000246"";""um"";""Numerical"";"""";"""";"""";"""";"
";""CO_366:0000250"";""EyeCol_E_1to19"";"""";""Trial evaluation"";"""";"""";"""";""IITA"";""Michael Abberton"";"""";""EN"";""Bambara groundnut"";""CO_366:0000248"";""Eye colour"";""Morphological trait"";""The color of the eye."";"""";""EyeCol"";"""";""Eye"";""colour"";""Standard IITA"";"""";""CO_366:0000249"";""Eye colour - Estimation"";""Estimation"";""Visual rating of the color of the eye"";"""";"""";""CO_366:0000017"";""1 to 19"";""Nominal"";"""";"""";"""";"""";""1= Black small dotted spots on brown background without"
"eye"";""2 = Dark brown small dotted spots on cream background"
"without eye"";""3 = Black and grey mottles on cream background without eye"";""4 = Black and brown mottles on cream background with grey"
"butterfly-like eye"";""5 = Black marbled spots on cream background with grey"
"butterfly-like eye"";""6 = Dark brown marbled spots on cream background with"
"grey butterfly-like eye"";""7 = Black rhomboid spots on cream background on the"
"micropylar end with grey butterfly-like eye"";""8 = Dark brown rhomboid spots on cream background on"
"the micropylar end with grey butterfly-like eye"";""9 = Black rhomboid spots on cream background on both"
micropylar and non-micropylar ends with grey
"butterfly-like eye"";""10 = Dark brown rhomboid spots on cream background on"
both micropylar and non-micropylar ends with grey
"butterfly-like eye"";"
";""CO_366:0000254"";""EyePat_E_1to12"";"""";""Trial evaluation"";"""";"""";"""";""IITA"";""Michael Abberton"";"""";""EN"";""Bambara groundnut"";""CO_366:0000251"";""Eye pattern"";""Morphological trait"";""The shape of the pigment pattern."";"""";""EyePat"";"""";""Eye"";""pattern"";""Standard IITA"";"""";""CO_366:0000252"";""Eye pattern - Estimation"";""Estimation"";""Use of diagram illustrating eye pattern (IITA Genetic Resource Center)"";"""";"""";""CO_366:0000253"";""1 to 12"";""Nominal"";"""";"""";"""";"""";""1 = Cream testa with black butterfly-like eye"";""2 = Cream testa with dark red butterfly-like eye"";""3 = Cream testa with grey butterfly-like eye"";""4 = Cream testa with black triangular eye"";""5 = Cream testa with brown triangular eye"";""6 = Cream with grey triangular eye"";""7 = Cream testa with black irregular eye"";""8 = Cream testa with grey double thick lines on both sides of"
"the eye"";""9 = Cream testa with brown circular eye"";""10 = Light brown testa with grey butterfly-like eye"";"
";""CO_366:0000258"";""CanWid_M"";"""";""Trial evaluation"";"""";"""";"""";""IITA"";""Michael Abberton"";"""";""EN"";""Bambara groundnut"";""CO_366:0000255"";""Canopy width"";""Morphological trait"";""Width of the canopy."";"""";""CanWid"";"""";""Canopy"";""width"";""Standard IITA"";""ENVO:01001242"";""CO_366:0000256"";""Canopy width -

Measurement"";""Measurement"";""Average canopy width of three/five representative plants of an accession."";"""";"""";""CO_366:0000257"";""No
scale name found"";""Numerical"";"""";"""";"""";"""";"
";""CO_366:0000262"";""GColSdTta_E"";"""";""Trial evaluation"";"""";"""";"""";""IITA"";""Michael Abberton"";"""";""EN"";""Bambara
groundnut"";""CO_366:0000259"";""Ground colour of seed testa"";""Morphological trait"";""The ground colour of seed
testa."";"""";""SdCTstacCol"";"""";""Seed coat (testa)"";""colour"";""Standard IITA"";"""";""CO_366:0000260"";""Seed coat color -
Estimation"";""Estimation"";""To be observed on seeds less than three months old"";"""";"""";""CO_366:0000261"";""No scale name
found"";""Text"";"""";"""";"""";"""";"
";""CO_366:0000266"";""PigArEye_E"";"""";""Trial evaluation"";"""";"""";"""";""IITA"";""Michael Abberton"";"""";""EN"";""Bambara
groundnut"";""CO_366:0000263"";""Pigmentation around eye"";""Morphological trait"";""The pigmentation around the
eye."";"""";""PigArEye"";"""";""Seed eye"";""pigmentation"";""Standard IITA"";"""";""CO_366:0000264"";""Pigmentation around eye -
Estimation"";""Estimation"";""Visual rating of the color around the eye"";"""";"""";""CO_366:0000265"";""No scale name
found"";""Ordinal"";"""";"""";"""";"""";"
";""CO_366:0000270"";""PodAreaN_Cp_cm2"";"""";""Trial evaluation"";"""";"""";"""";""IITA"";""Michael Abberton"";"""";""EN"";""Bambara
groundnut"";""CO_366:0000267"";""Pods number per area"";""Agronomical trait"";""The number of pods per
area."";"""";""PodAreaN"";"""";""Pods"";""number per area"";""Standard IITA"";"""";""CO_366:0000268"";""Number of pods -
Computation"";""Computation"";""Count number of pods per area"";"""";"""";""CO_366:0000269"";""m2"";""Numerical"";"""";"""";"""";"""";"
";""CO_366:0000274"";""TrifN_Ct_Lvs"";"""";""Trial evaluation"";"""";"""";"""";""IITA"";""Michael Abberton"";"""";""EN"";""Bambara
groundnut"";""CO_366:0000271"";""Trifoliate leaves number"";""Morphological trait"";""The total number of trifoliate
leaves."";"""";""TrifLvsN"";"""";""Plant leaves"";""trifoliate number"";""Standard IITA"";"""";""CO_366:0000272"";""Number of trifoliate leaves -
Counting"";""Counting"";""Number of leaves with trifoliate shape"";"""";"""";""CO_366:0000273"";""leaves"";""Nominal"";"""";"""";"""";"""";"
";""CO_366:0000278"";""OpenflwCol_E_col"";"""";""Trial evaluation"";"""";"""";"""";""IITA"";""Michael Abberton"";"""";""EN"";""Bambara
groundnut"";""CO_366:0000275"";""Open flower colour"";""Morphological trait"";""Color of the open
flowers."";"""";""OpenflwCol"";"""";""Flower"";""color"";""Standard IITA"";""PPO:0001033"   "PATO:0000014"";""CO_366:0000276"";""Open
flower color - Estimation"";""Estimation"";""Visual classification of flower colour in the experimental plot"";"""";"""";""CO_366:0000277"";""No scale
name found"";""Text"";"""";"""";"""";"""";"
";""CO_366:0000282"";""PodFill_Ct"";"""";""Trial evaluation"";"""";"""";"""";""IITA"";""Michael Abberton"";"""";""EN"";""Bambara
groundnut"";""CO_366:0000279"";""Pod fill"";""Agronomical trait"";"""";"""";""PodFill"";"""";""Pod"";""filling"";""Standard
IITA"";"""";""CO_366:0000280"";""Pod filling - Counting"";""Counting"";"""";"""";"""";""CO_366:0000281"";""No scale name
found"";""Numerical"";"""";"""";"""";"""";"
";""CO_366:0000286"";""ShellHrvst_M_Plot/kg"";"""";""Trial evaluation"";"""";"""";"""";""IITA"";""Michael Abberton"";"""";""EN"";""Bambara
groundnut"";""CO_366:0000283"";""Shelled harvest/plot(kg)"";""Agronomical trait"";"""";"""";""ShellHrvst"";"""";""Shelled
cob"";""harvest"";""Standard IITA"";"""";""CO_366:0000284"";""Shelled harvest -
Measurement"";""Measurement"";"""";"""";"""";""CO_366:0000285"";""plot/kg"";""Numerical"";"""";"""";"""";"""";"
";""CO_366:0000290"";""VgIndx_E"";"""";""Trial evaluation"";"""";"""";"""";""IITA"";""Michael Abberton"";"""";""EN"";""Bambara
groundnut"";""CO_366:0000287"";""Vigor index"";""Agronomical trait"";""The vigor of the vine and leaves of the new
plant."";"""";""VgIndx"";"""";""Plant"";""vigor index"";""Standard IITA"";"""";""CO_366:0000288"";""Vigor index -
Estimation"";""Estimation"";""Visual assessment of the plant vigour in a plot at two month after planting"";"""";"""";""CO_366:0000289"";""No scale
name found"";""Nominal"";"""";"""";"""";"""";"
";""CO_366:0000294"";""PetCol_E_col"";"""";""Trial evaluation"";"""";"""";"""";""IITA"";""Michael Abberton"";"""";""EN"";""Bambara
groundnut"";""CO_366:0000291"";""Petiole colour"";""Morphological trait"";""Colour of
petiole."";"""";""PetCol"";"""";""Petiole"";""colour"";""Standard IITA"";"""";""CO_366:0000292"";""Petiole colour -
Estimation"";""Estimation"";""Observe petiole color and rate it"";"""";"""";""CO_366:0000293"";""No scale name
found"";""Nominal"";"""";"""";"""";"""";"
";""CO_366:0000297"";""TestPat_E_1to19"";"""";""Trial evaluation"";"""";"""";"""";""IITA"";""Michael Abberton"";"""";""EN"";""Bambara
groundnut"";""CO_366:0000295"";""Testa pattern"";""Morphological trait"";""The pattern of seed
testa."";"""";""TestPat"";"""";""Testa"";""pattern"";""Standard IITA"";""FLOPO:0010985"";""CO_366:0000296"";""Testa pattern -
Estimation"";""Estimation"";""Visual assessment of the pattern of the seed testa"";"""";"""";""CO_366:0000017"";""1 to
19"";""Nominal"";"""";"""";"""";"""";""1= Black small dotted spots on brown background without"
"eye"";""2 = Dark brown small dotted spots on cream background"
"without eye"";""3 = Black and grey mottles on cream background without eye"";""4 = Black and brown mottles on cream background with grey"
"butterfly-like eye"";""5 = Black marbled spots on cream background with grey"
"butterfly-like eye"";""6 = Dark brown marbled spots on cream background with"
"grey butterfly-like eye"";""7 = Black rhomboid spots on cream background on the"
"micropylar end with grey butterfly-like eye"";""8 = Dark brown rhomboid spots on cream background on"
"the micropylar end with grey butterfly-like eye"";""9 = Black rhomboid spots on cream background on both"
micropylar and non-micropylar ends with grey
"butterfly-like eye"";""10 = Dark brown rhomboid spots on cream background on"
both micropylar and non-micropylar ends with grey
"butterfly-like eye"";"
";""CO_366:0000300"";""Flw50_Ct_day"";"""";""Trial evaluation"";"""";"""";"""";""IITA"   UoN   " IPGRI"";""Michael Abberton"   " Sean
Mayes"";"""";""EN"";""Bambara groundnut"";""CO_366:0000298"";""Days to 50% flowering"";""Phenological trait"";""Number of days to when 50% of
the plants have started flowering."";"""";""Flw50"";"""";""Plant"";""flowering time"";""Standard IITA" " UoN"";"""";""CO_366:0000299"";""Days to
50% flowering - Counting"";""Counting"";""Counting the number of days from sowing to 50% of plants started
flowering"";"""";"""";""CO_366:0000013"";""day"";""Duration"";"""";"""";"""";"""";"
";""CO_366:0000304"";""PhotoReacTpe_E_1to3"";"""";""Trial evaluation"";"""";"""";"""";""IPGRI"";"""";"""";""EN"";""Bambara
groundnut"";""CO_366:0000301"";""Photoperiodic reaction type"";""Physiological trait"";""The reaction type to a determined
photoperiod."";"""";""PhotoReacTpe"";"""";""Plant reaction"";""photoperiod type"";""Standard IPGRI"";"""";""CO_366:0000302"";""Photoperiodic
reaction type - Estimation"";""Estimation"";"""";"""";"""";""CO_366:0000303"";""1 to 3"";""Nominal"";"""";"""";"""";"""";""Short day"";""Day
neutral"";""Long day"";"
";""CO_366:0000308"";""SdCol_E_1to9"";"""";""Trial evaluation"";""Harvest"";"""";"""";""IPGRI"";"""";"""";""EN"";""Bambara
groundnut"";""CO_366:0000305"";""Seed colour"";""Morphological trait"";""The colour of the
seed."";"""";""SdCol"";"""";""Seed"";""colour"";""Standard IPGRI"";""PO:0009010"   "PATO:0000014"";""CO_366:0000306"";""Seed colour/pattern
- Estimation"";""Estimation"";""It is divided into 3 subgroups. See trait descriptor"";"""";"""";""CO_366:0000307"";""1 to
9"";""Nominal"";"""";"""";"""";"""";"
";""CO_366:0000312"";""SdCarb_Cp_pct"";"""";""Trial evaluation"";"""";"""";"""";""IPGRI"";"""";"""";""EN"";""Bambara
groundnut"";""CO_366:0000309"";""Seed carbohydrate content"";""Quality trait"";""The content of carbohydrate in the
seed."";"""";""SdCarb"";"""";""Seed"";""carbohydrate"";""Standard IPGRI"";""TO:0000291"";""CO_366:0000310"";""Carbohydrate content -
Computation"";""Computation"";"""";"""";"""";""CO_366:0000311"";""pct"";""Numerical"";"""";"""";"""";"""";"
";""CO_366:0000316"";""LTemp_E_Reac"";"""";""Trial evaluation"";"""";"""";"""";""IPGRI"";"""";"""";""EN"";""Bambara
groundnut"";""CO_366:0000313"";""Reaction to low temperature"";""Abiotic stress trait"";""The reaction to low
temperature."";"""";""Ltemp"";"""";""Low temperature"";""reaction"";""Standard IPGRI"";""NCIT:C25637"

"PATO:0001306"";""CO_366:0000314"";""Reaction to low temperature - Estimation"";""Estimation"";"""";"""";"""";""CO_366:0000315"";""No scale name found"";""Nominal"";"""";"""";"""";"""";"
";""CO_366:0000320"";""Drght_E_Reac"";"""";""Trial evaluation"";"""";"""";"""";""IPGRI"";"""";"""";""EN"";""Bambara groundnut"";""CO_366:0000317"";""Reaction to drought"";""Abiotic stress trait"";""Drought reaction."";"""";""Dro"";"""";""Drought"";""reaction"";""Standard IPGRI"";""NCIT:C25637" "ENVO:1000745"";""CO_366:0000318"";""Reaction to drought - Estimation"";""Estimation"";"""";"""";"""";""CO_366:0000319"";""No scale name found"";""Numerical"";"""";"""";"""";"""";"
";""CO_366:0000324"";""PtInterNodRatio_Cp_ratio"";"""";""Trial evaluation"";""Growth"";"""";"""";""UoN"";""Sean Mayes"";"""";""EN"";""Bambara groundnut"";""CO_366:0000321"";""Petiole-internode ratio"";""Morphological trait"";""The ratio of measurement from the petiole to internode"";"""";""PtInterNodRatio"";"""";""Petiole internode"";""ratio"";""Standard UoN"";"""";""CO_366:0000322"";""Petiole internode ratio - Computation"";""Computation"";""The ratio of measurement of the petiole to internode"";"""";"""";""CO_366:0000323"";""ratio"";""Numerical"";"""";"""";"""";"""";"
";""CO_366:0000327"";""PodDryWt_Cp_g"";"""";""Trial evaluation"";""Harvest"";"""";"""";""UoN"";""Sean Mayes"";"""";""EN"";""Bambara groundnut"";""CO_366:0000325"";""Pod dry weight"";""Agronomical trait"";""The weight of pod dry."";"""";""PodDryWt"";"""";""Plant"";""pod dry weight"";""Standard UoN"";""BTO:0001095"   NCIT:C80399 "PATO:0000128"";""CO_366:0000326"";""Pod dry weight - Computation"";""Computation"";""Average weight of pods taken from 5 plants per plot"";"""";"""";""CO_366:0000014"";""g"";""Numerical"";"""";"""";"""";"""";"
";""CO_366:0000330"";""SingSW_Cp_g"";"""";""Trial evaluation"";""Harvest"";"""";"""";""UoN"";""Sean Mayes"";"""";""EN"";""Bambara groundnut"";""CO_366:0000328"";""Single seed weight"";""Agronomical trait"";""The weight of a single seed."";"""";""SingSW"";"""";""Seed"";""single seed weight"";""Standard UoN"";""NCIT:C48440"   "TO:0000181"";""CO_366:0000329"";""Single seed weight - Computation"";""Computation"";""Average of 100 seed weight"";"""";"""";""CO_366:0000014"";""g"";""Numerical"";"""";"""";"""";"""";"
";""CO_366:0000333"";""ShW_Cp_g"";"""";""Trial evaluation"";""Harvest"";"""";"""";""UoN"";""Sean Mayes"";"""";""EN"";""Bambara groundnut"";""CO_366:0000331"";""Shell weight"";""Agronomical trait"";""The weight of the shell."";"""";""ShW"";"""";""Seed"";""shell weight"";""Standard UoN"";"""";""CO_366:0000332"";""Shell weight - Computation"";""Computation"";""100 dry pods weighed then shelled"";"""";"""";""CO_366:0000014"";""g"";""Numerical"";"""";"""";"""";"""";"
";""CO_366:0000336"";""Shell_Cp_Pct"";"""";""Trial evaluation"";""Harvest"";"""";"""";""UoN"   " IITA"";""Sean Mayes" " Michael Abberton"";"""";""EN"";""Bambara groundnut"";""CO_366:0000334"";""Shelling percentage"";""Agronomical trait"";""Average percentage of 10 pods based on weight of mature seeds."";"""";""Shell"";"""";""Seed"";""shelling %"";""Standard UoN"";"""";""CO_366:0000335"";""Shelling percentage - Computation"";""Computation"";""Average percentage of 10 pods based on weight of mature seeds (at 12% moinsture content)"";"""";"""";""CO_366:0000009"";""%"";""Numerical"";"""";""0.0"";""50.0"";"""";"
";""CO_366:0000339"";""SdWt_Cp_g"";"""";""Trial evaluation"";""harvest"";"""";"""";""UoN"";""Sean Mayes"";"""";""EN"";""Bambara groundnut"";""CO_366:0000337"";""Seed weight"";""Agronomical trait"";""The weight of the seed."";"""";""SdWt"";"""";""Seed"";""weight"";""Standard UoN"";""TO:0000181"";""CO_366:0000338"";""Seed weight - Computation"";""Computation"";""Average weight of seeds from 5 plants taken with a plot after drying"";"""";"""";""CO_366:0000014"";""g"";""Numerical"";"""";"""";"""";"""";"
";""CO_366:0000343"";""SdN_Ct_perplant"";"""";""Trial evaluation"";""harvest"";"""";"""";""UoN"";""Sean Mayes"";"""";""EN"";""Bambara groundnut"";""CO_366:0000340"";""Seed number per plant"";""Agronomical trait"";""The number of seed per plant."";"""";""SdN"";"""";""Plant"";""seed number"";""Standard UoN"";""PO:0009010" "SIO:000366"";""CO_366:0000341"";""Seed per plant - Counting"";""Counting"";""Average number of seeds taken from 5 plants per plot"";"""";"""";""CO_366:0000342"";""number"";""Numerical"";"""";"""";"""";"""";"
";""CO_366:0000346"";""ShtDWT_Cp_g"";"""";""Trial evaluation"";""harvest"";"""";"""";""UoN"";""Sean Mayes"";"""";""EN"";""Bambara groundnut"";""CO_366:0000344"";""Shoot dry weight"";""Agronomical trait"";""The weight of the dry shoot."";"""";""ShtDWT"";"""";""Shoot"";""dry weight"";""Standard UoN"";""TO:0000552"";""CO_366:0000345"";""Shoot dry weight - Computation"";""Computation"";""Weight of aboveground biomassof harvested plants"";"""";"""";""CO_366:0000014"";""g"";""Numerical"";"""";"""";"""";"""";"
";""CO_366:0000349"";""DMat_Ct_Day"";"""";""Trial evaluation"";""Full maturity"";"""";"""";""UoN"" IITA"";""Sean Mayes" " Michael Abberton"";"""";""EN"";""Bambara groundnut"";""CO_366:0000347"";""Days to maturity"";""Phenological trait"";""The number of days from sowing to full maturity"";"""";""DMat"";"""";""Plant"";""days to Maturity"";""Standard UoN" " IITA"";""TO:0000469"";""CO_366:0000348"";""Days to maturity - Counting"";""Counting"";""Number of days from sowing to full maturity"";"""";"""";""CO_366:0000013"";""day"";""Duration"";"""";"""";"""";"""";"
";""CO_366:0000352"";""SdB_M_mgkg"";"""";""Trial evaluation"";"""";"""";"""";""UoN"";""Sean Mayes"";"""";""EN"";""Bambara groundnut"";""CO_366:0000350"";""Seed boron content"";""Biochemical trait"";""The content of boron in the seed"";""Seed boron concentration"";""SdB"";"""";""Seed"";""boron"";""Standard UoN"   " IITA"";""PO:0009010"   "TO0006043"";""CO_366:0000351"";""Seed boron content - Measurement"";""Measurement"";"""";"""";"""";""CO_366:0000018"";""mg/kg"";""Numerical"";"""";"""";"""";"""";"
";""CO_366:0000355"";""SdMg_M_mgkg"";"""";""Trial evaluation"";"""";"""";"""";""UoN"";""Sean Mayes"";"""";""EN"";""Bambara groundnut"";""CO_366:0000353"";""Seed magnesium content"";""Biochemical trait"";""The content of magnesium in the seed"";""Seed magnesium concentration"";""SdMg"";"""";""Seed"";""magnesium"";""Standard UoN"   " IITA"";""PO:0009010" "TO:0006044"";""CO_366:0000354"";""Seed magnesium content - Measurement"";""Measurement"";"""";"""";"""";""CO_366:0000018"";""mg/kg"";""Numerical"";"""";"""";"""";"""";"
";""CO_366:0000358"";""SdP_M_mgkg"";"""";""Trial evaluation"";"""";"""";"""";""UoN"";""Sean Mayes"";"""";""EN"";""Bambara groundnut"";""CO_366:0000356"";""Seed phosphorus content"";""Biochemical trait"";""The content of phosphorus in the seed"";""Seed phosphorus concentration"";""SdP"";"""";""Seed"";""phosphorus"";""Standard UoN"   " IITA"";""PO:0009010"   "TO:0002666"";""CO_366:0000357"";""Seed phosphorus content - Measurement"";""Measurement"";"""";"""";"""";""CO_366:0000018"";""mg/kg"";""Numerical"";"""";"""";"""";"""";"
";""CO_366:0000361"";""SdS_M_mgkg"";"""";""Trial evaluation"";"""";"""";"""";""UoN"";""Sean Mayes"";"""";""EN"";""Bambara groundnut"";""CO_366:0000359"";""Seed sulphur content"";""Biochemical trait"";""The content of sulphur in the seed"";""Seed sulphur concentration"";""SdS"";"""";""Seed"";""sulphur"";""Standard UoN" " IITA"";"""";""CO_366:0000360"";""Seed sulphur content - Measurement"";""Measurement"";"""";"""";"""";""CO_366:0000018"";""mg/kg"";""Numerical"";"""";"""";"""";"""";"
";""CO_366:0000364"";""SdK_M_mgkg"";"""";""Trial evaluation"";"""";"""";"""";""UoN"";""Sean Mayes"";"""";""EN"";""Bambara groundnut"";""CO_366:0000362"";""Seed potassium content"";""Biochemical trait"";""The content of potassium in the seed"";""Seed potassium concentration"";""SdK"";"""";""Seed"";""potassium"";""Standard UoN"   " IITA"";""PO:0009010"   "TO:0000609"";""CO_366:0000363"";""Seed potassium content - Measurement"";""Measurement"";"""";"""";"""";""CO_366:0000018"";""mg/kg"";""Numerical"";"""";"""";"""";"""";"
";""CO_366:0000367"";""SdCa_M_mgkg"";"""";""Trial evaluation"";"""";"""";"""";""UoN"";""Sean Mayes"";"""";""EN"";""Bambara groundnut"";""CO_366:0000365"";""Seed calcium content"";""Biochemical trait"";""The content of calcium in the seed"";""Seed calcium concentration"";""SdCa"";"""";""Seed"";""calcium"";""Standard UoN"   " IITA"";"""";""CO_366:0000366"";""Seed calcium content - Measurement"";""Measurement"";"""";"""";"""";""CO_366:0000018"";""mg/kg"";""Numerical"";"""";"""";"""";"""";"
";""CO_366:0000370"";""SdTi_M_mgkg"";"""";""Trial evaluation"";"""";"""";"""";""UoN"";""Sean Mayes"";"""";""EN"";""Bambara groundnut"";""CO_366:0000368"";""Seed titanium content"";""Biochemical trait"";""The content of titanium in the seed"";""Seed titanium concentration"";""SdTi"";"""";""Seed"";""titanium"";""Standard UoN"   " IITA"";"""";""CO_366:0000369"";""Seed titanium content - Measurement"";""Measurement"";"""";"""";"""";""CO_366:0000018"";""mg/kg"";""Numerical"";"""";"""";"""";"""";"
";""CO_366:0000373"";""SdBe_M_mgkg"";"""";""Trial evaluation"";"""";"""";"""";""UoN"";""Sean Mayes"";"""";""EN"";""Bambara groundnut"";""CO_366:0000371"";""Seed beryllium content"";""Biochemical trait"";""The content of beryllium in the seed"";""Seed beryllium concentration"";""SdBe"";"""";""Seed"";""beryllium"";""Standard UoN"   " IITA"";"""";""CO_366:0000372"";""Seed beryllium content - Measurement"";""Measurement"";"""";"""";"""";""CO_366:0000018"";""mg/kg"";""Numerical"";"""";"""";"""";"""";"
";""CO_366:0000376"";""SdAl_M_mgkg"";"""";""Trial evaluation"";"""";"""";"""";""UoN"";""Sean Mayes"";"""";""EN"";""Bambara groundnut"";""CO_366:0000374"";""Seed aluminium content"";""Biochemical trait"";""The content of aluminium in the seed"";""Seed aluminium

concentration"";""SdAl"";"""";""Seed"";""aluminium"";""Standard UoN" " IITA"";"""";""CO_366:0000375"";""Seed aluminium content -
Measurement"";""Measurement"";"""";"""";"""";""CO_366:0000018"";""mg/kg"";""Numerical"";"""";"""";"""";"""";"
";""CO_366:0000379"";""SdV_M_mgkg"";"""";""Trial evaluation"";"""";"""";"""";""UoN"";""Sean Mayes"";"""";""EN"";""Bambara
groundnut"";""CO_366:0000377"";""Seed vanadium content"";""Biochemical trait"";""The content of vanadium in the seed"";""Seed vanadium
concentration"";""SdV"";"""";""Seed"";""vanadium"";""Standard UoN"   " IITA"";"""";""CO_366:0000378"";""Seed vanadium content -
Measurement"";""Measurement"";"""";"""";"""";""CO_366:0000018"";""mg/kg"";""Numerical"";"""";"""";"""";"""";"
";""CO_366:0000382"";""SdCr_M_mgkg"";"""";""Trial evaluation"";"""";"""";"""";""UoN"";""Sean Mayes"";"""";""EN"";""Bambara
groundnut"";""CO_366:0000380"";""Seed chromium content"";""Biochemical trait"";""The content of chromium in the seed"";""Seed chromium
concentration"";""SdCr"";"""";""Seed"";""chromium"";""Standard UoN"   " IITA"";""PO:0009010"   "TO:0001045"";""CO_366:0000381"";""Seed
chromium content - Measurement"";""Measurement"";"""";"""";"""";""CO_366:0000018"";""mg/kg"";""Numerical"";"""";"""";"""";"""";"
";""CO_366:0000385"";""SdMn_M_mgkg"";"""";""Trial evaluation"";"""";"""";"""";""UoN"";""Sean Mayes"";"""";""EN"";""Bambara
groundnut"";""CO_366:0000383"";""Seed manganese content"";""Biochemical trait"";""The content of manganese in the seed"";""Seed manganese
concentration"";""SdMn"";"""";""Seed"";""manganese"";""Standard UoN"" IITA"";""TO:0020091"";""CO_366:0000384"";""Seed manganese content -
Measurement"";""Measurement"";"""";"""";"""";""CO_366:0000018"";""mg/kg"";""Numerical"";"""";"""";"""";"""";"
";""CO_366:0000388"";""SdFe_M_mgkg"";"""";""Trial evaluation"";"""";"""";"""";""UoN"";""Sean Mayes"";"""";""EN"";""Bambara
groundnut"";""CO_366:0000386"";""Seed iron content"";""Biochemical trait"";""The content of iron in the seed"";""Seed iron
concentration"";""SdFe"";"""";""Seed"";""iron"";""Standard UoN"   " IITA"";"""";""CO_366:0000387"";""Seed iron content -
Measurement"";""Measurement"";"""";"""";"""";""CO_366:0000018"";""mg/kg"";""Numerical"";"""";"""";"""";"""";"
";""CO_366:0000391"";""SdCo_M_mgkg"";"""";""Trial evaluation"";"""";"""";"""";""UoN"";""Sean Mayes"";"""";""EN"";""Bambara
groundnut"";""CO_366:0000389"";""Seed cobalt content"";""Biochemical trait"";""The content of cobalt in the seed"";""Seed cobalt
concentration"";""SdCo"";"""";""Seed"";""cobalt"";""Standard UoN" " IITA"";""PO:0009010"   "TO:0006050"";""CO_366:0000390"";""Seed cobalt
content - Measurement"";""Measurement"";"""";"""";"""";""CO_366:0000018"";""mg/kg"";""Numerical"";"""";"""";"""";"""";"
";""CO_366:0000394"";""SdNi_M_mgkg"";"""";""Trial evaluation"";"""";"""";"""";""UoN"";""Sean Mayes"";"""";""EN"";""Bambara
groundnut"";""CO_366:0000392"";""Seed nickel content"";""Biochemical trait"";""The content of nickel in the seed"";""Seed nickel
concentration"";""SdNi"";"""";""Seed"";""nickel"";""Standard UoN" " IITA"";""PO:0009010"   "TO:0006051"";""CO_366:0000393"";""Seed nickel
content - Measurement"";""Measurement"";"""";"""";"""";""CO_366:0000018"";""mg/kg"";""Numerical"";"""";"""";"""";"""";"
";""CO_366:0000397"";""SdCu_M_mgkg"";"""";""Trial evaluation"";"""";"""";"""";""UoN"";""Sean Mayes"";"""";""EN"";""Bambara
groundnut"";""CO_366:0000395"";""Seed copper content"";""Biochemical trait"";""The content of cooper in the seed"";""Seed copper
concentration"";""SdCu"";"""";""Seed"";""copper"";""Standard UoN"   " IITA"";""PO:0009010"   "TO:0020092"";""CO_366:0000396"";""Seed
copper content - Measurement"";""Measurement"";"""";"""";"""";""CO_366:0000018"";""mg/kg"";""Numerical"";"""";"""";"""";"""";"
";""CO_366:0000400"";""SdZn_M_mgkg"";"""";""Trial evaluation"";"""";"""";"""";""UoN"";""Sean Mayes"";"""";""EN"";""Bambara
groundnut"";""CO_366:0000398"";""Seed zinc content"";""Biochemical trait"";""The content of zinc in the seed"";""Seed zinc
concentration"";""SdZn"";"""";""Seed"";""zinc"";""Standard UoN"   " IITA"";"""";""CO_366:0000399"";""Seed zinc content -
Measurement"";""Measurement"";"""";"""";"""";""CO_366:0000018"";""mg/kg"";""Numerical"";"""";"""";"""";"""";"
";""CO_366:0000403"";""SdGa_M_mgkg"";"""";""Trial evaluation"";"""";"""";"""";""UoN"";""Sean Mayes"";"""";""EN"";""Bambara
groundnut"";""CO_366:0000401"";""Seed gallium content"";""Biochemical trait"";""The content of gallium in the seed"";""Seed gallium
concentration"";""SdGa"";"""";""Seed"";""gallium"";""Standard UoN"   " IITA"";"""";""CO_366:0000402"";""Seed gallium content -
Measurement"";""Measurement"";"""";"""";"""";""CO_366:0000018"";""mg/kg"";""Numerical"";"""";"""";"""";"""";"
";""CO_366:0000406"";""SdAs_M_mgkg"";"""";""Trial evaluation"";"""";"""";"""";""UoN"";""Sean Mayes"";"""";""EN"";""Bambara
groundnut"";""CO_366:0000404"";""Seed arsenic content"";""Biochemical trait"";""The content of arsenic in the seed"";""Seed arsenic
concentration"";""SdAs"";"""";""Seed"";""arsenic"";""Standard UoN"   " IITA"";""PO:0009010"   "TO:0006054"";""CO_366:0000405"";""Seed
arsenic content - Measurement"";""Measurement"";"""";"""";"""";""CO_366:0000018"";""mg/kg"";""Numerical"";"""";"""";"""";"""";"
";""CO_366:0000409"";""SdSe_M_mgkg"";"""";""Trial evaluation"";"""";"""";"""";""UoN"";""Sean Mayes"";"""";""EN"";""Bambara
groundnut"";""CO_366:0000407"";""Seed selenium content"";""Biochemical trait"";""The content of selenium in the seed"";""Seed selenium
concentration"";""SdSe"";"""";""Seed"";""selenium"";""Standard UoN"   " IITA"";""PO:0009010"   "TO:0006055"";""CO_366:0000408"";""Seed
selenium content - Measurement"";""Measurement"";"""";"""";"""";""CO_366:0000018"";""mg/kg"";""Numerical"";"""";"""";"""";"""";"
";""CO_366:0000412"";""SdRb_M_mgkg"";"""";""Trial evaluation"";"""";"""";"""";""UoN"";""Sean Mayes"";"""";""EN"";""Bambara
groundnut"";""CO_366:0000410"";""Seed rubidium content"";""Biochemical trait"";""The content of rubidium in the seed"";""Seed rubidium
concentration"";""SdRb"";"""";""Seed"";""rubidium"";""Standard UoN"   " IITA"";""PO:0009010"   "TO:0001049"";""CO_366:0000411"";""Seed
rubidium content - Measurement"";""Measurement"";"""";"""";"""";""CO_366:0000018"";""mg/kg"";""Numerical"";"""";"""";"""";"""";"
";""CO_366:0000415"";""SdSr_M_mgkg"";"""";""Trial evaluation"";"""";"""";"""";""UoN"";""Sean Mayes"";"""";""EN"";""Bambara
groundnut"";""CO_366:0000413"";""Seed strontium content"";""Biochemical trait"";""The content of strontium in the seed"";""Seed strontium
concentration"";""SdSr"";"""";""Seed"";""strontium"";""Standard UoN"   " IITA"";""PO:0009010"   "TO:0001050"";""CO_366:0000414"";""Seed
strontium content - Measurement"";""Measurement"";"""";"""";"""";""CO_366:0000018"";""mg/kg"";""Numerical"";"""";"""";"""";"""";"
";""CO_366:0000418"";""SdMo_M_mgkg"";"""";""Trial evaluation"";"""";"""";"""";""UoN"";""Sean Mayes"";"""";""EN"";""Bambara
groundnut"";""CO_366:0000416"";""Seed molybdenum content"";""Biochemical trait"";""The content of molybdenum in the seed"";""Seed molybdenum
concentration"";""SdMo"";"""";""Seed"";""molybdenum"";""Standard UoN"   " IITA"";""PO:0009010"
"TO:0006056"";""CO_366:0000417"";""Seed molybdenum content -
Measurement"";""Measurement"";"""";"""";"""";""CO_366:0000018"";""mg/kg"";""Numerical"";"""";"""";"""";"""";"
";""CO_366:0000421"";""SdAg_M_mgkg"";"""";""Trial evaluation"";"""";"""";"""";""UoN"";""Sean Mayes"";"""";""EN"";""Bambara
groundnut"";""CO_366:0000419"";""Seed silver content"";""Biochemical trait"";""The content of silver in the seed"";""Seed silver
concentration"";""SdAg"";"""";""Seed"";""silver"";""Standard UoN" " IITA"";"""";""CO_366:0000420"";""Seed silver content -
Measurement"";""Measurement"";"""";"""";"""";""CO_366:0000018"";""mg/kg"";""Numerical"";"""";"""";"""";"""";"
";""CO_366:0000424"";""SdCd_M_mgkg"";"""";""Trial evaluation"";"""";"""";"""";""UoN"";""Sean Mayes"";"""";""EN"";""Bambara
groundnut"";""CO_366:0000422"";""Seed cadmium content"";""Biochemical trait"";""The content of cadmium in the seed"";""Seed cadmium
concentration"";""SdCd"";"""";""Seed"";""cadmium"";""Standard UoN"   " IITA"";""PO:0009010"   "TO:0006059"";""CO_366:0000423"";""Seed
cadmium content - Measurement"";""Measurement"";"""";"""";"""";""CO_366:0000018"";""mg/kg"";""Numerical"";"""";"""";"""";"""";"
";""CO_366:0000427"";""SdCs_M_mgkg"";"""";""Trial evaluation"";"""";"""";"""";""UoN"";""Sean Mayes"";"""";""EN"";""Bambara
groundnut"";""CO_366:0000425"";""Seed caesium content"";""Biochemical trait"";""The content of caesium in the seed"";""Seed caesium
concentration"";""SdCs"";"""";""Seed"";""caesium"";""Standard UoN"   " IITA"";"""";""CO_366:0000426"";""Seed caesium content -
Measurement"";""Measurement"";"""";"""";"""";""CO_366:0000018"";""mg/kg"";""Numerical"";"""";"""";"""";"""";"
";""CO_366:0000430"";""SdBa_M_mgkg"";"""";""Trial evaluation"";"""";"""";"""";""UoN"";""Sean Mayes"";"""";""EN"";""Bambara
groundnut"";""CO_366:0000428"";""Seed barium content"";""Biochemical trait"";""The content of barium in the seed"";""Seed barium
concentration"";""SdBa"";"""";""Seed"";""barium"";""Standard UoN"   " IITA"";""PO:0009010"   "TO:0001043"";""CO_366:0000429"";""Seed
barium content - Measurement"";""Measurement"";"""";"""";"""";""CO_366:0000018"";""mg/kg"";""Numerical"";"""";"""";"""";"""";"
";""CO_366:0000433"";""SdTl_M_mgkg"";"""";""Trial evaluation"";"""";"""";"""";""UoN"";""Sean Mayes"";"""";""EN"";""Bambara
groundnut"";""CO_366:0000431"";""Seed thallium content"";""Biochemical trait"";""The content of thallium in the seed"";""Seed thallium
concentration"";""SdTl"";"""";""Seed"";""thallium"";""Standard UoN"   " IITA"";"""";""CO_366:0000432"";""Seed thallium content -
Measurement"";""Measurement"";"""";"""";"""";""CO_366:0000018"";""mg/kg"";""Numerical"";"""";"""";"""";"""";"
";""CO_366:0000436"";""SdPb_M_mgkg"";"""";""Trial evaluation"";"""";"""";"""";""UoN"";""Sean Mayes"";"""";""EN"";""Bambara
groundnut"";""CO_366:0000434"";""Seed lead content"";""Biochemical trait"";""The content of lead in the seed"";""Seed lead

concentration"";""SdPb"";"""";""Seed"";""lead"";""Standard UoN" " IITA"";""PO:0009010" "TO:0001047"";""CO_366:0000435"";""Seed lead
content - Measurement"";""Measurement"";"""";"""";"""";""CO_366:0000018"";""mg/kg"";""Numerical"";"""";"""";"""";"""";"
";""CO_366:0000439"";""SdU_M_mgkg"";"""";""Trial evaluation"";"""";"""";"""";""UoN"";""Sean Mayes"";"""";""EN"";""Bambara
groundnut"";""CO_366:0000437"";""Seed uranium content"";""Biochemical trait"";""The content of uranium in the seed"";""Seed uranium
concentration"";""SdU"";"""";""Seed"";""uranium"";""Standard UoN" " IITA"";"""";""CO_366:0000438"";""Seed uranium content -
Measurement"";""Measurement"";"""";"""";"""";""CO_366:0000018"";""mg/kg"";""Numerical"";"""";"""";"""";"""";"
