# Supplementary material for: Challenges for FAIR-compliant description and comparison of crop phenotype data with standardized controlled vocabularies
Source: Database (Oxford). 2021 May 15;2021:baab028. doi: 10.1093/database/baab028 (PMC8122365; doi:10.1093/database/baab028)
Supplement: baab028_Supp [file baab028_supp.zip › SupplementalTable5.pdf]

	MIAPPE					
line #	MIAPPE Check list	Definition	Examples	Format	Cardinality	Associations from CropStoreDB
DM-1	**Investigation**	Investigations are research programmes with defined aims. They can exist at various scales (for example, they could encompass a grant-funded programme of work, the various components comprising a peer-reviewed publication, or a single			1 per MIAPPE submission	
DM-2	**Investigation unique ID**	Identifier comprising the unique name of the institution/database hosting the submission of the investigation data, and the accession number of the investigation in that institution.	EBI:12345678	Unique identifier	0-1	n/a
DM-3	**Investigation title**	Human-readable string summarising the investigation.	CropStoreDB	Free text (short)	1	n/a
DM-4	**Investigation description**	Human-readable text describing the investigation in more detail.	In order to maximize the impact of high quality crop science, there is a continuing need for explicit management of complex datasets relating to plant and crop genetics. We are addressing the first stage in dataset curation and dissemination, by providing a data model and tools that reflect the relationships understood and manipulated by researchers and breeders.	Free text	0-1	n/a
DM-5	**Submission date**	Date of submission of the dataset presently being described to a host repository.	2012-12-17	Date/Time (ISO 8601, optional time zone)	0-1	n/a
DM-6	**Public release date**	Date of first public release of the dataset presently being described.	2013-02-25	Date/Time (ISO 8601, optional time zone)	0-1	n/a
DM-7	**License**	License for the reuse of the data associated with this investigation. The Creative Commons licenses cover most use cases and are recommended.	CC BY-SA 4.0, Unreported	Unique identifier	0-1	n/a
DM-8	**MIAPPE version**	The version of MIAPPE used.	1.1	Version number	1	1.1
DM-9	**Associated publication**	An identifier for a literature publication where the investigation is described. Use of DOIs is recommended.	https://doi.org/10.12688/f1000research.11301.2	DOI	0+	n/a
DM-10	**Study**	A study (or experiment) comprises a series of assays (or measurements) of one or more types, undertaken to answer a particular biological question.			1+ per investigation	
DM-11	**Study unique ID**	Unique identifier comprising the name or identifier for the institution/database hosting the submission of the study data, and the identifier of the study in that institution.	http://www.cropstoredb.org/	Unique identifier	0-1	plant_trials.plant_trial_id
DM-12	**Study title**	Human-readable text summarising the study	Introducing the Brassica Information Portal: Towards integrating genotypic and phenotypic Brassica crop data.	Free text (short)	1	plant_trials.project_descriptor
DM-13	**Study description**	Human-readable text describing the study	There are four input workbook corresponding to the three primary modules of the database (cs_plants (including traits), cs_linkage_maps (including map loci, QTL), cs_markers (including alleles and genotypes) and one for auxiliary/additional data tables. Each worksheet corresponds directly to a SINGLE table within the database, and each column a field. Below we provide guidelines on how to use these input templates	Free text	0-1	plant_trials.plant_trial_description
DM-14	**Start date of study**	Date and, if relevant, time when the experiment started	2/04/2014	Date/Time (ISO 8601, optional time zone)	1	plant_trials.trial_year
DM-15	**End date of study**	Date and, if relevant, time when the experiment ended		Date/Time (ISO 8601, optional time zone)	0-1	n/a
DM-16	**Contact institution**	Name and address of the institution responsible for the study.	UoN, CFF	Free text (short)	1	plant_trial.institute_id
DM-17	**Geographic location (country)**	The country where the experiment took place, either as a full name or preferably as a 2-letter code.	UK, Africa	Country name or 2-letter code (ISO 3166)	1	plant_trial.trial_location
DM-18	**Experimental site name**	The name of the natural site, experimental field, greenhouse, phenotyping facility, etc. where the experiment took place.	Agronomy Bay, UK, Notwane Farm, Gabarone, Botswana	Free text (short)	1	plant_trials.trial_location_site_name
DM-19	**Geographic location (latitude)**	Latitude of the experimental site in degrees, in decimal format.		Degrees in the decimal format (ISO 6709)	0-1 (1 if longitude is provided)	plant_trials.latitude
DM-20	**Geographic location (longitude)**	Longitute of the experimental site in degrees, in decimal format.		Degrees in the decimal format (ISO 6709)	0-1 (1 if latitude is provided)	plant_trials.longitude
DM-21	**Geographic location (altitude)**	Altitude of the experimental site, provided in metres (m).		Numeric + unit abbreviation	0-1	plant_trials.altitude
DM-22	**Description of the experimental design**	Short description of the experimental design, possibly including statistical design. In specific cases, e.g. legacy datasets or data computed from several studies, the experimental design can be "unknown"/"NA", "aggregated/reduced data", or simply 'none'.	Glass house experiments in Agronomy Bay, UK to study morphological and genetic studies of 35 landraces. Each landrace was planted in replicates of 3. Field trials in Gabaone, Botswana to study morhological and genetic diversity of 31 single genotype plants. Each plant was picked from one of three plants (A,B,C) from the lines tested in Agronomy Bay	Free text	1	plant_trials.design_type, plant_trials.statistical_factors, plant_trials.design_factors, plant_trials.design_layout_matrix
DM-23	**Type of experimental design**	Type of experimental design of the study, in the form of an accession number from the Crop Ontology.	CO_715:0000145	Crop Ontology term (subclass of "CO_715:0000003")	0-1	plant_trials.design_type
DM-24	**Observation unit level hierarchy**	Hierarchy of the different levels of repetitions between each others	block>rep>plot	Formatted text (level>level)	0-1	plant_trials.design_factors
DM-25	**Observation unit description**	General description of the observation units in the study.	Observation units consisted in individual plots themselves consisting of a row of 15 plants at a density of approximately six plants per square meter.<br>NA	Free text	1	n/a
DM-26	**Description of growth facility**	Short description of the facility in which the study was carried out.	Glass house condition and field environment condition	Free text (short)	1	plant_trials.plant_trial_descriptor
DM-27	**Type of growth facility**	Type of growth facility in which the study was carried out, in the form of an accession number from the Crop Ontology. Same as before	CO_715:0000162	Crop Ontology term (subclass of "CO_715:0000005")	0-1	n/a
DM-28	**Cultural practices**	General description of the cultural practices of the study.	Irrigation was applied according needs during summer to prevent water stress.	Free text	0-1	n/a
DM-29	**Map of experimental design**	Representation of the experimental design.	https://urgi.versailles.inra.fr/files/ephesis/181000503/181000503_plan.xls	URL or File name (of gis or tabular file like csv or tsv)	0+	plant_trials.design_layout_matrix
DM-30	**Person**	A human involved in the investigation or specifically any of its studies.			1+ per investigation / 0+ per study	
DM-31	**Person name**	The name of the person (either full name or as used in scientific publications)	Sean Mayes, Razlin Azman	Name	1	cs_people.full_name
DM-32	**Person email**	The electronic mail address of the person.	sean.mayes@cffresearch.org, razlir	email address	0-1	cs_people.person_id
DM-33	**Person ID**	An identifier for the data submitter. If that submitter is an individual, ORCID identifiers are recommended.	orcid.org/0000-0003-4770-3662	Unique identifier	0-1	cs_people.person_id
DM-34	**Person role**	Type of contribution of the person to the investigation	author; data submitter; described by whom	Free text (short)	1+	cs_people.role
DM-35	**Person affiliation**	The institution the person belongs to	Associate Professor, UoN. Research Theme Leader/Programme Director, CFF.	Free text (short)	1+	cs_people.current_institution_id
DM-36	**Data File**	A file or digital object holding observation data recorded during one or more assays of the study, typically in tabular form. Multiple data files may be provided per study, and each file can include observations for several observation units and			0+ per study	

DM-37	**Data file link**	Link to the data file (or digital object) in a public database or in a persistant institutional repository; or identifier of the data file when submitted together with the MIAPPE submission.	http://www.cropstoredb.org/	URL or File name	1	n/a
DM-38	**Data file description**	Description of the format of the data file. May be a standard file format name, or a description of organization of the data in a tabular file.	FASTA<br>tab-delimited<br>column headers headers: 1. A 2. B 3. C	Free text (short)	1	n/a
DM-39	**Data file version**	The version of the dataset (the actual data).		Software version number	1	n/a
DM-40	**Biological Material**	The biological material being studied (e.g. plants grown from a certain bag or seed, or plants grown in a particular field). The original source of that material (e.g., the seeds or the original plant cloned) is called the material source, which, when held by			1+ per study;<br>0+ per observation unit	
DM-41	**Biological material ID**	Code used to identify the biological material in the data file. Should be unique within the Investigation. Can correspond to experimental plant ID, seed lot ID, etc… This material identification is different from a BiosampleID which corresponds to Observation Unit or Samples sections below.	su_NOTT_08_0030009_01	Unique identifier	1	plant_scoring_units.scoring_unit_id
DM-42	**Organism**	An identifier for the organism at the species level. Use of the NCBI taxon ID is recommended.	NCBI:115715	Unique identifier	1	plant_line.genus, plant_line.species
DM-43	**Genus**	Genus name for the organism under study, according to standard scientific nomenclature.	Vigna	Genus name	0-1	plant_line.genus
DM-44	**Species**	Species name (formally: specific epithet) for the organism under study, according to standard scientific nomenclature.	Vigna subterranea	Species name	0-1	plant_line.species
DM-44'	**Infraspecific name**	Name of any subtaxa level, including variety, crossing name, etc. It can be used to store any additional taxonomic identifier. Either free text description or key-value pair list format (the key is the name of the rank and the value is the value of the rank). Ranks can be among the following terms: subspecies, cultivar, variety, subvariety, convariety, group, subgroup, hybrid, line, form, subform. For MCPD compliance, the following abbreviations are allowed: 'subsp.' (subspecies); 'convar.' (convariety); 'var.' (variety); 'f.' (form); 'Group' (cultivar group).		Free text, or key-value pair list, or MCPD-compliant format	0-1	plant_line.subtaxa
DM-45	**Biological material latitude**	Latitude of the studied biological material. [Alternative identifier for in situ material]	+39.067	Degrees in the decimal format (ISO 6709)	0-1 (1 if longitude is provided)	plant_accessions.latitude
DM-46	**Biological material longitude**	Longitude of the studied biological material. [Alternative identifier for in situ material]	-8.73	Degrees in the decimal format (ISO 6709)	0-1 (1 if latitude is provided)	plant_accessions.longitud
DM-47	**Biological material altitude**	Altitude of the studied biological material, provided in meters (m). [Alternative identifier for in situ material]		Numeric + unit abbreviation	0-1	plant_accessions.elevation
DM-48	**Biological material coordinates uncertainty**	Circular uncertainty of the coordinates, preferably provided in meters (m). [Alternative identifier for in situ material]	200 m	Numeric	0-1	n/a
DM-49	**Biological material preprocessing**	Description of any process or treatment applied uniformely to the biological material, prior to the study itself. Can be provided as free text or as an accession number from a suitable controlled vocabulary.	EO:0007210 - PVY(NTN); transplanted from study http://phenome-fppn.fr/maugio/2013/t2351 observation unit ID: pot:894	Plant Environment Ontology and/or free text	0+	trait_descriptors.materials
DM-50	**Material source ID (Holding institute/stock centre, accession)**	An identifier for the source of the biological material, in the form of a key-value pair comprising the name/identifer of the repository from which the material was sourced plus the accession number of the repository for that material. Where an accession number has not been assigned, but the material has been derived from the crossing of known accessions, the material can be defined as follows: "mother_accession X father_accession", or, if father is unknown, as "mother_accession X UNKNOWN". For in situ material, the region of provenance may be used when an accession is not available.	INRA:W95115_inra<br>ICNF:PNB-RPI	Unique identifier	0-1	plant_trials.institute_id:plant_accessions.plant_accession
DM-51	**Material source DOI**	Digital Object Identifier (DOI) of the material source	doi:10.15454/1.4658436467893904E12	DOI	0-1	n/a
DM-52	**Material source latitude**	Latitude of the material source. [Alternative identifier for in situ material]	+39.067	Degrees in the decimal format (ISO 6709)	0-1 (1 if longitude is provided)	n/a
DM-53	**Material source longitude**	Longitude of the material source. [Alternative identifier for in situ material]	-8.73	Degrees in the decimal format (ISO 6709)	0-1 (1 if latitude is provided)	n/a
DM-54	**Material source altitude**	Altitude of the material source, provided in metres (m). [Alternative identifier for in situ material]	10 m	Numeric + unit abbreviation	0-1	n/a
DM-55	**Material source coordinates uncertainty**	Circular uncertainty of the coordinates, provided in meters (m). [Alternative identifier for in situ material]	Latitude:+2.341; row:4 ; X:3; Y:6; Xm:35; Ym:65; Block:1; Plot:894	Numeric + unit abbreviation	0-1	n/a
DM-56	**Material source description**	Description of the material source	Each landrace was planted in replicates of 3. Each plant was picked from one of three plants (A,B,C) from the lines tested in Agronomy Bay.	Free text	0-1	n/a
DM-57	**Environment**	Environmental parameters that were kept constant throughout the study and did not change betweeen observation units or assays. Environment characteristics that vary over time, i.e. environmental variables, should be recorded as Observed			0-1 per study	
DM-58	**Environment parameter**	Name of the environment parameter constant within the experiment.	sowing density<br>rooting medium composition; pH	Free text (see Appendix I)	1+	n/a
DM-59	**Environment parameter value**	Value of the environment parameter (defined above) constant within the experiment.	300 seeds per m2<br>Clay 50% plus sand; 6.5	Free text	1 per parameter	n/a
DM-60	**Experimental Factor**	The object of a study is to ascertain the impact of one or more factors on the biological material. Thus, a factor is, by definition a condition that varies between observation units, which may be biotic (pest, disease interaction) or abiotic			0+ per study;<br>0+ per observation unit	
DM-61	**Experimental Factor type**	Name/Acronym of the experimental factor.	Watering	Free text (see Appendix II)	1	n/a
DM-62	**Experimental Factor description**	Free text description of the experimental factor. This include all relevant treatments planification and protocol planed for all the plant targeted by a given experimental factor.	Daily watering 1 L per plant.	Free text	0-1	n/a
DM-63	**Experimental Factor values**	List of possible values for the factor.	Watered; Unwatered	Free text	2+ per factor	n/a
DM-64	**Event**	An event is discrete occurrence at a particular time in the experiment (which can be natural, such as rain, or unnatural, such as planting, watering, etc). Events may be the realization of Factors or parts of Factors, or may be confounding to Factors.			0+ per study/observation unit	
DM-65	**Event type**	Short name of the event.		Free text (short)	1	occasions
DM-66	**Event acession number**	Accession number of the event type in a suitable controlled vocabulary (Crop Ontology).		Crop Ontology term (subclass of CO_715:0000006)	0-1	n/a
DM-67	**Event description**	Description of the event, including details such as amount applied and possibly duration of the event.	Sowing using seed drill<br>Fertilizer application: Ammonium nitrate at 3 kg/m2	Free text	0-1	n/a
DM-68	**Event date**	Date and time of the event.	9/04/2014, 2/04/2014	Date/Time (ISO 8601, optional time zone)	1+	occasions.start_date,occasions.end_date
DM-69	**Observation Unit**	Observation units are objects that are subject to particular instances of observsation and measurement. An observation unit comprises one or more plants, and their environment. Synonym : Experimental unit.			1+ per study	
DM-70	**Observation unit ID**	Identifier used to identify the observation unit in data files containing the values observed or measured on that unit. Must be locally unique.	plot:894	Unique identifier	1	plant_scoring_units.scoring_unit_id

DM-71	**Observation unit type**	Type of observation unit in textual form, usually one of the following: block, sub-block, plot, plant, trial, pot, replication or replicate, individual, virtual_trial, unit-parcel	Glass house condition and field environment condition	Free text	1	plant_scoring_units.number_units_scored, plant_scoring_units.scoring_unit_sample_size, plant_scoring_units.scoring_unit_frame_size
DM-72	**External ID**	Identifier for the observation unit in a persistant repository, comprises the name of the repository and the identifier of the observation unit therein. The EBI Biosamples repository can be used. URI are recommended when possible.	Biosamples:SAMEA4202911	Unique identifier	0+	n/a
DM-73	**Spatial distribution**	Type and value of a spatial coordinate (georeference or relative) or level of observation (plot 45, subblock 7, block 2) provided as a key-value pair of the form type:value. Levels of observation must be consistent with those listed in the Study section.	Latitude:+2.341; row:4 ; X:3; Y:6; Xm:35; Ym:65; Block:1; Plot:894	Formatted text (Key:value)	0+	plant_scoring_units.scoring_unit_frame_size, may also relates to design factors
DM-74	**Observation Unit factor value**	List of values for each factor applied to the observation unit.	Watered	Free text	0+	design_factors.*
DM-75	**Sample**	A sample is a portion of plant tissue extracted from an observation unit for the purpose of sub-plant observations and/or molecular studies. A sample must be used when there is a physical sample that needs to be stored and traced. Otherwise,			0+ per observation unit	
DM-76	**Sample ID**	Unique identifier for the sample.	Trait_descriptor_id	Unique identifier	1	plant_scoring_units.scoring_unit_id
DM-77	**Plant structure development stage**	The stage in the life of a plant structure during which the sample was taken, in the form of an accession number to a suitable controlled vocabulary (Plant Ontology, BBCH scale)	PO:0025340 gametophyte vegetative stage	Plant Ontology term (subclass or PO:0009012) or BBCH scale term	0-1	trait_descriptors.stage_score
DM-78	**Plant anatomical entity**	A description of the plant part (e.g. leaf) or the plant product (e.g. resin) from which the sample was taken, in the form of an accession number to a suitable controlled vocabulary (Plant Ontology).	PO:0009025, TO:0000766, PO:0009053, PO:0009010, CO_343:0000072	Plant Ontology term (subclass of PO:0025131)	1	plant_scoring_units.scored_plant_part
DM-79	**Sample description**	Any information not captured by the other sample fields, including quantification, sample treatments and processing.	trait_description	Free text	0-1	trait_descriptors.materials
DM-80	**Collection date**	The date and time when the sample was collected / harvested	2/04/2014, 9/04/2014	Date/Time	1	n/a
DM-81	**External ID**	An identifier for the sample in a persistant repository, comprising the name of the repository and the accession number of the observation unit therein. Submission to the EBI Biosamples repository is recommended. URI are recommended when possible.	Biosamples:SAMEA4202911	Unique identifier	0+	n/a
DM-82	**Observed Variable**	An observed variable describes how a measurement has been made. It typically takes the form of a measured characteristic of the observation unit (plant or environmental trait), associated to the method and unit of measurement.			1+ per study	
DM-83	**Variable ID**	Code used to identify the variable in the data file. We recommend using a variable definition from the Crop Ontology where possible. Otherwise, the Crop Ontology naming convention is recommended: <trait abbreviation>_<method abbreviation>_<scale abbreviation>). A variable ID must be unique within a given investigation.	TLfltLng_M_mm	Unique identifier	1	trait_descriptors.trait_descriptor_id
DM-84	**Variable name**	Name of the variable.		Free text	0-1	n/a
DM-85	**Variable accession number**	Accession number of the variable in the Crop Ontology		Crop Ontology term	0-1	n/a
DM-86	**Trait**	Name of the (plant or environmental) trait under observation		Free text	1	trait_descriptors.descriptor_name
DM-87	**Trait accession number**	Accession number of the trait in a suitable controlled vocabulary (Crop Ontology, Trait Ontology).		Term from Plant Trait Ontology, Crop Ontology, or XML Environment Ontology	0-1	n/a
DM-88	**Method**	Name of the method of observation	Measurement	Free text	1	trait_descriptors.scoring_method
DM-89	**Method accession number**	Accession number of the method in a suitable controlled vocabulary (Crop Ontology, Trait Ontology).		Term from Plant Trait Ontology, Crop Ontology, or XML Environment Ontology	0-1	n/a
DM-90	**Method description**	Textual description of the method, which may extend a method defined in an external reference with specific parameters, e.g. growth stage, inoculation precise organ (leaf number)	Length of median leaflet at the fourth internode	Free text	0-1	trait_descriptors.scoring_method
DM-91	**Reference associated to the method**	URI/DOI of reference describing the method.		URI or DOI	0-1	trait_descriptors.
DM-92	**Scale**	Name of the scale associated with the variable	mm	Unique identifier	1	trait_descriptors.units_of_measurements, trait_grades.trait_grade
DM-93	**Scale accession number**	Accession number of the scale in a suitable controlled vocabulary (Crop Ontology).		Crop Ontology term	0-1	n/a
DM-94	**Time scale**	Name of the scale or unit of time with which observations of this type were recorded in the data file (for time series studies).	Numerical	Free text	0+	trait_de+G2:G97scriptors.timing
